# Impaired Aggrephagy, Interrupted Vesicular Trafficking, and Cellular Stress, Lead to Protein Aggregation, and Synaptic Dysfunction in Cerebellum of Children and Adults with Idiopathic Autism

**DOI:** 10.1007/s12311-025-01880-5

**Published:** 2025-08-08

**Authors:** S. Hossein Fatemi, Timothy D. Folsom, Arthur Eschenlauer, Thierry Chekouo

**Affiliations:** 1 Department of Psychiatry and Behavioral Sciences, University of Minnesota Medical School, 420 Delaware Street SE, Minneapolis, MN 55455 USA; 2 Department of Pediatrics, University of Minnesota Medical School, Minneapolis, MN 55455 USA; 3 Masonic Cancer Center, University of Minnesota Medical School, Minneapolis, MN 55455 USA; 4 Minnesota Supercomputing Institute, 599 Walter Library, 117 Pleasant Street, Minneapolis, MN 55455 USA; 5 Division of Biostatistics and Health Data Science, School of Public Health, University of Minnesota, Minneapolis, MN 55455 USA

**Keywords:** Autism, Cerebellum, Proteomics, Proteostasis, Vesicular Transport, Protein Misfolding, Aggrephagy, Macroautophagy

## Abstract

Autism spectrum disorder (ASD) is a debilitating neurodevelopmental disorder with genetic and environmental etiologies involving several brain areas exhibiting abnormalities of cognition and social behavior. Previous work showed involvement of synaptic abnormalities in dorsolateral prefrontal cortex [1]. We hypothesized whether similar synaptic proteins were involved in pathology of cerebellar vermis of children and adults with ASD. Subcellular fractions of synaptosomes from cerebellar vermal cortices of age-, brain area-, and postmortem-interval-matched samples from children and adults with idiopathic ASD vs. controls were subjected to HPLC-tandem mass spectrometry. Analysis of proteomic data in cerebellar vermis of children with ASD showed enrichment of significantly downregulated pathways and proteins (FDR-adjusted *p* < 0.05) involved in protein folding, Rho GTPase cycle, aggrephagy, macroautophagy, anterograde and retrograde transport, proteinopathy, protein stability, and cell response to stress. Enrichment of significantly upregulated pathways and proteins (FDR-adjusted *p* < 0.05) involved processes of glycolysis, gluconeogenesis, metabolism of amino acids, and degradation of lysine, fatty acids, valine, leucine, and isoleucine. Analysis of proteomic data in cerebellar vermis of adults with ASD showed enrichment of significantly downregulated pathways and proteins (FDR-adjusted *p* < 0.05) involved in aggrephagy, COPI-mediated anterograde transport and COPI-independent Golgi-to-ER retrograde transport, endocytosis, presynaptic, postsynaptic, and PSD related vesicle mediated activities, serotonin and dopamine neurotransmitter release, and neurodegeneration-related diseases. Enrichment of significantly upregulated pathways and proteins (FDR-adjusted *p* < 0.05) in adults with ASD included peptide cross-linking, amyloidosis, intermediate filament organization, citrullination, methylation, and proteolysis. Overall, the proteomic data support the concept that cerebellar abnormalities in synaptic structure and function begin during fetal cerebellar development [2], culminate in early childhood, and evolve into adulthood, consistent with pathologic involvement of genes subserving the cognitive domains in ASD.

**Supplementary Information:**

The online version contains supplementary material available at 10.1007/s12311-025-01880-5.

## Introduction

Autism is a debilitating neurodevelopmental disorder with a rising prevalence and heterogeneous etiology characterized by deficits in communication, cognition, and behavior [3]. The genetic data point to a large set of genes involved in the etiopathology of autism [4]. Brain structural abnormalities involve prefrontal cortex [1], parietal cortex, amygdala, hippocampus, and cerebellum [5, 6].

The human cerebellum undergoes a highly protracted timetable of growth from early first trimester to the end of the second postnatal year [7]. Structurally, it is composed of the granular layer, the Purkinje layer and the molecular layer. Histologically, cerebellar tissue consists of inhibitory cell types [Purkinje cells (PCs), Golgi cells, stellate cells, basket cells, Lugaro cells], excitatory cell types [granular cells (GC), unipolar brush cells], and glial cells [7]. Cerebellar developmental stages include neuronal proliferation, migration, differentiation, axonal growth, synaptogenesis and pruning [7]. Human cerebellar volume increases from birth to a peak of growth at age 11.8 years in females and 15.6 years in males [2], correlating with improvement in cognitive functions during childhood and adolescence [2]. Many factors can interfere with the continuity and timetable of growth during the development of the cerebellar cortex including genetic and immune-related abnormalities that may lead to the development of dysfunctional circuitry in human cerebellum [7, 8].

Recent emerging evidence points to involvement of cerebellum in cognition, especially as related to etiology of autism and ataxias [7, 8], A recent proteomic analysis of posterior inferior cerebellum showed abnormalities in several pre-and postsynaptic proteins in subjects with autism [9]. Additionally, Broek et al. [10] reported significant alterations in several markers of myelination, synaptic vesicle release, and astrocytic maturation in lateral posterior and anterior cerebellum of subjects with autism. However, both previous proteomic studies were limited due to lack of age stratification and use of cerebellar areas not frequently associated with pathology in ASD [9, 10]. A more recent publication [11] has compared RNA sequence data (mRNA) in age-stratified cerebellar tissues (young vs. adult), clearly exhibiting age-dependent changes in transcriptomic data in ASD. However, comparison of these novel data to ours, shows limited data similarity as their work presents RNA data vs. our novel proteomic data as presented here. Thus, as there continues to be a dearth of published proteomic studies focusing on cerebellar pathology in children and adults with autism, we performed a quantitative proteomic study focusing on synaptic proteome in age-stratified subjects with idiopathic autism and in a pathologic site, i.e. vermis, which is frequently associated with pathology of autism [12].

## Materials and Methods

### Human Brain Procurement

All experimental procedures were approved by the Institutional Review Board of the University of Minnesota School of Medicine. Postmortem blocks of cerebellar vermis (Table 1) were obtained from the Autism Research Foundation and their affiliated brain banks (NICHD Brain and Tissue Bank for Developmental Disorders at the University of Maryland; the Harvard Brain Tissues Research Center; and the Autism Tissue Program). These cerebellar vermal samples, which have been used by our laboratory previously [12], are some of the most well-characterized and most-studied brain collections employed by multiple laboratories. Prior to freezing, brains were sectioned in half, dissected, and stored at -80 °C until further use. Consent from next of kin was given to the respective institutions. DSM-IV diagnoses were established prior to death by clinicians using all demographic and clinical data from available medical records from family interviews. Samples were matched for age, brain area, and postmortem interval [PMI]. All demographic information is listed in Table 1. None of the control samples had any history of neuropsychiatric disorders, seizure disorder, or intellectual disabilities. Each brain sample included both gray and white matter. The tissue samples were prepared for subcellular fractionation and proteomics as previously described and detailed previously [1, 13–15].

**Table 1:** Clinical characteristics of cerebellar vermis tissues in children and adults with idiopathic autism

Group	Children		Adults	
	Autism	Control	Autism	Control
Total *N*	5	5	5	5
Ethnicity	2AA:2 C:1 A	1AA:4 C	1AA:4 C	2AA:2U:1 C
Age	11.4 ± 3.2	9.2 ± 3.86	29.4 ± 4.75	32.6 ± 7.0
Sex	4 M:1 F	5 M	4 M:1 F	3 M:2 F
PMI	14.77 ± 5.12	19.6 ± 8.4	26.48 ± 8.90	21.46 ± 9.06

Comparison of autistic and control values were statistically nonsignificant using an unpaired T-test. A, Asian; AA, African American; C, Caucasian; U, unknown ethnicity; F, female; M, male

### Subcellular Fractionation

Human cerebellar vermis samples (*n* = 5 per group, 20 per brain area) were subjected to homogenization and subcellular fractionation as previously described [13]. Cerebellar samples were incubated for 4 min on ice in 1**x** isotonic extraction buffer (10-mM HEPES, 250-mM sucrose, 25-mM KCl, 1-mM EGTA, pH 7.8) plus protease inhibitors (PI) at a volume of 3**x** the weight of the sample. Subsequently, cerebellar tissue was homogenized four times (30 s each) using a motorized pestle. Following homogenization, a 60-µL aliquot was saved as total homogenate. The remaining homogenate underwent a low-speed centrifugation (700**x** g) (5415D centrifuge, Eppendorf North America, Hauppauge, NY, USA) for 10 min at 4 °C to separate the intact nuclei and heavy membranes (pellet 1) from the supernatant (supernatant 1). Next, supernatant 1 was centrifuged at 15,000**x** g for 10 min at 4 °C, to separate the crude membrane fraction (pellet 2) from the supernatant (supernatant 2). Pellet 2 was reconstituted in sucrose homogenization buffer and added to ultracentrifuge tubes containing 3 mL of Triton X-100 buffer (10-mM Tris–HCl, 1-mM Na_3_PO_4_, 5-mM NaF, 1-mM EDTA, 1-mM EGTA, 0.5% v/v Triton X-100, pH 7.4, + PI). A 30,000**x** g centrifugation step for 20 min (Optima L-90 K Ultracentrifuge, Beckman-Coulter, Indianapolis, IN, USA) at 4 °C resulted in a Triton-insoluble pellet (pellet 3) constituting the synaptic fraction. The synaptic fraction was then reconstituted in 40 µL of PBS + PI. Protein levels for synaptic fractions were determined using a Bradford assay and samples were stored at -80 °C until proteomic analysis. Subsequent proteomic studies were performed on age-stratified groups (control and ASD children vs. control and ASD adults) separately as described below.

### Orbitrap Fusion Liquid Chromatography-Mass Spectrometry Analysis

All procedures are as described previously [1]. Following reconstitution of the dried peptide fractions in 97.9:2.0:0.1 H_2_O: acetonitrile (ACN): formic acid (FA), each sample was labeled with a specific label from the Thermo Scientific TMTpro™ 16plex Label Reagent Set, and samples were pooled and then analyzed by capillary LC–MS with a Thermo Fisher Scientific (Waltham, MA) Dionex UltiMate 3,000 system in-line with Orbitrap Fusion mass spectrometer (Thermo Fisher Scientific). Peptides were loaded directly on-column in solvent A (99.9:0.1 H_2_O: FA) at maximum pressure (800 bar). Peptides were separated on a self-packed C18 column (Dr Maisch GmbH ReproSil-PUR) 1.9 μm, 120 A C18aq, 100 μm ID **x** 30-cm length at 55 °C with a biphasic gradient starting at 5% solvent B (99.9:0.1 ACN: FA) at a flow rate of 400 nl/min. The starting conditions were held for 2 min and then the gradient increased to 8% solvent B by 2.5 min. The flow was reduced to 315 nl/min and the gradient increased to 22% solvent B by 62 min and to 45% solvent B by 75 min. Finally, the gradient was increased to 90% solvent B by 77 min with a flowrate of 400 nl/min and held to 83 min followed by a return to starting conditions at 5% solvent B at 85 min and held to 92 min. Then, a top 12 data dependent acquisition method was utilized with the following MS parameters: ESI voltage 2.1 kV, ion transfer tube 275 °C; Orbitrap MS1 scan 120-k resolution in profile mode from 380 to 1580 *m*/*z* with 100-ms maximum injection time, 100% (4e^5^) automatic gain control (AGC); MS2 triggered on the top 12 most abundant ions above 5e^4^ counts, 1.6-Da quadrupole isolation window, fixed HCD activation with 40% collision energy, Orbitrap detection with 60-K resolution at 200 *m*/*z*, first mass fixed at 122 *m*/*z*, 150-ms max injection time, 1e^6^ AGC, and 40-s dynamic exclusion duration with ± 10 ppm mass tolerance.

### Database Search

Peptide tandem MS data were processed using Sequest [Thermo Fisher Scientific, San Jose, CA, in Proteome Discoverer (PD) 2.5]. The human (taxonID 9606) Universal Proteome (UP000005640) target protein sequence database was downloaded from UniProt (www.uniprot.org/) on 2019 July 12 and merged with a common lab contaminant protein database (http://www.thegpm.org/cRAP/index.html); the number of protein sequences was 74,234 sequences. Peptide search parameters specified trypsin digestion with a maximum of two missed cleavage sites, fragment ion mass tolerance 0.05 Da, and precursor tolerance 15 ppm. Variable modifications were set for the oxidation of methionine, pyroglutamic acid conversion from glutamine, deamidation of asparagine, acetyl and/or met-loss of the protein N-terminus, and TMT10plex of lysine and peptide N-terminus. Carbamidomethyl of cysteine was specified as a fixed modification.

### Criteria for Protein Identification

1% protein and peptide FDR filters were applied using the Percolator Algorithm [16] in PD.

### Protein Quantification

PD for TMT-based protein quantification was run with the following parameters: unique and razor peptides were included, shared peptides were excluded, impurity corrections were applied, co-isolation threshold maximum was 50%, normalization was performed on the total peptide amount; protein ratio calculations were performed using pairwise ratio-based mode; and hypothesis testing was performed using the background t-test approach. PD employed the Benjamini–Hochberg FDR procedure to control for errors associated with multiple hypothesis tests [17].

### Statistical and Bioinformatic Analyses of Proteomic Data

Peptide quantification data were exported from PD software and imported into the R statistical programming language environment [18]. The peptide quantitations were used for a univariate approach; for each protein, a linear mixed model (LMM) was fitted to its quantitated, unique peptides; the LMM assumed correlation among those peptide levels and used the disease condition (autism) as a covariate. We repeated these analyses for children and adults respectively which allowed us to estimate autism main effects (via log_2_ FC calculation) within each age group and for each identified protein. To account for multiple hypothesis testing, the Benjamini–Hochberg FDR control procedure [17] was used to adjust the *P*-values obtained for the effect of disease status; these adjusted *P*-values were used as the criterion for assessments of significance of relative expression for individual proteins as described previously [1].

Enrichment analysis of differentially expressed down- and up-regulated proteins in children and adults with ASD employed several databases including String, Wiki, Reactome, KEGG, Monarch, GO and Compartments (Tables 3, 4, 5 and 6, S5-S8). Lastly, SynGo and Simon’s Foundation websites (https://syngoportal.org/ and https://gene.sfari.org/ respectively) were consulted for the identification of synaptic proteins and risk genes for ASD (Table 2).

**Table 2:** Selected cerebellar vermis differentially expressed genes/proteins (FDR-adjusted *P* < 0.05) in children and adults with idiopathic autism

Class			Protein	Children	Adults	PS, Syn, PSD^1^	ASD RG^2^
I.	Synaptic Function						
	A.	Dendritic formation	DYSPL3	↑	—	+	+
		Dendritic spine formation	Septin 11	—	↓	+	—
			ITSN1	—	↓	+	+
		Mature dendritic spine formation	Septin 11	—	↓	+	—
	B.	Dendritic elimination, microglia	C3	↑	—	—	—
	C.	Dendritic maturation	PTPRZ1	↓	—	+	—
	D.	Dendritic arborization	Septin 6	↓	—	+	—
			Septin 11	—	↓	+	—
	E.	Dendritic stabilization	Septin 11	—	↓	+	—
	F.	Spine and cytoskeleton dynamics	DOCK1	↓	—	+	+
			GSN	↑	—	—	—
			PLEC	↑	—	—	—
			RDX	↑	—	+	—
			VCL	↑	↑	—	—
			VCAN	—	↑	+	—
			CAPZA2	↓	—	—	—
			SPTBN1	—	↓	+	+
			SPTBN2	↓	—	+	—
			SPTAN1	↓	—	+	—
			EZR	↑	—	+	—
			AHNAK	↑	—	—	+
II.	Synaptic Neurotransmission		SYT1	↓	—	+	+
			STXBP1	—	↓	+	+
			SYN1	—	↓	+	+
			SYN2	—	↓	+	+
			Septin 4	—	↓	+	—
III.	Synaptic Signaling						
	A.	Rho G signaling	ITSN1	—	↓	+	+
			ARHGAP26	↓	—	—	—
			DOCK1	↓	—	+	+
		Purkinje cell	ITPR1	↓	—	+	+
			NCKAP1	↓	—	+	+
			DYNLRB1	↓	—	—	—
			GDI-1	↑	—	+	—
IV.	Synaptic Protein Synthesis						
	A.	Gene transcription	HNRNPK	↓	—	+	+
			HNRNPD	↓	—	+	+
			EEF1A2	↓	↓	+	+
			EiF3L	↓	—	+	—
			SFPQ	↓	—	+	—
			FARSB	↓	↓	—	—
	B.	mRNA processing	AFG3L2	—	↓	—	—
			PABPC1	↓	—	+	+
			RPL0	↓	—	+	—
			RPLP2	↓	—	+	—
	C.	Protein folding and macroautophagy	CCT1 (TCP1)	↓	—	—	—
			CCT2	↓	—	—	—
			CCT3	↓	—	—	—
			CCT4	↓	—	—	+
			CCT5	↓	—	—	—
			CCT6A	↓	—	—	—
			CCT7	↓	—	—	—
			CCT8	↓	—	—	—
	D.	Chaperonin	HSPA1B	↑	—	—	—
			HSPD1	↑	—	—	—
			HSP90AA1	—	↓	+	—
V.	Protein Degradation						
	A.	Proteasome	PSMD13	↓	—	—	—
	B.	Ubiquitin proteasome	KRT9	—	↑	—	—
	C.	ER proteasomal sensing	ECPAS	↓	—	—	+
	D.	MVB synaptic proteostasis	SGIP1	—	↓	+	—
	E.	Autophagy/Aggrephagy	CCT1	↓	—	—	—
			CCT2	↓	—	—	—
			DYNC1H1	↓	↓	—	+
			DYNC1I1	↓	—	—	—
			DYNC1LI1	↓	—	—	—
			DYNC1LI2	↓	—	—	—
			PIK3C3	↓	—	+	—
			MAP1B	—	↓	+	+
			MACF1	—	↓	—	+
			CTSD	—	↑	+	—
		Mitophagy	PHB2	↑	—	+	—
	F.	Proteolysis	CTSD	—	↑	+	—
			Septin 3	↓	↓	+	—
			Septin 4	—	↓	+	—
			Septin 6	↓	↓	+	—
			Septin 9	—	↓	+	—
	G.	Phagosome	Septin 11	—	↓	+	—
			PHB2	↑	—	+	—
	H.	Protein aggregation	Septin 4	—	↓	+	—
			Septin 6	↓	↓	+	—
			HNRNPK	↓	—	+	+
			HNRNPD	↓	—	+	+
VI.	Cellular Stress						
	A.	Oxidative stress	GLO1	↑	—	—	+
			ECPAS	↓	—	—	+
			ALDH2	↑	—	—	—
	B.	Cell redox regulation	PRDX1	↑	—	—	—
			PRDX3	—	↑	—	—
VII.	Synaptic Trafficking						
	A.	Endocytosis	ITSN1	—	↓	+	+
			AP2A1	↓	—	+	—
			AP2B1	↓	—	+	—
			DNM1	—	↓	+	+
			DNM3	—	↓	+	—
	B.	ER-Golgi	Serpina1	↑	—	—	—
			Serpinb3	—	↑	—	—
			CAPZA2	↓	—	—	—
	C.	Golgi complex	SYNE1	↓	↓	+	+
			AP3B2	—	↓	+	—
			AP2A1	↓	—	+	—
			GDI-1	↑	—	+	—
	D.	Synaptic vesicle development and maintenance	DNM1	—	↓	+	+
			DNM3	—	↓	+	—
	E.	Post-Golgi vesicle-mediated transport-Anterograde axonal transport	AP3B2	↓	—	+	—
		-Golgi-ER retrograde transport	SYNE1	↓	↓	+	+
			DYNC1H1	↓	↓	—	+
			DYNC1I1	↓	—	—	—
			DYNC1LI1	↓	—	—	—
			DYNC1LI2	↓	—	—	—
		-Anterograde transport	DYNC1H1	↓	↓	—	+
		-Retrograde lysosomal transport	Septin 9	—	↓	+	—
		-Endosome-Golgi-ER retrograde transport	DYNLRB1	↓	—	—	—
		-stalled synaptic endocytosis	DNM1	—	↓	+	+
	F.	Dendritic transport of messenger ribonucleoprotein complex and kinase-associated granules	SFPQ	↓	—	+	—
	G.	Synaptic docking and fusion	STXBP1	—	↓	+	+
			NSF	—	↓	+	—
			Septin5A	—	↓	+	—
	H.	Synaptic vesicle function	SYNE1	↓	↓	+	+
			NSF	—	↓	+	—
			AP3B2	—	↓	+	—
	I.	Synaptic vesicle fission	DNM1	—	↓	+	+
			DNM3	—	↓	+	—
	J.	Exocytosis	ITSN1	—	↓	+	+
			SPTAN1	↓	—	+	—
			Septin 3	↓	↓	+	—
			Septin 6	↓	↓	+	—
			Septin 11	—	↓	+	—
VIII.	Neurodegeneration		Septin 4	—	↓	+	—
			TUBB2A	—	↓	—	—
			DYNC1H1	↓	↓	—	+
			FARSB	↓	↓	—	—
			SYNE1	↓	↓	+	+
			FAT2	—	↓	—	—
			AFG3L2	—	↓	—	—
		Purkinje cells	GRID2	—	↓	+	+
			HK1	—	↓	—	—
			MAP1B	—	↓	+	+
IX.	Myelination, oligodendrocytes		MBP	—	↑	—	—
			MDH2	—	↓	—	—
X.	Miscellaneous						
		Mature astrocytes	GFAP	↑	—	—	+
		Glycolysis, gluconeogenesis	TPI1	↑	—	—	—
		RNA polymerase II transport protein	RBMX	↓	—	+	—
		Postsynaptic signaling	MPP1	—	↓	+	—
			MPP6	—	↓	—	—
		Actin, RNA binding	AHNAK	↑	—	—	+
		Axon pathfinding, Speech	NOVA1	↓	—	—	—
		Synaptic transmission	GRID2	—	↓	+	+
		Mitochondrial dysfunction	AFG3L2	—	↓	—	—
		Glutamate synapse	ITSN1	—	↓	+	+

^1^presynaptic, synaptic, postsynaptic, synaptic membrane-associated proteins identified in SYNGO database

^2^risk genes/proteins for autism identified in SFARI database

↑ = significantly upregulated genes/proteins (FDR-adjusted *P* < 0.05) identified by proteomic analysis; ↓=significantly downregulated genes/proteins (FDR-adjusted *P* < 0.05) identified by proteomic analysis; + = present; — = absent; — = no change; PS  =  presynapstic; PSD = postsynaptic density; RG = risk gene

**Table 3:** Selected cerebellar vermis-enriched (FDR-adjusted *P* < 0.05) pathways in downregulated proteins (FDR-adjusted *P* < 0.05) of children with idiopathic autism

Pathways		Strength Score	Database
1.	Folding of actin by CCT/TriC	2.42	Reactome
2.	Formation of tubulin folding intermediates by CCT/TriC	2.02	
3.	Prefoldin-mediated transfer of substrate to CCT/TriC	1.99	
4.	BBSome-mediated cargo targeting to cilium	1.95	
5.	Cooperation of PDCL (PhLP1) and TriC/CCT in G-protein beta folding	1.84	
6.	Association of TriC/CCT with target proteins during biosynthesis	1.83	
7.	RHOBTB2 GTPase cycle	1.76	
8.	Retrograde neurotrophin signaling	1.67	
9.	VLDLR internalization and degradation	1.61	
10.	Trafficking of GLUR2-containing AMPA receptors	1.59	
11.	Aggrephagy	1.50	
12.	COPI-independent Golgi-to-ER retrograde traffic	1.50	
13.	HSP90 chaperone cycle for steroid hormone receptors in the presence of ligand	1.47	
14.	COPI-mediated anterograde transport	1.42	
15.	Macroautophagy	1.09	
16.	L13a-mediated translational silencing of ceruloplasmin expression	1.08	
17	Cap-dependent translation initiation	1.05	
18.	Rho GTPases activate formins	0.98	
19.	Translation	0.83	
20.	Rho GTPase effectors	0.83	
21.	Rho GTPase cycle	0.82	
22.	Membrane trafficking	0.76	
23.	Axon guidance	0.78	
24.	Cellular response to stress	0.60	
1.	Chaperonin-containing T-complex	2.42	GO Component
2.	DNA-dependent protein kinase complex	2.22	
3.	AP-2 adaptor complex	1.97	
4.	Paraspeckles	1.91	
5.	tRNA-splicing ligase complex	1.91	
6.	Spectrin-associated cytoskeleton	1.86	
7.	Cytoplasmic dynein complex	1.85	
8.	Septin ring	1.67	
9.	Septin complex	1.67	
10.	Clathrin-coated endocytic vesicle membrane	1.15	
11.	Microtubule	0.64	
12.	Synapse	0.47	
1.	ATP-dependent protein folding chaperone	1.93	GO Function
2.	Dynein heavy chain binding	1.85	
3.	Unfolded protein binding	1.36	
4.	mRNA 3-UTR binding	1.11	
1.	Chaperone-mediated protein folding	1.57	GO Process
2.	Regulation of telomerase activity	1.51	
3.	Regulation of mRNA splicing via spliceosome	1.17	
4.	Regulation of protein stability	0.95	
5.	RNA splicing	0.85	
1.	Chaperonin TCP-1 conserved site	2.52	STRING
2.	Chaperonin TRP-1, conserved site and cellular proteostasis	2.31	
3.	NOPS, and amyotrophic lateral sclerosis type 21	2.12	
4.	tRNA-splicing ligase complex	2.12	
5.	Nonhomologous end joining complex	2.12	
6.	Cytoplasmic dynein complex	1.96	
7.	Cytoplasmic dynein complex, and dynactin complex	1.74	
8.	Mixed inclusion multisystem proteinopathy, and zinc finger CHHC-type	1.79	
1.	Dynein 1 light intermediate chain	2.52	InterPro
2.	Chaperonin TCP-1 conserved site	2.47	
3.	Spectrin repeat	1.65	
4.	RNA recognition motif domain, eukaryote	1.63	
5.	Armadillo-type fold	0.94	
1.	Actin capping	1.71	UniProt
2.	Dynein	1.70	
3.	Chaperone	1.12	
4.	Motor protein	1.09	
5.	Methylation	0.70	
6.	Acetylation	0.59	
1.	AP-2 adaptor complex	1.97	Compartments
2.	DNA ligase IV complex	1.97	
3.	tRNA-splicing ligase complex	1.97	
4.	Paraspackles	1.91	
5.	Spectrin	1.91	
6.	Septin ring	1.67	
7.	Septin complex	1.67	
8.	Cytoplasmic stress granule	1.19	
9.	Cytoplasmic ribonucleoprotein granule	1.04	
10.	Microtubule	1.10	
11.	Endocytic vesicle membrane	0.99	

**Table 4:** Selected cerebellar vermis-enriched (FDR-adjusted *P* < 0.05) pathways in upregulated proteins (FDR-adjusted *P* < 0.05) of children with idiopathic autism

Pathways		Strength Score	Database
1.	Phosphopyruvate hydratase complex	2.45	Compartment
2.	Costamere	2.21	
3.	Podosome	1.75	
4.	Cortical actin cytoskeleton	1.59	
5.	Cortical cytoskeleton	1.55	
6.	Ficolin-1-rich granule lumen	1.36	
7.	Extracellular exosome	1.26	
8.	Secretory granule lumen	1.07	
1.	Glyceraldehyde-3-phosphate dehydrogenase (NAD+) nonphosphorylating activity	2.30	GO Function
2.	Aldehyde dehydrogenase (NAD+) activity	2.12	
3.	NAD binding	1.58	
4.	Cadherin binding	1.23	
5.	Cell adhesion molecule binding	1.05	
6.	Enzyme binding	0.61	
1.	Ezrin/radixin/moesin, alpha helical domain	2.55	InterPro
2.	Moesin tail domain superfamily	2.45	
3.	Aldehyde dehydrogenase, glutamic acid active site	2.12	
4.	Aldehyde/histidinal dehydrogenase	2.02	
1.	Beta alanine metabolism	1.86	KEGG
2.	Pyruvate metabolism	1.77	
3.	Tryptophan metabolism	1.71	
4.	Fatty acid degradation	1.70	
5.	Valine, leucine, and isoleucine degradation	1.66	
6.	Glycolysis/gluconeogenesis	1.64	
7.	Biosynthesis of amino acids	1.46	
8.	Carbon metabolism	1.38	
9.	Regulation of actin cytoskeleton	1.13	
1.	Actin capping	1.87	UniProt
2.	Lyase	1.25	
3.	NAD	1.18	
4.	Actin-binding	1.02	
5.	Methylation	0.70	
6.	Acetylation	0.64	
7.	Cytoplasm	0.34	
8.	Phosphoprotein	0.30	
1.	Finnish type amyloidosis	2.37	Diseases
2.	Primary cutaneous amyloidosis	2.05	
1.	Regulation of actin cytoskeleton	1.28	Wiki Pathways

**Table 5:** Selected cerebellar vermis-enriched (FDR-adjusted *P* < 0.05) pathways in downregulated proteins (FDR-adjusted *P* < 0.05) of adults with idiopathic autism

Pathways		Strength Score	Database
1.	Golgi to plasma membrane protein transport	1.69	GO Process
2.	Synaptic vesicle localization	1.69	
3.	Synaptic vesicle endocytosis	1.55	
4.	Activation of innate immune response	1.48	
5.	Synaptic vesicle cycle	1.44	
6.	Cytoskeleton-dependent cytokinesis	1.42	
7.	Vesicle-mediated transport	0.66	
8.	Neurogenesis	0.62	
1.	GTP binding	1.19	GO Function
2.	GTPase activity	1.15	
1.	Ku70:Ku80 complex	2.69	GO Component
2.	DNA-dependent protein kinase complex	2.39	
3.	Septin ring	2.25	
4.	Septin complex	2.25	
5.	Dendritic spine	1.05	
6.	Postsynaptic density	1.03	
7.	Presynapse	0.97	
8.	Axon	0.96	
9.	Glutamatergic synapse	0.95	
10.	Postsynapse	0.94	
11.	Microtubule	0.94	
12.	Synaptic vesicle	0.97	
13.	Synaptic membrane	0.90	
14.	Synapse	0.82	
1.	Septin ring	2.39	STRiNG
2.	Spectrin and ankryn UPA domain	2.22	
3.	Microtubule-dependent trafficking	2.22	
4.	Post-chaperonin tubulin folding pathway, and calponin repeat	1.58	
5.	Clathrin coat and presynaptic endocytosis	1.53	
6.	Mixed, including post-chaperonin tubulin folding pathway and microtubule end	1.42	
7.	Mixed, including Golgi-to-ER retrograde transport and cytoplasmic dynein complex	1.20	
1.	Synaptic vesicle cycle	1.44	KEGG
1.	Constituitive signaling by overexpressed ERBB1	1.95	REACTOME
2.	Serotonin neurotransmitter release cycle	1.91	
3.	Retrograde neurotrophin signaling	1.85	
4.	Dopamine neurotransmitter cycle	1.81	
5.	Microtubule-dependent trafficking of connexons from Golgi to the plasma membrane	1.71	
6.	Signaling by FLT3 fusion proteins	1.71	
7.	Aggrephagy	1.67	
8.	Post-chaperonin tubulin folding pathway	1.65	
9.	Formation of tubulin folding intermediates by CCT/TriC	1.60	
10.	HSP90 chaperone cycle for steroid hormone	1.55	
11.	COPI-mediated anterograde transport	1.47	
12.	COPI-independent Golgi-to-ER retrograde traffic	1.45	
13.	Post NMDA receptor activation events	1.23	
14.	Golgi-to-ER retrograde traffic	1.17	
15.	Clathrin-mediated endocytosis	1.13	
16.	Transmission across chemical synapses	1.11	
17.	Neurotransmitter receptors and postsynaptic signal transmission	0.98	
18.	Vesicle-mediated transport	0.91	
19.	Axon guidance	0.91	
1.	Ku70:Ku80 complex	2.69	Compartments
2.	Septin ring	2.25	
3.	Septin complex	2.25	
4.	DNA ligase IV complex	2.15	
5.	DNA-dependent protein kinase complex	2.04	
6.	Spectrin	1.91	
7.	Nuclear telomere cap complex	1.88	
8.	Non-homologous end-joining complex	1.79	
9.	Axon	1.11	
10.	Presynapse	1.11	
11.	Microtubule	1.10	
12.	Actin cytoskeleton	0.91	
13.	Synapse	0.90	
1.	Systemic lupus erythematosus	1.67	UniProt
2.	Endocytosis	1.28	
3.	Epilepsy	1.14	
4.	Neurodegeneration	0.99	
5.	Synapse	0.93	
1.	Cerebellar ataxia	1.26	Diseases
2.	Epilepsy	1.05	
3.	Neurodegenerative disease	0.86	
1.	Abnormal hippocampus morphology	1.52	Monarch
2.	Lateral ventricle dilation	1.40	
3.	Epileptic encephalopathy	1.39	
4.	Autism	1.17	
5.	Cerebellar atrophy	1.06	
6.	Autistic behavior	0.93	
7.	Abnormal cortical gyration	0.95	
8.	Delayed speech and language development	0.79	

**Table 6:** Selected cerebellar vermis-enriched (FDR-adjusted *P* < 0.05) pathways in upregulated proteins (FDR-adjusted *P* < 0.05) of adults with idiopathic autism

Pathways		Strength Score	Database
1.	Positive regulation of plasminogen activation	2.23	GO Process
2.	Intermediate filament organization	2.11	
3.	Peptide cross-linking	1.96	
4.	Keratinocyte differentiation	1.81	
5.	Regulation of endopeptidase activity	0.99	
6.	Regulation of proteolysis	0.81	
1.	Zonula adherens	2.18	GO Component
2.	Desmosome	2.04	
3.	Compact myelin	2.02	
4.	Intermediate filament	1.69	
5.	Ficolin-1-rich granule lumen	1.44	
6.	Ficolin-1-rich granule	1.41	
7.	Secretory granule lumen	1.23	
8.	Secretory granule	0.93	
1.	Estrogen signaling pathway	1.49	KEGG
2.	Biosynthesis of amino acids	1.45	
1.	Neutrophil degranulation	1.15	Reactome
2.	Developmental biology	1.04	
1.	Primary cutaneous amyloidosis	2.03	Diseases
2.	Amyloidosis	1.56	
1.	Macroglia	1.54	Tissues
2.	Interstitial cell of Cajal	1.15	
1.	Fascia adherens	2.53	Compartments
2.	Keratin filament	2.37	
3.	Zonula adherens	2.23	
4.	Desmosome	2.07	
5.	Intermediate filament	2.04	
6.	Peptidase inhibitor complex	1.90	
7.	Specific granule lumen	1.64	
8.	Ficolin-1-rich granule lumen	1.44	
9.	Vacular lumen	1.31	
10.	Secretory granule lumen	1.30	
11.	Secretory granule	1.02	
1.	Intermediate filament	2.07	UniProt
2.	Keratin	1.79	
3.	Citrullination	1.49	
4.	Methylation	0.69	
1.	Keratin, type II	2.32	InterPro
2.	Keratin, type II head	2.28	
3.	Keratin, type I	2.03	

### SDS-PAGE and Immunoblotting

Protein samples (in PBS) were mixed at a 1:1 ratio with non-reducing 2X Novex Tris-Glycine SDS Sample Buffer. 10 µg of total protein was loaded per lane on Novex Tris-Glycine Mini Protein Gels, 4–20%, 1.0 mm, 15 well gels. The gels were run at 225 V for 40 min and transferred onto nitrocellulose membranes at 40 V overnight at 4 °C. The membranes were blocked in 5% non-fat milk (NFM) in 0.1% tween 20/PBS (PBST) at room temperature for 1 h. The membranes were incubated in primary antibody diluted in 5% NFM/PBST overnight at 4 °C, then washed 3**x** with PBST for 5 min each. The membranes were incubated with secondary antibodies diluted in 5% NFM/PBST at room temperature for 1 h, washed 3**x** with PBST and 1**x** with PBS for 5 min each, and then scanned with a LI-COR Odyssey CLx machine. The membranes were initially blotted with rabbit anti-GFAP antibody Z0334, then stripped in 0.2 M NaOH (two 5-min incubations) and re-probed with mouse-anti-GFAP SMI 26 and rabbit anti-pan-actin antibodies. Parallel equally loaded gels were used for Coomassie staining of total protein.

### Antibodies

The following antibodies were used for immunoblots: purified mouse monoclonal anti-GFAP (SMI 26) from BioLegend (1:2000); polyclonal rabbit anti-GFAP (Z0334) from Agilent/DAKO (1:5000); rabbit anti-pan-actin from Cell Signaling Technology (1:2000). The following secondary antibodies and concentrations were utilized: IRDye 800CW goat anti-rabbit IgG (LI-COR, WB: 1:5000), IRDye 680RD donkey anti-mouse IgG (LI-COR, WB 1:5000).

### Data Analysis

Image Studio version 5.2 (LI-COR) was used to perform densitometry on immunoblots. Adobe Photoshop 26.4.1 version was used to quantify the lane intensity (37-250 kDa range) of Coomassie-stained gels. Bar graphs were generated with the GraphPad Prism 10 software and statistical analysis was performed via unpaired t-test.

## Results

Analysis of significantly downregulated proteins in children (FDR-adjusted *p* < 0.05, Tables 2, S1, S5) exhibited significant enrichment of pathways involved in protein folding, Rho GTPase cycle, trafficking of alpha-amino-3-hydroxy-5-methyl-4-isoxazolepropionic acid (AMPA) receptors, aggrephagy, macroautophagy, anterograde and retrograde endoplasmic reticulum (ER)-Golgi and Golgi-ER transport, membrane trafficking, DNA-dependent protein kinase activity, unfolded protein binding, tRNA splicing ligase complex, proteinopathy, mRNA splicing and DNA repair (FDR-adjusted *p* < 0.05, Tables 3, S1). In contrast, upregulated proteins in children with ASD showed enrichment of pathways involved in metabolic pathways related to biosynthesis of amino acids, degradation of valine, leucine, isoleucine, pyruvate metabolism, glycolysis, gluconeogenesis, carbon metabolism, actin capping, methylation, acetylation, amyloidosis, nicotinamide adenine dinucleotide (NAD) activity and binding and regulation of actin cytoskeleton (Table 4, Table S2).

Analysis of significantly downregulated proteins in adults (FDR-adjusted *p* < 0.05, Tables 5, S7) exhibited significant enrichment of pathways involved in endocytosis, exocytosis, protein transport, presynaptic, synaptic, and postsynaptic activities, Golgi to ER and Golgi to plasma membrane trafficking, aggrephagy, DNA repair, protein folding, serotonin and dopamine neurotransmitter release, clathrin mediated endocytosis, axon guidance, glutamate and post-NMDA receptor activation, and disease process involving neurodegeneration, epilepsy, cerebellar ataxias, autism, and systemic lupus erythematosus (FDR-adjusted *p* < 0.05, Tables 2 and 5). Review of significant enrichment of upregulated pathways in cerebellum of ASD adults (FDR-adjusted *p* < 0.05) revealed involvement of gene sets in peptide cross-linking, regulation of proteolysis, amyloidosis, citrullination, methylation, endopeptidase and plasminogen activity, and biosynthesis of amino acids (Table 6, S4, S8, Table 2).

Proteomic analysis of cerebellar synaptic fractions obtained from different groups (autistic vs. controls; children vs. adults) demonstrated a list of several thousand peptides per group. Quantitation of identified proteins and enumeration of differentially expressed proteins were based on peptide quantitation approach employed previously [1]. The peptide quantification approach was used to generate FDR-adjusted *p* values for significantly altered, differentially expressed proteins (Table 2, S1-S4). Comparison of results based on this method helped us to obtain statistically reliable tests to ascertain autistic pathologically-derived proteomic results when compared to control values.

For cerebellar vermis of autistic children, using the peptide-based LMM method, 8689 peptides were detected and 2689 proteins were identified based on the presence of at least two unique peptides or more per protein. This technique yielded 88 proteins significant at the 5% level (28 upregulated and 60 downregulated after FDR adjustment; Tables S1-S2).

Proteomic data obtained from cerebellar vermis of adult subjects diagnosed with ASD using peptide analytic technique yielded 9094 peptides and 2580 proteins with 71 proteins significant at the 5% level (FDR-adjusted *p* < 0.05; 30 upregulated and 41 downregulated; Tables S3-S4).

**Fig. 1:**
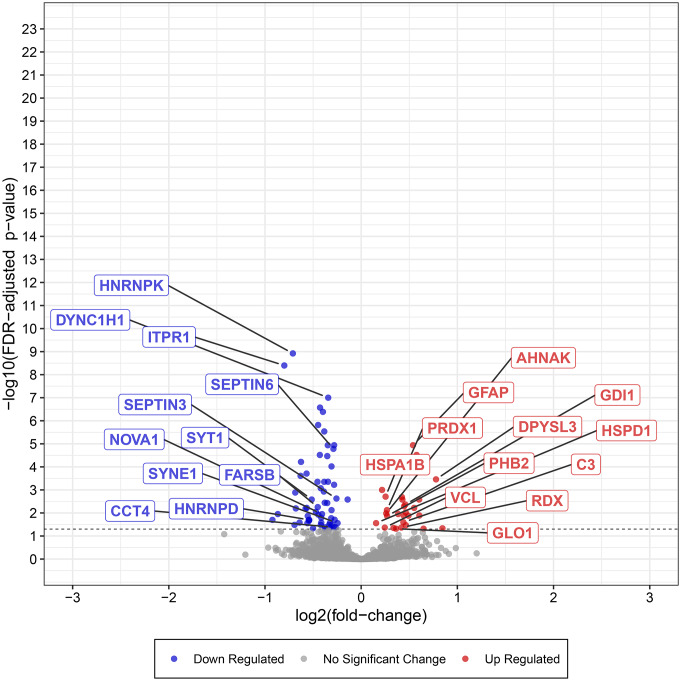
Expression of selected synaptic and associated proteins significantly downregulated or upregulated (FDR-adjusted *p* < 0.05) in cerebellar vermis of children with idiopathic ASD. Data shown are plots of -log_10_ (adjusted *p*-value) vs. log_2_ (fold change) for all identified proteins. Synaptic and associated proteins are labeled when above the threshold of significance

**Fig. 2:**
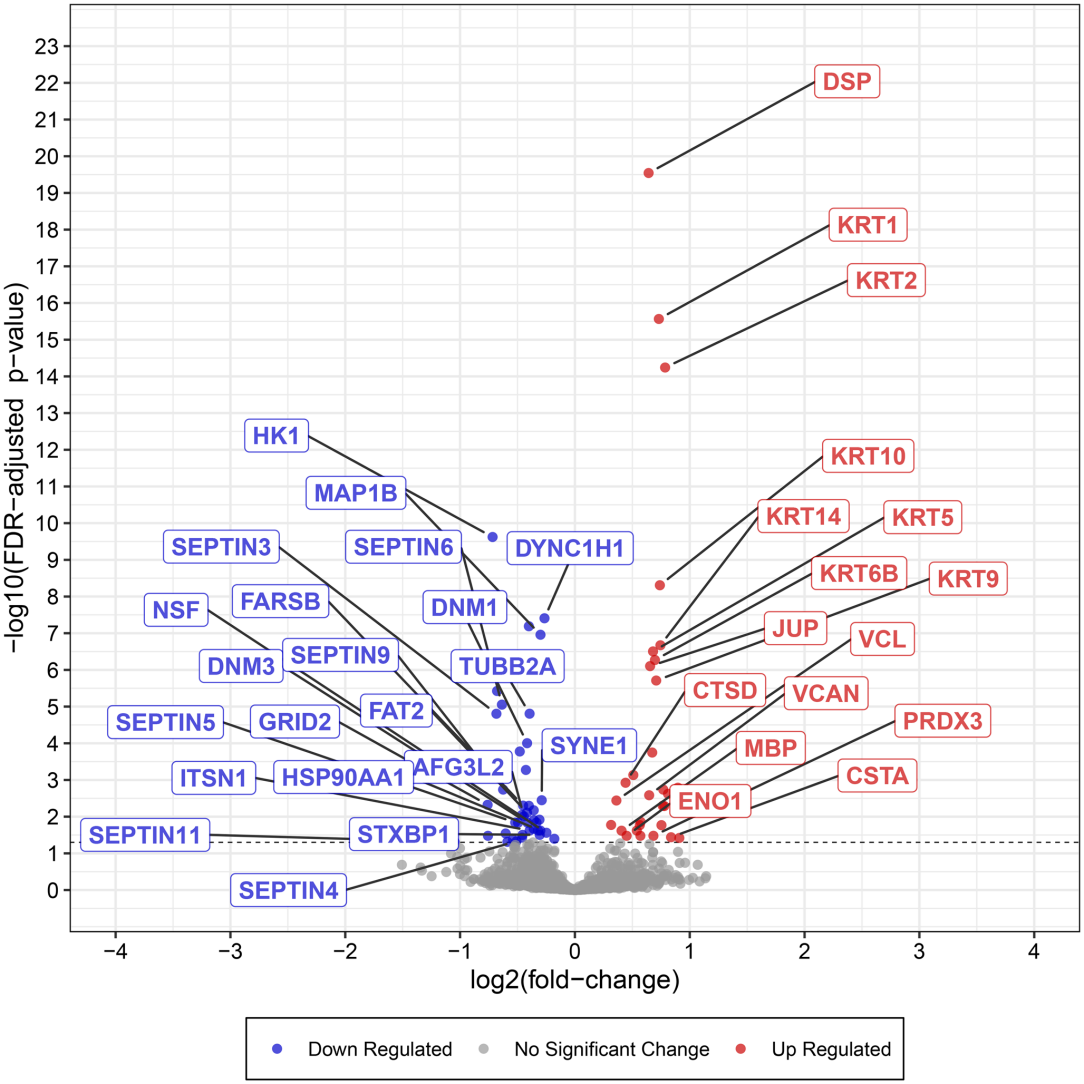
Expression of selected synaptic and associated proteins significantly downregulated or upregulated (FDR-adjusted *p* < 0.05) in cerebellar vermis of adults with idiopathic ASD. Data shown are plots of -log_10_ (adjusted *p*-value) vs. log_2_ (fold change) for all identified proteins. Synaptic and associated proteins are labeled when above the threshold of significance

**Fig. 3:**
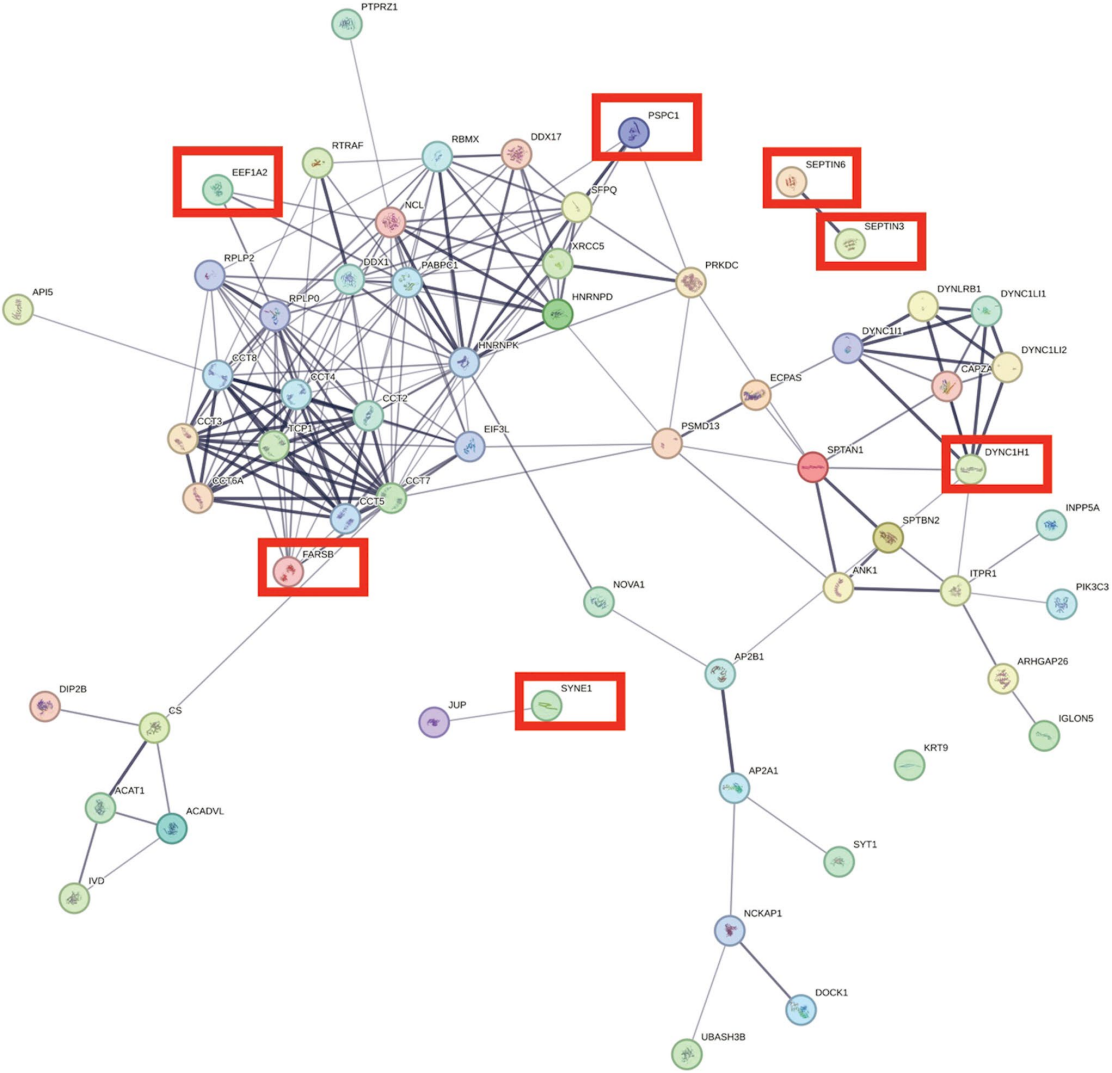
STRING protein-protein interaction network generated from statistically significant downregulated cerebellar vermis proteins from children with idiopathic autism. Network nodes represent proteins. Edges represent protein-protein associations (i.e., jointly contribute to a shared function). Edge confidence is measured by the intensity of the edge in the network. Red-squared nodes are also downregulated proteins in adults with idiopathic autism

**Fig. 4:**
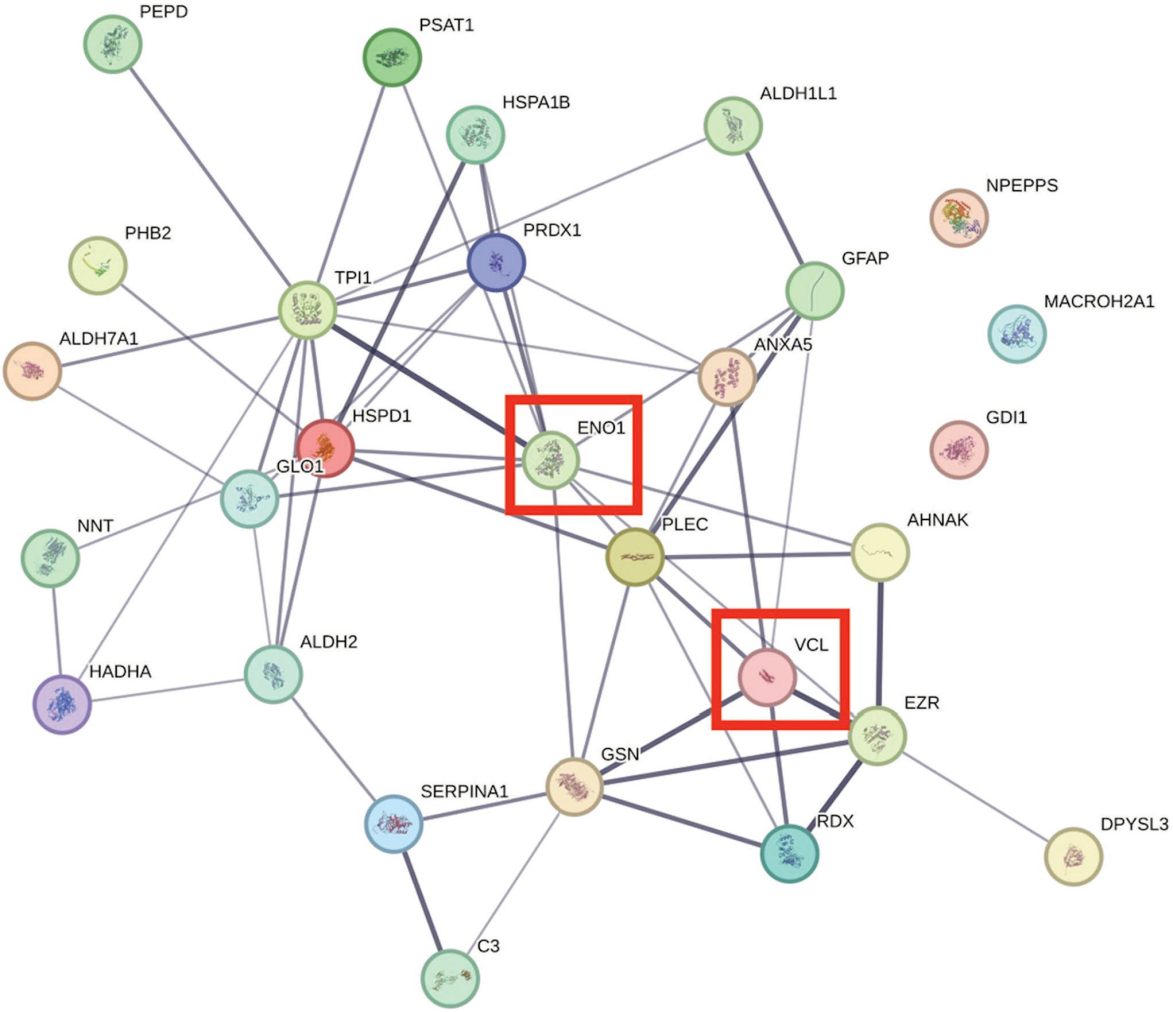
STRING protein-protein interaction network generated from statistically significant upregulated cerebellar vermis proteins from children with idiopathic autism. Network nodes represent proteins. Edges represent protein-protein associations (i.e., jointly contribute to a shared function). Edge confidence is measured by the intensity of the edge in the network. Red-squared nodes are also upregulated proteins in adults with idiopathic autism

**Fig. 5:**
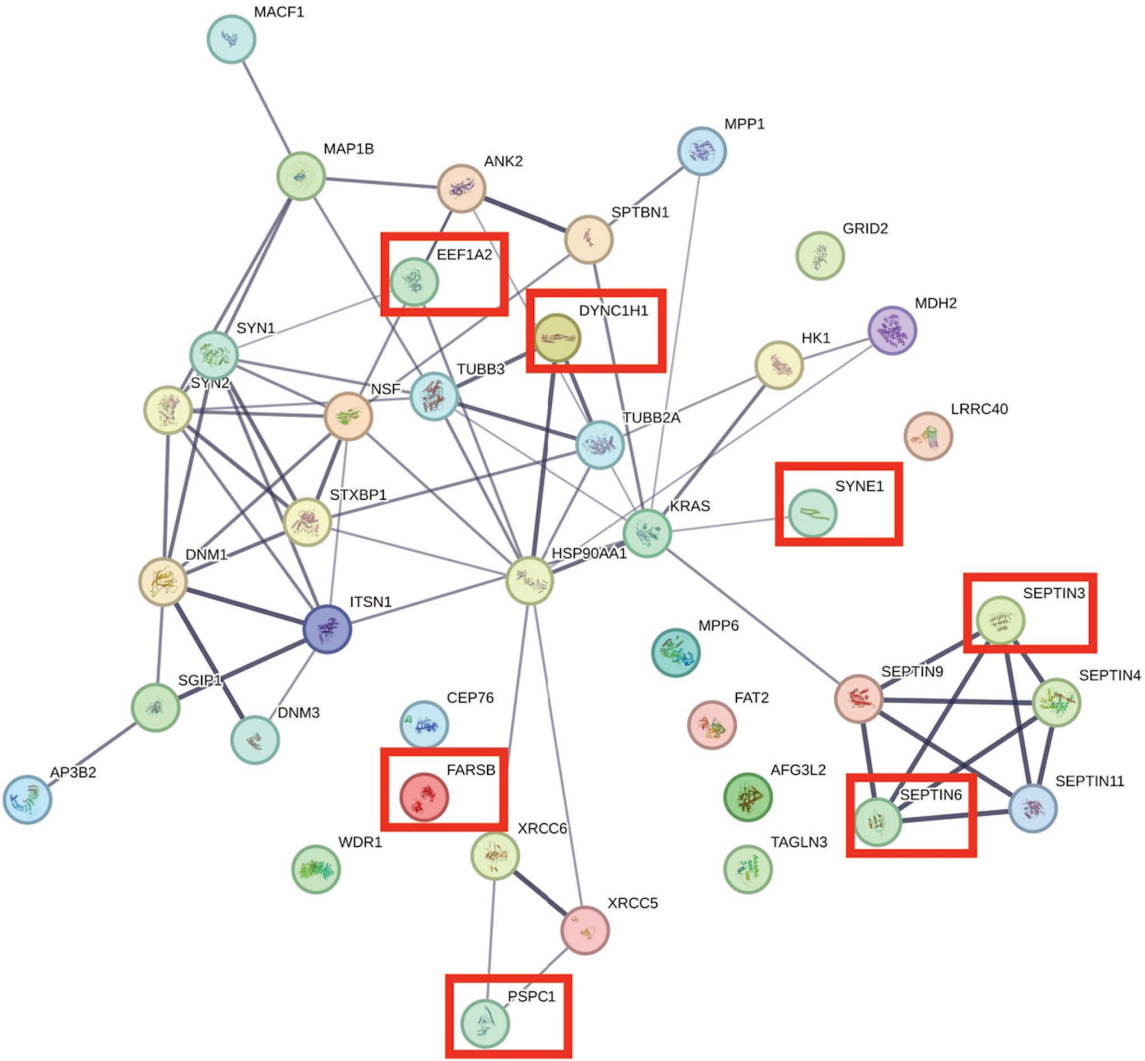
STRING protein-protein interaction network generated from statistically significant downregulated cerebellar vermis proteins from adults with idiopathic autism. Network nodes represent proteins. Edges represent protein-protein associations (i.e., jointly contribute to a shared function). Edge confidence is measured by the intensity of the edge in the network. Red-squared nodes are also downregulated proteins in children with idiopathic autism

**Fig. 6:**
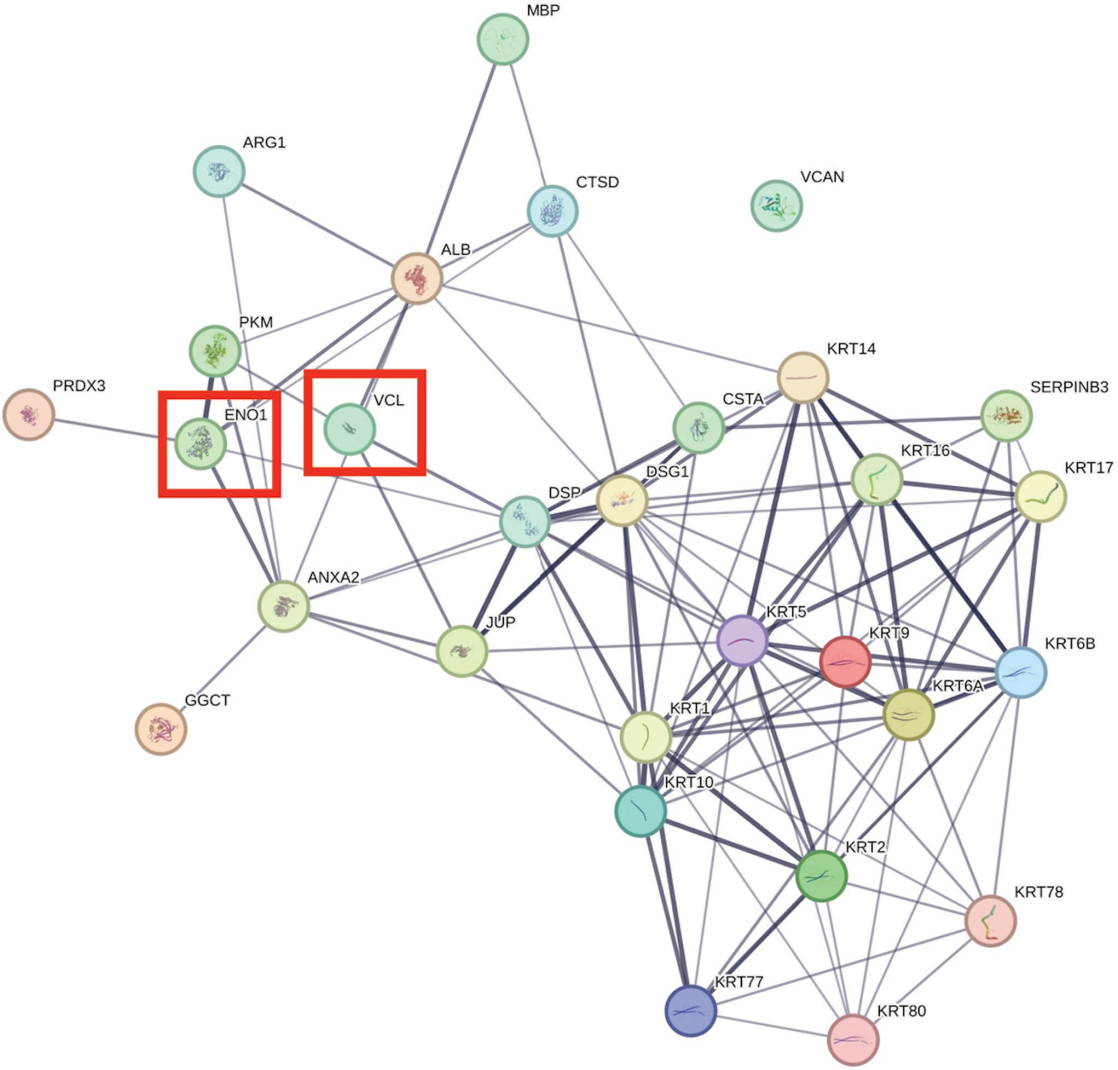
STRING protein-protein interaction network generated from statistically significant upregulated cerebellar vermis proteins from adults with idiopathic autism. Network nodes represent proteins. Edges represent protein-protein associations (i.e., jointly contribute to a shared function). Edge confidence is measured by the intensity of the edge in the network. Red-squared nodes are also upregulated proteins in children with idiopathic autism

Comparison of several differentially expressed proteins (FDR-adj. *p* < 0.05) between children and adults with ASD showed significant downregulation in seven proteins in both children and adults with ASD (Figs. 1, 2, 3, 4, 5 and 6, Tables S1-S4, Table 2) including markers for retrograde transport (DYNC1H1), protein translation, elongation, and regulation of chaperonin-mediated autophagy (EEF1A2), aminoacylation of cognate amino acids (phenylalanine aminoacyl-tRNA synthesis FARSB), DNA damage (PSPC1), synaptic autophagy (septin 3), axonal outgrowth and dendritic branching (septin 6), and regulation of postsynaptic neurotransmitter receptor endocytosis (SYNE1). In contrast, two proteins were overexpressed in children and adults including ENO1 and VCL. Lastly, levels of JUP and KRT9 were decreased in children but increased in adults with ASD (Figs. 1 and 2; Tables 2, S1-S4).

### Children with ASD

Many cerebellar developmental proteins involved in dendritogenesis, synaptic structure, and PC circuitry were significantly altered in vermis of children with autism (Figs. 1 and 3; Table 2 and S1-S2). DPYSL3 protein, which is involved in dendritic spine formation and synaptic plasticity [19], was significantly upregulated in autistic vermis (DPYSL3: log_2_ FC = + 0.7780, FDR adj. *P* = 0.0003; Fig. 1, Table S2). Receptor-type tyrosine-protein phosphatase zeta (PTPRZ1) participates in cerebellar development [20, 21] and is involved in cell communication between granule cell precursors, Bergman glia [22], and PCs via its target, protein pleiotrophin (PTN) [23], was reduced significantly in ASD children (log_2_ FC = -0.3529, FDR adj. *P* = 0.0037, Table S1). Interestingly, complement C3 [24], a protein involved in synaptic pruning of postsynaptic membranes [24], upregulated in PFC of ASD brains [1] was upregulated in vermis of autistic children (log_2_ FC = + 0.4385, FDR adj. *P* = 0.0253, Figs. 1 and 4, Table S2).

The protein levels for a number of proteins involved in vesicular trafficking and secretory pathways were altered significantly in cerebellar vermis of children with autism (Tables 2, S1-S2). Endoplasmic reticulum (ER)-Golgi complex associated protein alpha 1 antitrypsin (SERPINA1) was upregulated significantly (log_2_ FC = + 0.4431, FDR adj. *P* = 0.0131, Table S2). Several proteins associated with intracellular trafficking process including cytoplasmic dynein heavy chain 1 (DYNC1H1), a risk gene for ASD (Fig. 1), cytoplasmic dynein 1 intermediate chain 1 (DYNC1I1), cytoplasmic dynein 1 light intermediate chain 1 (DYNC1LI1), and DYNC1LI2 were significantly reduced in vermis of ASD children (DYNC1H1: log_2_ FC = -0.3429, FDR adj. *P* = 0.0000; DYNC1I1: log_2_ FC = -0.9228, FDR adj. *P* = 0.0200; DYNC1LI1: log_2_ FC = -0.4543, FDR adj. *P* = 0.0004; DYNC1LI2: log_2_ FC = -0.3114, FDR adj. *P* = 0.0169). Additionally, dynein light chain roadblock type 1 (DYNLRB1) which interacts with Rab6 isoforms and colocalizes to the Golgi complex, participating in retrograde transport from endosome via Golgi complex to the ER, was significantly reduced in autistic cerebellum of children (log_2_ FC = -0.6920, FDR adj. *P* = 0.0329, Tables 2, S1). Levels for markers of exocytosis, septin 3 and 6 were reduced significantly in ASD vermal tissues (septin 3: log_2_ FC = -0.2572, FDR adj. *P* = 0.0024; septin 6: log_2_ FC = -0.2887, FDR adj. *P* = 0.0000, Fig. 1, Table S1).

An important protein involved in neurotransmitter release, synaptotagmin 1 (SYT1) was reduced in children with ASD (log_2_ FC = -0.5145, FDR adj. *P* = 0.0026, Fig. 1). Another protein, spectrin alpha chain, non-erythrocytic 1 (SPTAN1) which is associated with neurotransmitter release, was downregulated in ASD children (log_2_ FC = -0.1410, FDR adj. *P* = 0.0026). Level of a related protein with a role in synaptogenesis, spectrin beta chain non-erythrocytic 2 (SPTBN2), was also reduced significantly (log_2_ FC = -0.3789, FDR adj. *P* = 0.0036). A protein linked to and involved in mitophagy and mitochondrial autophagic degradation, prohibitin 2 (PHB2) was upregulated significantly in children with ASD (PHB2: log_2_ FC = + 0.2694, FDR adj. *P* = 0.0135).

Several Rho-GTPase signaling proteins involved in secretory pathways were abnormally regulated in ASD child cerebellar vermis such as Rho GEF protein, dedicator of cytokinesis protein 1 (DOCK1), RhoGAP protein ARHGAP26, Rho GTPase effector Nck-associated protein 1 (NCKAP1), inositol 1,4,5-triphosphate receptor type 1 (ITPR1), Rab/Rho GTPase regulator, Rab GDP-dissociation inhibitor alpha (GDI1), and Rab6 effector DYNLRB1. Levels of five of these proteins were reduced significantly including DYNLRB1 (see previous section), ARHGAP26 (log_2_ FC = -0.5554, FDR adj. *P* = 0.0135), DOCK1 (log_2_ FC = -0.4147, FDR adj. *P* = 0.0262), ITPR1 (log_2_ FC = -0.7994, FDR adj. *P* = 0.0000), and NCKAP1 (log_2_ FC = -0.3838, FDR adj. *P* = 0.0000). By contrast, level of an important Rab/Rho GTPase regulator GDI1 was increased significantly (log_2_ FC = + 0.4589, FDR adj. *P* = 0.0043, Fig. 1).

Several proteins which participate in Ca^++^ regulation in autistic cerebellum included SYT1, ITPR1, INPP5A, and AHNAK (Table 2). Levels of all except one (AHNAK) were reduced. The level was elevated for AHNAK, a regulator of voltage-gated, calcium channel activity (log_2_ FC = + 0.2605, FDR adj. *P* = 0.0104, Fig. 1). ITRP1 level was reduced (Table S1). Level of type 1 inositol 1,4,5-triphosphate 5 (INPP5A) was also reduced (log_2_ FC = -0.5419, FDR adj. *P* = 0.0217, Table S1). Level of SYT1 was also reduced as described previously (Fig. 1).

We next evaluated several proteins involved in proteolysis, protein folding, oxidative stress, mitochondrial functions (energy production and metabolism), and cytoskeletal function (actin and microtubulin-related transport and secretory pathway). There were significant alterations in levels of proteins involved in protein folding, (TCP1, CCT2, CCT3, CCT4, CCT5, CCT6A, CCT7, CCT8, all downregulated, FDR-adjusted *P* < 0.05, Tables 2, S1), cytoskeletal functions (PLEC, RDX, VCL, all upregulated, all FDR-adjusted *P* < 0.05), chaperonin activity (HSPA1B, HSPD1, upregulated; FDR-adjusted *P* < 0.05, Table S2), DNA damage repair (XRCC5, downregulated, FDR-adjusted *P* < 0.05 Table S1), proteolysis (ECPAS, PSMD13, downregulated; FDR-adjusted *P* < 0.05, Table S1), oxidative stress (GLO1, ALDH2, upregulated; FDR-adjusted *P* < 0.05, Table S2), mitochondrial stress response (DDX1, downregulated; FDR-adjusted *P* < 0.05, Table S1) mitochondrial cell redox homeostasis, (NNT upregulated; FDR-adjusted *P* < 0.05, Table S2), glycolysis (GLO1, TPI1, ENO1, all upregulated; FDR-adjusted *P* < 0.05, Table S2), fatty acid beta oxidation (HADHA, upregulated; FDR-adjusted *P* < 0.05, Table S2), carbohydrate metabolism (mitochondrial CS, downregulated; FDR-adjusted *P* < 0.05, Table S1), one carbon metabolism (ALDH1L1, upregulated; FDR-adjusted *P* < 0.05, Table S2), actin-related function (GSN, upregulated; NCKAP1, downregulated; FDR-adjusted *P* < 0.05, Tables S1-S2), microtubulin-related (DYNLRB1, downregulated; FDR-adjusted *P* < 0.05, Table S1), tight-junction-related (EZR, upregulated; FDR-adjusted *P* < 0.05, Table S2), splicing (RBMX, downregulated; FDR-adjusted *P* < 0.05, Table S1), and finally, metaloprotease-related (PEPD, upregulated; FDR-adjusted *P* < 0.05, Table S2). Lastly, an important protein related to long term potentiation (LTP), glutamate function, and cognition (PRKDC, downregulated; FDR-adjusted *P* < 0.05, Table S1) was reduced significantly in cerebellar vermis of ASD children (Table 2).

### Adults with ASD

Six members of the septin family of cytoskeletal proteins were downregulated in vermis of adults with ASD (Figs. 2 and 5; Tables 2 and 5, S3). These proteins consisted of septin 3 (log_2_ FC = -0.6837, FDR adj. *P* = 0.000), septin 5 (log_2_ FC = -0.5202, FDR adj. *P* = 0.0145), septin 4 (log_2_ FC = -0.5097, FDR adj. *P* = 0.0470), septin 6 (log_2_ FC = -0.6358, FDR adj. *P* = 0.0000), septin 9 (log_2_ FC = -0.6259, FDR adj. *P* = 0.0018), and septin 11 (log_2_ FC = -1.0475, FDR adj. *P* = 0.0486) (Fig. 5; Tables 2, S3). Two members of synaptic vesicle exocytosis protein (SYN1) and synaptic vesicle clustering and glutamate synaptic reserve pool vesicle protein (SYN2) were downregulated significantly (SYN1: log_2_ FC = -0.6779, FDR adj. *P* = 0.0000; SYN2: log_2_ FC = -0.4951, FDR adj. *P* = 0.0146, Table 2, S3). A further presynaptic protein involved in release of neurotransmitter via regulation of syntaxin, syntaxin-binding protein 1 (STXBP1) was decreased significantly in adults (log_2_ FC = -0.3065, FDR adj. *P* = 0.0313, Fig. 2; Tables 2 and 5, S3). Two members of synaptic development, maturation and maintenance-related proteins, dynamins 1 and 3 were reduced significantly (DNM1: log_2_ FC = -0.4164, FDR adj. *P* = 0.0001; DNM3: log_2_ FC = -0.4124, FDR adj. *P* = 0.0146, Fig. 2; Table 2, S3). Membrane palmitoylated proteins 1 and 6 (MPP1 and MPP6), involved in protein transport (MPP1: log_2_ FC = -0.7572, FDR adj. *P* = 0.0332) and synaptic receptor clustering, sleep cycle and myelination (MPP6: log_2_ FC = -0.3349, FDR adj. *P* = 0.0145) were decreased in vermis of adult ASD subjects. Spectrin repeat containing nuclear envelope protein 1 (SYNE1) which is involved in postsynaptic neurotransmitter endocytosis and postsynaptic plasticity was reduced significantly (Fig. 2) in ASD adults (log_2_ FC = -0.2891, FDR adj. *P* = 0.0036). Additional downregulated proteins of relevance to pathology of ASD in adult subjects included AFG3L2 (mitochondrial proteostasis and PC degeneration), ANK2 (ER-Golgi vesicular transport; endosomal-lysosomal marker), AP3B2 (synaptic vesicle endocytosis), DYNC1H1 (retrograde axonal transport), EEF1A2 (regulation of chaperone-mediated autophagy and protein biosynthesis), GRID2 (synaptic transmission), ITSN1 (synaptic vesicle endocytosis, exocytosis), HSP90AA1 (response to unfolded protein and stress and synaptic cycling of AMPA receptors), MACF1 (Golgi to plasma membrane transport), MAP1B (synaptic plasticity), NSF (vesicle transport and membrane fusion), SGIP1 (synaptic proteostasis), SPTBN1 (ER to Golgi vesicle mediated transport, Golgi to plasma membrane transport), TUBB2A (microtubule dynamics), TUBB3 (axon guidance), and WDR1 (actin severing).

Thirty proteins were significantly upregulated (FDR-adjusted *p* < 0.05; Fig. 6; Table 6) in adults with ASD (Tables 2 and 6, S4). Thirteen of these proteins included members of the keratin family of cytoskeletal proteins (Fig. 2; Tables 2, S4). The remainder of the upregulated proteins included ALB (many functions, i.e., oxidant detoxification and receptor mediated endocytosis), ANXA2 (vesicle budding from membrane and receptor recycling), CSTA (regulation of proteolysis), CTSD (autophagy, proteolysis), DSG1 (cell adhesion), DSP (cell-cell adhesion), ENO1 (glycolysis), GGCT (glutathione synthesis), JUP (cell-cell adhesion and signaling), MBP (myelination), PKM (glycolysis), PRDX3 (cell redox), SERPINB3 (negative regulation of proteolysis), VCAN (ER-Golgi-lysosomal function, cell adhesion) and VCL (axon extension). Many of these proteins are subject to phosphorylation and methylation and act as intermediate filaments with proclivity to formation of insoluble oligomerization and toxic interactions [25].

## Discussion

The current quantitative proteomic study has identified, evaluated, and compared the presence of 88 differentially expressed proteins (FDR-adjusted *p* < 0.05; 28 upregulated and 60 downregulated) from cerebellar vermis of children with autism vs. 71 differentially expressed proteins (FDR-adjusted *p* < 0.05; 30 upregulated and 41 downregulated) from adults with ASD.

Aberrant synaptic homeostasis and connectivity has been demonstrated in brains of subjects with autism [26–28]. Recently, we reported on involvement of proteomic abnormalities in dorsolateral prefrontal cortex of children and adults with ASD [1]. Cerebellum, a site involved in motor control and cognitive functions with extensive connections with other brain areas including thalamus and prefrontal cortex [29–31] is also involved in etiopathology of autism [7, 29–31]. Recent cross species comparison of cerebellar role in cognition during evolution of brain function in humans vs. mouse and non-mammalian species suggested that disease-specific genes related to synaptic plasticity and cognition diverged during human evolution of cerebellum [31]. Several of the genes related to autism are related to slow process of cerebellar growth which manifest in late postnatal stages of granule cell differentiation and PC maturation [31]. Indeed, genes responsible for synaptic plasticity and glutamatergic/GABAergic function are overrepresented in autistic cerebellum because of their role in cerebello-cortical circuitry and involvement in the process of axon and dendrite development, synaptic transmission, and synaptic plasticity [29, 31].

Thus, as the maturation of synaptic connections in human cerebellum is a slow and protracted process [29, 31–33] and cerebellum undergoes sensitive and vulnerable timetable of growth in human prenatal and postnatal development [29, 33], alterations in the structure or functioning of cerebellar PCs (which constitute the major GABAergic output of cerebellum to cerebellar nuclei) can have major implications on normal development of human brain. Abnormalities in genes coding for proteins that regulate the normal processes of membrane trafficking, such as clathrin-dependent endocytosis, exocytosis, neurotransmitter release, presynaptic and postsynaptic functions and autophagy, may result in development of autism or schizophrenia [26–28, 33].

Evidence for cerebellar developmental abnormalities in children with ASD included increases in protein levels for dendritic spine formation (DPYSL3) and excessive synaptic pruning (complement C3) and reductions in septin 6, a protein involved in dendritic arborization. Adults with ASD exhibited reductions in markers of dendritic arborization (septin 6, septin 11), and spine maturation (septin 11). Interestingly, PTPRZ1, a receptor for pleiotrophin (PTN) which helps in PC dendritogenesis [20, 21], was downregulated in children with ASD. Previous work [22] indicated that early disruption in PTN levels leads to abnormal cerebellar circuit formation [22]. Our data indicate that reduction in PTPRZ1 in child ASD subjects is consistent with interruption in the presence and function of PTN and PTPRZ1 during critical periods of PC development [23]. For example, in rodents, PTPRZ1 mRNA is increased between P0 and P4, persisting until P12 [23]. The protein level is high for the first two weeks postnatally, then declines. Exogenous administration of PTN during P4 and P6 leads to apoptosis of PC dendrites. Indeed, increases in spine density on apical dendrites of pyramidal neurons from layer 2 in frontal, temporal and parietal cortices have been observed in ASD brain [34] consistent with hyperconnectivity in local brain circuits with resultant overt spine density correlating positively with cognitive dysfunction in autism [34]. Moreover, brain morphology in fragile X syndrome (FXS), a variant of ASD, is also characterized with increased spine density and tortuosity of spine architecture.

**Fig. 7:**
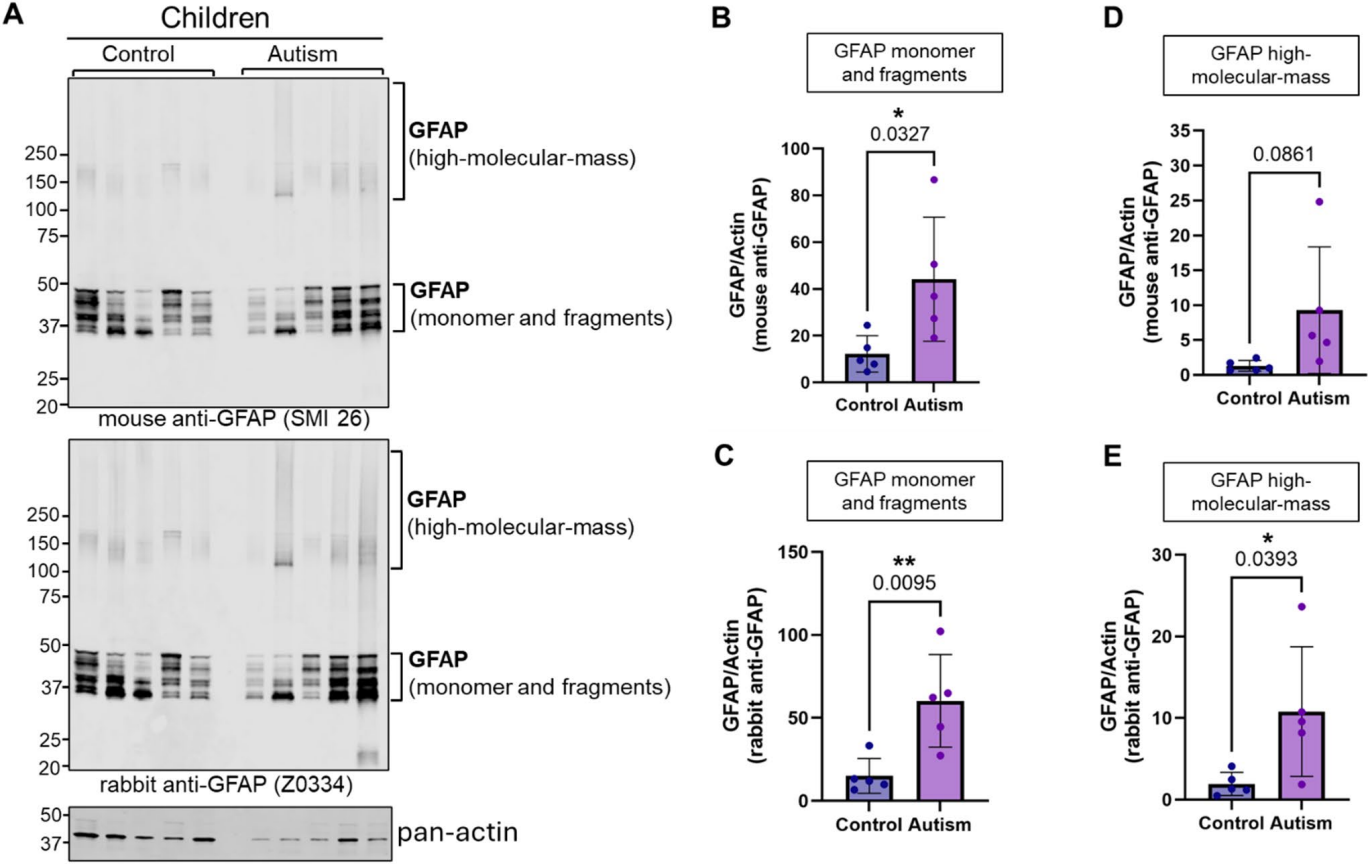
Analysis of GFAP protein expression in vermis tissue lysates from control and autism children brain donors. (**A**) Immunoblot of GFAP and pan-actin in 5 control and 5 autism subjects. Two different antibodies against GFAP were used: mouse monoclonal SMI 26 (top blot) and rabbit polyclonal Z0664 (middle blot). Pan actin blot (bottom) serves as loading control. (**B**) Quantification of GFAP monomer and fragments from SMI 26 blot. *p < 0.05; unpaired t-test. (**C**) Quantification of GFAP monomer and fragments from Z0334 blot. **p < 0.01; unpaired t-test. (**D**) Quantification of high-molecular-mass (> 100 kDa) GFAP species from SMI 26 blot. (**E**) Quantification of high-molecular-mass (> 100 kDa) GFAP species from Z0334 blot. *p < 0.05; unpaired t-test

**Fig. 8:**
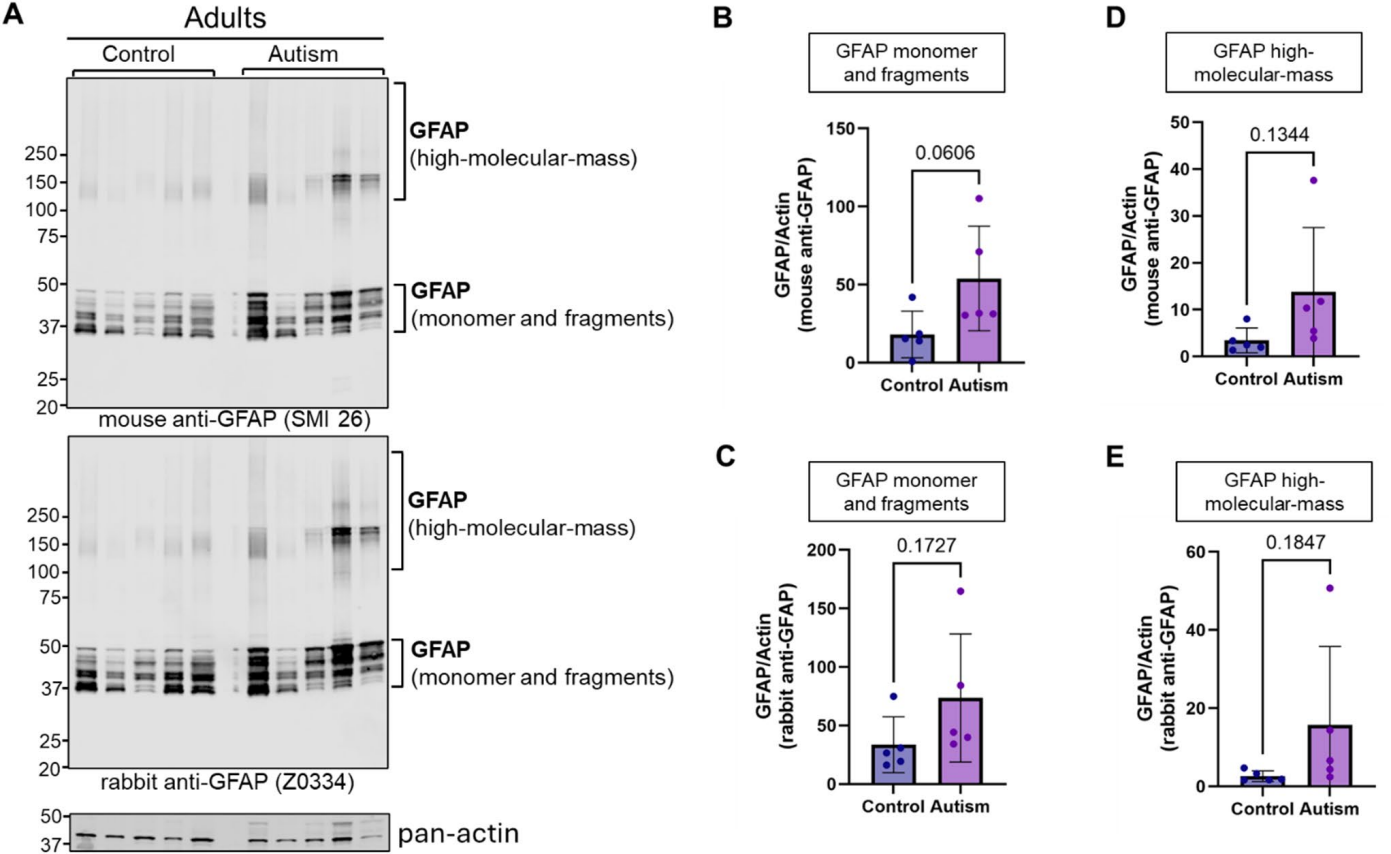
Analysis of GFAP protein expression in vermis tissue lysates from control and autism adult brain donors. (**A**) Immunoblot of GFAP and pan-actin in 5 control and 5 autism subjects. Two different antibodies against GFAP were used: mouse monoclonal SMI 26 (top blot) and rabbit polyclonal Z0664 (middle blot). Pan actin blot (bottom) serves as loading control. (**B**) Quantification of GFAP monomer and fragments from SMI 26 blot. (**C**) Quantification of GFAP monomer and fragments from Z0334 blot. (**D**) Quantification of high-molecular-mass (> 100 kDa) GFAP species from SMI 26 blot. (**E**) Quantification of high-molecular-mass (> 100 kDa) GFAP species from Z0334 blot

**Fig. 9:**
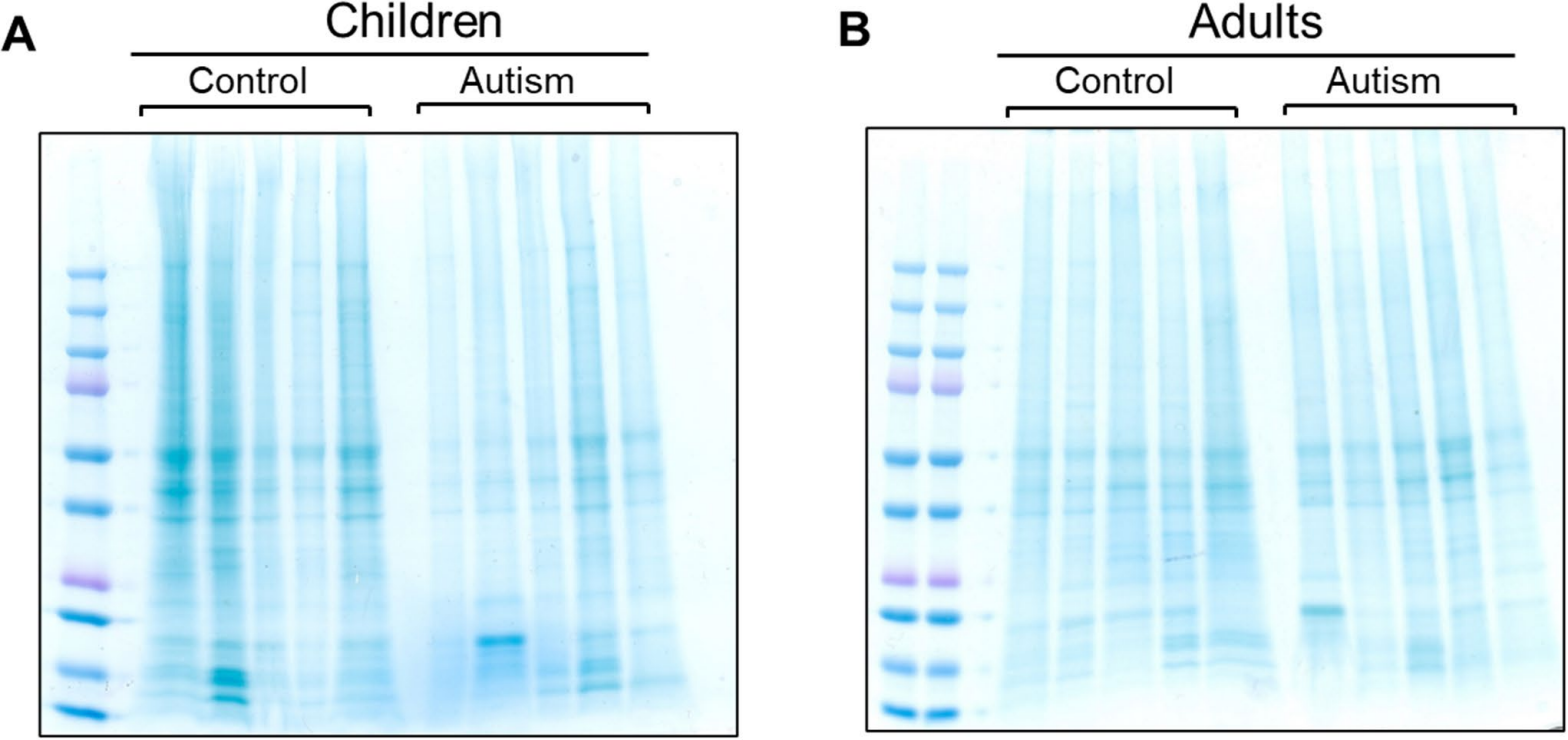
Coomassie staining analysis of total protein in vermis tissue lysates from control and autism children and adult brain donors. (**A**) Coomassie-stained gel of the same samples as those shown in Fig. 7. (**B**) Coomassie-stained gel of the same samples as those shown in Fig. 8

Recent reports suggest participation of glial and microglial cells, and oligodendrocytes in brain synaptic development and neuroinflammation [35]. Indeed, levels of glial fibrillary acidic protein (GFAP), an intracellular type III intermediate filament protein, predominantly localized to astrocytes [36, 37] and a marker of mature astrocytes, reactive gliosis and early synaptic pathology [38, 39] was elevated significantly in children with ASD (Figs. 1, 7 and 8; Tables 2 and S2). Proteomic levels of GFAP were confirmed via western blotting experiments employing two specific antibodies against GFAP (Figs. 7, 8, and 9). Interestingly, these experiments showed significant increases in monomer and high-molecular-mass forms of GFAP in children with ASD (Fig. 7). We also observed nonsignificant increases in GFAP monomer and high-molecular-mass species in adult subjects with ASD (Figs. 8 and 9). Presence of high-molecular-mass forms of GFAP in both children (Figs. 7A and E; *p* = 0.0393) and non-significantly in adults with ASD (Fig. 8A, D and E) is novel and has been reported to occur in Alexander disease [40] previously due to oxidative stress and altered redox signaling, conditions that occur in ASD brain. Overexpression of GFAP in ASD has been replicated by several other groups [10, 39, 41] indicating evidence for emergence of neuroinflammation as early as childhood in ASD [39]. Additionally, levels of myelin basic protein (MBP), released by oligodendrocytes, was increased significantly in adults with ASD. Complement C3, released by microglial activation, was also elevated in children with ASD, implicating the role of glial and oligodendroglial as well as microglial cells in etiology of synaptic pathology in ASD [10, 41].

A multitude of proteins participating in various steps in secretory pathways including ER-Golgi trafficking, intra-Golgi transport, endosome-lysosomal function, endocytosis as well as exocytosis exhibited significant alterations in children and adults with ASD (Tables 2, S1-S4). The level of a member of the serpin family of serine-type endopeptidase inhibitors, namely serpinB3, localized to ER-Golgi compartments, was elevated in adult cerebellum of subjects with ASD (Tables 2 and S4). In contrast, only serpina1 was significantly elevated in children with ASD (Table S2). Levels of multiple members of serpins are also upregulated in neurodegenerative diseases [42]. Serpina1 upregulation has been reported in brain and CSF of subjects with Creutzfeldt-Jakob disease (CJD) and frontotemporal lobar degeneration (FTLD) [43]. SerpinB3 upregulation has been associated with a number of cancers and autoimmune disorders [44]. High levels of tissue plasminogen activator (TPA) whose activity is regulated by serine protease inhibitor neuroserpin [45], has been associated with ASD [46, 47] and observed in the valproic acid model of autism [48]. It also plays a role in granule cell migration [49, 50], PC neuron development and PC survival [50–52]. Despite neuroserpin’s role in synaptic plasticity, learning and memory, its levels were not altered in fusiform gyrus of ASD patients nor in dorsolateral prefrontal cortex of subjects with schizophrenia [45]. A previous report [53] also denied presence of any polymorphisms in plasminogen activator inhibitor-1 gene in autistic disorder. However, our current data implicates a role for serpins in pathology of ASD.

GDI-1 protein level was significantly upregulated in vermis of children with ASD (Fig. 1; Tables 2 and S2). GDIs are important regulators of rho GTPase activity [54, 55] and actin dynamics with disruption in their function causing synaptic dysfunction. Gdi1-deficient mice exhibit short term memory deficits [56] and have impairments in synaptic vesicle biogenesis and recycling including defective endosomal-dependent recycling abnormalities [57]. Overexpression of GDI1 proteins leads to disruption of actin cytoskeleton, causing inactivation of rho/rab proteins. The expression levels of GDIs have been reported to be upregulated in a dosage-dependent manner in patients with mental retardation due to recurrent copy number gain at Xq28 [58, 59]. Recent reports also indicate presence of variants in GDI1 in autistic individuals [60, 61]. Mechanistically, overexpression of GDI1 and 2 proteins leads to loss of stress fibers and focal contact sites and disappearance of cell-cell adhesion belts, inactivating Rho GDP proteins and activating depolymerization of actin cytoskeleton in differentiated human keratinocytes [62], consistent with a major role of GDI1 in cytoskeletal dynamics [63, 64]. Several proteins interact with GDIs, including the ERM protein family (ezrin, radixin, and moesin) [55]. Indeed, levels of ezrin (EZR) and radixin (RDX) were upregulated significantly in children with ASD (Fig. 1; Table 2). This pathology is not restricted to ASD and has been reported to occur in neuroblastoma N2a cells transfected with mutant prion protein [65], leading to impaired post-Golgi trafficking and overexpression of GDI1 and in postmortem brain of subjects with schizophrenia [66]. Lastly, mammalian expression of GDI1 in the brain is involved in control of endocytic and exocytic vesicular pathways and Rab proteins [56, 60] with overexpression of GDI-1 in children with ASD, consistent with premature development of mature mushroom-shaped spines prior to full development of cerebellum [67].

Dynein cytoplasmic 1 heavy chain 1 (DYNC1H)1, an important protein involved in retrograde transport, was significantly reduced in both children and adults with autism (Figs. 1 and 2). DYNC1H1 is a risk gene for autism (SFARI Eagle score = 13.45) which participates in several steps in vesicular trafficking including dynein-dynactin cargo adaptor complex formation/activity, retrograde transport and cellular transport [68]. This protein functions not only in neurons but also in non-neuronal cells participating in general cellular trafficking and is localized to Golgi and endosomal compartments [68] with abnormal functioning in several neurologic disorders including Charcot-Marie-Tooth disease, intellectual disability, epileptic encephalopathies, developmental delays, autism, ALS, and Huntington’s disease [69, 70]. Interestingly, four related proteins, DYNC1I1, DYNC1LI1, DYNC1LI2 and DYNLRB1 were also reduced significantly in cerebellar vermis of children with autism (Tables 2, S1). Dyneins are microtubule-activated ATPases that function as molecular motors. Mutations in the DYNC1H1 gene are responsible for intellectual disability and neuronal migration defects [70] and play a role in neurodegenerative disorders [71].

DYNLRB1, a protein involved in endosome-Golgi-ER retrograde transport [72] was downregulated in children with ASD. DYNLRB1 is a Rab6 effector protein and thus, involved in Rab6-dependent retrograde transport [72, 73]. Recent evidence indicates that DYNLRB1 is an essential component for dynein-mediated transport [74]. Deficits in this protein cause severe impairment in the axonal transport of lysosomes and retrograde signaling endosomes. Thus, its downregulation in ASD may account for some of the critical functions that are associated with ASD synaptopathy [74].

Adaptor-related proteins (2A1, 2B1) were altered significantly in cerebellum of children with ASD (Tables 2 and S1), while adaptor-related protein 3B2 was downregulated in adults with ASD only (Tables 2 and S3). AP2A1 and AP2B1 proteins are involved in vesicle endocytosis and synaptic vesicle cycle machinery [75, 76]. AP3B2 protein is involved in neuron-specific sorting and vesicular function [77]. Spontaneous mouse mutations involving AP3 protein complex leads to defective development of lysosomes and secretory granules [77–79] and in neurons, defective synaptic vesicles [77, 80–83]. Moreover, AP3B2 deficient mice exhibit decreased zinc stores in synaptic structures [77]. AP3B2 protein deficit in adults with ASD reflects abnormalities in several vesicular trafficking steps including synaptic vesicle function, endocytosis and retrograde transport. AP3B2 variants have been associated with early-onset encephalopathy exhibiting seizures, developmental regression and intellectual disability with onset of encephalopathy before one year of age and are localized structurally to defective synaptic vesicles [84, 85]. Thus, downregulation of AP3B2 protein in adult subjects with ASD implicates defective vesicular trafficking of cargo proteins from trans-Golgi network and/or endosomes to lysosomes in autistic pathology [85]. Our current report is the first report on involvement of AP3B2 gene in etiology of autism. Furthermore, novel mutations in AP3B2 have been identified in individuals with developmental and epileptic encephalopathies [86, 87] as well as in psychosis [88]. Falk et al. [88] identified anti-AP3β2 autoantibodies in 23 patients who all reported persecutory delusions.

Two members of the GTP-binding cytoskeletal septin protein family, namely septin 3 and 6 were reduced significantly (Fig. 1) in children with autism (septin 3: log_2_ FC = -0.2572, FDR adj. *P* = 0.0024; septin 6: log_2_ FC = -0.2887, FDR adj. *P* = 0.0000). Interestingly, six members of the septin family were reduced significantly (Fig. 2) in adults with ASD (septin 3: log_2_ FC = -0.6837, FDR adj. *P* = 0.0000; septin 4: log_2_ FC = -0.5097, FDR adj. *P* = 0.0470; septin 5: log_2_ FC = -0.5202, FDR adj. *P* = 0.0145 septin 6: log_2_ FC = -0.6358, FDR adj. *P* = 0.0000; septin 9: log_2_ FC = -0.6250, FDR adj. *P* = 0.0018; septin 11: log_2_ FC = -1.0475, FDR adj. *P* = 0.0486). Septins have roles in neuronal migration, axonal, dendritic and spine development and synaptic plasticity [89]. Additionally, septins play major roles in presynaptic function of synaptic vesicle exocytosis, anterograde dendritic transport of neurotransmitters, synaptic docking and fusion as well as postsynaptic regulation of synapse organization [89].

Septin 3 is a developmentally-regulated neuron-specific protein highly enriched in the presynaptic terminals and involved in synaptic vesicle recycling and exocytosis [90]. Recent reports indicate that septin 3 is associated with the process of synaptic autophagy [91]. As the level of septin 3 increases after birth, its significant decline in ASD children reveals early developmental disruption in septin 3 function in the process of autophagy. Septin 4 is a component of presynaptic scaffold generated by both neurons and astroglia [92, 93] which is required for the suppression of alpha-synuclein neurotoxicity [93]. Thus, loss of septin 4 enhances self-aggregation of alpha-synuclein [94]. Interestingly, septin 4 may accumulate in tau-based deposits in both Alzheimer’s and Parkinson’s disease [94]. Furthermore, thermal stress can cause unfolding of septin 4 into amyloid-like filaments in vitro [94]. Thus, reduction in septin 4 in adult vermal tissue may infer susceptibility of autistic brain to amyloid aggregation and neurodegeneration [95]. Septin 5 gene is deleted in velocardiofacial syndrome (22q11.2 deletion syndrome), implicated significantly in both autism and schizophrenia [96–99]. Septin 5 gene has been associated with affective behaviors, cognition and social interaction functions, exemplified in cerebellar involvement in autism [100, 101]. Septin 5 transcripts in humans undergo alternative splicing leading to different isoforms that exhibit different temporal and brain localization [102]. Septin V1 (adult form or long form) is involved in cognition [100, 101]; phosphorylation by cyclin-dependent kinase 5, causes decrease in binding to syntaxin 1 [103, 104] and potential degradation by ubiquitin-dependent proteasome degradation protein parkin [102]. Thus, reduction in level of septin 5 in adult ASD vermis may be due to various mechanisms which are currently unknown. Interestingly, several cases of autoimmune septin 5 cerebellar ataxias have been reported whereby patients exhibit acute symptoms of loss of balance, incoordination, and speech impediment and exhibit cerebellar atrophy and neurodegeneration [105]. As septin 5 regulates synaptic vesicle docking, it may lead to abnormal neurotransmitter release due to transformation of microdomain to nanodomain coupling of calcium influx and adversely impacting synaptic active zone-release of dopamine, glutamate, and serotonin [99, 106].

Septin 6, another presynaptic protein is involved in processes of axonal outgrowth, dendritic lengthening and branching, localized at the neck of dendritic spines [107]. Septin 6 has been associated with abnormal phosphorylation in both Alzheimer’s disease and schizophrenia [108, 109]. Septin 9, a further presynaptic protein is involved in retrograde transport of lysosomes due to oxidative stress [110]. Additionally, septin 9 may participate in anterograde dendritic transport of neurotransmitter complex [111] potentially via its interaction with KIF17, a kinesin 2 family motor protein. Thus, downregulation of septin 9 in adult ASD vermis may interfere with normal dendritic transport and release of NR2B cargo at dendritic spines [110, 112, 113]. Lastly, septin 11, a postsynaptic regulator of GABAergic synapse, highly expressed by PCs of the cerebellum, and localized to dendritic spines, and involved in dendritic arborization and maturation [114] has been associated with phagosome formation process [115] and discovered in sporadic frontotemporal lobar degeneration [116]. The perturbed expression of several members of septin family of proteins in cerebellar vermis of both children and adults with ASD may reflect potential involvement of protein aggregation as a central pathologic factor observed in etiopathogenesis of neurodevelopmental disorders such as ASD, schizophrenia and Down syndrome as well as neurodegenerative disorders such as Alzheimer’s disease, Parkinson’s disease, Huntington’s and frontotemporal dementia [117, 118].

Spectrin members of the cytoskeletal scaffold proteins α2 spectrin (SPTAN1) and β3 spectrin (SPTBN2) were significantly decreased in children with autism. SPTAN1 participates in neurotransmitter release and exocytosis [119]. SPTBN2 is involved in PC morphogenesis, ER-Golgi vesicular trafficking, and synaptic function between parallel fiber and PC synapse [119, 120]. Level of SPTBN1 was reduced in adults with autism. SPTBN1 is a risk gene for ASD and participates in trafficking from ER to PSD [121–123]. Reductions in levels of this protein may cause inefficient release of neurotransmitters and abnormal transit from ER to PSD [119]. Increases in levels of SPTAN1 and SPTBN1 and their breakdown products have been observed in senile plaques in Alzheimer’s disease [124, 125] and in Parkinson’s disease Lewy body protein products [126] which could lead to aggregation of misfolded proteins and resultant neurotoxicity in brains of adult subjects with ASD.

Cathepsin D (CTSD), a marker of lysosomes [127], participates in autophagy-lysosomal pathway in Huntington’s disease [128], and previously reported to be elevated in ASD [129, 130]. Level of CTSD was elevated significantly in adults with ASD. Furthermore, level of hexokinase 1 (HK1), which participates in glycolysis and catalyzes the first rate limiting step in glycolysis via phosphorylation of glucose to glucose-6-phosphate [131] was significantly downregulated in adults with autism. HK1 variants have been identified in developmental encephalopathies [131], and Alzheimer’s disease murine model [132]. Thus, reduction in levels of proteins involved in mitochondrial function and energy production may act synergistically to impair brain function in subjects with autism.

Protocadherin FAT2 protein was downregulated in adult subjects with autism (Fig. 2). Protein with multiple EGF-like domains (MEGF1), a mammalian homologue of Drosophila Fat protein is expressed in the internal granule cell layer of cerebellum which peaks during the third postnatal week and remains at high expression levels in adult rat cerebellum [133]. FAT2 expression is restricted to the parallel fibers of the cerebellum, thus impacting the branching of PC dendrites and synapse formation process during development [133]. FAT2 protein is localized to ER, Golgi, and trans Golgi compartments of neurons and is involved in ER-Golgi transport function [133]. Furthermore, FAT2 is involved in cerebellar granule cell synaptogenesis [133]. Variants of FAT2 have been identified in females with autism [61] and in spinocerebellar ataxias [134–136].

Microtubule-actin crosslinking factor 1 (MACF1) isoform 1 was downregulated in adults with autism. MACF1 is considered a risk gene for autism (SFARI) and serves two functions in vesicular traffic, namely anterograde Golgi-associated transit and Golgi to plasma membrane trafficking [137, 138]. MACF1 is essential for proper modulation of actin and microtubule cytoskeletal network [139]. Deficits in MACF1 impair early steps in autophagy and vesicular transport from trans Golgi to the plasma membrane [139]. New evidence also points to a role of MACF1 in the etiology of bipolar disorder and psychosis [140]. Furthermore, MACF1 is involved in COPII secretory machinery and trafficking from ER to Golgi complex [141].

Several proteins involved in neurotransmitter release, PSD/glutamate function were altered significantly in children and adults with autism (Tables 2, S1, S3). Synaptotagmin 1 (SYT1) is a critical protein involved in calcium-triggered exocytosis and neurotransmitter release [142]. Synaptotagmins interact with multiple proteins involved in synaptic vesicle trafficking including AP2 adaptor protein complex, neurexins, SNAP-25 and synaptobrevin [142]. SYT1 mRNA expression is strongly positive in rat cerebellar granule cells at P0, P4-6, P13-15, P20 and adulthood, coincident with granule cell differentiation [142]. However, SYT1 mRNA expression is evident only during P4-P6 period in rat PCs [142]. There is limited expression in rat cerebellar Golgi cells during P15-P20 period [142]. SYT2 expression is strongly positive in rat cerebellar granule cells only during postnatal periods, P13-15, P20, and adulthood [142]. SYT2 expression is evident in rat PCs during postnatal periods P13-P15, P20 and adulthood [142]. SYT1 alterations have been associated with neurologic disorders [143], and autism [10, 144]. Furthermore, SYT4-like variants have also been reported in ASD [145]. Thus, reductions in levels of SYT1 in children with ASD confirm previous reports indicating presence of abnormalities in synaptotagmin family of proteins in ASD. Interestingly, levels of SH3-containing GRB2-like protein 3-interacting protein 1 (SGIP1) which plays a role in clathrin-mediated endocytosis and acts as a selective endocytic adaptor for internalization of SYT1 protein at receptor sites [146] was reduced in adults with ASD.

Synapsin 1 (SYN1), an important presynaptic phosphoprotein and member of a family of proteins involved in synaptic vesicular function [147] was downregulated in cerebellar vermis of adult subjects with idiopathic autism. Synapsin 1 is localized to the extrinsic cytoplasmic surface of synaptic vesicles and thus controls the readily releasable pool of synaptic vesicles [148]. Synapsin 1 variants have been identified in 91% of subjects with neurodevelopmental disorders [149]. Recent experimental evidence indicates lack of synapsin in triple knockout mice leading to alterations in presynaptic plasticity in hippocampal mossy fibers of male mice [147]. The synapsin KO mice exhibited reduced reserve pool of synaptic vesicles and exhibited slower replenishment of vesicular availability consistent with the role of synapsins in short term memory processing [147]. Reduction in synapsin 1 in cerebellar vermis of adults with autism may play an important role in cognitive contribution to cerebellar pathology in autism [7]. Lastly, synapsin 1 is considered a risk gene for autism [150]. SYN2 level was also downregulated in cerebellum of adults with ASD (Table S3). SYN2 is also a presynaptic protein which impacts the readily releasable pool of synaptic vesicles. Mutations in SYN1 and SYN2 have been associated with ASD [150].

A further protein involved in synaptic vesicular function downregulated in adults with autism (Table 2, S3) was N-ethylmaleimide sensitive factor protein (NSF). NSF is involved in synaptic vesicle functioning, synaptic docking and fusion, and exocytosis [151–153]. NSF is considered a member of PSD and acts as a serotonin transporter binding protein. Reduction of NSF levels have been identified in a mouse model of autism [153, 154].

Syntaxin binding protein 1 (n-Sec 1, Munc 18.1, STXBP1) was significantly reduced in cerebellar vermis of adult subjects with autism (Table S3). There is increased expression of this protein in cerebellum [155]. STXBP1 participates in several aspects of vesicular trafficking including synaptic vesicle docking and fusion [156], neurotransmitter release [157], and exocytosis [156]. Indeed, docking of secretory vesicles is syntaxin-dependent which implicates the important role of STXBP1 [158]. It is not surprising that abnormalities in expression of this protein can lead to a diverse number of brain disorders including a developmental group of brain disorders identified as STXBP1 encephalopathies [159]. Recent experimental evidence identifies subtypes of GABAergic/glycinergic and glutamatergic STXBP1 encephalopathies [159]. Clinically, STXBP1 encephalopathy symptoms include intellectual disability, epilepsy, and motor dysfunctions [159]. Additionally, 19–42% of patients with STXBP1 abnormalities present with autistic-like features [157, 160]. As participation of NSF and STXBP1 is a requirement for normal exocytosis to occur [156], reductions in levels of both proteins in cerebellar vermis of adult subjects with ASD, indicate presence of abnormal vesicle docking machinery as the most probable mechanism of insufficient neurotransmitter release being contributory to pathologic function of cerebellum in idiopathic autism. Supportive experimental evidence in Munc 18 − 1 haploinsufficient mice indicates presence of cognitive impairment and abnormal synaptic plasticity due to less readily available synaptic vesicles for neurotransmitter release being consistent with causation of cognitive deficits in autism [161].

Prohibitin 2 (PHB2), an inner mitochondrial membrane mitophagy receptor [162] was upregulated in vermis of children with ASD (Tables 2, S2). Increase in PHB2 level has been associated with neurodegeneration [163] and oligodendrocyte-associated mitophagy [164]. There is also evidence for increased PHB levels in Rett’s syndrome [165] and involvement of PHB2 in several neuropsychiatric disorders including ASD [166].

Eight members of the T-complex protein-1 ring complex (TRiC/CCT) family of protein folding machinery were reduced significantly in children with ASD (Tables 2, S1) (TCP1, CCT2, CCT3, CCT5, CCT6A, CCT7, CCT8, FDR-adjusted *p* < 0.05). Recent reports [167, 168] provide evidence for presence of variants in several TRiC/CCT members including CCT1, CCT3, CCT5, CCT6A, CCT7 and CCT8, in brain malformations, seizures, and intellectual disability. Interestingly, homozygous mutants with CCT3 mutations showed absence of cerebellar granule cells and exhibited malpositioned PCs and F-actin disorganization [167]. Several subjects with brain malformation displayed ataxia, cerebellar atrophy (including cerebellar vermis) and hypomyelination [167]. Proteomic analysis of patient-derived fibroblasts demonstrated downregulation of lysosomal, ubiquitination-related, mitochondrial, metabolic proteins [167] and upregulated proteins associated with proteosomal and stress-related chaperones [167]. Interestingly, we observed overexpression of group I chaperonins (HSPD1, HSPA1B) in cerebellar vermis of children with ASD and downregulation of HSP90AA1 in vermis of adults with ASD, implicating involvement of chaperone-mediated protein misfolding and defective autophagy process in etiology of ASD. Thus, it might be that altered levels of chaperonins, heat shock proteins, and abnormal proteostasis in ASD leads to increased insoluble aggregates, mimicking a scenario similar to that seen in Alzheimer’s disease, Parkinson’s disease, Huntington’s disease [169, 170], and schizophrenia [171, 172].

Several proteins were significantly downregulated in both children and adults with ASD which exhibited enrichment in pathways related to neurodegeneration (Tables S1-S3, S5, S7) including DYNC1H1 (children and adults, [71]), FARSB (children and adults, [173]), FAT2 (adults, [136]), SYNE1 (children and adults, [174]), AFG3L2 (adults, [175]), GRID2 (adults, [176]), HK1 (adults, [132]), and MAP1B (adults, [177]), implicating involvement of neurodegenerative processes in cerebellar vermis of children and adults with ASD, a finding previously reported in ASD [9]. Lastly, we identified several proteins considered detergent-insoluble in proteomic studies which lead to protein aggregation and subsequent cellular neurodegeneration [178–183]. In children, the putative insoluble proteins included overexpressed GDI-1, GFAP, Plec1, Psat1, TPI-1 and underexpressed proteins CS, KRT9 and SFPQ (Figs. 1, 2, 3, 4, 5, 6, 7, 8 and 9; Tables 2, S1, S2). In adults overexpressed proteins included KRT1, KRT9, KRT10, KRT16, MBP, CTSD, and PKM; downregulated proteins consisted of SPTBN1, STXBP1, SYN1, and MDH2 (Figs. 1, 2, 3, 4, 5 and 6; Tables 2, S3, S4). Thus, even downregulated proteins have the potential to cause insolubility and aggregation in the vermis of ASD subjects.

Recent evidence indicates that synaptic proteins may transition in their molecular conformation from liquid to gel and lastly to an amyloid state under various physiologic or pathologic conditions such as oxidative stress [184, 185]. As described earlier, several identified brain proteins in ASD brains can undergo aggregation and misassembly in endoplasmic reticulum or other cellular compartments and convert from liquid states to an amyloid-like conformation and be involved in a spectrum of neuropsychiatric disorders i.e., neurodevelopmental disorders such as autism and schizophrenia [171], neurodevelopmental-neurodegenerative disorders such as Down syndrome and neurodegenerative disorders such as Alzheimer’s disease, Parkinson’s disorder, and Huntington’s disease [184]. These so-called protein condensation diseases which may associate with aggrephagy, ER stress, and protein conformation abnormalities, if treated early, may be targets of potential new therapies [184]. As suggested by Nucifora et al. [171] some subtypes of schizophrenia exhibit protein aggregation and insolubility when examined by biochemical assays. Abraham et al. [9] presented evidence to support shared converging pathways between neurodegenerative processes and autism. We have provided evidence for significant alterations in levels of several proteins which are susceptible to aggregation. Several of these proteins such as GDI-1, GFAP (Table 2; Figs. 7, 8 and 9), and MBP have been implicated in pathology of schizophrenia and ASD [39, 41, 66] via neuronal-glial interactions. Additionally, many reports indicate that increase in GFAP may be a marker of neuroinflammation, neurodegeneration and abnormal cognition in Alzheimer’s disease and Parkinson’s disease [38]. Thus, it is possible that in a heterogeneous disorder such as ASD, some subtypes of autism, may exhibit misfolding, aggrephagy, protein aggregation, and potentially, neurodegeneration. Indeed, three recent large-scale epidemiologic studies provide evidence for linkage between dementia and ASD [186–188]. Vivanti et al. [186] showed an increased risk for development of early-onset dementia in subjects with ASD. Yin et al. [187] showed a four-fold increase in risk of developing Parkinson’s disease in ASD subjects. Finally, Chang et al. [188] showed presence of familial co-aggregation between ASD and different types of dementias. Additionally, transcriptomic and proteomic studies provide genetic linkage between neurodegeneration and ASD [9, 189]. Lastly, identification of significant alterations in levels of several synaptic proteins related to neurodegeneration in our cerebellar data (Figs. 1, 2, 3, 4, 5, 6, 7 and 8; Table 2) are based on highly significant (FDR-adjusted *p* < 0.05) protein changes in children and adults with ASD. Finally, two recent reports show significant increases in α-synuclein in plasma of subjects with ASD [190, 191], connoting evidence for presence of a marker for neurodegeneration (similar to Parkinson’s disease) in subjects with ASD.

The current study has several limitations. The statistical power of the study was limited due to scarcity of well-characterized cerebellar vermis of children and adults with idiopathic ASD. A further limitation of this study pertains to inadequate supply of female subjects mostly due to lower prevalence of ASD in female subjects [192]. Thus, the results of the current proteomic study should be considered preliminary until future validation studies with higher power can be carried out to confirm our proteomic results.

In conclusion, proteomic investigation of the cerebellar vermis in children and adults with idiopathic autism demonstrates evidence for age-dependent synaptic network dysfunction based on involvement of multiple etiopathologic mechanisms. Children with ASD exhibited abnormal dendritic formation, elimination, maturation, and arborization (DYPSL3, C3, PTPRZ1, septin 6), downregulation of Rho signaling proteins (ARHGAP26, DOCK1, ITPR1, NCKAP1, DYNLRB1), downregulation of protein synthetic machinery involved in transcription (HNRNPK, HNRNPD, EEF1A2, EiF3L, SFPQ), translation (AFG3L2, RPLP2), protein assembly and folding (CCT1, CCT2, CCT3, CCT5, CCT6A, CCT7, CCT8), downregulation of protein degradation (PSMD13, septin 3, septin 6), interruption in vesicular trafficking (AP2A1, AP2B1, DYNC1H1, DYNCII1, DYNC1LI1, DYNC1LI2, DYNLRB1, SPTAN1), oxidative stress (GLO1, ALDH2, ECPAS), abnormal speech development (NOVA1), proteinopathy (RBMX, HNRNPD, HNRNPK) and aggrephagy (DYNC1H1 family). Adults with ASD exhibited downregulation of dendritic spine formation and arborization (septin 6, septin 11), downregulation of synaptic neurotransmission (STXBP1, SYNE1, SYN1, SYN2, septin 4), downregulation of synaptic vesicle docking, fusion, and recycling (septin 5, septin 11, SYN1, NSF, AP3B2, STXBP1), clathrin-mediated endocytosis, retrograde vesicle transport, retrograde lysosome transport and exocytosis (DNM1, DNM3, serpinb3, AP3B2, SYNE1, DYNC1H1, septin 9, septin 3, septin 6, septin 11), synaptic proteostasis (SGIP1), abnormal autophagy (CTSD), abnormal myelination (MBP, MDH2), abnormal protein aggregation (KRT1, KRT9, KRT10, KRT16, CTSD, SPTBN1, STXBP1, SYN1, PKM) and neurodegeneration (AFG3L2, DYNCIH1, FARSB, FAT2, GRID2, HK1, SYNE1, MAP1B). Childhood-specific abnormalities included impaired Rho signaling, neuroinflammation, oxidative stress, retrograde transport deficits, synaptic pruning and protein folding pathways. Adult-specific abnormal pathways consisted of myelination process. However, shared etiological mechanisms occurring in children and adults with ASD included dendritic spine formation and arborization, interrupted vesicular trafficking, aggrephagy and autophagy, synaptic neurotransmission, protein synthesis and degradation, cell redox and homeostatic balance, and actin-microtubule/intermediate filament and cytoskeletal abnormalities. While abnormalities of protein synthesis and dendritic pruning were more extensive in children, synaptic deficits in neurotransmission, protein aggregation and neurodegenerative processes predominated in adult subjects with ASD. These abnormalities and associated cytoskeletal dysfunction lead to impairment of cognitive processes in cerebellum of subjects with autism. These novel results point to etiology of autism as a developmental brain disorder which may be amenable to therapeutic interventions focusing on reduction of oxidative stress and ameliorating defective process of proteostasis [184, 193].

## Electronic Supplementary Material

Below is the link to the electronic supplementary material.

Supplementary Material 1: Tables S1, S2, S3, S4, S5, S6, S7, S8, S9, S10)

## Data Availability

The mass spectrometry proteomics data have been deposited to the ProteomeXchange Consortium via the PRiDE partner repository with the dataset identifier PXD061746 and doi: 10.6019/PXD061746.
