# Supplementary material for: Impaired Aggrephagy, Interrupted Vesicular Trafficking, and Cellular Stress, Lead to Protein Aggregation, and Synaptic Dysfunction in Cerebellum of Children and Adults with Idiopathic Autism
Source: Cerebellum. 2025 Aug 8;24(5):140. doi: 10.1007/s12311-025-01880-5 (PMC12334520; doi:10.1007/s12311-025-01880-5)
Supplement: Supplementary file 1 — Supplementary Material 1: Tables S1, S2, S3, S4, S5, S6, S7, S8, S9, S10) [file 12311_2025_1880_MOESM1_ESM.pdf]

Supplementary Data for "Impaired aggrephagy, interrupted vesicular trafficking, and cellular stress lead to protein aggregation, and synaptic dysfunction in cerebellum of children and adults with idiopathic autism"

S. Hossein Fatemi, Timothy Folsom, Arthur Eschenlauer, and Thierry Chekouo

List of Tables

S1 Statistically significant downregulated cerebellar vermis proteins from children with idiopathic autism (FDR-adjusted $p < 0.05$) . . . 2

S2 Statistically significant upregulated cerebellar vermis proteins from children with idiopathic autism (FDR-adjusted $p < 0.05$) . . . 4

S3 Statistically significant downregulated cerebellar vermis proteins from adults with idiopathic autism (FDR-adjusted $p < 0.05$) . . . 5

S4 Statistically significant upregulated cerebellar vermis proteins from adults with idiopathic autism (FDR-adjusted $p < 0.05$) . . . 6

S5 Cerebellar vermis pathways enriched in downregulated proteins (FDR-adjusted $p < 0.05$) of children with idiopathic autism . . . 7

S6 Cerebellar vermis pathways enriched in upregulated proteins (FDR-adjusted $p < 0.05$) of children with idiopathic autism . . . 60

S7 Cerebellar vermis pathways enriched in downregulated proteins (FDR-adjusted $p < 0.05$) of adults with idiopathic autism . . . 83

S8 Cerebellar vermis pathways enriched in upregulated proteins (FDR-adjusted $p < 0.05$) of adults with idiopathic autism . . . 132

S9 Non-significant synaptic proteins of cerebellar vermis in children with idiopathic autism (FDR-adjusted $p > 0.05$) . . . 164

S10 Non-significant synaptic proteins of cerebellar vermis in adults with idiopathic autism (FDR-adjusted $p > 0.05$) . . . 224

Table S1: Statistically significant downregulated cerebellar vermis proteins from children with idiopathic autism (FDR-adjusted $p < 0.05$)

Gene name	FDR-adjusted p-value	log2(fold-change)	Number of peptides
ACADVL	0.0006	-0.2817	12
ACAT1	0.0418	-0.2664	10
ANK1	0.0432	-0.3235	13
AP2A1	0.0001	-0.3085	16
AP2B1	0.0000	-0.2810	24
API5	0.0262	-0.6408	3
ARHGAP26	0.0135	-0.5554	7
CAPZA2	0.0269	-0.3252	4
CCT2	0.0000	-0.4278	19
CCT3	0.0004	-0.3842	13
CCT4	0.0418	-0.2842	11
CCT5	0.0000	-0.4475	15
CCT6A	0.0000	-0.3976	15
CCT7	0.0000	-0.3470	19
CCT8	0.0000	-0.3525	19
CS	0.0008	-0.4204	12
DDX1	0.0001	-0.6256	10
DDX17	0.0002	-0.6300	9
DIP2B	0.0329	-0.3060	7
DOCK1	0.0262	-0.4147	4
DYNC1H1	0.0000	-0.3429	68
DYNC1I1	0.0200	-0.9228	2
DYNC1LI1	0.0004	-0.4543	9
DYNC1LI2	0.0169	-0.3114	5
DYNLRB1	0.0329	-0.6930	2
ECPAS	0.0063	-0.6796	3
EEF1A2	0.0075	-0.3081	17
EIF3L	0.0276	-0.5591	1
FARSB	0.0056	-0.4516	12
HNRNPD	0.0200	-0.5395	4
HNRNPK	0.0000	-0.7101	12
IGLON5	0.0200	-0.5452	2
INPP5A	0.0217	-0.5419	4
ITPR1	0.0000	-0.7994	18
IVD	0.0480	-0.3757	5
JUP	0.0197	-0.2758	19
KRT9	0.0000	-0.4298	29
NCKAP1	0.0000	-0.3838	14
NCL	0.0002	-0.5680	14
NOVA1	0.0112	-0.4070	8
PABPC1	0.0161	-0.4146	7
PIK3C3	0.0329	-0.3325	6
PRKDC	0.0089	-0.4706	10
PSMD13	0.0012	-0.3883	8
PSPC1	0.0112	-0.8666	4

Table S1: Statistically significant downregulated cerebellar vermis proteins from children with idiopathic autism (FDR-adjusted $p < 0.05$) *(continued)*

Gene name	FDR-adjusted p-value	log2(fold-change)	Number of peptides
PTPRZ1	0.0037	-0.3529	15
RBMX	0.0064	-0.5777	5
RPLP0	0.0329	-0.4206	5
RPLP2	0.0118	-0.4459	6
RTRAF	0.0463	-0.5008	4
SEPTIN3	0.0024	-0.2572	10
SEPTIN6	0.0000	-0.2887	11
SFPQ	0.0060	-0.5659	8
SPTAN1	0.0026	-0.1410	52
SPTBN2	0.0036	-0.3789	14
SYNE1	0.0276	-0.2454	27
SYT1	0.0026	-0.5145	7
TCP1	0.0004	-0.3454	14
UBASH3B	0.0418	-0.3771	2
XRCC5	0.0013	-0.6853	10

Table S2: Statistically significant upregulated cerebellar vermis proteins from children with idiopathic autism (FDR-adjusted $p < 0.05$)

Gene name	FDR-adjusted p-value	log2(fold-change)	Number of peptides
AHNAK	0.0104	0.2605	18
ALDH1L1	0.0026	0.4287	12
ALDH2	0.0131	0.4913	5
ALDH7A1	0.0446	0.3363	9
ANXA5	0.0129	0.6052	5
C3	0.0253	0.4385	6
DPYSL3	0.0003	0.7780	7
ENO1	0.0000	0.5764	16
EZR	0.0463	0.8450	4
GDI1	0.0043	0.4589	9
GFAP	0.0000	0.5389	32
GLO1	0.0482	0.3631	3
GSN	0.0112	0.3848	12
H2AFY	0.0482	0.6486	4
HADHA	0.0021	0.4236	20
HSPA1B	0.0020	0.2537	22
HSPD1	0.0276	0.1546	15
NNT	0.0060	0.5507	13
NPEPPS	0.0095	0.4629	9
PEPD	0.0340	0.4650	1
PHB2	0.0135	0.2694	13
PLEC	0.0010	0.2170	54
PRDX1	0.0075	0.2678	14
PSAT1	0.0026	0.6024	10
RDX	0.0430	0.4171	8
SERPINA1	0.0131	0.4431	7
TPI1	0.0051	0.4459	14
VCL	0.0432	0.2454	13

Table S3: Statistically significant downregulated cerebellar vermis proteins from adults with idiopathic autism (FDR-adjusted $p < 0.05$)

Gene name	FDR-adjusted p-value	log2(fold-change)	Number of peptides
AFG3L2	0.0098	-0.4447	5
ANK2	0.0000	-0.4011	32
AP3B2	0.0125	-0.3571	8
CEP76	0.0090	-0.4614	3
DNM1	0.0001	-0.4164	30
DNM3	0.0146	-0.4124	13
DYNC1H1	0.0000	-0.2651	70
EEF1A2	0.0067	-0.3589	17
FARSB	0.0050	-0.4512	12
FAT2	0.0078	-0.4169	14
GRID2	0.0047	-0.7604	6
HK1	0.0000	-0.7169	30
HSP90AA1	0.0239	-0.3009	21
ITSN1	0.0239	-0.3944	10
KRAS	0.0421	-0.5395	4
LRRC40	0.0287	-0.6032	3
MACF1	0.0121	-0.3087	15
MAP1B	0.0000	-0.2997	42
MDH2	0.0051	-0.4009	15
MPP1	0.0332	-0.7572	2
MPP6	0.0145	-0.3349	10
NSF	0.0275	-0.2482	28
PSPC1	0.0372	-0.4602	6
SEPTIN11	0.0486	-1.0475	3
SEPTIN3	0.0000	-0.6837	10
SEPTIN4	0.0470	-0.5097	5
SEPTIN5	0.0145	-0.5202	7
SEPTIN6	0.0000	-0.6358	13
SEPTIN9	0.0018	-0.6250	8
SGIP1	0.0486	-0.5882	4
SPTBN1	0.0401	-0.1794	42
STXBP1	0.0313	-0.3065	21
SYN1	0.0000	-0.6779	17
SYN2	0.0146	-0.4951	9
SYNE1	0.0036	-0.2891	25
TAGLN3	0.0313	-0.4560	9
TUBB2A	0.0000	-0.3946	30
TUBB3	0.0239	-0.3206	18
WDR1	0.0214	-0.3592	8
XRCC5	0.0002	-0.4802	11
XRCC6	0.0005	-0.4269	11

Table S4: Statistically significant upregulated cerebellar vermis proteins from adults with idiopathic autism (FDR-adjusted $p < 0.05$)

Gene name	FDR-adjusted p-value	log2(fold-change)	Number of peptides
ALB	0.0007	0.5085	27
ANXA2	0.0018	0.7694	5
ARG1	0.0051	0.7649	7
CSTA	0.0364	0.8370	3
CTSD	0.0012	0.4411	12
DSG1	0.0332	0.5700	6
DSP	0.0000	0.6419	64
ENO1	0.0332	0.4504	10
GGCT	0.0380	0.9093	4
IGHM	0.0024	0.8111	6
JUP	0.0000	0.6548	22
KRT1	0.0000	0.7310	54
KRT10	0.0000	0.7397	20
KRT14	0.0000	0.7445	27
KRT16	0.0170	0.5648	13
KRT17	0.0139	0.5725	12
KRT2	0.0000	0.7856	34
KRT5	0.0000	0.6803	21
KRT6A	0.0170	0.7534	7
KRT6B	0.0000	0.6970	21
KRT77	0.0002	0.6731	13
KRT78	0.0051	0.7782	8
KRT80	0.0239	0.5392	9
KRT9	0.0000	0.7080	30
MBP	0.0241	0.4059	17
PKM	0.0168	0.3151	22
PRDX3	0.0332	0.6838	4
SERPINB3	0.0016	0.8967	7
VCAN	0.0026	0.6465	7
VCL	0.0036	0.3613	13

Table S5: Cerebellar vermis pathways enriched in downregulated proteins (FDR-adjusted $p < 0.05$) of children with idiopathic autism

Category	Term ID	Term description	Gene count	Back-ground	Strength	Signal	FDR	Matching proteins in network
GO Process	GO:1904851	Positive regulation of establishment of protein localization to telomere	8	10	2.42	3.75	3.0e-12	CCT2, CCT3, CCT4, CCT5, CCT6A, CCT7, CCT8, TCP1
GO Process	GO:1904871	Positive regulation of protein localization to Cajal body	8	11	2.38	3.74	3.0e-12	CCT2, CCT3, CCT4, CCT5, CCT6A, CCT7, CCT8, TCP1
GO Process	GO:1904874	Positive regulation of telomerase RNA localization to Cajal body	8	15	2.24	3.61	4.7e-12	CCT2, CCT3, CCT4, CCT5, CCT6A, CCT7, CCT8, TCP1
GO Process	GO:0032212	Positive regulation of telomere maintenance via telomerase	9	34	1.94	3.29	1.1e-11	CCT2, CCT3, CCT4, CCT5, CCT6A, CCT7, CCT8, TCP1, XRCC5
GO Process	GO:0032206	Positive regulation of telomere maintenance	10	67	1.69	2.88	3.7e-11	CCT2, CCT3, CCT4, CCT5, CCT6A, CCT7, CCT8, HNRNPD, TCP1, XRCC5
GO Process	GO:2001252	Positive regulation of chromosome organization	11	107	1.53	2.60	6.9e-11	CCT2, CCT3, CCT4, CCT5, CCT6A, CCT7, CCT8, HNRNPD, SFPQ, TCP1, XRCC5
GO Process	GO:2000278	Regulation of DNA biosynthetic process	10	124	1.42	2.07	7.2e-09	CCT2, CCT3, CCT4, CCT5, CCT6A, CCT7, CCT8, HNRNPD, TCP1, XRCC5
GO Process	GO:1900182	Positive regulation of protein localization to nucleus	9	91	1.51	2.11	1.5e-08	CCT2, CCT3, CCT4, CCT5, CCT6A, CCT7, CCT8, JUP, TCP1
GO Process	GO:0033044	Regulation of chromosome organization	12	253	1.19	1.71	1.6e-08	CCT2, CCT3, CCT4, CCT5, CCT6A, CCT7, CCT8, DYNC1LI1, HNRNPD, SFPQ, TCP1, XRCC5
GO Process	GO:0007339	Binding of sperm to zona pellucida	7	39	1.77	2.17	6.0e-08	CCT2, CCT3, CCT4, CCT5, CCT7, CCT8, TCP1

Table S5: Cerebellar vermis pathways enriched in downregulated proteins (FDR-adjusted $p < 0.05$) of children with idiopathic autism *(continued)*

Category	Term ID	Term description	Gene count	Back-ground	Strength	Signal	FDR	Matching proteins in network
GO Process	GO:0061077	Chaperone-mediated protein folding	8	71	1.57	2.01	7.0e-08	CCT2, CCT3, CCT4, CCT5, CCT6A, CCT7, CCT8, TCP1
GO Process	GO:1901998	Toxin transport	7	42	1.74	2.11	8.8e-08	CCT2, CCT3, CCT4, CCT5, CCT7, CCT8, TCP1
GO Process	GO:0010638	Positive regulation of organelle organization	14	508	0.96	1.25	2.4e-07	CCT2, CCT3, CCT4, CCT5, CCT6A, CCT7, CCT8, DYNC1H1, HNRNPD, NCKAP1, SFPQ, SYT1, TCP1, XRCC5
GO Process	GO:0051054	Positive regulation of DNA metabolic process	11	304	1.07	1.29	1.4e-06	CCT2, CCT3, CCT4, CCT5, CCT6A, CCT7, CCT8, HNRNPD, PRKDC, TCP1, XRCC5
GO Process	GO:0033043	Regulation of organelle organization	18	1190	0.70	0.83	5.4e-06	CAPZA2, CCT2, CCT3, CCT4, CCT5, CCT6A, CCT7, CCT8, DYNC1H1, DYNC1LI1, HNRNPD, NCKAP1, SFPQ, SPTAN1, SPTBN2, SYT1, TCP1, XRCC5
GO Process	GO:0050821	Protein stabilization	8	215	1.09	0.95	1.9e-04	CCT2, CCT3, CCT4, CCT5, CCT6A, CCT7, CCT8, TCP1
GO Process	GO:0051130	Positive regulation of cellular component organization	15	1049	0.67	0.67	2.2e-04	CCT2, CCT3, CCT4, CCT5, CCT6A, CCT7, CCT8, DYNC1H1, HNRNPD, HNRNPK, NCKAP1, SFPQ, SYT1, TCP1, XRCC5
GO Process	GO:1904951	Positive regulation of establishment of protein localization	9	319	0.97	0.85	2.7e-04	CCT2, CCT3, CCT4, CCT5, CCT6A, CCT7, CCT8, JUP, TCP1
GO Process	GO:0031647	Regulation of protein stability	9	328	0.95	0.83	3.2e-04	CCT2, CCT3, CCT4, CCT5, CCT6A, CCT7, CCT8, PRKDC, TCP1
GO Process	GO:0051972	Regulation of telomerase activity	5	51	1.51	1.06	3.2e-04	CCT2, CCT4, HNRNPD, TCP1, XRCC5

Table S5: Cerebellar vermis pathways enriched in downregulated proteins (FDR-adjusted $p < 0.05$) of children with idiopathic autism *(continued)*

Category	Term ID	Term description	Gene count	Back-ground	Strength	Signal	FDR	Matching proteins in network
GO Process	GO:0090666	scaRNA localization to Cajal body	3	5	2.29	1.08	5.9e-04	CCT2, CCT4, TCP1
GO Process	GO:0010557	Positive regulation of macromolecule biosynthetic process	19	1935	0.51	0.50	1.0e-03	CCT2, CCT3, CCT4, CCT5, CCT6A, CCT7, CCT8, DDX17, HNRNPD, HNRNPK, JUP, NCL, PABPC1, PRKDC, RBMX, RTRAF, SFPQ, TCP1, XRCC5
GO Process	GO:0070201	Regulation of establishment of protein localization	10	528	0.79	0.64	1.6e-03	CCT2, CCT3, CCT4, CCT5, CCT6A, CCT7, CCT8, JUP, PIK3C3, TCP1
GO Process	GO:0051973	Positive regulation of telomerase activity	4	34	1.59	0.87	1.8e-03	CCT2, CCT4, TCP1, XRCC5
GO Process	GO:0031328	Positive regulation of cellular biosynthetic process	19	2041	0.49	0.46	2.1e-03	CCT2, CCT3, CCT4, CCT5, CCT6A, CCT7, CCT8, DDX17, HNRNPD, HNRNPK, JUP, NCL, PABPC1, PRKDC, RBMX, RTRAF, SFPQ, TCP1, XRCC5
GO Process	GO:0045935	Positive regulation of nucleobase-containing compound metabolic process	19	2056	0.48	0.46	2.3e-03	CCT2, CCT3, CCT4, CCT5, CCT6A, CCT7, CCT8, DDX17, HNRNPD, HNRNPK, JUP, NCL, PABPC1, PRKDC, RBMX, RTRAF, SFPQ, TCP1, XRCC5

Table S5: Cerebellar vermis pathways enriched in downregulated proteins (FDR-adjusted p < 0.05) of children with idiopathic autism *(continued)*

Category	Term ID	Term description	Gene count	Back-ground	Strength	Signal	FDR	Matching proteins in network
GO Process	GO:0009987	Cellular process	58	14826	0.11	0.27	2.7e-03	ACADVL, ACAT1, ANK1, AP2A1, AP2B1, API5, ARHGAP26, CAPZA2, CCT2, CCT3, CCT4, CCT5, CCT6A, CCT7, CCT8, CS, DDX1, DDX17, DIP2B, DOCK1, DYNC1H1, DYNC1I1, DYNC1LI1, DYNC1LI2, DYNLRB1, ECPAS, EEF1A2, EIF3L, FARSB, HNRNPD, HNRNPK, INPP5A, ITPR1, IVD, JUP, KRT9, NCKAP1, NCL, NOVA1, PABPC1, PIK3C3, PRKDC, PSMD13, PTPRZ1, RBMX, RPLP0, RPLP2, RTRAF, SEPTIN3, SEPTIN6, SFPQ, SPTAN1, SPTBN2, SYNE1, SYT1, TCP1, UBASH3B, XRCC5
GO Process	GO:0065008	Regulation of biological quality	26	3654	0.37	0.39	2.8e-03	ACADVL, AP2A1, AP2B1, CAPZA2, CCT2, CCT3, CCT4, CCT5, CCT6A, CCT7, CCT8, DIP2B, DYNC1H1, HNRNPD, ITPR1, JUP, NCKAP1, NOVA1, PABPC1, PRKDC, RPLP0, SPTAN1, SPTBN2, SYT1, TCP1, UBASH3B
GO Process	GO:0000398	mRNA splicing, via spliceosome	7	245	0.97	0.65	3.4e-03	DDX1, DDX17, HNRNPK, NOVA1, PABPC1, RBMX, SFPQ

Table S5: Cerebellar vermis pathways enriched in downregulated proteins (FDR-adjusted p < 0.05) of children with idiopathic autism *(continued)*

Category	Term ID	Term description	Gene count	Back-ground	Strength	Signal	FDR	Matching proteins in network
GO Process	GO:0051128	Regulation of cellular component organization	20	2365	0.44	0.42	3.9e-03	CAPZA2, CCT2, CCT3, CCT4, CCT5, CCT6A, CCT7, CCT8, DIP2B, DYNC1H1, DYNC1LI1, HNRNPD, HNRNPK, NCKAP1, SFPQ, SPTAN1, SPTBN2, SYT1, TCP1, XRCC5
GO Process	GO:0008380	RNA splicing	8	370	0.85	0.58	4.9e-03	DDX1, DDX17, HNRNPK, NOVA1, PABPC1, RBMX, RTRAF, SFPQ
GO Process	GO:0071840	Cellular component organization or biogenesis	33	5639	0.28	0.34	4.9e-03	ANK1, ARHGAP26, CAPZA2, CCT2, CCT3, CCT8, DDX1, DDX17, DIP2B, DOCK1, DYNC1H1, DYNC1LI1, DYNC1LI2, ECPAS, EIF3L, FARSB, JUP, KRT9, NCKAP1, PIK3C3, PRKDC, PSMD13, PTPRZ1, RBMX, RPLP0, SEPTIN6, SFPQ, SPTAN1, SPTBN2, SYNE1, SYT1, TCP1, XRCC5
GO Process	GO:0016043	Cellular component organization	32	5436	0.29	0.33	6.4e-03	ANK1, ARHGAP26, CAPZA2, CCT2, CCT3, CCT8, DDX1, DIP2B, DOCK1, DYNC1H1, DYNC1LI1, DYNC1LI2, ECPAS, EIF3L, FARSB, JUP, KRT9, NCKAP1, PIK3C3, PRKDC, PSMD13, PTPRZ1, RBMX, RPLP0, SEPTIN6, SFPQ, SPTAN1, SPTBN2, SYNE1, SYT1, TCP1, XRCC5

Table S5: Cerebellar vermis pathways enriched in downregulated proteins (FDR-adjusted $p < 0.05$) of children with idiopathic autism *(continued)*

Category	Term ID	Term description	Gene count	Back-ground	Strength	Signal	FDR	Matching proteins in network
GO Process	GO:0022607	Cellular component assembly	20	2467	0.43	0.39	6.7e-03	CAPZA2, CCT2, CCT3, CCT8, DDX1, DYNC1H1, ECPAS, EIF3L, FARSB, JUP, NCKAP1, PIK3C3, PRKDC, RBMX, RPLP0, SEPTIN6, SPTBN2, SYT1, TCP1, XRCC5
GO Process	GO:0043933	Protein-containing complex organization	15	1465	0.53	0.43	6.8e-03	CAPZA2, CCT2, CCT3, CCT8, DDX1, ECPAS, EIF3L, FARSB, PIK3C3, PRKDC, RBMX, RPLP0, SYT1, TCP1, XRCC5
GO Process	GO:0048024	Regulation of mRNA splicing, via spliceosome	5	110	1.17	0.64	6.8e-03	DDX17, HNRNPK, NCL, NOVA1, RBMX
GO Process	GO:0044085	Cellular component biogenesis	21	2702	0.41	0.38	7.0e-03	CAPZA2, CCT2, CCT3, CCT8, DDX1, DDX17, DYNC1H1, ECPAS, EIF3L, FARSB, JUP, NCKAP1, PIK3C3, PRKDC, RBMX, RPLP0, SEPTIN6, SPTBN2, SYT1, TCP1, XRCC5
GO Process	GO:0065003	Protein-containing complex assembly	14	1303	0.55	0.43	7.6e-03	CAPZA2, CCT2, CCT3, CCT8, DDX1, ECPAS, EIF3L, FARSB, PRKDC, RBMX, RPLP0, SYT1, TCP1, XRCC5
GO Process	GO:0022618	Ribonucleoprotein complex assembly	6	203	0.99	0.56	9.9e-03	DDX1, EIF3L, PRKDC, RBMX, RPLP0, XRCC5
GO Process	GO:1903311	Regulation of mRNA metabolic process	7	302	0.88	0.53	9.9e-03	DDX17, HNRNPD, HNRNPK, NCL, NOVA1, PABPC1, RBMX
GO Process	GO:0002218	Activation of innate immune response	4	65	1.31	0.58	0.013	PRKDC, PSPC1, SFPQ, XRCC5
GO Process	GO:0034462	Small-subunit processome assembly	2	3	2.34	0.57	0.021	PRKDC, XRCC5

Table S5: Cerebellar vermis pathways enriched in downregulated proteins (FDR-adjusted p < 0.05) of children with idiopathic autism *(continued)*

Category	Term ID	Term description	Gene count	Back-ground	Strength	Signal	FDR	Matching proteins in network
GO Process	GO:0051179	Localization	27	4512	0.29	0.28	0.030	ANK1, AP2A1, AP2B1, CCT2, CCT3, CCT4, CCT5, CCT7, CCT8, DDX1, DOCK1, DYNC1H1, DYNC1I1, DYNC1LI2, ITPR1, JUP, PIK3C3, RPLP0, RTRAF, SEPTIN3, SEPTIN6, SFPQ, SPTBN2, SYNE1, SYT1, TCP1, XRCC5
GO Process	GO:0060341	Regulation of cellular localization	11	963	0.57	0.35	0.032	CCT2, CCT3, CCT4, CCT5, CCT6A, CCT7, CCT8, DYNC1H1, JUP, PIK3C3, TCP1
GO Process	GO:1903312	Negative regulation of mRNA metabolic process	4	92	1.15	0.42	0.045	HNRNPD, HNRNPK, PABPC1, RBMX
GO Function	GO:0140662	ATP-dependent protein folding chaperone	8	31	1.93	2.72	1.3e-09	CCT2, CCT3, CCT4, CCT5, CCT6A, CCT7, CCT8, TCP1
GO Function	GO:0003723	RNA binding	26	1672	0.71	1.03	1.6e-09	API5, CCT3, CCT4, CCT5, CCT6A, CS, DDX1, DDX17, DYNC1H1, DYNC1LI1, EIF3L, FARSB, HNRNPD, HNRNPK, NCL, NOVA1, PABPC1, PRKDC, PSPC1, RBMX, RPLP0, RTRAF, SFPQ, SYNE1, TCP1, XRCC5
GO Function	GO:0140657	ATP-dependent activity	14	543	0.93	1.14	1.1e-06	CCT2, CCT3, CCT4, CCT5, CCT6A, CCT7, CCT8, DDX1, DDX17, DYNC1H1, DYNC1I1, DYNLRB1, TCP1, XRCC5
GO Function	GO:0051082	Unfolded protein binding	8	115	1.36	1.47	3.5e-06	CCT2, CCT3, CCT4, CCT5, CCT6A, CCT7, CCT8, TCP1

Table S5: Cerebellar vermis pathways enriched in downregulated proteins (FDR-adjusted p < 0.05) of children with idiopathic autism *(continued)*

Category	Term ID	Term description	Gene count	Background	Strength	Signal	FDR	Matching proteins in network
GO Function	GO:0043168	Anion binding	25	2404	0.53	0.65	8.6e-06	ACADVL, ACAT1, CCT2, CCT3, CCT4, CCT5, CCT6A, CCT7, CCT8, DDX1, DDX17, DYNC1H1, DYNC1LI1, DYNC1LI2, EEF1A2, FARSB, ITPR1, IVD, PIK3C3, PRKDC, SEPTIN3, SEPTIN6, SYT1, TCP1, XRCC5
GO Function	GO:0032555	Purine ribonucleotide binding	22	1903	0.58	0.69	1.3e-05	ACADVL, ACAT1, CCT2, CCT3, CCT4, CCT5, CCT6A, CCT7, CCT8, DDX1, DDX17, DYNC1H1, DYNC1LI1, DYNC1LI2, EEF1A2, FARSB, PIK3C3, PRKDC, SEPTIN3, SEPTIN6, TCP1, XRCC5
GO Function	GO:0000166	Nucleotide binding	23	2168	0.54	0.64	1.8e-05	ACADVL, ACAT1, CCT2, CCT3, CCT4, CCT5, CCT6A, CCT7, CCT8, DDX1, DDX17, DYNC1H1, DYNC1LI1, DYNC1LI2, EEF1A2, FARSB, IVD, PIK3C3, PRKDC, SEPTIN3, SEPTIN6, TCP1, XRCC5

Table S5: Cerebellar vermis pathways enriched in downregulated proteins (FDR-adjusted p < 0.05) of children with idiopathic autism *(continued)*

Category	Term ID	Term description	Gene count	Back-ground	Strength	Signal	FDR	Matching proteins in network
GO Function	GO:0005515	Protein binding	43	7242	0.29	0.44	1.8e-05	ACADVL, ACAT1, ANK1, AP2A1, AP2B1, API5, CAPZA2, CCT2, CCT3, CCT4, CCT5, CCT6A, CCT7, CCT8, DIP2B, DOCK1, DYNC1H1, DYNC1I1, DYNC1LI1, DYNC1LI2, DYNLRB1, EEF1A2, HNRNPD, HNRNPK, INPP5A, ITPR1, IVD, JUP, NCL, PABPC1, PRKDC, PTPRZ1, RBMX, RTRAF, SEPTIN3, SFPQ, SPTAN1, SPTBN2, SYNE1, SYT1, TCP1, UBASH3B, XRCC5
GO Function	GO:0016887	ATP hydrolysis activity	10	333	0.99	1.03	2.8e-05	CCT2, CCT3, CCT4, CCT5, CCT6A, CCT7, CCT8, DDX1, DDX17, TCP1
GO Function	GO:0017111	Nucleoside-triphosphatase activity	13	650	0.82	0.88	2.8e-05	CCT2, CCT3, CCT4, CCT5, CCT6A, CCT7, CCT8, DDX1, DDX17, EEF1A2, SEPTIN3, SEPTIN6, TCP1
GO Function	GO:0032559	Adenyl ribonucleotide binding	19	1554	0.60	0.68	3.3e-05	ACADVL, ACAT1, CCT2, CCT3, CCT4, CCT5, CCT6A, CCT7, CCT8, DDX1, DDX17, DYNC1H1, DYNC1LI1, DYNC1LI2, FARSB, PIK3C3, PRKDC, TCP1, XRCC5

Table S5: Cerebellar vermis pathways enriched in downregulated proteins (FDR-adjusted p < 0.05) of children with idiopathic autism *(continued)*

Category	Term ID	Term description	Gene count	Back-ground	Strength	Signal	FDR	Matching proteins in network
GO Function	GO:0036094	Small molecule binding	24	2507	0.50	0.59	3.5e-05	ACADVL, ACAT1, CCT2, CCT3, CCT4, CCT5, CCT6A, CCT7, CCT8, DDX1, DDX17, DYNC1H1, DYNC1LI1, DYNC1LI2, EEF1A2, FARSB, ITPR1, IVD, PIK3C3, PRKDC, SEPTIN3, SEPTIN6, TCP1, XRCC5
GO Function	GO:0035639	Purine ribonucleoside triphosphate binding	20	1834	0.55	0.62	6.3e-05	CCT2, CCT3, CCT4, CCT5, CCT6A, CCT7, CCT8, DDX1, DDX17, DYNC1H1, DYNC1LI1, DYNC1LI2, EEF1A2, FARSB, PIK3C3, PRKDC, SEPTIN3, SEPTIN6, TCP1, XRCC5
GO Function	GO:1901363	Heterocyclic compound binding	37	5977	0.31	0.43	1.2e-04	ACADVL, ACAT1, API5, CCT2, CCT3, CCT4, CCT5, CCT6A, CCT7, CCT8, CS, DDX1, DDX17, DYNC1H1, DYNC1LI1, DYNC1LI2, EEF1A2, EIF3L, FARSB, HNRNPD, HNRNPK, IVD, NCL, NOVA1, PABPC1, PIK3C3, PRKDC, PSPC1, RBMX, RPLP0, RTRAF, SEPTIN3, SEPTIN6, SFPQ, SYNE1, TCP1, XRCC5

Table S5: Cerebellar vermis pathways enriched in downregulated proteins (FDR-adjusted $p < 0.05$) of children with idiopathic autism *(continued)*

Category	Term ID	Term description	Gene count	Background	Strength	Signal	FDR	Matching proteins in network
GO Function	GO:0097159	Organic cyclic compound binding	37	6050	0.30	0.42	1.6e-04	ACADVL, ACAT1, API5, CCT2, CCT3, CCT4, CCT5, CCT6A, CCT7, CCT8, CS, DDX1, DDX17, DYNC1H1, DYNC1LI1, DYNC1LI2, EEF1A2, EIF3L, FARSB, HNRNPD, HNRNPK, IVD, NCL, NOVA1, PABPC1, PIK3C3, PRKDC, PSPC1, RBMX, RPLP0, RTRAF, SEPTIN3, SEPTIN6, SFPQ, SYNE1, TCP1, XRCC5
GO Function	GO:0005524	ATP binding	17	1491	0.57	0.59	3.0e-04	CCT2, CCT3, CCT4, CCT5, CCT6A, CCT7, CCT8, DDX1, DDX17, DYNC1H1, DYNC1LI1, DYNC1LI2, FARSB, PIK3C3, PRKDC, TCP1, XRCC5

Table S5: Cerebellar vermis pathways enriched in downregulated proteins (FDR-adjusted $p < 0.05$) of children with idiopathic autism *(continued)*

Category	Term ID	Term description	Gene count	Back-ground	Strength	Signal	FDR	Matching proteins in network
GO Function	GO:0005488	Binding	55	12838	0.15	0.31	3.9e-04	ACADVL, ACAT1, ANK1, AP2A1, AP2B1, API5, ARHGAP26, CAPZA2, CCT2, CCT3, CCT4, CCT5, CCT6A, CCT7, CCT8, CS, DDX1, DDX17, DIP2B, DOCK1, DYNC1H1, DYNC1I1, DYNC1LI1, DYNC1LI2, DYNLRB1, ECPAS, EEF1A2, EIF3L, FARSB, HNRNPD, HNRNPK, INPP5A, ITPR1, IVD, JUP, NCL, NOVA1, PABPC1, PIK3C3, PRKDC, PSPC1, PTPRZ1, RBMX, RPLP0, RTRAF, SEPTIN3, SEPTIN6, SFPQ, SPTAN1, SPTBN2, SYNE1, SYT1, TCP1, UBASH3B, XRCC5
GO Function	GO:0003676	Nucleic acid binding	27	4003	0.35	0.38	2.7e-03	API5, CCT3, CCT4, CCT5, CCT6A, CS, DDX1, DDX17, DYNC1H1, DYNC1LI1, EEF1A2, EIF3L, FARSB, HNRNPD, HNRNPK, NCL, NOVA1, PABPC1, PRKDC, PSPC1, RBMX, RPLP0, RTRAF, SFPQ, SYNE1, TCP1, XRCC5
GO Function	GO:0045504	Dynein heavy chain binding	3	14	1.85	0.82	3.1e-03	DYNC1I1, DYNC1LI1, DYNC1LI2
GO Function	GO:0003729	mRNA binding	7	326	0.85	0.51	0.011	CCT5, HNRNPD, HNRNPK, NCL, NOVA1, PABPC1, RBMX
GO Function	GO:0060090	Molecular adaptor activity	7	385	0.78	0.41	0.029	ANK1, AP2A1, ECPAS, JUP, SEPTIN3, SEPTIN6, SYNE1

Table S5: Cerebellar vermis pathways enriched in downregulated proteins (FDR-adjusted $p < 0.05$) of children with idiopathic autism *(continued)*

Category	Term ID	Term description	Gene count	Back-ground	Strength	Signal	FDR	Matching proteins in network
GO Function	GO:0042162	Telomeric DNA binding	3	37	1.43	0.46	0.039	HNRNPD, NCL, XRCC5
GO Function	GO:0003730	mRNA 3-UTR binding	4	102	1.11	0.41	0.047	CCT5, HNRNPD, NOVA1, PABPC1
GO Component	GO:0005832	Chaperonin-containing T-complex	8	10	2.42	4.03	3.9e-13	CCT2, CCT3, CCT4, CCT5, CCT6A, CCT7, CCT8, TCP1
GO Component	GO:0032991	Protein-containing complex	45	5506	0.43	0.67	6.2e-11	AP2A1, AP2B1, API5, CAPZA2, CCT2, CCT3, CCT4, CCT5, CCT6A, CCT7, CCT8, DDX1, DDX17, DOCK1, DYNC1H1, DYNC1I1, DYNC1LI1, DYNC1LI2, DYNLRB1, ECPAS, EEF1A2, EIF3L, FARSB, HNRNPD, HNRNPK, ITPR1, JUP, NCKAP1, NCL, PABPC1, PIK3C3, PRKDC, PSMD13, RBMX, RPLP0, RPLP2, RTRAF, SEPTIN3, SEPTIN6, SFPQ, SPTAN1, SPTBN2, SYNE1, TCP1, XRCC5

Table S5: Cerebellar vermis pathways enriched in downregulated proteins (FDR-adjusted $p < 0.05$) of children with idiopathic autism *(continued)*

Category	Term ID	Term description	Gene count	Back-ground	Strength	Signal	FDR	Matching proteins in network
GO Component	GO:0043232	Intracellular non-membrane-bounded organelle	43	5191	0.43	0.67	2.3e-10	ACADVL, ANK1, ARHGAP26, CAPZA2, CCT2, CCT3, CCT4, CCT5, CCT6A, CCT7, CCT8, DDX1, DDX17, DYNC1H1, DYNC1I1, DYNC1LI1, DYNC1LI2, DYNLRB1, ECPAS, EIF3L, HNRNPK, ITPR1, JUP, KRT9, NCKAP1, NCL, NOVA1, PABPC1, PIK3C3, PRKDC, PSPC1, RBMX, RPLP0, RPLP2, RTRAF, SEPTIN3, SEPTIN6, SFPQ, SPTAN1, SPTBN2, SYNE1, TCP1, XRCC5
GO Component	GO:0099080	Supramolecular complex	24	1366	0.76	1.15	2.3e-10	ANK1, CCT2, CCT3, CCT4, CCT5, CCT6A, CCT7, CCT8, DDX1, DYNC1H1, DYNC1I1, DYNC1LI1, DYNC1LI2, DYNLRB1, HNRNPK, JUP, KRT9, NCKAP1, NCL, PABPC1, RPLP0, SEPTIN6, SYNE1, TCP1
GO Component	GO:0005856	Cytoskeleton	28	2369	0.59	0.83	1.2e-08	ANK1, ARHGAP26, CAPZA2, CCT2, CCT3, CCT4, CCT5, CCT6A, CCT7, CCT8, DYNC1H1, DYNC1I1, DYNC1LI1, DYNC1LI2, DYNLRB1, ECPAS, HNRNPK, JUP, KRT9, NCKAP1, PIK3C3, RTRAF, SEPTIN3, SEPTIN6, SPTAN1, SPTBN2, SYNE1, TCP1

Table S5: Cerebellar vermis pathways enriched in downregulated proteins (FDR-adjusted p < 0.05) of children with idiopathic autism *(continued)*

Category	Term ID	Term description	Gene count	Back-ground	Strength	Signal	FDR	Matching proteins in network
GO Component	GO:0031982	Vesicle	35	3957	0.46	0.66	2.6e-08	ACAT1, AP2A1, AP2B1, CAPZA2, CCT2, CCT3, CCT4, CCT5, CCT6A, CCT7, CCT8, CS, DIP2B, DYNC1H1, DYNC1I1, DYNC1LI1, DYNC1LI2, ECPAS, HNRNPK, ITPR1, JUP, KRT9, NCKAP1, NCL, PABPC1, PIK3C3, PSMD13, RBMX, RPLP0, RPLP2, SEPTIN6, SPTAN1, SYT1, TCP1, XRCC5
GO Component	GO:0002199	Zona pellucida receptor complex	5	8	2.31	2.37	6.3e-08	CCT2, CCT3, CCT4, CCT8, TCP1
GO Component	GO:0099512	Supramolecular fiber	18	1000	0.77	1.02	1.4e-07	ANK1, CCT2, CCT3, CCT4, CCT5, CCT6A, CCT7, CCT8, DYNC1H1, DYNC1I1, DYNC1LI1, DYNC1LI2, DYNLRB1, JUP, KRT9, NCKAP1, SYNE1, TCP1
GO Component	GO:0099513	Polymeric cytoskeletal fiber	16	757	0.84	1.11	1.4e-07	CCT2, CCT3, CCT4, CCT5, CCT6A, CCT7, CCT8, DYNC1H1, DYNC1I1, DYNC1LI1, DYNC1LI2, DYNLRB1, JUP, KRT9, NCKAP1, TCP1
GO Component	GO:0005874	Microtubule	13	453	0.97	1.28	1.7e-07	CCT2, CCT3, CCT4, CCT5, CCT6A, CCT7, CCT8, DYNC1H1, DYNC1I1, DYNC1LI1, DYNC1LI2, DYNLRB1, TCP1

Table S5: Cerebellar vermis pathways enriched in downregulated proteins (FDR-adjusted $p < 0.05$) of children with idiopathic autism *(continued)*

Category	Term ID	Term description	Gene count	Back-ground	Strength	Signal	FDR	Matching proteins in network
GO Component	GO:0005737	Cytoplasm	56	12056	0.18	0.38	2.2e-06	ACADVL, ACAT1, ANK1, AP2A1, AP2B1, API5, ARHGAP26, CAPZA2, CCT2, CCT3, CCT4, CCT5, CCT6A, CCT7, CCT8, CS, DDX1, DDX17, DIP2B, DOCK1, DYNC1H1, DYNC1I1, DYNC1LI1, DYNC1LI2, DYNLRB1, ECPAS, EEF1A2, EIF3L, FARSB, HNRNPD, HNRNPK, ITPR1, IVD, JUP, KRT9, NCKAP1, NCL, NOVA1, PABPC1, PIK3C3, PRKDC, PSMD13, PSPC1, RPLP0, RPLP2, RTRAF, SEPTIN3, SEPTIN6, SFPQ, SPTAN1, SPTBN2, SYNE1, SYT1, TCP1, UBASH3B, XRCC5
GO Component	GO:0005868	Cytoplasmic dynein complex	5	23	1.85	1.75	2.7e-06	DYNC1H1, DYNC1I1, DYNC1LI1, DYNC1LI2, DYNLRB1
GO Component	GO:0070062	Extracellular exosome	23	2096	0.56	0.70	2.7e-06	ACAT1, CAPZA2, CCT2, CCT3, CCT4, CCT5, CCT6A, CCT7, CCT8, CS, DIP2B, DYNC1H1, HNRNPK, JUP, KRT9, NCKAP1, NCL, PABPC1, RBMX, RPLP0, RPLP2, SPTAN1, TCP1

Table S5: Cerebellar vermis pathways enriched in downregulated proteins (FDR-adjusted $p < 0.05$) of children with idiopathic autism *(continued)*

Category	Term ID	Term description	Gene count	Back-ground	Strength	Signal	FDR	Matching proteins in network
GO Component	GO:0005829	Cytosol	37	5438	0.35	0.50	4.0e-06	ANK1, AP2A1, AP2B1, ARHGAP26, CAPZA2, CCT2, CCT3, CCT4, CCT5, CCT6A, CCT7, CCT8, DDX1, DDX17, DOCK1, DYNC1H1, DYNC1I1, DYNC1LI1, DYNC1LI2, EIF3L, FARSB, HNRNPD, JUP, KRT9, NCKAP1, PABPC1, PIK3C3, PRKDC, PSMD13, RPLP0, RPLP2, RTRAF, SFPQ, SPTAN1, SPTBN2, TCP1, XRCC5
GO Component	GO:0015630	Microtubule cytoskeleton	18	1355	0.64	0.76	7.0e-06	CCT2, CCT3, CCT4, CCT5, CCT6A, CCT7, CCT8, DYNC1H1, DYNC1I1, DYNC1LI1, DYNC1LI2, DYNLRB1, ECPAS, RTRAF, SEPTIN3, SEPTIN6, SPTAN1, TCP1
GO Component	GO:1990904	Ribonucleoprotein complex	13	687	0.79	0.89	1.3e-05	API5, DDX1, DDX17, EIF3L, HNRNPD, HNRNPK, NCL, PABPC1, PRKDC, RBMX, RPLP0, RPLP2, XRCC5

Table S5: Cerebellar vermis pathways enriched in downregulated proteins (FDR-adjusted $p < 0.05$) of children with idiopathic autism *(continued)*

Category	Term ID	Term description	Gene count	Back-ground	Strength	Signal	FDR	Matching proteins in network
GO Component	GO:0043229	Intracellular organelle	56	13231	0.14	0.32	1.2e-04	ACADVL, ACAT1, ANK1, AP2A1, AP2B1, API5, ARHGAP26, CAPZA2, CCT2, CCT3, CCT4, CCT5, CCT6A, CCT7, CCT8, CS, DDX1, DDX17, DIP2B, DOCK1, DYNC1H1, DYNC1I1, DYNC1LI1, DYNC1LI2, DYNLRB1, ECPAS, EEF1A2, EIF3L, HNRNPD, HNRNPK, ITPR1, IVD, JUP, KRT9, NCKAP1, NCL, NOVA1, PABPC1, PIK3C3, PRKDC, PSMD13, PSPC1, RBMX, RPLP0, RPLP2, RTRAF, SEPTIN3, SEPTIN6, SFPQ, SPTAN1, SPTBN2, SYNE1, SYT1, TCP1, UBASH3B, XRCC5
GO Component	GO:0005576	Extracellular region	28	4175	0.34	0.41	8.3e-04	ACAT1, CAPZA2, CCT2, CCT3, CCT4, CCT5, CCT6A, CCT7, CCT8, CS, DIP2B, DYNC1H1, HNRNPK, IGLON5, JUP, KRT9, NCKAP1, NCL, PABPC1, PSMD13, PTPRZ1, RBMX, RPLP0, RPLP2, SPTAN1, SPTBN2, TCP1, XRCC5

Table S5: Cerebellar vermis pathways enriched in downregulated proteins (FDR-adjusted p < 0.05) of children with idiopathic autism *(continued)*

Category	Term ID	Term description	Gene count	Back-ground	Strength	Signal	FDR	Matching proteins in network
GO Component	GO:0030054	Cell junction	19	2115	0.47	0.49	8.3e-04	AP2A1, AP2B1, ARHGAP26, EEF1A2, HNRNPD, HNRNPK, ITPR1, JUP, NCKAP1, PABPC1, PTPRZ1, RPLP0, RPLP2, SEPTIN3, SEPTIN6, SPTAN1, SPTBN2, SYNE1, SYT1
GO Component	GO:0036464	Cytoplasmic ribonucleoprotein granule	7	249	0.97	0.76	9.2e-04	DDX1, DYNC1I1, HNRNPK, NCL, PABPC1, RPLP0, SYNE1
GO Component	GO:0005615	Extracellular space	24	3247	0.39	0.43	9.4e-04	ACAT1, CAPZA2, CCT2, CCT3, CCT4, CCT5, CCT6A, CCT7, CCT8, CS, DIP2B, DYNC1H1, HNRNPK, JUP, KRT9, NCKAP1, NCL, PABPC1, RBMX, RPLP0, RPLP2, SPTAN1, SPTBN2, TCP1
GO Component	GO:0005813	Centrosome	10	609	0.73	0.63	1.1e-03	CCT4, CCT5, CCT8, DYNC1H1, DYNC1LI1, DYNC1LI2, DYNLRB1, ECPAS, RTRAF, TCP1

Table S5: Cerebellar vermis pathways enriched in downregulated proteins (FDR-adjusted $p < 0.05$) of children with idiopathic autism *(continued)*

Category	Term ID	Term description	Gene count	Back-ground	Strength	Signal	FDR	Matching proteins in network
GO Component	GO:0043227	Membrane-bounded organelle	54	13188	0.13	0.28	2.1e-03	ACADVL, ACAT1, ANK1, AP2A1, AP2B1, API5, CAPZA2, CCT2, CCT3, CCT4, CCT5, CCT6A, CCT7, CCT8, CS, DDX1, DDX17, DIP2B, DOCK1, DYNC1H1, DYNC1I1, DYNC1LI1, DYNC1LI2, DYNLRB1, ECPAS, EEF1A2, EIF3L, HNRNPD, HNRNPK, ITPR1, IVD, JUP, KRT9, NCKAP1, NCL, NOVA1, PABPC1, PIK3C3, PRKDC, PSMD13, PSPC1, RBMX, RPLP0, RPLP2, RTRAF, SEPTIN6, SFPQ, SPTAN1, SPTBN2, SYNE1, SYT1, TCP1, UBASH3B, XRCC5
GO Component	GO:0005938	Cell cortex	7	304	0.88	0.64	2.6e-03	CAPZA2, DYNC1H1, NCL, SEPTIN3, SEPTIN6, SPTAN1, SPTBN2
GO Component	GO:1902494	Catalytic complex	15	1539	0.51	0.46	2.8e-03	DYNC1H1, DYNC1I1, DYNC1LI1, DYNC1LI2, DYNLRB1, ECPAS, FARSB, HNRNPK, ITPR1, PABPC1, PIK3C3, PRKDC, PSMD13, RBMX, XRCC5
GO Component	GO:0042995	Cell projection	19	2379	0.42	0.42	3.1e-03	ANK1, AP2A1, CCT4, CCT8, DIP2B, DYNC1H1, DYNLRB1, HNRNPK, INPP5A, NCKAP1, PABPC1, PIK3C3, RPLP0, SEPTIN3, SEPTIN6, SFPQ, SPTAN1, SPTBN2, SYT1

Table S5: Cerebellar vermis pathways enriched in downregulated proteins (FDR-adjusted $p < 0.05$) of children with idiopathic autism *(continued)*

Category	Term ID	Term description	Gene count	Back-ground	Strength	Signal	FDR	Matching proteins in network
GO Component	GO:0030141	Secretory granule	11	873	0.62	0.51	3.3e-03	CCT2, CCT8, DYNC1H1, DYNC1LI1, ITPR1, JUP, PSMD13, SPTAN1, SYT1, TCP1, XRCC5
GO Component	GO:0034774	Secretory granule lumen	7	321	0.85	0.61	3.3e-03	CCT2, CCT8, DYNC1H1, JUP, PSMD13, SPTAN1, XRCC5
GO Component	GO:0044297	Cell body	9	570	0.71	0.55	3.3e-03	CCT2, CCT3, CCT4, CCT5, CCT7, CCT8, DIP2B, SPTBN2, TCP1
GO Component	GO:0005622	Intracellular anatomical structure	57	14891	0.10	0.26	3.4e-03	ACADVL, ACAT1, ANK1, AP2A1, AP2B1, API5, ARHGAP26, CAPZA2, CCT2, CCT3, CCT4, CCT5, CCT6A, CCT7, CCT8, CS, DDX1, DDX17, DIP2B, DOCK1, DYNC1H1, DYNC1I1, DYNC1LI1, DYNC1LI2, DYNLRB1, ECPAS, EEF1A2, EIF3L, FARSB, HNRNPD, HNRNPK, ITPR1, IVD, JUP, KRT9, NCKAP1, NCL, NOVA1, PABPC1, PIK3C3, PRKDC, PSMD13, PSPC1, RBMX, RPLP0, RPLP2, RTRAF, SEPTIN3, SEPTIN6, SFPQ, SPTAN1, SPTBN2, SYNE1, SYT1, TCP1, UBASH3B, XRCC5
GO Component	GO:0099503	Secretory vesicle	12	1047	0.58	0.49	3.4e-03	CCT2, CCT8, DYNC1H1, DYNC1LI1, ITPR1, JUP, PSMD13, SEPTIN6, SPTAN1, SYT1, TCP1, XRCC5

Table S5: Cerebellar vermis pathways enriched in downregulated proteins (FDR-adjusted p < 0.05) of children with idiopathic autism *(continued)*

Category	Term ID	Term description	Gene count	Back-ground	Strength	Signal	FDR	Matching proteins in network
GO Component	GO:0031410	Cytoplasmic vesicle	19	2482	0.40	0.39	4.6e-03	AP2A1, AP2B1, CCT2, CCT4, CCT8, DYNC1H1, DYNC1I1, DYNC1LI1, DYNC1LI2, ECPAS, ITPR1, JUP, PIK3C3, PSMD13, SEPTIN6, SPTAN1, SYT1, TCP1, XRCC5
GO Component	GO:0070418	DNA-dependent protein kinase complex	2	4	2.22	0.75	6.1e-03	PRKDC, XRCC5
GO Component	GO:0106002	mCRD-mediated mRNA stability complex	2	5	2.12	0.70	8.2e-03	HNRNPD, PABPC1
GO Component	GO:0005925	Focal adhesion	7	416	0.74	0.47	0.012	ARHGAP26, HNRNPK, JUP, NCKAP1, PABPC1, RPLP0, RPLP2
GO Component	GO:0030122	AP-2 adaptor complex	2	7	1.97	0.63	0.013	AP2A1, AP2B1
GO Component	GO:0005681	Spliceosomal complex	5	197	0.92	0.50	0.015	API5, HNRNPK, NCL, PABPC1, RBMX
GO Component	GO:0042382	Paraspeckles	2	8	1.91	0.60	0.015	PSPC1, SFPQ
GO Component	GO:0072669	tRNA-splicing ligase complex	2	8	1.91	0.60	0.015	DDX1, RTRAF
GO Component	GO:0098562	Cytoplasmic side of membrane	5	207	0.90	0.49	0.017	ANK1, AP2A1, AP2B1, EEF1A2, JUP
GO Component	GO:0008091	Spectrin	2	9	1.86	0.58	0.018	SPTAN1, SPTBN2
GO Component	GO:0014731	Spectrin-associated cytoskeleton	2	9	1.86	0.58	0.018	ANK1, SPTBN2
GO Component	GO:0070419	Nonhomologous end joining complex	2	9	1.86	0.58	0.018	PRKDC, XRCC5
GO Component	GO:0045202	Synapse	12	1350	0.47	0.34	0.024	AP2A1, AP2B1, EEF1A2, HNRNPD, ITPR1, PTPRZ1, RPLP0, SEPTIN3, SEPTIN6, SPTBN2, SYNE1, SYT1

Table S5: Cerebellar vermis pathways enriched in downregulated proteins (FDR-adjusted p < 0.05) of children with idiopathic autism *(continued)*

Category	Term ID	Term description	Gene count	Back-ground	Strength	Signal	FDR	Matching proteins in network
GO Component	GO:0005730	Nucleolus	10	996	0.52	0.35	0.028	ACADVL, DDX17, EIF3L, ITPR1, NCL, NOVA1, PRKDC, PSPC1, SYNE1, XRCC5
GO Component	GO:0098687	Chromosomal region	6	365	0.73	0.40	0.030	DYNC1I1, DYNC1LI1, DYNC1LI2, PRKDC, SEPTIN6, XRCC5
GO Component	GO:0005940	Septin ring	2	14	1.67	0.48	0.034	SEPTIN3, SEPTIN6
GO Component	GO:0031105	Septin complex	2	14	1.67	0.48	0.034	SEPTIN3, SEPTIN6
GO Component	GO:0070013	Intracellular organelle lumen	29	5660	0.23	0.25	0.034	ACADVL, ACAT1, API5, CCT2, CCT4, CCT8, CS, DDX1, DDX17, DOCK1, DYNC1H1, ECPAS, EIF3L, HNRNPD, HNRNPK, ITPR1, IVD, JUP, NCL, NOVA1, PRKDC, PSMD13, PSPC1, RBMX, RTRAF, SFPQ, SPTAN1, SYNE1, XRCC5
GO Component	GO:0016020	Membrane	41	9523	0.15	0.23	0.041	ACADVL, ANK1, AP2A1, AP2B1, API5, CAPZA2, CS, DDX1, DDX17, DIP2B, DOCK1, DYNC1H1, DYNC1LI1, DYNC1LI2, DYNLRB1, ECPAS, EEF1A2, EIF3L, FARSB, HNRNPK, INPP5A, ITPR1, IVD, JUP, KRT9, NCKAP1, NCL, PABPC1, PIK3C3, PRKDC, PSMD13, PTPRZ1, RBMX, RPLP0, RPLP2, SEPTIN6, SPTAN1, SPTBN2, SYNE1, SYT1, XRCC5
GO Component	GO:0030669	Clathrin-coated endocytic vesicle membrane	3	70	1.15	0.43	0.041	AP2A1, AP2B1, SYT1

Table S5: Cerebellar vermis pathways enriched in downregulated proteins (FDR-adjusted $p < 0.05$) of children with idiopathic autism *(continued)*

Category	Term ID	Term description	Gene count	Back-ground	Strength	Signal	FDR	Matching proteins in network
GO Component	GO:0000776	Kinetochore	4	165	0.90	0.38	0.049	DYNC1I1, DYNC1LI1, DYNC1LI2, SEPTIN6
STRING clusters	CL:1883	Chaperonin TCP-1, conserved site, and Cellular proteostasis	8	13	2.31	3.66	4.0e-12	CCT2, CCT3, CCT4, CCT5, CCT6A, CCT7, CCT8, TCP1
STRING clusters	CL:1885	Chaperonin TCP-1, conserved site	6	6	2.52	3.05	6.3e-10	CCT2, CCT4, CCT5, CCT6A, CCT7, CCT8
STRING clusters	CL:26297	Cytoplasmic dynein complex, and Dynactin complex	6	36	1.74	1.77	1.7e-06	CAPZA2, DYNC1H1, DYNC1I1, DYNC1LI1, DYNC1LI2, DYNLRB1
STRING clusters	CL:26300	Cytoplasmic dynein complex	5	18	1.96	1.74	3.6e-06	DYNC1H1, DYNC1I1, DYNC1LI1, DYNC1LI2, DYNLRB1
STRING clusters	CL:2333	mRNA processing, and CRD-mediated mRNA stability complex	5	55	1.47	1.02	4.4e-04	HNRNPD, HNRNPK, PSPC1, RBMX, SFPQ
STRING clusters	CL:2342	Mixed, incl. Multisystem proteinopathy, and Zinc finger, CHHC-type	3	16	1.79	0.71	6.7e-03	HNRNPD, HNRNPK, RBMX
STRING clusters	CL:13780	Nef Mediated CD8 Down-regulation	2	5	2.12	0.47	0.041	AP2A1, AP2B1
STRING clusters	CL:2432	NOPS, and Amyotrophic lateral sclerosis type 21	2	5	2.12	0.47	0.041	PSPC1, SFPQ
STRING clusters	CL:26316	Dynein light chain, type 1/2, conserved site, and Omphalocele	2	5	2.12	0.47	0.041	DYNC1LI2, DYNLRB1
STRING clusters	CL:3211	tRNA-splicing ligase complex	2	5	2.12	0.47	0.041	DDX1, RTRAF
STRING clusters	CL:6250	Nonhomologous end joining complex	2	5	2.12	0.47	0.041	PRKDC, XRCC5

Table S5: Cerebellar vermis pathways enriched in downregulated proteins (FDR-adjusted $p < 0.05$) of children with idiopathic autism *(continued)*

Category	Term ID	Term description	Gene count	Back-ground	Strength	Signal	FDR	Matching proteins in network
KEGG	hsa05132	Salmonella infection	7	209	1.04	0.76	1.3e-03	DYNC1H1, DYNC1I1, DYNC1LI1, DYNC1LI2, DYNLRB1, NCKAP1, PIK3C3
KEGG	hsa04962	Vasopressin-regulated water reabsorption	4	43	1.48	0.83	2.1e-03	DYNC1H1, DYNC1I1, DYNC1LI1, DYNC1LI2
KEGG	hsa04145	Phagosome	5	141	1.07	0.58	9.1e-03	DYNC1H1, DYNC1I1, DYNC1LI1, DYNC1LI2, PIK3C3
KEGG	hsa05131	Shigellosis	5	218	0.88	0.38	0.049	DOCK1, ITPR1, PIK3C3, SEPTIN3, SEPTIN6
Reactome	HSA-390450	Folding of actin by CCT/TriC	8	10	2.42	4.01	4.3e-13	CCT2, CCT3, CCT4, CCT5, CCT6A, CCT7, CCT8, TCP1
Reactome	HSA-389960	Formation of tubulin folding intermediates by CCT/TriC	8	25	2.02	3.14	6.7e-11	CCT2, CCT3, CCT4, CCT5, CCT6A, CCT7, CCT8, TCP1
Reactome	HSA-389957	Prefoldin mediated transfer of substrate to CCT/TriC	8	27	1.99	3.10	7.5e-11	CCT2, CCT3, CCT4, CCT5, CCT6A, CCT7, CCT8, TCP1
Reactome	HSA-6814122	Cooperation of PDCL (PhLP1) and TRiC/CCT in G-protein beta folding	8	38	1.84	2.77	4.8e-10	CCT2, CCT3, CCT4, CCT5, CCT6A, CCT7, CCT8, TCP1
Reactome	HSA-390471	Association of TriC/CCT with target proteins during biosynthesis	8	39	1.83	2.76	4.9e-10	CCT2, CCT3, CCT4, CCT5, CCT6A, CCT7, CCT8, TCP1

Table S5: Cerebellar vermis pathways enriched in downregulated proteins (FDR-adjusted $p < 0.05$) of children with idiopathic autism *(continued)*

Category	Term ID	Term description	Gene count	Back-ground	Strength	Signal	FDR	Matching proteins in network
Reactome	HSA-392499	Metabolism of proteins	26	1917	0.65	0.92	4.5e-09	ANK1, CAPZA2, CCT2, CCT3, CCT4, CCT5, CCT6A, CCT7, CCT8, DDX17, DYNC1H1, DYNC1I1, DYNC1LI1, DYNC1LI2, EEF1A2, EIF3L, FARSB, HNRNPK, PABPC1, PRKDC, PSMD13, RPLP0, RPLP2, SPTAN1, SPTBN2, TCP1
Reactome	HSA-5620922	BBSome-mediated cargo-targeting to cilium	6	22	1.95	2.26	6.2e-08	CCT2, CCT3, CCT4, CCT5, CCT8, TCP1
Reactome	HSA-5663205	Infectious disease	17	917	0.78	1.02	2.8e-07	AP2A1, AP2B1, DOCK1, DYNC1H1, DYNC1I1, DYNC1LI1, DYNC1LI2, HNRNPK, ITPR1, NCKAP1, PIK3C3, PSMD13, RPLP0, RPLP2, SFPQ, SYT1, XRCC5
Reactome	HSA-6807878	COPI-mediated anterograde transport	8	101	1.42	1.75	2.8e-07	ANK1, CAPZA2, DYNC1H1, DYNC1I1, DYNC1LI1, DYNC1LI2, SPTAN1, SPTBN2
Reactome	HSA-2132295	MHC class II antigen presentation	8	122	1.33	1.57	9.5e-07	AP2A1, AP2B1, CAPZA2, DYNC1H1, DYNC1I1, DYNC1LI1, DYNC1LI2, SPTBN2
Reactome	HSA-194315	Signaling by Rho GTPases	14	672	0.84	1.01	2.1e-06	ARHGAP26, CCT2, CCT6A, CCT7, DOCK1, DYNC1H1, DYNC1I1, DYNC1LI1, DYNC1LI2, JUP, NCKAP1, PIK3C3, RBMX, SPTAN1

Table S5: Cerebellar vermis pathways enriched in downregulated proteins (FDR-adjusted p < 0.05) of children with idiopathic autism *(continued)*

Category	Term ID	Term description	Gene count	Back-ground	Strength	Signal	FDR	Matching proteins in network
Reactome	HSA-168256	Immune System	22	1979	0.56	0.69	4.7e-06	AP2A1, AP2B1, CAPZA2, CCT2, CCT8, DOCK1, DYNC1H1, DYNC1I1, DYNC1LI1, DYNC1LI2, ITPR1, JUP, NCKAP1, PIK3C3, PRKDC, PSMD13, PTPRZ1, RPLP0, SPTAN1, SPTBN2, TCP1, XRCC5
Reactome	HSA-5617833	Cilium Assembly	8	200	1.12	1.13	2.4e-05	CCT2, CCT3, CCT4, CCT5, CCT8, DYNC1H1, DYNLRB1, TCP1
Reactome	HSA-6811436	COPI-independent Golgi-to-ER retrograde traffic	5	52	1.50	1.21	8.8e-05	CAPZA2, DYNC1H1, DYNC1I1, DYNC1LI1, DYNC1LI2
Reactome	HSA-3371497	HSP90 chaperone cycle for steroid hormone receptors (SHR) in the presence of ligand	5	55	1.47	1.18	1.1e-04	CAPZA2, DYNC1H1, DYNC1I1, DYNC1LI1, DYNC1LI2
Reactome	HSA-9013418	RHOBTB2 GTPase cycle	4	23	1.76	1.23	1.3e-04	CCT2, CCT6A, CCT7, RBMX
Reactome	HSA-168249	Innate Immune System	14	1041	0.64	0.65	2.3e-04	CAPZA2, CCT2, CCT8, DOCK1, DYNC1H1, DYNC1LI1, ITPR1, JUP, NCKAP1, PIK3C3, PRKDC, PSMD13, SPTAN1, XRCC5
Reactome	HSA-199991	Membrane Trafficking	11	626	0.76	0.72	2.7e-04	ANK1, AP2A1, AP2B1, CAPZA2, DYNC1H1, DYNC1I1, DYNC1LI1, DYNC1LI2, SPTAN1, SPTBN2, SYT1
Reactome	HSA-422475	Axon guidance	10	551	0.78	0.70	5.0e-04	ANK1, AP2A1, AP2B1, DOCK1, PABPC1, PSMD13, RPLP0, RPLP2, SPTAN1, SPTBN2

Table S5: Cerebellar vermis pathways enriched in downregulated proteins (FDR-adjusted p < 0.05) of children with idiopathic autism *(continued)*

Category	Term ID	Term description	Gene count	Back-ground	Strength	Signal	FDR	Matching proteins in network
Reactome	HSA-9012999	RHO GTPase cycle	9	449	0.82	0.71	6.6e-04	ARHGAP26, CCT2, CCT6A, CCT7, DOCK1, JUP, NCKAP1, RBMX, SPTAN1
Reactome	HSA-162582	Signal Transduction	21	2540	0.43	0.47	7.2e-04	AP2A1, AP2B1, ARHGAP26, CCT2, CCT6A, CCT7, DOCK1, DYNC1H1, DYNC1I1, DYNC1LI1, DYNC1LI2, ITPR1, JUP, NCKAP1, PIK3C3, PSMD13, PTPRZ1, RBMX, SFPQ, SPTAN1, SPTBN2
Reactome	HSA-9646399	Aggrephagy	4	42	1.50	0.96	7.4e-04	DYNC1H1, DYNC1I1, DYNC1LI1, DYNC1LI2
Reactome	HSA-1280218	Adaptive Immune System	11	758	0.68	0.60	1.1e-03	AP2A1, AP2B1, CAPZA2, DYNC1H1, DYNC1I1, DYNC1LI1, DYNC1LI2, ITPR1, PIK3C3, PSMD13, SPTBN2
Reactome	HSA-373760	L1CAM interactions	5	120	1.14	0.74	2.4e-03	ANK1, AP2A1, AP2B1, SPTAN1, SPTBN2
Reactome	HSA-9609646	HCMV Infection	5	126	1.11	0.71	2.9e-03	DYNC1H1, DYNC1I1, DYNC1LI1, DYNC1LI2, HNRNPK
Reactome	HSA-8953854	Metabolism of RNA	10	705	0.67	0.54	3.1e-03	DDX1, HNRNPD, HNRNPK, NCL, PABPC1, PSMD13, RBMX, RPLP0, RPLP2, RTRAF
Reactome	HSA-1632852	Macroautophagy	5	133	1.09	0.69	3.5e-03	DYNC1H1, DYNC1I1, DYNC1LI1, DYNC1LI2, PIK3C3
Reactome	HSA-9013422	RHOBTB1 GTPase cycle	3	23	1.63	0.78	3.6e-03	CCT2, CCT7, RBMX
Reactome	HSA-6798695	Neutrophil degranulation	8	476	0.74	0.53	5.4e-03	CCT2, CCT8, DYNC1H1, DYNC1LI1, JUP, PSMD13, SPTAN1, XRCC5

Table S5: Cerebellar vermis pathways enriched in downregulated proteins (FDR-adjusted $p < 0.05$) of children with idiopathic autism *(continued)*

Category	Term ID	Term description	Gene count	Back-ground	Strength	Signal	FDR	Matching proteins in network
Reactome	HSA-1266738	Developmental Biology	12	1108	0.55	0.45	5.9e-03	ANK1, AP2A1, AP2B1, DOCK1, JUP, KRT9, PABPC1, PSMD13, RPLP0, RPLP2, SPTAN1, SPTBN2
Reactome	HSA-445095	Interaction between L1 and Ankyrins	3	31	1.50	0.68	7.2e-03	ANK1, SPTAN1, SPTBN2
Reactome	HSA-9006934	Signaling by Receptor Tyrosine Kinases	8	521	0.70	0.48	9.0e-03	AP2A1, AP2B1, DOCK1, ITPR1, JUP, NCKAP1, PIK3C3, PTPRZ1
Reactome	HSA-141444	Amplification of signal from unattached kinetochores via a MAD2 inhibitory signal	4	94	1.15	0.58	0.010	DYNC1H1, DYNC1I1, DYNC1LI1, DYNC1LI2
Reactome	HSA-4420097	VEGFA-VEGFR2 Pathway	4	96	1.14	0.58	0.011	DOCK1, ITPR1, JUP, NCKAP1
Reactome	HSA-182218	Nef Mediated CD8 Down-regulation	2	6	2.04	0.66	0.011	AP2A1, AP2B1
Reactome	HSA-72766	Translation	6	290	0.83	0.50	0.012	EEF1A2, EIF3L, FARSB, PABPC1, RPLP0, RPLP2
Reactome	HSA-195258	RHO GTPase Effectors	6	292	0.83	0.50	0.012	DYNC1H1, DYNC1I1, DYNC1LI1, DYNC1LI2, NCKAP1, PIK3C3
Reactome	HSA-2467813	Separation of Sister Chromatids	5	189	0.94	0.52	0.013	DYNC1H1, DYNC1I1, DYNC1LI1, DYNC1LI2, PSMD13
Reactome	HSA-9609690	HCMV Early Events	4	102	1.11	0.56	0.013	DYNC1H1, DYNC1I1, DYNC1LI1, DYNC1LI2
Reactome	HSA-156827	L13a-mediated translational silencing of Ceruloplasmin expression	4	110	1.08	0.53	0.016	EIF3L, PABPC1, RPLP0, RPLP2

Table S5: Cerebellar vermis pathways enriched in downregulated proteins (FDR-adjusted $p < 0.05$) of children with idiopathic autism *(continued)*

Category	Term ID	Term description	Gene count	Back-ground	Strength	Signal	FDR	Matching proteins in network
Reactome	HSA-2262752	Cellular responses to stress	9	747	0.60	0.41	0.016	ACADVL, CAPZA2, DYNC1H1, DYNC1I1, DYNC1LI1, DYNC1LI2, PSMD13, RPLP0, RPLP2
Reactome	HSA-167590	Nef Mediated CD4 Down-regulation	2	9	1.86	0.58	0.018	AP2A1, AP2B1
Reactome	HSA-9648025	EML4 and NUDC in mitotic spindle formation	4	116	1.05	0.51	0.018	DYNC1H1, DYNC1I1, DYNC1LI1, DYNC1LI2
Reactome	HSA-72737	Cap-dependent Translation Initiation	4	118	1.05	0.51	0.018	EIF3L, PABPC1, RPLP0, RPLP2
Reactome	HSA-2500257	Resolution of Sister Chromatid Cohesion	4	125	1.02	0.48	0.022	DYNC1H1, DYNC1I1, DYNC1LI1, DYNC1LI2
Reactome	HSA-450408	AUF1 (hnRNP D0) binds and destabilizes mRNA	3	54	1.26	0.51	0.023	HNRNPD, PABPC1, PSMD13
Reactome	HSA-3270619	IRF3-mediated induction of type I IFN	2	13	1.70	0.50	0.030	PRKDC, XRCC5
Reactome	HSA-5140745	WNT5A-dependent internalization of FZD2, FZD5 and ROR2	2	13	1.70	0.50	0.030	AP2A1, AP2B1
Reactome	HSA-5663220	RHO GTPases Activate Formins	4	139	0.98	0.44	0.030	DYNC1H1, DYNC1I1, DYNC1LI1, DYNC1LI2
Reactome	HSA-597592	Post-translational protein modification	12	1405	0.45	0.32	0.030	ANK1, CAPZA2, DDX17, DYNC1H1, DYNC1I1, DYNC1LI1, DYNC1LI2, HNRNPK, PRKDC, PSMD13, SPTAN1, SPTBN2
Reactome	HSA-3858494	Beta-catenin independent WNT signaling	4	143	0.96	0.43	0.032	AP2A1, AP2B1, ITPR1, PSMD13

Table S5: Cerebellar vermis pathways enriched in downregulated proteins (FDR-adjusted p < 0.05) of children with idiopathic autism *(continued)*

Category	Term ID	Term description	Gene count	Back-ground	Strength	Signal	FDR	Matching proteins in network
Reactome	HSA-177504	Retrograde neurotrophin signalling	2	14	1.67	0.49	0.032	AP2A1, AP2B1
Reactome	HSA-5099900	WNT5A-dependent internalization of FZD4	2	15	1.64	0.48	0.035	AP2A1, AP2B1
Reactome	HSA-8866427	VLDLR internalisation and degradation	2	16	1.61	0.46	0.039	AP2A1, AP2B1
Reactome	HSA-416993	Trafficking of GluR2-containing AMPA receptors	2	17	1.59	0.45	0.043	AP2A1, AP2B1
Reactome	HSA-69620	Cell Cycle Checkpoints	5	272	0.78	0.37	0.043	DYNC1H1, DYNC1I1, DYNC1LI1, DYNC1LI2, PSMD13
WikiPathways	WP4949	16p11.2 proximal deletion syndrome	8	74	1.55	1.96	1.0e-07	CCT2, CCT3, CCT4, CCT5, CCT6A, CCT7, CCT8, TCP1
WikiPathways	WP4532	Intraflagellar transport proteins binding to dynein	5	27	1.78	1.50	1.6e-05	DYNC1H1, DYNC1I1, DYNC1LI1, DYNC1LI2, DYNLRB1
WikiPathways	WP5085	Vasopressin-regulated water reabsorption	4	43	1.48	0.78	3.2e-03	DYNC1H1, DYNC1I1, DYNC1LI1, DYNC1LI2
WikiPathways	WP411	mRNA processing	5	125	1.12	0.59	9.1e-03	DDX1, HNRNPD, HNRNPK, RBMX, SFPQ
TISSUES	BTO:0000345	Digestive gland	39	2881	0.65	1.07	2.6e-15	ACADVL, ACAT1, ANK1, AP2B1, API5, CAPZA2, CCT2, CCT3, CCT4, CCT5, CCT6A, CCT7, CCT8, CS, DDX1, DDX17, DYNC1H1, DYNC1LI2, DYNLRB1, EIF3L, HNRNPD, HNRNPK, ITPR1, IVD, JUP, NCL, PABPC1, PRKDC, PSMD13, PSPC1, RBMX, RPLP0, RPLP2, RTRAF, SFPQ, SPTAN1, SPTBN2, TCP1, XRCC5

Table S5: Cerebellar vermis pathways enriched in downregulated proteins (FDR-adjusted $p < 0.05$) of children with idiopathic autism *(continued)*

Category	Term ID	Term description	Gene count	Back-ground	Strength	Signal	FDR	Matching proteins in network
TISSUES	BTO:0000772	Lymphoblast	17	240	1.37	2.93	2.6e-15	CCT2, CCT3, CCT4, CCT5, CCT7, CCT8, DDX1, EIF3L, HNRNPD, HNRNPK, NCL, PABPC1, PSMD13, RPLP0, RPLP2, SFPQ, TCP1
TISSUES	BTO:0000142	Brain	50	5733	0.46	0.75	3.2e-15	ACADVL, ANK1, AP2B1, API5, ARHGAP26, CAPZA2, CCT2, CCT3, CCT4, CCT5, CCT6A, CCT7, CCT8, CS, DDX1, DDX17, DIP2B, DYNC1H1, DYNC1I1, DYNC1LI1, DYNC1LI2, ECPAS, EEF1A2, EIF3L, FARSB, HNRNPK, INPP5A, ITPR1, IVD, NCKAP1, NCL, NOVA1, PABPC1, PRKDC, PSMD13, PTPRZ1, RBMX, RPLP0, RPLP2, RTRAF, SEPTIN3, SEPTIN6, SFPQ, SPTAN1, SPTBN2, SYNE1, SYT1, TCP1, UBASH3B, XRCC5
TISSUES	BTO:0000775	Lymphocyte	23	698	1.03	1.99	3.2e-15	CCT2, CCT3, CCT4, CCT5, CCT7, CCT8, DDX1, DYNC1H1, DYNC1LI1, EIF3L, HNRNPD, HNRNPK, ITPR1, KRT9, NCL, PABPC1, PSMD13, RPLP0, RPLP2, SFPQ, SPTAN1, TCP1, UBASH3B

Table S5: Cerebellar vermis pathways enriched in downregulated proteins (FDR-adjusted p < 0.05) of children with idiopathic autism *(continued)*

Category	Term ID	Term description	Gene count	Back-ground	Strength	Signal	FDR	Matching proteins in network
TISSUES	BTO:0000759	Liver	33	2125	0.71	1.17	2.0e-14	ACADVL, ACAT1, ANK1, CAPZA2, CCT2, CCT3, CCT4, CCT5, CCT6A, CCT7, CCT8, CS, DDX1, DDX17, DYNC1H1, EIF3L, HNRNPD, HNRNPK, ITPR1, IVD, JUP, NCL, PRKDC, PSMD13, PSPC1, RBMX, RPLP0, RPLP2, SFPQ, SPTAN1, SPTBN2, TCP1, XRCC5
TISSUES	BTO:0000089	Blood	31	1824	0.75	1.25	2.1e-14	ACADVL, ANK1, CAPZA2, CCT2, CCT3, CCT4, CCT5, CCT6A, CCT7, CCT8, DDX1, DYNC1H1, DYNC1LI1, DYNLRB1, EIF3L, HNRNPD, HNRNPK, ITPR1, IVD, JUP, KRT9, NCL, PABPC1, PSMD13, RPLP0, RPLP2, SEPTIN6, SFPQ, SPTAN1, TCP1, UBASH3B
TISSUES	BTO:0000574	Hematopoietic cell	25	1019	0.91	1.62	2.1e-14	ANK1, CCT2, CCT3, CCT4, CCT5, CCT7, CCT8, DDX1, DYNC1H1, DYNC1LI1, EIF3L, HNRNPD, HNRNPK, ITPR1, JUP, KRT9, NCL, PABPC1, PSMD13, RPLP0, RPLP2, SFPQ, SPTAN1, TCP1, UBASH3B

Table S5: Cerebellar vermis pathways enriched in downregulated proteins (FDR-adjusted $p < 0.05$) of children with idiopathic autism *(continued)*

Category	Term ID	Term description	Gene count	Back-ground	Strength	Signal	FDR	Matching proteins in network
TISSUES	BTO:0000751	Leukocyte	24	924	0.93	1.67	2.5e-14	CCT2, CCT3, CCT4, CCT5, CCT7, CCT8, DDX1, DYNC1H1, DYNC1LI1, EIF3L, HNRNPD, HNRNPK, ITPR1, JUP, KRT9, NCL, PABPC1, PSMD13, RPLP0, RPLP2, SFPQ, SPTAN1, TCP1, UBASH3B
TISSUES	BTO:0000570	Hematopoietic system	35	2755	0.62	0.98	4.0e-13	ACADVL, ANK1, API5, CAPZA2, CCT2, CCT3, CCT4, CCT5, CCT6A, CCT7, CCT8, CS, DDX1, DYNC1H1, DYNC1LI1, DYNLRB1, EIF3L, HNRNPD, HNRNPK, ITPR1, IVD, JUP, KRT9, NCL, PABPC1, PSMD13, RBMX, RPLP0, RPLP2, SEPTIN6, SFPQ, SPTAN1, SYNE1, TCP1, UBASH3B
TISSUES	BTO:0000753	Lymphoid tissue	27	1600	0.74	1.18	4.5e-12	CCT2, CCT3, CCT4, CCT5, CCT7, CCT8, CS, DDX1, DYNC1H1, DYNC1LI1, EIF3L, HNRNPD, HNRNPK, ITPR1, KRT9, NCL, PABPC1, PSMD13, RBMX, RPLP0, RPLP2, SEPTIN6, SFPQ, SPTAN1, SYNE1, TCP1, UBASH3B

Table S5: Cerebellar vermis pathways enriched in downregulated proteins (FDR-adjusted p < 0.05) of children with idiopathic autism *(continued)*

Category	Term ID	Term description	Gene count	Back-ground	Strength	Signal	FDR	Matching proteins in network
TISSUES	BTO:0001491	Viscus	43	5378	0.42	0.65	1.9e-10	ACADVL, ACAT1, ANK1, AP2A1, AP2B1, API5, ARHGAP26, CAPZA2, CCT2, CCT3, CCT4, CCT5, CCT6A, CCT7, CCT8, CS, DDX1, DDX17, DYNC1H1, DYNC1LI2, DYNLRB1, EEF1A2, EIF3L, HNRNPD, HNRNPK, ITPR1, IVD, JUP, NCL, PABPC1, PRKDC, PSMD13, PSPC1, RBMX, RPLP0, RPLP2, SFPQ, SPTAN1, SPTBN2, SYNE1, TCP1, UBASH3B, XRCC5
TISSUES	BTO:0001488	Endocrine gland	45	6403	0.36	0.57	2.9e-09	ACADVL, ACAT1, ANK1, AP2A1, AP2B1, API5, CAPZA2, CCT2, CCT3, CCT4, CCT5, CCT6A, CCT7, CCT8, CS, DDX1, DDX17, DYNC1H1, DYNC1LI1, DYNC1LI2, DYNLRB1, EIF3L, FARSB, HNRNPD, HNRNPK, ITPR1, IVD, JUP, NCL, PABPC1, PIK3C3, PRKDC, PSMD13, PSPC1, RBMX, RPLP0, RPLP2, RTRAF, SEPTIN6, SFPQ, SPTAN1, SPTBN2, SYNE1, TCP1, XRCC5

Table S5: Cerebellar vermis pathways enriched in downregulated proteins (FDR-adjusted $p < 0.05$) of children with idiopathic autism *(continued)*

Category	Term ID	Term description	Gene count	Back-ground	Strength	Signal	FDR	Matching proteins in network
TISSUES	BTO:0000080	Male reproductive gland	29	2658	0.55	0.78	1.3e-08	ACADVL, AP2B1, API5, CCT2, CCT3, CCT4, CCT5, CCT6A, CCT7, CCT8, CS, DDX17, DYNC1H1, DYNC1LI2, DYNLRB1, EIF3L, FARSB, HNRNPK, IVD, NCL, PABPC1, PIK3C3, RBMX, RPLP0, RPLP2, SEPTIN6, SYNE1, TCP1, XRCC5
TISSUES	BTO:0000522	Gland	46	7004	0.33	0.53	1.3e-08	ACADVL, ACAT1, ANK1, AP2A1, AP2B1, API5, CAPZA2, CCT2, CCT3, CCT4, CCT5, CCT6A, CCT7, CCT8, CS, DDX1, DDX17, DYNC1H1, DYNC1LI1, DYNC1LI2, DYNLRB1, EIF3L, FARSB, HNRNPD, HNRNPK, ITPR1, IVD, JUP, NCL, PABPC1, PIK3C3, PRKDC, PSMD13, PSPC1, RBMX, RPLP0, RPLP2, RTRAF, SEPTIN6, SFPQ, SPTAN1, SPTBN2, SYNE1, TCP1, UBASH3B, XRCC5

Table S5: Cerebellar vermis pathways enriched in downregulated proteins (FDR-adjusted $p < 0.05$) of children with idiopathic autism *(continued)*

Category	Term ID	Term description	Gene count	Back-ground	Strength	Signal	FDR	Matching proteins in network
TISSUES	BTO:0000083	Female reproductive system	43	6111	0.36	0.56	1.5e-08	ACADVL, AP2A1, AP2B1, API5, ARHGAP26, CAPZA2, CCT2, CCT3, CCT4, CCT5, CCT6A, CCT7, CCT8, CS, DDX1, DDX17, DOCK1, DYNC1H1, DYNC1LI2, DYNLRB1, EIF3L, FARSB, HNRNPD, HNRNPK, ITPR1, IVD, JUP, NCKAP1, NCL, PABPC1, PIK3C3, PRKDC, PSMD13, PSPC1, RBMX, RPLP0, RPLP2, SEPTIN6, SPTAN1, SYNE1, TCP1, UBASH3B, XRCC5
TISSUES	BTO:0000203	Respiratory system	23	1707	0.65	0.88	4.6e-08	ACADVL, API5, CCT3, CCT5, CCT7, DDX1, DDX17, DYNC1H1, EEF1A2, EIF3L, HNRNPD, HNRNPK, JUP, NCKAP1, NCL, PABPC1, PRKDC, PSMD13, RBMX, RPLP0, RPLP2, SPTAN1, XRCC5

Table S5: Cerebellar vermis pathways enriched in downregulated proteins (FDR-adjusted $p < 0.05$) of children with idiopathic autism *(continued)*

Category	Term ID	Term description	Gene count	Back-ground	Strength	Signal	FDR	Matching proteins in network
TISSUES	BTO:0001489	Whole body	59	13099	0.17	0.38	8.0e-08	ACADVL, ACAT1, ANK1, AP2A1, AP2B1, API5, ARHGAP26, CAPZA2, CCT2, CCT3, CCT4, CCT5, CCT6A, CCT7, CCT8, CS, DDX1, DDX17, DIP2B, DOCK1, DYNC1H1, DYNC1I1, DYNC1LI1, DYNC1LI2, DYNLRB1, ECPAS, EEF1A2, EIF3L, FARSB, HNRNPD, HNRNPK, INPP5A, ITPR1, IVD, JUP, KRT9, NCKAP1, NCL, NOVA1, PABPC1, PIK3C3, PRKDC, PSMD13, PSPC1, PTPRZ1, RBMX, RPLP0, RPLP2, RTRAF, SEPTIN3, SEPTIN6, SFPQ, SPTAN1, SPTBN2, SYNE1, SYT1, TCP1, UBASH3B, XRCC5
TISSUES	BTO:0001271	Leukemia cell	17	1067	0.72	0.89	1.3e-06	ANK1, CCT2, CCT3, CCT4, CCT5, DDX1, DDX17, HNRNPD, HNRNPK, NCL, PIK3C3, PRKDC, RPLP0, RPLP2, SFPQ, SPTAN1, XRCC5
TISSUES	BTO:0000580	Blood cancer cell	18	1234	0.68	0.84	1.6e-06	ANK1, CCT2, CCT3, CCT4, CCT5, CCT8, DDX1, DDX17, HNRNPD, HNRNPK, NCL, PIK3C3, PRKDC, RPLP0, RPLP2, SFPQ, SPTAN1, XRCC5

Table S5: Cerebellar vermis pathways enriched in downregulated proteins (FDR-adjusted p < 0.05) of children with idiopathic autism *(continued)*

Category	Term ID	Term description	Gene count	Back-ground	Strength	Signal	FDR	Matching proteins in network
TISSUES	BTO:0000763	Lung	19	1395	0.65	0.81	1.7e-06	ACADVL, CCT3, CCT5, CCT7, DDX1, DDX17, DYNC1H1, EEF1A2, EIF3L, HNRNPD, JUP, NCL, PABPC1, PRKDC, PSMD13, RBMX, RPLP0, RPLP2, SPTAN1
TISSUES	BTO:0000534	Gonad	22	1923	0.57	0.73	1.9e-06	AP2B1, API5, CCT2, CCT3, CCT4, CCT5, CCT7, CCT8, CS, DYNC1H1, DYNC1LI2, EIF3L, FARSB, HNRNPK, PABPC1, PIK3C3, RBMX, RPLP0, SEPTIN6, SYNE1, TCP1, XRCC5
TISSUES	BTO:0003099	Internal female genital organ	26	2804	0.48	0.62	4.0e-06	ACADVL, AP2A1, CCT3, CCT5, DDX1, DDX17, DOCK1, DYNC1H1, DYNC1LI2, DYNLRB1, EIF3L, HNRNPD, ITPR1, JUP, NCKAP1, NCL, PIK3C3, PRKDC, PSPC1, RBMX, RPLP0, RPLP2, SEPTIN6, SPTAN1, SYNE1, XRCC5
TISSUES	BTO:0003914	Interstitial cell of Cajal	8	193	1.13	1.19	1.3e-05	CAPZA2, CCT2, CCT3, CCT5, CCT6A, CCT7, HNRNPK, TCP1
TISSUES	BTO:0000254	Female reproductive gland	26	3017	0.45	0.57	1.7e-05	AP2B1, API5, CCT2, CCT3, CCT4, CCT5, CCT7, CCT8, CS, DYNC1H1, DYNC1LI2, DYNLRB1, EIF3L, FARSB, HNRNPK, IVD, NCL, PABPC1, PIK3C3, RBMX, RPLP0, RPLP2, SEPTIN6, SYNE1, TCP1, XRCC5

Table S5: Cerebellar vermis pathways enriched in downregulated proteins (FDR-adjusted $p < 0.05$) of children with idiopathic autism *(continued)*

Category	Term ID	Term description	Gene count	Back-ground	Strength	Signal	FDR	Matching proteins in network
TISSUES	BTO:0000058	Alimentary canal	21	2021	0.53	0.63	1.9e-05	ACADVL, CAPZA2, CCT2, CCT3, CCT5, CCT6A, CCT7, CCT8, DDX17, DYNC1H1, EEF1A2, EIF3L, HNRNPK, NCL, PABPC1, RBMX, RPLP0, RPLP2, SPTAN1, TCP1, XRCC5
TISSUES	BTO:0001363	Testis	20	1868	0.55	0.64	2.5e-05	AP2B1, CCT2, CCT3, CCT4, CCT5, CCT7, CCT8, DYNC1H1, DYNC1LI2, EIF3L, FARSB, HNRNPK, PABPC1, PIK3C3, RBMX, RPLP0, SEPTIN6, SYNE1, TCP1, XRCC5
TISSUES	BTO:0000088	Cardiovascular system	15	1057	0.67	0.74	3.4e-05	ACADVL, ACAT1, ARHGAP26, CCT7, CS, DYNC1H1, DYNC1LI1, EIF3L, IVD, RBMX, RPLP0, SPTAN1, SPTBN2, SYNE1, XRCC5
TISSUES	BTO:0003096	Internal male genital organ	22	2388	0.48	0.56	6.4e-05	AP2B1, CCT2, CCT3, CCT4, CCT5, CCT7, CCT8, CS, DYNC1H1, DYNC1LI2, EIF3L, FARSB, HNRNPK, PABPC1, PIK3C3, RBMX, RPLP0, RPLP2, SEPTIN6, SYNE1, TCP1, XRCC5
TISSUES	BTO:0000511	Gastrointestinal tract	19	1816	0.54	0.60	7.0e-05	ACADVL, CAPZA2, CCT2, CCT3, CCT5, CCT6A, CCT7, CCT8, DDX17, DYNC1H1, EEF1A2, EIF3L, HNRNPK, NCL, PABPC1, RBMX, RPLP0, SPTAN1, TCP1

Table S5: Cerebellar vermis pathways enriched in downregulated proteins (FDR-adjusted $p < 0.05$) of children with idiopathic autism *(continued)*

Category	Term ID	Term description	Gene count	Back-ground	Strength	Signal	FDR	Matching proteins in network
TISSUES	BTO:0001374	Thymus	8	271	0.99	0.92	1.2e-04	CCT4, CS, EIF3L, HNRNPD, HNRNPK, RBMX, RPLP2, SEPTIN6
TISSUES	BTO:0000562	Heart	12	738	0.73	0.73	1.3e-04	ACADVL, ACAT1, ARHGAP26, CCT7, CS, DYNC1H1, EIF3L, RPLP0, SPTAN1, SPTBN2, SYNE1, XRCC5
TISSUES	BTO:0000586	Colonic cancer cell	8	280	0.97	0.90	1.5e-04	DDX17, EEF1A2, HNRNPD, HNRNPK, NCL, PABPC1, PSPC1, SFPQ
TISSUES	BTO:0000421	Connective tissue	13	954	0.65	0.64	2.8e-04	ACAT1, CAPZA2, CCT2, CCT3, CCT5, CCT6A, CCT7, CS, EIF3L, HNRNPK, PABPC1, TCP1, XRCC5
TISSUES	BTO:0000131	Blood plasma	9	424	0.84	0.77	3.1e-04	ANK1, CAPZA2, CCT2, CCT4, CCT6A, IVD, SEPTIN6, TCP1, UBASH3B
TISSUES	BTO:0000671	Kidney	13	1039	0.61	0.58	6.6e-04	CAPZA2, CCT3, DYNC1H1, DYNLRB1, EIF3L, HNRNPK, PSMD13, PSPC1, RBMX, RPLP0, RPLP2, SYNE1, UBASH3B
TISSUES	BTO:0000132	Blood platelet	8	363	0.86	0.72	7.9e-04	ANK1, CAPZA2, CCT2, CCT4, CCT6A, SEPTIN6, TCP1, UBASH3B
TISSUES	BTO:0001424	Uterus	13	1117	0.58	0.54	1.3e-03	AP2A1, CCT5, DDX1, DDX17, DYNC1LI2, EIF3L, ITPR1, NCKAP1, PIK3C3, RBMX, RPLP0, SPTAN1, XRCC5

Table S5: Cerebellar vermis pathways enriched in downregulated proteins (FDR-adjusted $p < 0.05$) of children with idiopathic autism *(continued)*

Category	Term ID	Term description	Gene count	Back-ground	Strength	Signal	FDR	Matching proteins in network
TISSUES	BTO:0000174	Embryonic structure	19	2369	0.42	0.42	2.5e-03	ACADVL, CCT3, CCT5, CCT7, DDX17, DOCK1, DYNC1H1, DYNC1LI2, DYNLRB1, EIF3L, HNRNPD, JUP, PRKDC, PSMD13, PSPC1, RPLP0, SEPTIN6, SPTAN1, SYNE1
TISSUES	BTO:0001023	Ovary cancer cell	4	84	1.19	0.64	6.7e-03	API5, HNRNPD, PABPC1, SFPQ
TISSUES	BTO:0000232	Cerebellum	8	519	0.70	0.49	8.1e-03	ACADVL, CCT3, CCT7, DYNC1LI1, NOVA1, RPLP2, RTRAF, SYNE1
TISSUES	BTO:0000146	Brain stem	9	675	0.64	0.46	8.9e-03	ACADVL, CCT3, CCT7, DYNC1LI1, NOVA1, PTPRZ1, RPLP2, RTRAF, SYNE1
TISSUES	BTO:0000255	Brain cell line	5	188	0.94	0.52	0.012	CCT2, HNRNPK, RPLP0, SFPQ, SPTAN1
TISSUES	BTO:0000887	Muscle	11	1070	0.53	0.39	0.014	ANK1, AP2A1, AP2B1, EEF1A2, HNRNPK, PABPC1, RBMX, RPLP0, RTRAF, SFPQ, SYNE1
TISSUES	BTO:0001490	Other source	3	43	1.36	0.58	0.014	DYNC1H1, SPTAN1, SYNE1
TISSUES	BTO:0000180	Cervical carcinoma cell	6	322	0.79	0.45	0.018	HNRNPD, HNRNPK, NCL, PIK3C3, PRKDC, XRCC5
TISSUES	BTO:0000445	Cerebral lobe	9	759	0.59	0.40	0.018	CAPZA2, CCT2, DDX17, DYNC1H1, ECPAS, EEF1A2, NOVA1, SEPTIN3, SYT1
TISSUES	BTO:0000202	Sense organ	11	1124	0.51	0.36	0.020	CAPZA2, CCT3, CCT7, DDX1, DYNC1H1, EIF3L, HNRNPK, NCL, PABPC1, RPLP0, RPLP2
TISSUES	BTO:0001253	Skin	11	1151	0.50	0.35	0.024	CCT3, CCT6A, CCT7, DDX17, EIF3L, HNRNPK, NCL, PABPC1, RPLP0, RPLP2, XRCC5

Table S5: Cerebellar vermis pathways enriched in downregulated proteins (FDR-adjusted $p < 0.05$) of children with idiopathic autism *(continued)*

Category	Term ID	Term description	Gene count	Back-ground	Strength	Signal	FDR	Matching proteins in network
TISSUES	BTO:0001042	Amygdala	5	226	0.86	0.44	0.025	CAPZA2, CCT2, DDX17, NOVA1, SYT1
TISSUES	BTO:0001355	Temporal lobe	8	633	0.62	0.38	0.025	CAPZA2, CCT2, DDX17, ECPAS, EEF1A2, NOVA1, SEPTIN3, SYT1
TISSUES	BTO:0000551	Lung cancer cell	4	131	1.00	0.46	0.026	CCT5, CCT6A, HNRNPK, JUP
TISSUES	BTO:0000439	Eye	9	835	0.55	0.35	0.032	CAPZA2, CCT3, CCT7, DDX1, DYNC1H1, EIF3L, NCL, PABPC1, RPLP0
TISSUES	BTO:0001493	Trunk	10	1014	0.51	0.34	0.032	CCT4, CS, EIF3L, HNRNPD, HNRNPK, PABPC1, RBMX, RPLP2, SEPTIN6, XRCC5
COMPARTMENTS	GOCC:0005832	Chaperonin-containing T-complex	8	10	2.42	4.01	4.4e-13	CCT2, CCT3, CCT4, CCT5, CCT6A, CCT7, CCT8, TCP1
COMPARTMENTS	GOCC:0043232	Intracellular non-membrane-bounded organelle	38	3309	0.58	0.90	1.3e-12	ACADVL, ANK1, CAPZA2, CCT2, CCT3, CCT4, CCT5, CCT6A, CCT7, CCT8, DDX1, DYNC1H1, DYNC1I1, DYNC1LI1, DYNC1LI2, DYNLRB1, ECPAS, HNRNPK, KRT9, NCKAP1, NCL, NOVA1, PABPC1, PIK3C3, PRKDC, PSPC1, RBMX, RPLP0, RPLP2, RTRAF, SEPTIN3, SEPTIN6, SFPQ, SPTAN1, SPTBN2, SYNE1, TCP1, XRCC5

Table S5: Cerebellar vermis pathways enriched in downregulated proteins (FDR-adjusted $p < 0.05$) of children with idiopathic autism *(continued)*

Category	Term ID	Term description	Gene count	Back-ground	Strength	Signal	FDR	Matching proteins in network
COMPARTMENTS	GOCC:0032991	Protein-containing complex	44	5325	0.43	0.67	7.1e-11	ANK1, AP2A1, AP2B1, API5, CAPZA2, CCT2, CCT3, CCT4, CCT5, CCT6A, CCT7, CCT8, DDX1, DOCK1, DYNC1H1, DYNC1I1, DYNC1LI1, DYNC1LI2, DYNLRB1, ECPAS, EEF1A2, EIF3L, FARSB, HNRNPD, HNRNPK, ITPR1, JUP, NCKAP1, NCL, PABPC1, PIK3C3, PRKDC, PSMD13, RBMX, RPLP0, RPLP2, RTRAF, SEPTIN3, SEPTIN6, SPTAN1, SPTBN2, SYNE1, TCP1, XRCC5
COMPARTMENTS	GOCC:0099080	Supramolecular complex	20	844	0.89	1.39	1.9e-10	ANK1, CCT2, CCT3, CCT4, CCT5, CCT6A, CCT7, CCT8, DDX1, DYNC1H1, DYNC1I1, DYNC1LI1, HNRNPK, KRT9, NCKAP1, NCL, PABPC1, RPLP0, SYNE1, TCP1
COMPARTMENTS	GOCC:0005856	Cytoskeleton	25	1575	0.72	1.07	4.5e-10	ANK1, CAPZA2, CCT2, CCT3, CCT4, CCT5, CCT6A, CCT7, CCT8, DYNC1H1, DYNC1I1, DYNC1LI1, DYNC1LI2, DYNLRB1, ECPAS, KRT9, NCKAP1, PIK3C3, RTRAF, SEPTIN3, SEPTIN6, SPTAN1, SPTBN2, SYNE1, TCP1

Table S5: Cerebellar vermis pathways enriched in downregulated proteins (FDR-adjusted $p < 0.05$) of children with idiopathic autism *(continued)*

Category	Term ID	Term description	Gene count	Back-ground	Strength	Signal	FDR	Matching proteins in network
COMPARTMENTS	GOCC:0043229	Intracellular organelle	53	9609	0.26	0.46	3.7e-08	ACADVL, ACAT1, ANK1, AP2A1, AP2B1, API5, CAPZA2, CCT2, CCT3, CCT4, CCT5, CCT6A, CCT7, CCT8, CS, DDX1, DDX17, DIP2B, DOCK1, DYNC1H1, DYNC1I1, DYNC1LI1, DYNC1LI2, DYNLRB1, ECPAS, EEF1A2, HNRNPD, HNRNPK, ITPR1, IVD, JUP, KRT9, NCKAP1, NCL, NOVA1, PABPC1, PIK3C3, PRKDC, PSMD13, PSPC1, RBMX, RPLP0, RPLP2, RTRAF, SEPTIN3, SEPTIN6, SFPQ, SPTAN1, SPTBN2, SYNE1, SYT1, TCP1, XRCC5
COMPARTMENTS	GOCC:0005737	Cytoplasm	49	8195	0.29	0.48	6.4e-08	ACADVL, ACAT1, ANK1, AP2A1, AP2B1, API5, ARHGAP26, CAPZA2, CCT2, CCT3, CCT4, CCT5, CCT6A, CCT7, CCT8, CS, DDX1, DIP2B, DOCK1, DYNC1H1, DYNC1I1, DYNC1LI1, DYNC1LI2, DYNLRB1, ECPAS, EEF1A2, EIF3L, FARSB, HNRNPD, HNRNPK, ITPR1, IVD, JUP, KRT9, NCKAP1, NCL, PABPC1, PIK3C3, PSMD13, RPLP0, RPLP2, RTRAF, SEPTIN3, SEPTIN6, SPTAN1, SPTBN2, SYNE1, SYT1, TCP1

Table S5: Cerebellar vermis pathways enriched in downregulated proteins (FDR-adjusted $p < 0.05$) of children with idiopathic autism *(continued)*

Category	Term ID	Term description	Gene count	Back-ground	Strength	Signal	FDR	Matching proteins in network
COMPARTMENTS	GOCC:0005622	Intracellular	56	11512	0.20	0.40	3.1e-07	ACADVL, ACAT1, ANK1, AP2A1, AP2B1, API5, ARHGAP26, CAPZA2, CCT2, CCT3, CCT4, CCT5, CCT6A, CCT7, CCT8, CS, DDX1, DDX17, DIP2B, DOCK1, DYNC1H1, DYNC1I1, DYNC1LI1, DYNC1LI2, DYNLRB1, ECPAS, EEF1A2, EIF3L, FARSB, HNRNPD, HNRNPK, ITPR1, IVD, JUP, KRT9, NCKAP1, NCL, NOVA1, PABPC1, PIK3C3, PRKDC, PSMD13, PSPC1, RBMX, RPLP0, RPLP2, RTRAF, SEPTIN3, SEPTIN6, SFPQ, SPTAN1, SPTBN2, SYNE1, SYT1, TCP1, XRCC5
COMPARTMENTS	GOCC:0099512	Supramolecular fiber	13	555	0.89	1.06	2.3e-06	ANK1, CCT2, CCT3, CCT4, CCT5, CCT6A, CCT7, CCT8, DYNC1H1, KRT9, NCKAP1, SYNE1, TCP1
COMPARTMENTS	GOCC:0005868	Cytoplasmic dynein complex	5	21	1.89	1.78	2.4e-06	DYNC1H1, DYNC1I1, DYNC1LI1, DYNC1LI2, DYNLRB1
COMPARTMENTS	GOCC:0099513	Polymeric cytoskeletal fiber	11	382	0.98	1.13	3.7e-06	CCT2, CCT3, CCT4, CCT5, CCT6A, CCT7, CCT8, DYNC1H1, KRT9, NCKAP1, TCP1

Table S5: Cerebellar vermis pathways enriched in downregulated proteins (FDR-adjusted $p < 0.05$) of children with idiopathic autism *(continued)*

Category	Term ID	Term description	Gene count	Back-ground	Strength	Signal	FDR	Matching proteins in network
COMPARTMENTS	GOCC:0015630	Microtubule cytoskeleton	16	973	0.73	0.88	3.7e-06	CCT2, CCT3, CCT4, CCT5, CCT6A, CCT7, CCT8, DYNC1H1, DYNC1I1, DYNC1LI1, DYNC1LI2, DYNLRB1, ECPAS, RTRAF, SPTAN1, TCP1
COMPARTMENTS	GOCC:0005874	Microtubule	9	233	1.10	1.22	6.2e-06	CCT2, CCT3, CCT4, CCT5, CCT6A, CCT7, CCT8, DYNC1H1, TCP1
COMPARTMENTS	GOCC:0110165	Cellular anatomical entity	58	14060	0.13	0.32	6.7e-05	ACADVL, ACAT1, ANK1, AP2A1, AP2B1, API5, ARHGAP26, CAPZA2, CCT2, CCT3, CCT4, CCT5, CCT6A, CCT7, CCT8, CS, DDX1, DDX17, DIP2B, DOCK1, DYNC1H1, DYNC1I1, DYNC1LI1, DYNC1LI2, DYNLRB1, ECPAS, EEF1A2, EIF3L, FARSB, HNRNPD, HNRNPK, INPP5A, ITPR1, IVD, JUP, KRT9, NCKAP1, NCL, NOVA1, PABPC1, PIK3C3, PRKDC, PSMD13, PSPC1, PTPRZ1, RBMX, RPLP0, RPLP2, RTRAF, SEPTIN3, SEPTIN6, SFPQ, SPTAN1, SPTBN2, SYNE1, SYT1, TCP1, XRCC5
COMPARTMENTS	GOCC:1990904	Ribonucleoprotein complex	11	564	0.81	0.80	1.2e-04	API5, DDX1, HNRNPD, HNRNPK, NCL, PABPC1, PRKDC, RBMX, RPLP0, RPLP2, XRCC5

Table S5: Cerebellar vermis pathways enriched in downregulated proteins (FDR-adjusted p < 0.05) of children with idiopathic autism *(continued)*

Category	Term ID	Term description	Gene count	Back-ground	Strength	Signal	FDR	Matching proteins in network
COMPARTMENTS	GOCC:0005829	Cytosol	25	3054	0.43	0.51	1.3e-04	ANK1, AP2B1, ARHGAP26, CAPZA2, CCT2, CCT3, CCT4, CCT5, CCT6A, CCT7, CCT8, DDX1, DOCK1, DYNC1H1, DYNC1LI1, DYNC1LI2, FARSB, HNRNPD, KRT9, PABPC1, PIK3C3, RPLP0, RPLP2, SPTBN2, TCP1
COMPARTMENTS	GOCC:0002199	Zona pellucida receptor complex	3	5	2.29	1.28	1.5e-04	CCT2, CCT3, CCT8
COMPARTMENTS	GOCC:0005813	Centrosome	10	470	0.84	0.80	1.7e-04	CCT4, CCT5, CCT8, DYNC1H1, DYNC1LI1, DYNC1LI2, DYNLRB1, ECPAS, RTRAF, TCP1
COMPARTMENTS	GOCC:0008091	Spectrin	3	12	1.91	0.97	1.1e-03	ANK1, SPTAN1, SPTBN2
COMPARTMENTS	GOCC:0036464	Cytoplasmic ribonucleoprotein granule	6	179	1.04	0.73	1.8e-03	DDX1, HNRNPK, NCL, PABPC1, RPLP0, SYNE1
COMPARTMENTS	GOCC:0005938	Cell cortex	6	190	1.02	0.70	2.4e-03	ANK1, NCL, SEPTIN3, SEPTIN6, SPTAN1, SPTBN2
COMPARTMENTS	GOCC:0031982	Vesicle	18	2125	0.44	0.43	3.3e-03	AP2A1, AP2B1, CCT2, CCT8, DYNC1H1, DYNC1I1, DYNC1LI1, ECPAS, HNRNPK, ITPR1, JUP, NCL, PABPC1, PIK3C3, PSMD13, RBMX, SPTAN1, SYT1
COMPARTMENTS	GOCC:1902494	Catalytic complex	15	1710	0.46	0.38	0.011	DYNC1H1, DYNC1I1, DYNC1LI1, DYNC1LI2, DYNLRB1, ECPAS, FARSB, HNRNPK, ITPR1, PABPC1, PIK3C3, PRKDC, PSMD13, RBMX, XRCC5

Table S5: Cerebellar vermis pathways enriched in downregulated proteins (FDR-adjusted $p < 0.05$) of children with idiopathic autism *(continued)*

Category	Term ID	Term description	Gene count	Back-ground	Strength	Signal	FDR	Matching proteins in network
COMPARTMENTS	GOCC:0106002	mCRD-mediated mRNA stability complex	2	5	2.12	0.63	0.014	HNRNPD, PABPC1
COMPARTMENTS	GOCC:0030122	AP-2 adaptor complex	2	7	1.97	0.55	0.022	AP2A1, AP2B1
COMPARTMENTS	GOCC:0030141	Secretory granule	9	719	0.61	0.39	0.022	CCT2, CCT8, DYNC1H1, DYNC1LI1, ITPR1, JUP, PSMD13, SPTAN1, SYT1
COMPARTMENTS	GOCC:0032807	DNA ligase IV complex	2	7	1.97	0.55	0.022	PRKDC, XRCC5
COMPARTMENTS	GOCC:0072669	tRNA-splicing ligase complex	2	7	1.97	0.55	0.022	DDX1, RTRAF
COMPARTMENTS	GOCC:0042382	Paraspeckles	2	8	1.91	0.54	0.025	PSPC1, SFPQ
COMPARTMENTS	GOCC:0043227	Membrane-bounded organelle	41	9083	0.17	0.25	0.025	ACADVL, ACAT1, ANK1, AP2A1, AP2B1, API5, CCT2, CCT5, CCT8, CS, DDX1, DDX17, DIP2B, DOCK1, DYNC1H1, DYNC1I1, DYNC1LI1, ECPAS, EEF1A2, HNRNPD, HNRNPK, ITPR1, IVD, JUP, NCL, NOVA1, PABPC1, PIK3C3, PRKDC, PSMD13, PSPC1, RBMX, RPLP0, RPLP2, RTRAF, SEPTIN6, SFPQ, SPTAN1, SYNE1, SYT1, XRCC5
COMPARTMENTS	GOCC:0014731	Spectrin-associated cytoskeleton	2	9	1.86	0.52	0.028	ANK1, SPTBN2
COMPARTMENTS	GOCC:0070418	DNA-dependent protein kinase complex	2	9	1.86	0.52	0.028	PRKDC, XRCC5
COMPARTMENTS	GOCC:0031410	Cytoplasmic vesicle	14	1738	0.42	0.31	0.034	AP2A1, AP2B1, CCT2, CCT8, DYNC1H1, DYNC1I1, DYNC1LI1, ECPAS, ITPR1, JUP, PIK3C3, PSMD13, SPTAN1, SYT1

Table S5: Cerebellar vermis pathways enriched in downregulated proteins (FDR-adjusted p < 0.05) of children with idiopathic autism *(continued)*

Category	Term ID	Term description	Gene count	Back-ground	Strength	Signal	FDR	Matching proteins in network
COMPARTMENTS	GOCC:0030666	Endocytic vesicle membrane	4	135	0.99	0.41	0.042	AP2A1, AP2B1, PIK3C3, SYT1
COMPARTMENTS	GOCC:0034774	Secretory granule lumen	5	241	0.83	0.38	0.044	CCT2, CCT8, DYNC1H1, JUP, SPTAN1
COMPARTMENTS	GOCC:0005940	Septin ring	2	14	1.67	0.44	0.048	SEPTIN3, SEPTIN6
COMPARTMENTS	GOCC:0010494	Cytoplasmic stress granule	3	63	1.19	0.41	0.048	DDX1, HNRNPK, PABPC1
COMPARTMENTS	GOCC:0031105	Septin complex	2	14	1.67	0.44	0.048	SEPTIN3, SEPTIN6
UniProt Keywords	KW-0007	Acetylation	40	3362	0.59	0.96	1.7e-14	ACADVL, ACAT1, AP2B1, API5, CAPZA2, CCT2, CCT3, CCT4, CCT5, CCT6A, CCT7, CCT8, CS, DDX1, DDX17, DYNC1H1, DYNC1I1, DYNLRB1, ECPAS, EEF1A2, EIF3L, HNRNPD, HNRNPK, IVD, JUP, NCKAP1, NCL, PABPC1, PRKDC, PSMD13, PSPC1, RBMX, RPLP2, RTRAF, SEPTIN6, SFPQ, SPTAN1, SPTBN2, TCP1, XRCC5
UniProt Keywords	KW-0963	Cytoplasm	39	5095	0.40	0.58	8.2e-08	ANK1, API5, ARHGAP26, CCT2, CCT3, CCT4, CCT5, CCT6A, CCT7, CCT8, DDX1, DDX17, DOCK1, DYNC1H1, DYNC1I1, DYNC1LI1, DYNC1LI2, DYNLRB1, ECPAS, EIF3L, FARSB, HNRNPD, HNRNPK, ITPR1, JUP, NCL, PABPC1, PSPC1, RPLP0, RTRAF, SEPTIN3, SEPTIN6, SFPQ, SPTAN1, SPTBN2, SYNE1, SYT1, TCP1, UBASH3B

Table S5: Cerebellar vermis pathways enriched in downregulated proteins (FDR-adjusted p < 0.05) of children with idiopathic autism *(continued)*

Category	Term ID	Term description	Gene count	Back-ground	Strength	Signal	FDR	Matching proteins in network
UniProt Keywords	KW-0597	Phosphoprotein	48	8122	0.29	0.47	2.3e-07	ACADVL, ANK1, AP2A1, AP2B1, API5, CAPZA2, CCT2, CCT3, CCT4, CCT5, CCT6A, CCT8, DDX1, DDX17, DIP2B, DOCK1, DYNC1H1, DYNC1I1, DYNC1LI1, DYNC1LI2, ECPAS, EEF1A2, EIF3L, HNRNPD, HNRNPK, ITPR1, JUP, KRT9, NCL, NOVA1, PABPC1, PIK3C3, PRKDC, PSPC1, PTPRZ1, RBMX, RPLP0, RPLP2, SEPTIN3, SEPTIN6, SFPQ, SPTAN1, SPTBN2, SYNE1, SYT1, TCP1, UBASH3B, XRCC5
UniProt Keywords	KW-0206	Cytoskeleton	19	1235	0.70	0.90	4.4e-07	ANK1, ARHGAP26, CCT4, CCT5, CCT8, DYNC1H1, DYNC1I1, DYNC1LI1, DYNC1LI2, DYNLRB1, ECPAS, JUP, RTRAF, SEPTIN3, SEPTIN6, SPTAN1, SPTBN2, SYNE1, TCP1
UniProt Keywords	KW-0243	Dynein	5	33	1.70	1.50	1.4e-05	DYNC1H1, DYNC1I1, DYNC1LI1, DYNC1LI2, DYNLRB1
UniProt Keywords	KW-0143	Chaperone	8	197	1.12	1.16	1.8e-05	CCT2, CCT3, CCT4, CCT5, CCT6A, CCT7, CCT8, TCP1
UniProt Keywords	KW-0488	Methylation	15	973	0.70	0.79	1.8e-05	API5, CCT4, CCT7, CS, DDX17, DYNC1LI2, EEF1A2, HNRNPD, HNRNPK, INPP5A, NCL, PABPC1, PSPC1, RBMX, SFPQ

Table S5: Cerebellar vermis pathways enriched in downregulated proteins (FDR-adjusted $p < 0.05$) of children with idiopathic autism *(continued)*

Category	Term ID	Term description	Gene count	Back-ground	Strength	Signal	FDR	Matching proteins in network
UniProt Keywords	KW-0547	Nucleotide-binding	20	1776	0.57	0.67	1.8e-05	CCT2, CCT3, CCT4, CCT5, CCT6A, CCT7, CCT8, DDX1, DDX17, DYNC1H1, DYNC1LI1, DYNC1LI2, EEF1A2, FARSB, PIK3C3, PRKDC, SEPTIN3, SEPTIN6, TCP1, XRCC5
UniProt Keywords	KW-0067	ATP-binding	17	1379	0.61	0.68	3.9e-05	CCT2, CCT3, CCT4, CCT5, CCT6A, CCT7, CCT8, DDX1, DDX17, DYNC1H1, DYNC1LI1, DYNC1LI2, FARSB, PIK3C3, PRKDC, TCP1, XRCC5
UniProt Keywords	KW-0694	RNA-binding	11	686	0.72	0.67	4.6e-04	DDX1, DDX17, HNRNPD, HNRNPK, NCL, NOVA1, PABPC1, PSPC1, RBMX, RTRAF, SFPQ
UniProt Keywords	KW-1017	Isopeptide bond	17	1717	0.51	0.52	6.1e-04	CCT2, CCT3, CCT5, CCT6A, CCT7, CCT8, DDX1, DDX17, ECPAS, HNRNPD, HNRNPK, ITPR1, NCL, RBMX, RPLP0, SFPQ, XRCC5
UniProt Keywords	KW-0117	Actin capping	3	19	1.71	0.86	2.2e-03	CAPZA2, SPTAN1, SPTBN2
UniProt Keywords	KW-0505	Motor protein	5	132	1.09	0.70	3.1e-03	DYNC1H1, DYNC1I1, DYNC1LI1, DYNC1LI2, DYNLRB1
UniProt Keywords	KW-0832	Ubl conjugation	17	2399	0.37	0.30	0.030	CCT2, CCT3, CCT5, CCT6A, CCT7, CCT8, DDX1, ECPAS, HNRNPD, HNRNPK, ITPR1, NCL, PRKDC, RBMX, RPLP0, SFPQ, XRCC5
UniProt Keywords	KW-0507	mRNA processing	6	349	0.75	0.39	0.032	DDX1, DDX17, HNRNPK, PABPC1, RBMX, SFPQ
UniProt Keywords	KW-0090	Biological rhythms	4	145	0.96	0.39	0.046	HNRNPD, PRKDC, PSPC1, SFPQ

Table S5: Cerebellar vermis pathways enriched in downregulated proteins (FDR-adjusted p < 0.05) of children with idiopathic autism *(continued)*

Category	Term ID	Term description	Gene count	Back-ground	Strength	Signal	FDR	Matching proteins in network
Pfam	PF00118	TCP-1/cpn60 chaperonin family	8	15	2.24	3.60	4.9e-12	CCT2, CCT3, CCT4, CCT5, CCT6A, CCT7, CCT8, TCP1
InterPro	IPR002194	Chaperonin TCP-1, conserved site	8	9	2.47	3.96	7.6e-13	CCT2, CCT3, CCT4, CCT5, CCT6A, CCT7, CCT8, TCP1
InterPro	IPR017998	Chaperone tailless complex polypeptide 1 (TCP-1)	8	10	2.42	3.94	7.6e-13	CCT2, CCT3, CCT4, CCT5, CCT6A, CCT7, CCT8, TCP1
InterPro	IPR027413	GroEL-like equatorial domain superfamily	8	14	2.27	3.67	3.3e-12	CCT2, CCT3, CCT4, CCT5, CCT6A, CCT7, CCT8, TCP1
InterPro	IPR002423	Chaperonin Cpn60/GroEL/TCP-1 family	8	15	2.24	3.64	3.8e-12	CCT2, CCT3, CCT4, CCT5, CCT6A, CCT7, CCT8, TCP1
InterPro	IPR027409	GroEL-like apical domain superfamily	8	15	2.24	3.64	3.8e-12	CCT2, CCT3, CCT4, CCT5, CCT6A, CCT7, CCT8, TCP1
InterPro	IPR027410	TCP-1-like chaperonin intermediate domain superfamily	8	15	2.24	3.64	3.8e-12	CCT2, CCT3, CCT4, CCT5, CCT6A, CCT7, CCT8, TCP1
InterPro	IPR016024	Armadillo-type fold	9	337	0.94	0.75	9.6e-04	AP2A1, AP2B1, API5, DOCK1, ECPAS, ITPR1, JUP, PIK3C3, PRKDC
InterPro	IPR002017	Spectrin repeat	3	22	1.65	0.43	0.049	SPTAN1, SPTBN2, SYNE1
InterPro	IPR003954	RNA recognition motif domain, eukaryote	3	23	1.63	0.43	0.049	NCL, PABPC1, RBMX
InterPro	IPR008467	Dynein 1 light intermediate chain	2	2	2.52	0.45	0.049	DYNC1LI1, DYNC1LI2

Table S6: Cerebellar vermis pathways enriched in upregulated proteins (FDR-adjusted p < 0.05) of children with idiopathic autism

Category	Term ID	Term description	Gene count	Back-ground	Strength	Signal	FDR	Matching proteins in network
GO Process	GO:0006081	Cellular aldehyde metabolic process	4	62	1.66	0.58	0.035	ALDH2, ALDH7A1, GLO1, TPI1
GO Process	GO:0009611	Response to wounding	7	444	1.05	0.49	0.035	ANXA5, DPYSL3, GFAP, PHB2, PLEC, SERPINA1, VCL
GO Function	GO:0045296	Cadherin binding	8	334	1.23	1.17	8.5e-05	AHNAK, ENO1, EZR, HSPA1B, PLEC, PRDX1, RDX, VCL
GO Function	GO:0050839	Cell adhesion molecule binding	9	560	1.05	0.98	1.4e-04	AHNAK, ENO1, EZR, GFAP, HSPA1B, PLEC, PRDX1, RDX, VCL
GO Function	GO:0004029	Aldehyde dehydrogenase (NAD+) activity	3	16	2.12	0.97	4.0e-03	ALDH1L1, ALDH2, ALDH7A1
GO Function	GO:0019899	Enzyme binding	12	2084	0.61	0.43	0.012	ENO1, EZR, GDI1, GFAP, GSN, HSPA1B, HSPD1, MACROH2A1, RDX, SERPINA1, TPI1, VCL
GO Function	GO:0043878	glyceraldehyde-3-phosphate dehydrogenase (NAD+) (non-phosphorylating) activity	2	7	2.30	0.57	0.043	ALDH2, ALDH7A1
GO Function	GO:0051287	NAD binding	3	56	1.58	0.54	0.043	ALDH2, HADHA, NNT
GO Function	GO:0005488	Binding	27	12838	0.17	0.21	0.046	AHNAK, ALDH2, ALDH7A1, ANXA5, C3, DPYSL3, ENO1, EZR, GDI1, GFAP, GLO1, GSN, HADHA, HSPA1B, HSPD1, MACROH2A1, NNT, NPEPPS, PEPD, PHB2, PLEC, PRDX1, PSAT1, RDX, SERPINA1, TPI1, VCL

Table S6: Cerebellar vermis pathways enriched in upregulated proteins (FDR-adjusted $p < 0.05$) of children with idiopathic autism *(continued)*

Category	Term ID	Term description	Gene count	Back-ground	Strength	Signal	FDR	Matching proteins in network
GO Function	GO:0042802	Identical protein binding	11	2144	0.56	0.33	0.046	AHNAK, ALDH7A1, DPYSL3, ENO1, EZR, GFAP, PHB2, PRDX1, PSAT1, SERPINA1, TPI1
GO Component	GO:0070062	Extracellular exosome	22	2096	0.87	1.25	1.8e-13	AHNAK, ALDH1L1, ALDH2, ALDH7A1, ANXA5, C3, ENO1, EZR, GLO1, GSN, HSPA1B, HSPD1, MACROH2A1, NPEPPS, PEPD, PLEC, PRDX1, PSAT1, RDX, SERPINA1, TPI1, VCL
GO Component	GO:0005615	Extracellular space	23	3247	0.70	0.89	1.8e-11	AHNAK, ALDH1L1, ALDH2, ALDH7A1, ANXA5, C3, DPYSL3, ENO1, EZR, GLO1, GSN, HSPA1B, HSPD1, MACROH2A1, NPEPPS, PEPD, PLEC, PRDX1, PSAT1, RDX, SERPINA1, TPI1, VCL
GO Component	GO:0031982	Vesicle	23	3957	0.61	0.73	1.1e-09	AHNAK, ALDH1L1, ALDH2, ALDH7A1, ANXA5, C3, DPYSL3, ENO1, EZR, GLO1, GSN, HSPA1B, HSPD1, MACROH2A1, NPEPPS, PEPD, PLEC, PRDX1, PSAT1, RDX, SERPINA1, TPI1, VCL
GO Component	GO:0005925	Focal adhesion	8	416	1.13	1.18	2.3e-05	AHNAK, ANXA5, EZR, GSN, HSPA1B, PLEC, RDX, VCL
GO Component	GO:0043034	Costamere	3	16	2.12	1.32	5.0e-04	AHNAK, PLEC, VCL
GO Component	GO:0005829	Cytosol	19	5438	0.39	0.37	2.1e-03	AHNAK, ALDH1L1, ALDH7A1, ANXA5, DPYSL3, ENO1, EZR, GDI1, GFAP, GLO1, GSN, HSPA1B, HSPD1, NPEPPS, PLEC, PRDX1, PSAT1, TPI1, VCL

Table S6: Cerebellar vermis pathways enriched in upregulated proteins (FDR-adjusted $p < 0.05$) of children with idiopathic autism *(continued)*

Category	Term ID	Term description	Gene count	Back-ground	Strength	Signal	FDR	Matching proteins in network
GO Component	GO:0030863	Cortical cytoskeleton	4	102	1.44	0.94	2.3e-03	EZR, GSN, RDX, VCL
GO Component	GO:0043227	Membrane-bounded organelle	28	13188	0.17	0.25	2.3e-03	AHNAK, ALDH1L1, ALDH2, ALDH7A1, ANXA5, C3, DPYSL3, ENO1, EZR, GDI1, GFAP, GLO1, GSN, HADHA, HSPA1B, HSPD1, MACROH2A1, NNT, NPEPPS, PEPD, PHB2, PLEC, PRDX1, PSAT1, RDX, SERPINA1, TPI1, VCL
GO Component	GO:0072562	Blood microparticle	4	118	1.38	0.86	3.7e-03	ANXA5, C3, GSN, HSPA1B
GO Component	GO:1904813	ficolin-1-rich granule lumen	4	124	1.36	0.83	4.2e-03	GSN, HSPA1B, SERPINA1, VCL
GO Component	GO:0042383	Sarcolemma	4	132	1.33	0.80	5.0e-03	AHNAK, PLEC, RDX, VCL
GO Component	GO:0015629	Actin cytoskeleton	6	482	0.94	0.63	6.3e-03	AHNAK, DPYSL3, EZR, GSN, RDX, VCL
GO Component	GO:0005938	Cell cortex	5	304	1.06	0.66	7.6e-03	ENO1, EZR, GSN, RDX, VCL
GO Component	GO:0030864	Cortical actin cytoskeleton	3	73	1.46	0.67	0.016	GSN, RDX, VCL
GO Component	GO:0005737	Cytoplasm	26	12056	0.18	0.23	0.017	AHNAK, ALDH1L1, ALDH2, ALDH7A1, ANXA5, C3, DPYSL3, ENO1, EZR, GDI1, GFAP, GLO1, GSN, HADHA, HSPA1B, HSPD1, NNT, NPEPPS, PHB2, PLEC, PRDX1, PSAT1, RDX, SERPINA1, TPI1, VCL
GO Component	GO:0030016	Myofibril	4	237	1.07	0.51	0.031	AHNAK, ENO1, PLEC, VCL
GO Component	GO:0030054	Cell junction	10	2115	0.52	0.34	0.033	AHNAK, ANXA5, DPYSL3, EZR, GSN, HSPA1B, PHB2, PLEC, RDX, VCL
GO Component	GO:0099512	Supramolecular fiber	7	1000	0.69	0.40	0.033	AHNAK, DPYSL3, ENO1, EZR, GFAP, PLEC, VCL
GO Component	GO:0005903	Brush border	3	106	1.30	0.54	0.034	EZR, PLEC, VCL

Table S6: Cerebellar vermis pathways enriched in upregulated proteins (FDR-adjusted $p < 0.05$) of children with idiopathic autism *(continued)*

Category	Term ID	Term description	Gene count	Back-ground	Strength	Signal	FDR	Matching proteins in network
GO Component	GO:0099503	Secretory vesicle	7	1047	0.67	0.38	0.037	C3, DPYSL3, GSN, HSPA1B, HSPD1, SERPINA1, VCL
KEGG	hsa00010	Glycolysis / Gluconeogenesis	4	64	1.64	1.14	8.3e-04	ALDH2, ALDH7A1, ENO1, TPI1
KEGG	hsa00410	beta-Alanine metabolism	3	29	1.86	1.05	2.1e-03	ALDH2, ALDH7A1, HADHA
KEGG	hsa00071	Fatty acid degradation	3	42	1.70	1.00	2.5e-03	ALDH2, ALDH7A1, HADHA
KEGG	hsa00280	Valine, leucine and isoleucine degradation	3	46	1.66	0.99	2.5e-03	ALDH2, ALDH7A1, HADHA
KEGG	hsa00380	Tryptophan metabolism	3	41	1.71	1.00	2.5e-03	ALDH2, ALDH7A1, HADHA
KEGG	hsa00620	Pyruvate metabolism	3	36	1.77	1.01	2.5e-03	ALDH2, ALDH7A1, GLO1
KEGG	hsa01200	Carbon metabolism	4	116	1.38	0.91	2.5e-03	ENO1, HADHA, PSAT1, TPI1
KEGG	hsa05134	Legionellosis	3	55	1.58	0.94	3.1e-03	C3, HSPA1B, HSPD1
KEGG	hsa00310	Lysine degradation	3	60	1.55	0.91	3.6e-03	ALDH2, ALDH7A1, HADHA
KEGG	hsa01230	Biosynthesis of amino acids	3	73	1.46	0.82	5.6e-03	ENO1, PSAT1, TPI1
KEGG	hsa04810	Regulation of actin cytoskeleton	4	209	1.13	0.70	6.7e-03	EZR, GSN, RDX, VCL
KEGG	hsa00340	Histidine metabolism	2	21	1.83	0.75	0.013	ALDH2, ALDH7A1
KEGG	hsa00053	Ascorbate and aldarate metabolism	2	26	1.73	0.70	0.017	ALDH2, ALDH7A1
KEGG	hsa01100	Metabolic pathways	8	1435	0.59	0.40	0.017	ALDH2, ALDH7A1, ENO1, GLO1, HADHA, NNT, PSAT1, TPI1
KEGG	hsa00260	Glycine, serine and threonine metabolism	2	38	1.57	0.58	0.033	ALDH7A1, PSAT1
KEGG	hsa00330	Arginine and proline metabolism	2	47	1.48	0.52	0.046	ALDH2, ALDH7A1
WikiPathways	WP51	Regulation of actin cytoskeleton	4	149	1.28	0.49	0.048	EZR, GSN, RDX, VCL

Table S6: Cerebellar vermis pathways enriched in upregulated proteins (FDR-adjusted $p < 0.05$) of children with idiopathic autism *(continued)*

Category	Term ID	Term description	Gene count	Back-ground	Strength	Signal	FDR	Matching proteins in network
Monarch	HP:0001005	Dermatological manifestations of systemic disorders	7	259	1.28	0.99	8.5e-04	GSN, HADHA, PEPD, PRDX1, PSAT1, SERPINA1, TPI1
Monarch	HP:0002793	Abnormal pattern of respiration	9	624	1.01	0.82	8.5e-04	ALDH7A1, GFAP, GSN, HADHA, HSPD1, PLEC, PRDX1, SERPINA1, VCL
Monarch	HP:0002795	Abnormal respiratory system physiology	11	1151	0.83	0.68	9.7e-04	ALDH7A1, GFAP, GSN, HADHA, HSPD1, PEPD, PLEC, PRDX1, SERPINA1, TPI1, VCL
Monarch	HP:0002086	Abnormality of the respiratory system	12	1636	0.71	0.55	2.9e-03	ALDH7A1, GFAP, GSN, HADHA, HSPD1, PEPD, PLEC, PRDX1, PSAT1, SERPINA1, TPI1, VCL
Monarch	HP:0003011	Abnormality of the musculature	14	2384	0.62	0.49	2.9e-03	ALDH7A1, EZR, GDI1, GFAP, GSN, HADHA, HSPD1, MACROH2A1, PLEC, PRDX1, PSAT1, SERPINA1, TPI1, VCL
Monarch	HP:0011121	Abnormality of skin morphology	12	1648	0.71	0.55	2.9e-03	ALDH2, GFAP, GSN, HADHA, NNT, PEPD, PLEC, PRDX1, PSAT1, SERPINA1, TPI1, VCL
Monarch	HP:0011354	Generalized abnormality of skin	9	821	0.89	0.66	2.9e-03	ALDH2, GSN, HADHA, PEPD, PLEC, PRDX1, PSAT1, SERPINA1, TPI1
Monarch	HP:0011804	Abnormal muscle physiology	13	2088	0.64	0.51	2.9e-03	ALDH7A1, EZR, GDI1, GFAP, GSN, HADHA, HSPD1, PLEC, PRDX1, PSAT1, SERPINA1, TPI1, VCL
Monarch	HP:0025031	Abnormality of the digestive system	14	2389	0.62	0.49	2.9e-03	ALDH7A1, EZR, GDI1, GFAP, GSN, HADHA, HSPD1, NNT, PEPD, PLEC, PRDX1, PSAT1, SERPINA1, TPI1
Monarch	HP:0001638	Cardiomyopathy	7	453	1.04	0.73	3.3e-03	GSN, HADHA, NNT, PRDX1, SERPINA1, TPI1, VCL

Table S6: Cerebellar vermis pathways enriched in upregulated proteins (FDR-adjusted $p < 0.05$) of children with idiopathic autism *(continued)*

Category	Term ID	Term description	Gene count	Back-ground	Strength	Signal	FDR	Matching proteins in network
Monarch	HP:0009121	Abnormal axial skeleton morphology	13	2206	0.62	0.49	3.3e-03	ALDH7A1, EZR, GDI1, GFAP, HADHA, HSPD1, MACROH2A1, PEPD, PLEC, PRDX1, PSAT1, SERPINA1, TPI1
Monarch	HP:0011025	Abnormal cardiovascular system physiology	10	1169	0.78	0.58	3.3e-03	ALDH2, GFAP, GSN, HADHA, NNT, PLEC, PRDX1, SERPINA1, TPI1, VCL
Monarch	HP:0000118	Phenotypic abnormality	19	5129	0.42	0.37	3.6e-03	ALDH2, ALDH7A1, C3, EZR, GDI1, GFAP, GSN, HADHA, HSPD1, MACROH2A1, NNT, PEPD, PLEC, PRDX1, PSAT1, RDX, SERPINA1, TPI1, VCL
Monarch	HP:0000952	Jaundice	5	175	1.30	0.83	3.6e-03	HADHA, PEPD, PRDX1, SERPINA1, TPI1
Monarch	HP:0001574	Abnormality of the integument	13	2266	0.61	0.48	3.6e-03	ALDH2, GFAP, GSN, HADHA, MACROH2A1, NNT, PEPD, PLEC, PRDX1, PSAT1, SERPINA1, TPI1, VCL
Monarch	HP:0011805	Abnormal skeletal muscle morphology	11	1537	0.70	0.53	3.6e-03	GDI1, GFAP, GSN, HADHA, HSPD1, MACROH2A1, PLEC, PSAT1, SERPINA1, TPI1, VCL
Monarch	HP:0000929	Abnormal skull morphology	12	1954	0.64	0.49	3.8e-03	ALDH7A1, EZR, GDI1, GFAP, HADHA, HSPD1, MACROH2A1, PEPD, PLEC, PRDX1, PSAT1, SERPINA1
Monarch	HP:0033127	Abnormality of the musculoskeletal system	15	3173	0.52	0.43	3.8e-03	ALDH7A1, EZR, GDI1, GFAP, GSN, HADHA, HSPD1, MACROH2A1, PEPD, PLEC, PRDX1, PSAT1, SERPINA1, TPI1, VCL
Monarch	HP:0002094	Dyspnea	6	350	1.08	0.71	5.2e-03	ALDH7A1, HADHA, PLEC, PRDX1, SERPINA1, VCL

Table S6: Cerebellar vermis pathways enriched in upregulated proteins (FDR-adjusted $p < 0.05$) of children with idiopathic autism *(continued)*

Category	Term ID	Term description	Gene count	Back-ground	Strength	Signal	FDR	Matching proteins in network
Monarch	HP:0001626	Abnormality of the cardiovascular system	13	2438	0.57	0.44	5.3e-03	ALDH2, ALDH7A1, GFAP, GSN, HADHA, NNT, PEPD, PLEC, PRDX1, PSAT1, SERPINA1, TPI1, VCL
Monarch	HP:0002715	Abnormality of the immune system	11	1682	0.66	0.49	5.3e-03	C3, GFAP, GSN, HADHA, NNT, PEPD, PLEC, PRDX1, SERPINA1, TPI1, VCL
Monarch	HP:0010978	Abnormality of immune system physiology	10	1347	0.72	0.52	5.3e-03	C3, GFAP, GSN, HADHA, NNT, PEPD, PLEC, PRDX1, SERPINA1, TPI1
Monarch	HP:0011442	Abnormal central motor function	11	1689	0.66	0.49	5.3e-03	EZR, GDI1, GFAP, GSN, HADHA, HSPD1, NNT, PLEC, PRDX1, PSAT1, TPI1
Monarch	HP:0001396	Cholestasis	5	223	1.20	0.71	7.3e-03	HADHA, PEPD, PRDX1, SERPINA1, TPI1
Monarch	HP:0000707	Abnormality of the nervous system	15	3471	0.48	0.38	8.1e-03	ALDH2, ALDH7A1, EZR, GDI1, GFAP, GSN, HADHA, HSPD1, NNT, PEPD, PLEC, PRDX1, PSAT1, SERPINA1, TPI1
Monarch	HP:0001324	Muscle weakness	8	861	0.82	0.55	8.1e-03	GDI1, GFAP, GSN, HADHA, HSPD1, PLEC, SERPINA1, TPI1
Monarch	HP:0001939	Abnormality of metabolism/homeostasis	12	2168	0.59	0.44	8.1e-03	ALDH2, ALDH7A1, GFAP, GSN, HADHA, NNT, PEPD, PLEC, PRDX1, PSAT1, SERPINA1, VCL
Monarch	HP:0011458	Abdominal symptom	10	1448	0.69	0.48	8.1e-03	ALDH7A1, EZR, GFAP, HADHA, HSPD1, NNT, PLEC, PRDX1, PSAT1, SERPINA1
Monarch	HP:0000708	Behavioral abnormality	11	1839	0.62	0.45	8.7e-03	ALDH7A1, EZR, GDI1, GFAP, GSN, HADHA, PLEC, PRDX1, PSAT1, SERPINA1, TPI1

Table S6: Cerebellar vermis pathways enriched in upregulated proteins (FDR-adjusted $p < 0.05$) of children with idiopathic autism *(continued)*

Category	Term ID	Term description	Gene count	Back-ground	Strength	Signal	FDR	Matching proteins in network
Monarch	HP:0011015	Abnormal blood glucose concentration	5	252	1.14	0.66	0.010	ALDH7A1, HADHA, NNT, PRDX1, SERPINA1
Monarch	HP:0001263	Global developmental delay	10	1559	0.65	0.45	0.011	ALDH7A1, EZR, GDI1, HADHA, HSPD1, PEPD, PLEC, PRDX1, PSAT1, TPI1
Monarch	HP:0011446	Abnormality of higher mental function	12	2301	0.56	0.41	0.011	ALDH7A1, EZR, GDI1, GFAP, GSN, HADHA, HSPD1, NNT, PEPD, PLEC, PRDX1, SERPINA1
Monarch	HP:0012379	Abnormal enzyme/coenzyme activity	6	447	0.98	0.59	0.011	ALDH2, HADHA, PEPD, PLEC, PRDX1, SERPINA1
Monarch	HP:0012638	Abnormal nervous system physiology	14	3154	0.49	0.37	0.011	ALDH7A1, EZR, GDI1, GFAP, GSN, HADHA, HSPD1, NNT, PEPD, PLEC, PRDX1, PSAT1, SERPINA1, TPI1
Monarch	HP:0012759	Neurodevelopmental abnormality	12	2323	0.56	0.41	0.011	ALDH7A1, EZR, GDI1, GFAP, HADHA, HSPD1, NNT, PEPD, PLEC, PRDX1, PSAT1, TPI1
Monarch	HP:0002045	Hypothermia	3	45	1.67	0.74	0.013	GFAP, HADHA, PRDX1
Monarch	HP:0001250	Seizure	10	1607	0.64	0.44	0.013	ALDH7A1, EZR, GDI1, GFAP, HADHA, HSPD1, NNT, PEPD, PRDX1, PSAT1
Monarch	HP:0000079	Abnormality of the urinary system	10	1657	0.63	0.42	0.017	C3, GDI1, GSN, HADHA, HSPD1, NNT, PEPD, PLEC, PRDX1, SERPINA1
Monarch	HP:0000234	Abnormality of the head	13	2865	0.50	0.36	0.017	ALDH7A1, EZR, GDI1, GFAP, GSN, HADHA, HSPD1, MACROH2A1, PEPD, PLEC, PRDX1, PSAT1, SERPINA1
Monarch	HP:0011014	Abnormal glucose homeostasis	6	527	0.90	0.50	0.023	ALDH7A1, GFAP, HADHA, NNT, PRDX1, SERPINA1

Table S6: Cerebellar vermis pathways enriched in upregulated proteins (FDR-adjusted $p < 0.05$) of children with idiopathic autism *(continued)*

Category	Term ID	Term description	Gene count	Back-ground	Strength	Signal	FDR	Matching proteins in network
Monarch	HP:0000759	Abnormal peripheral nervous system morphology	6	530	0.90	0.49	0.023	GDI1, GFAP, GSN, PLEC, PRDX1, TPI1
Monarch	HP:0012639	Abnormal nervous system morphology	12	2546	0.52	0.36	0.023	ALDH2, ALDH7A1, EZR, GDI1, GFAP, GSN, HADHA, HSPD1, PLEC, PRDX1, PSAT1, TPI1
Monarch	HP:0100022	Abnormality of movement	10	1758	0.60	0.39	0.024	EZR, GDI1, GFAP, GSN, HADHA, HSPD1, PLEC, PRDX1, PSAT1, TPI1
Monarch	HP:0025032	Abnormality of digestive system physiology	10	1763	0.60	0.39	0.024	ALDH7A1, EZR, GFAP, HADHA, HSPD1, NNT, PLEC, PRDX1, PSAT1, SERPINA1
Monarch	EFO:0010155	Taste liking measurement	2	9	2.19	0.66	0.025	ALDH2, SERPINA1
Monarch	EFO:0010156	Sweet liking measurement	2	9	2.19	0.66	0.025	ALDH2, SERPINA1
Monarch	HP:0002493	Upper motor neuron dysfunction	8	1100	0.71	0.42	0.025	EZR, GDI1, GFAP, HADHA, HSPD1, NNT, PSAT1, TPI1
Monarch	HP:0000119	Abnormality of the genitourinary system	11	2189	0.55	0.36	0.026	C3, GDI1, GSN, HADHA, HSPD1, NNT, PEPD, PLEC, PRDX1, PSAT1, SERPINA1
Monarch	HP:0011097	Epileptic spasm	4	182	1.19	0.54	0.029	ALDH7A1, EZR, PRDX1, PSAT1
Monarch	HP:0001252	Hypotonia	9	1505	0.62	0.37	0.035	ALDH7A1, EZR, GDI1, GFAP, HADHA, HSPD1, PRDX1, PSAT1, TPI1
Monarch	HP:0001635	Congestive heart failure	4	193	1.16	0.51	0.035	HADHA, SERPINA1, TPI1, VCL
Monarch	HP:0007067	Distal peripheral sensory neuropathy	2	12	2.07	0.59	0.037	GSN, HADHA
Monarch	HP:0100543	Cognitive impairment	10	1903	0.57	0.35	0.038	ALDH7A1, EZR, GDI1, GFAP, GSN, HADHA, HSPD1, NNT, PEPD, PRDX1

Table S6: Cerebellar vermis pathways enriched in upregulated proteins (FDR-adjusted $p < 0.05$) of children with idiopathic autism *(continued)*

Category	Term ID	Term description	Gene count	Back-ground	Strength	Signal	FDR	Matching proteins in network
Monarch	HP:0010628	Facial palsy	4	204	1.14	0.49	0.041	GDI1, GFAP, GSN, PLEC
Monarch	HP:0025142	Constitutional symptom	7	906	0.74	0.39	0.043	GDI1, GFAP, HADHA, HSPD1, NNT, PLEC, SERPINA1
Monarch	HP:0003110	Abnormality of urine homeostasis	6	638	0.82	0.41	0.046	GSN, HADHA, NNT, PEPD, PRDX1, SERPINA1
Monarch	HP:0030956	Abnormality of cardiovascular system electrophysiology	5	403	0.94	0.44	0.046	GFAP, GSN, HADHA, PLEC, SERPINA1
Monarch	HP:0002011	Morphological central nervous system abnormality	11	2416	0.51	0.31	0.048	ALDH2, ALDH7A1, EZR, GDI1, GFAP, HADHA, HSPD1, PLEC, PRDX1, PSAT1, TPI1
Monarch	HP:0011277	Abnormality of the urinary system physiology	7	933	0.72	0.38	0.048	C3, GSN, HADHA, NNT, PEPD, PRDX1, SERPINA1
Monarch	HP:0031956	Elevated circulating aspartate aminotransferase concentration	2	15	1.97	0.54	0.048	HADHA, PEPD
Monarch	HP:0045045	Elevated circulating acylcarnitine concentration	2	15	1.97	0.54	0.048	HADHA, PRDX1
Monarch	HP:0100626	Chronic hepatic failure	2	15	1.97	0.54	0.048	HADHA, SERPINA1
DISEASES	DOID:0050637	Finnish type amyloidosis	3	9	2.37	1.06	2.6e-03	C3, GSN, SERPINA1
DISEASES	DOID:0050639	Primary cutaneous amyloidosis	3	19	2.05	0.83	8.9e-03	C3, GSN, SERPINA1

Table S6: Cerebellar vermis pathways enriched in upregulated proteins (FDR-adjusted $p < 0.05$) of children with idiopathic autism *(continued)*

Category	Term ID	Term description	Gene count	Back-ground	Strength	Signal	FDR	Matching proteins in network
TISSUES	BTO:0000345	Digestive gland	25	2881	0.79	1.10	7.4e-15	AHNAK, ALDH1L1, ALDH2, ALDH7A1, ANXA5, C3, DPYSL3, ENO1, GDI1, GLO1, GSN, HADHA, HSPA1B, HSPD1, MACROH2A1, NNT, NPEPPS, PEPD, PHB2, PLEC, PRDX1, RDX, SERPINA1, TPI1, VCL
TISSUES	BTO:0000759	Liver	23	2125	0.88	1.31	7.4e-15	AHNAK, ALDH1L1, ALDH2, ALDH7A1, ANXA5, C3, ENO1, GLO1, GSN, HADHA, HSPA1B, HSPD1, MACROH2A1, NNT, NPEPPS, PEPD, PHB2, PLEC, PRDX1, RDX, SERPINA1, TPI1, VCL
TISSUES	BTO:0001491	Viscus	27	5378	0.55	0.67	1.0e-11	AHNAK, ALDH1L1, ALDH2, ALDH7A1, ANXA5, C3, DPYSL3, ENO1, EZR, GDI1, GFAP, GLO1, GSN, HADHA, HSPA1B, HSPD1, MACROH2A1, NNT, NPEPPS, PEPD, PHB2, PLEC, PRDX1, RDX, SERPINA1, TPI1, VCL
TISSUES	BTO:0001703	Right atrium	8	61	1.97	3.65	2.6e-11	AHNAK, C3, HADHA, HSPD1, NNT, SERPINA1, TPI1, VCL
TISSUES	BTO:0001702	Left atrium	7	60	1.91	3.06	1.7e-09	AHNAK, C3, HADHA, NNT, SERPINA1, TPI1, VCL
TISSUES	BTO:0003914	Interstitial cell of Cajal	9	193	1.52	2.43	2.1e-09	ALDH2, DPYSL3, ENO1, GDI1, HSPA1B, HSPD1, PRDX1, SERPINA1, TPI1

Table S6: Cerebellar vermis pathways enriched in upregulated proteins (FDR-adjusted p < 0.05) of children with idiopathic autism *(continued)*

Category	Term ID	Term description	Gene count	Back-ground	Strength	Signal	FDR	Matching proteins in network
TISSUES	BTO:0000058	Alimentary canal	18	2021	0.80	1.00	2.4e-09	AHNAK, ALDH2, ANXA5, C3, DPYSL3, ENO1, EZR, GDI1, GLO1, GSN, HADHA, HSPA1B, HSPD1, PLEC, PRDX1, SERPINA1, TPI1, VCL
TISSUES	BTO:0000522	Gland	27	7004	0.43	0.51	3.5e-09	AHNAK, ALDH1L1, ALDH2, ALDH7A1, ANXA5, C3, DPYSL3, ENO1, EZR, GDI1, GFAP, GLO1, GSN, HADHA, HSPA1B, HSPD1, MACROH2A1, NNT, NPEPPS, PEPD, PHB2, PLEC, PRDX1, RDX, SERPINA1, TPI1, VCL
TISSUES	BTO:0000511	Gastrointestinal tract	17	1816	0.82	1.03	4.7e-09	AHNAK, ALDH2, ANXA5, C3, DPYSL3, ENO1, EZR, GDI1, GLO1, GSN, HADHA, HSPA1B, HSPD1, PRDX1, SERPINA1, TPI1, VCL
TISSUES	BTO:0001488	Endocrine gland	26	6403	0.46	0.53	7.6e-09	AHNAK, ALDH1L1, ALDH2, ALDH7A1, ANXA5, C3, DPYSL3, ENO1, GDI1, GFAP, GLO1, GSN, HADHA, HSPA1B, HSPD1, MACROH2A1, NNT, NPEPPS, PEPD, PHB2, PLEC, PRDX1, RDX, SERPINA1, TPI1, VCL
TISSUES	BTO:0001484	Nervous system	25	6016	0.47	0.54	2.9e-08	AHNAK, ALDH1L1, ALDH2, ALDH7A1, ANXA5, DPYSL3, ENO1, EZR, GDI1, GFAP, GLO1, GSN, HADHA, HSPA1B, HSPD1, MACROH2A1, NPEPPS, PEPD, PHB2, PLEC, PRDX1, PSAT1, RDX, SERPINA1, TPI1

Table S6: Cerebellar vermis pathways enriched in upregulated proteins (FDR-adjusted $p < 0.05$) of children with idiopathic autism *(continued)*

Category	Term ID	Term description	Gene count	Back-ground	Strength	Signal	FDR	Matching proteins in network
TISSUES	BTO:0000255	Brain cell line	8	188	1.48	2.12	3.7e-08	ENO1, EZR, GFAP, HSPA1B, HSPD1, PRDX1, TPI1, VCL
TISSUES	BTO:0000227	Central nervous system	24	5825	0.46	0.52	1.8e-07	AHNAK, ALDH1L1, ALDH2, ALDH7A1, ANXA5, DPYSL3, ENO1, EZR, GDI1, GFAP, GLO1, GSN, HADHA, HSPD1, MACROH2A1, NPEPPS, PEPD, PHB2, PLEC, PRDX1, PSAT1, RDX, SERPINA1, TPI1
TISSUES	BTO:0000570	Hematopoietic system	18	2755	0.66	0.75	2.1e-07	AHNAK, ALDH2, C3, ENO1, GDI1, GFAP, GLO1, GSN, HADHA, HSPD1, MACROH2A1, PHB2, PLEC, PRDX1, PSAT1, SERPINA1, TPI1, VCL
TISSUES	BTO:0001629	Left ventricle	6	78	1.73	2.25	2.5e-07	AHNAK, HADHA, NNT, SERPINA1, TPI1, VCL
TISSUES	BTO:0003099	Internal female genital organ	18	2804	0.65	0.73	2.5e-07	AHNAK, ALDH7A1, ANXA5, C3, ENO1, EZR, GLO1, GSN, HSPA1B, HSPD1, MACROH2A1, PEPD, PLEC, PRDX1, PSAT1, SERPINA1, TPI1, VCL
TISSUES	BTO:0000089	Blood	15	1824	0.76	0.86	4.6e-07	AHNAK, C3, ENO1, GFAP, GLO1, GSN, HSPD1, MACROH2A1, PHB2, PLEC, PRDX1, PSAT1, SERPINA1, TPI1, VCL

Table S6: Cerebellar vermis pathways enriched in upregulated proteins (FDR-adjusted $p < 0.05$) of children with idiopathic autism *(continued)*

Category	Term ID	Term description	Gene count	Back-ground	Strength	Signal	FDR	Matching proteins in network
TISSUES	BTO:0003091	Urogenital system	25	7090	0.39	0.45	8.7e-07	AHNAK, ALDH1L1, ALDH7A1, ANXA5, C3, DPYSL3, ENO1, EZR, GDI1, GFAP, GLO1, GSN, HADHA, HSPA1B, HSPD1, MACROH2A1, NPEPPS, PEPD, PHB2, PLEC, PRDX1, PSAT1, SERPINA1, TPI1, VCL
TISSUES	BTO:0000763	Lung	13	1395	0.82	0.91	1.8e-06	AHNAK, ANXA5, C3, DPYSL3, ENO1, EZR, GDI1, HSPD1, NPEPPS, PRDX1, PSAT1, SERPINA1, TPI1
TISSUES	BTO:0000282	Head	24	6642	0.41	0.46	2.2e-06	AHNAK, ALDH1L1, ALDH2, ALDH7A1, ANXA5, DPYSL3, ENO1, EZR, GDI1, GFAP, GLO1, GSN, HADHA, HSPD1, MACROH2A1, NPEPPS, PEPD, PHB2, PRDX1, PSAT1, RDX, SERPINA1, TPI1, VCL
TISSUES	BTO:0000775	Lymphocyte	10	698	1.00	1.15	2.5e-06	AHNAK, ENO1, HSPD1, PHB2, PLEC, PRDX1, PSAT1, SERPINA1, TPI1, VCL
TISSUES	BTO:0000083	Female reproductive system	23	6111	0.42	0.47	3.3e-06	AHNAK, ALDH7A1, ANXA5, C3, DPYSL3, ENO1, EZR, GDI1, GLO1, GSN, HADHA, HSPA1B, HSPD1, MACROH2A1, NPEPPS, PEPD, PHB2, PLEC, PRDX1, PSAT1, SERPINA1, TPI1, VCL
TISSUES	BTO:0000284	Organism form	16	2542	0.65	0.69	3.6e-06	AHNAK, ALDH7A1, ANXA5, ENO1, EZR, GFAP, HSPA1B, HSPD1, MACROH2A1, PEPD, PLEC, PRDX1, PSAT1, SERPINA1, TPI1, VCL

Table S6: Cerebellar vermis pathways enriched in upregulated proteins (FDR-adjusted $p < 0.05$) of children with idiopathic autism *(continued)*

Category	Term ID	Term description	Gene count	Back-ground	Strength	Signal	FDR	Matching proteins in network
TISSUES	BTO:0003092	Urinary system	12	1249	0.83	0.90	4.5e-06	AHNAK, ALDH1L1, ALDH7A1, ENO1, EZR, GFAP, GSN, HSPD1, PEPD, PRDX1, TPI1, VCL
TISSUES	BTO:0001244	Urinary tract	12	1258	0.83	0.89	4.7e-06	AHNAK, ALDH1L1, ALDH7A1, ENO1, EZR, GFAP, GSN, HSPD1, PEPD, PRDX1, TPI1, VCL
TISSUES	BTO:0000574	Hematopoietic cell	11	1019	0.88	0.95	5.7e-06	AHNAK, ENO1, GLO1, HSPD1, PHB2, PLEC, PRDX1, PSAT1, SERPINA1, TPI1, VCL
TISSUES	BTO:0000142	Brain	22	5733	0.43	0.47	6.9e-06	AHNAK, ALDH1L1, ALDH2, ALDH7A1, ANXA5, DPYSL3, ENO1, EZR, GDI1, GFAP, GLO1, GSN, HADHA, HSPD1, MACROH2A1, NPEPPS, PEPD, PHB2, PRDX1, PSAT1, RDX, TPI1
TISSUES	BTO:0000174	Embryonic structure	15	2369	0.65	0.67	9.6e-06	AHNAK, ALDH7A1, ANXA5, ENO1, EZR, GFAP, HSPA1B, HSPD1, MACROH2A1, PEPD, PLEC, PRDX1, SERPINA1, TPI1, VCL
TISSUES	BTO:0000421	Connective tissue	10	954	0.87	0.88	2.9e-05	ALDH2, ANXA5, DPYSL3, ENO1, GDI1, HSPA1B, HSPD1, PRDX1, SERPINA1, TPI1
TISSUES	BTO:0000975	Ovary	8	528	1.03	1.04	3.6e-05	AHNAK, ANXA5, C3, ENO1, MACROH2A1, SERPINA1, TPI1, VCL
TISSUES	BTO:0000088	Cardiovascular system	10	1057	0.82	0.80	7.0e-05	AHNAK, C3, ENO1, GSN, HADHA, HSPD1, NNT, SERPINA1, TPI1, VCL

Table S6: Cerebellar vermis pathways enriched in upregulated proteins (FDR-adjusted $p < 0.05$) of children with idiopathic autism *(continued)*

Category	Term ID	Term description	Gene count	Back-ground	Strength	Signal	FDR	Matching proteins in network
TISSUES	BTO:0000648	Intestine	11	1349	0.76	0.73	7.1e-05	AHNAK, C3, ENO1, EZR, GLO1, GSN, HADHA, HSPD1, SERPINA1, TPI1, VCL
TISSUES	BTO:0005810	Immune system	12	1664	0.71	0.68	7.1e-05	AHNAK, ENO1, GDI1, GFAP, HSPD1, PHB2, PLEC, PRDX1, PSAT1, SERPINA1, TPI1, VCL
TISSUES	BTO:0000439	Eye	9	835	0.88	0.84	9.0e-05	ENO1, GDI1, GFAP, GLO1, GSN, PRDX1, RDX, TPI1, VCL
TISSUES	BTO:0000202	Sense organ	10	1124	0.80	0.76	1.1e-04	ENO1, GDI1, GFAP, GLO1, GSN, NPEPPS, PRDX1, RDX, TPI1, VCL
TISSUES	BTO:0000634	Integument	13	2112	0.64	0.61	1.1e-04	ANXA5, ENO1, EZR, GDI1, GFAP, GSN, HSPA1B, HSPD1, PEPD, PHB2, PRDX1, TPI1, VCL
TISSUES	BTO:0001253	Skin	10	1151	0.79	0.74	1.3e-04	ANXA5, ENO1, GDI1, GSN, HSPA1B, HSPD1, PEPD, PHB2, PRDX1, TPI1
TISSUES	BTO:0000586	Colonic cancer cell	6	280	1.18	1.09	1.4e-04	ENO1, HSPA1B, HSPD1, PHB2, PLEC, TPI1
TISSUES	BTO:0001078	Placenta	10	1244	0.75	0.69	2.4e-04	AHNAK, ALDH7A1, ANXA5, ENO1, EZR, MACROH2A1, PEPD, PLEC, PRDX1, SERPINA1
TISSUES	BTO:0000753	Lymphoid tissue	11	1600	0.68	0.62	3.1e-04	AHNAK, ENO1, GDI1, HSPD1, PHB2, PLEC, PRDX1, PSAT1, SERPINA1, TPI1, VCL
TISSUES	BTO:0001271	Leukemia cell	9	1067	0.77	0.67	5.2e-04	ANXA5, ENO1, HSPA1B, HSPD1, MACROH2A1, PHB2, PRDX1, TPI1, VCL

Table S6: Cerebellar vermis pathways enriched in upregulated proteins (FDR-adjusted $p < 0.05$) of children with idiopathic autism *(continued)*

Category	Term ID	Term description	Gene count	Back-ground	Strength	Signal	FDR	Matching proteins in network
TISSUES	BTO:0001489	Whole body	28	13099	0.18	0.27	5.2e-04	AHNAK, ALDH1L1, ALDH2, ALDH7A1, ANXA5, C3, DPYSL3, ENO1, EZR, GDI1, GFAP, GLO1, GSN, HADHA, HSPA1B, HSPD1, MACROH2A1, NNT, NPEPPS, PEPD, PHB2, PLEC, PRDX1, PSAT1, RDX, SERPINA1, TPI1, VCL
TISSUES	BTO:0000443	Adipocyte	3	42	1.70	1.06	1.6e-03	ALDH2, ANXA5, HSPD1
TISSUES	BTO:0001490	Other source	3	43	1.69	1.06	1.6e-03	AHNAK, PLEC, VCL
TISSUES	BTO:0000706	Large intestine	7	750	0.82	0.61	2.9e-03	AHNAK, C3, EZR, GLO1, GSN, SERPINA1, VCL
TISSUES	BTO:0000445	Cerebral lobe	7	759	0.81	0.61	3.0e-03	AHNAK, ALDH1L1, ALDH7A1, GFAP, GSN, HADHA, RDX
TISSUES	BTO:0000671	Kidney	8	1039	0.73	0.56	3.0e-03	ALDH7A1, ENO1, EZR, GFAP, GSN, HSPD1, PEPD, TPI1
TISSUES	BTO:0001175	Retina	5	322	1.04	0.73	3.4e-03	ENO1, GDI1, GFAP, RDX, VCL
TISSUES	BTO:0000091	Ascites	3	61	1.54	0.89	3.9e-03	ENO1, HSPD1, SERPINA1
TISSUES	BTO:0001487	Adipose tissue	4	176	1.20	0.77	4.4e-03	ALDH2, ANXA5, ENO1, HSPD1
TISSUES	BTO:0001424	Uterus	8	1117	0.70	0.52	4.5e-03	ALDH7A1, ANXA5, ENO1, GLO1, GSN, HSPD1, PEPD, VCL
TISSUES	BTO:0000545	Gut	3	69	1.49	0.84	5.3e-03	AHNAK, PLEC, VCL
TISSUES	BTO:0000132	Blood platelet	5	363	0.99	0.66	5.5e-03	C3, GSN, HSPD1, SERPINA1, VCL
TISSUES	BTO:0000988	Pancreas	6	626	0.83	0.56	7.8e-03	AHNAK, ENO1, GSN, HSPA1B, PRDX1, TPI1
TISSUES	BTO:0001485	Muscular system	8	1220	0.66	0.47	7.8e-03	ALDH2, ANXA5, ENO1, HSPA1B, NNT, NPEPPS, SERPINA1, TPI1

Table S6: Cerebellar vermis pathways enriched in upregulated proteins (FDR-adjusted $p < 0.05$) of children with idiopathic autism *(continued)*

Category	Term ID	Term description	Gene count	Back-ground	Strength	Signal	FDR	Matching proteins in network
TISSUES	BTO:0001355	Temporal lobe	6	633	0.82	0.55	8.0e-03	ALDH1L1, ALDH7A1, GFAP, GSN, HADHA, RDX
TISSUES	BTO:0000269	Colon	6	639	0.82	0.55	8.3e-03	AHNAK, EZR, GLO1, GSN, SERPINA1, VCL
TISSUES	BTO:0001418	Urinary bladder	4	217	1.11	0.67	8.6e-03	AHNAK, ALDH1L1, PRDX1, VCL
TISSUES	BTO:0001279	Spinal cord	4	233	1.08	0.63	0.011	AHNAK, GFAP, PLEC, SERPINA1
TISSUES	BTO:0000583	Bone marrow cancer cell	5	442	0.90	0.55	0.012	ANXA5, ENO1, HSPA1B, PRDX1, VCL
TISSUES	BTO:0000772	Lymphoblast	4	240	1.07	0.62	0.012	HSPD1, PHB2, PRDX1, PSAT1
TISSUES	BTO:0000254	Female reproductive gland	12	3017	0.45	0.34	0.014	AHNAK, ANXA5, C3, ENO1, GSN, HADHA, HSPD1, MACROH2A1, PRDX1, SERPINA1, TPI1, VCL
TISSUES	BTO:0001129	Prostate gland	5	476	0.87	0.52	0.016	AHNAK, ENO1, PRDX1, TPI1, VCL
TISSUES	BTO:0000887	Muscle	7	1070	0.66	0.43	0.018	ALDH2, ANXA5, ENO1, HSPA1B, NNT, NPEPPS, TPI1
TISSUES	BTO:0000080	Male reproductive gland	11	2658	0.46	0.34	0.019	AHNAK, ANXA5, ENO1, GDI1, GLO1, GSN, HADHA, HSPD1, PRDX1, TPI1, VCL
TISSUES	BTO:0000782	T-lymphocyte	4	279	1.00	0.55	0.019	AHNAK, ENO1, PLEC, TPI1
TISSUES	BTO:0000601	Hippocampus	4	299	0.97	0.52	0.023	ALDH7A1, GFAP, GSN, RDX
TISSUES	BTO:0000928	Limbic system	6	811	0.72	0.43	0.023	ALDH1L1, ALDH7A1, GFAP, GSN, HADHA, RDX
TISSUES	BTO:0000379	Embryo	6	824	0.71	0.42	0.025	AHNAK, GFAP, HSPA1B, PLEC, TPI1, VCL
TISSUES	BTO:0000180	Cervical carcinoma cell	4	322	0.94	0.48	0.029	ANXA5, ENO1, EZR, VCL
TISSUES	BTO:0000493	Gall bladder	2	42	1.53	0.53	0.045	AHNAK, VCL
TISSUES	BTO:0001486	Skeletal system	7	1307	0.58	0.33	0.049	C3, ENO1, MACROH2A1, NNT, NPEPPS, SERPINA1, TPI1

Table S6: Cerebellar vermis pathways enriched in upregulated proteins (FDR-adjusted $p < 0.05$) of children with idiopathic autism *(continued)*

Category	Term ID	Term description	Gene count	Back-ground	Strength	Signal	FDR	Matching proteins in network
COMPARTMENTS	GOCC:0065010	Extracellular membrane-bounded organelle	12	473	1.25	1.90	2.1e-09	AHNAK, ANXA5, C3, ENO1, EZR, GSN, HSPA1B, HSPD1, PRDX1, RDX, SERPINA1, VCL
COMPARTMENTS	GOCC:1903561	Extracellular vesicle	12	500	1.23	1.85	2.1e-09	AHNAK, ANXA5, C3, ENO1, EZR, GSN, HSPA1B, HSPD1, PRDX1, RDX, SERPINA1, VCL
COMPARTMENTS	GOCC:0070062	Extracellular exosome	11	428	1.26	1.86	5.1e-09	AHNAK, ANXA5, C3, ENO1, EZR, GSN, HSPA1B, HSPD1, PRDX1, SERPINA1, VCL
COMPARTMENTS	GOCC:0005737	Cytoplasm	27	8195	0.37	0.43	4.1e-07	AHNAK, ALDH1L1, ALDH2, ALDH7A1, ANXA5, C3, DPYSL3, ENO1, EZR, GDI1, GFAP, GLO1, GSN, HADHA, HSPA1B, HSPD1, NNT, NPEPPS, PEPD, PHB2, PLEC, PRDX1, PSAT1, RDX, SERPINA1, TPI1, VCL
COMPARTMENTS	GOCC:0005925	Focal adhesion	8	269	1.32	1.61	1.3e-06	AHNAK, ANXA5, EZR, GSN, HSPA1B, PLEC, RDX, VCL
COMPARTMENTS	GOCC:0005829	Cytosol	17	3054	0.59	0.60	1.9e-05	AHNAK, ALDH1L1, ALDH7A1, ANXA5, DPYSL3, ENO1, EZR, GDI1, GFAP, GLO1, GSN, HSPA1B, HSPD1, PLEC, PSAT1, TPI1, VCL
COMPARTMENTS	GOCC:0005622	Intracellular	28	11512	0.23	0.31	6.9e-05	AHNAK, ALDH1L1, ALDH2, ALDH7A1, ANXA5, C3, DPYSL3, ENO1, EZR, GDI1, GFAP, GLO1, GSN, HADHA, HSPA1B, HSPD1, MACROH2A1, NNT, NPEPPS, PEPD, PHB2, PLEC, PRDX1, PSAT1, RDX, SERPINA1, TPI1, VCL
COMPARTMENTS	GOCC:0043034	Costamere	3	13	2.21	1.44	2.7e-04	AHNAK, PLEC, VCL

Table S6: Cerebellar vermis pathways enriched in upregulated proteins (FDR-adjusted p < 0.05) of children with idiopathic autism *(continued)*

Category	Term ID	Term description	Gene count	Back-ground	Strength	Signal	FDR	Matching proteins in network
COMPARTMENTS	GOCC:0031982	Vesicle	13	2125	0.63	0.57	3.8e-04	AHNAK, ANXA5, C3, ENO1, EZR, GSN, HSPA1B, HSPD1, PRDX1, RDX, SERPINA1, TPI1, VCL
COMPARTMENTS	GOCC:0030863	Cortical cytoskeleton	4	79	1.55	1.10	9.1e-04	EZR, GSN, RDX, VCL
COMPARTMENTS	GOCC:0005938	Cell cortex	5	190	1.27	0.95	1.1e-03	ENO1, EZR, GSN, RDX, VCL
COMPARTMENTS	GOCC:0015629	Actin cytoskeleton	6	363	1.07	0.81	1.6e-03	AHNAK, DPYSL3, EZR, GSN, RDX, VCL
COMPARTMENTS	GOCC:0043226	Organelle	25	10113	0.24	0.28	3.2e-03	AHNAK, ALDH2, ALDH7A1, ANXA5, C3, DPYSL3, ENO1, EZR, GFAP, GLO1, GSN, HADHA, HSPA1B, HSPD1, MACROH2A1, NNT, NPEPPS, PEPD, PHB2, PLEC, PRDX1, RDX, SERPINA1, TPI1, VCL
COMPARTMENTS	GOCC:0072562	Blood microparticle	4	118	1.38	0.87	3.2e-03	ANXA5, C3, GSN, HSPA1B
COMPARTMENTS	GOCC:1904813	ficolin-1-rich granule lumen	4	124	1.36	0.86	3.5e-03	GSN, HSPA1B, SERPINA1, VCL
COMPARTMENTS	GOCC:0000015	Phosphopyruvate hydratase complex	2	5	2.45	0.98	4.2e-03	ENO1, TPI1
COMPARTMENTS	GOCC:0030864	Cortical actin cytoskeleton	3	54	1.59	0.82	7.1e-03	GSN, RDX, VCL
COMPARTMENTS	GOCC:0110165	Cellular anatomical entity	28	14060	0.15	0.23	7.7e-03	AHNAK, ALDH1L1, ALDH2, ALDH7A1, ANXA5, C3, DPYSL3, ENO1, EZR, GDI1, GFAP, GLO1, GSN, HADHA, HSPA1B, HSPD1, MACROH2A1, NNT, NPEPPS, PEPD, PHB2, PLEC, PRDX1, PSAT1, RDX, SERPINA1, TPI1, VCL
COMPARTMENTS	GOCC:0099512	Supramolecular fiber	6	555	0.88	0.56	0.010	AHNAK, DPYSL3, EZR, GFAP, PLEC, VCL
COMPARTMENTS	GOCC:0042383	Sarcolemma	3	68	1.49	0.73	0.011	AHNAK, PLEC, VCL

Table S6: Cerebellar vermis pathways enriched in upregulated proteins (FDR-adjusted $p < 0.05$) of children with idiopathic autism *(continued)*

Category	Term ID	Term description	Gene count	Back-ground	Strength	Signal	FDR	Matching proteins in network
COMPARTMENTS	GOCC:0005856	Cytoskeleton	9	1575	0.60	0.40	0.018	AHNAK, DPYSL3, EZR, GFAP, GSN, HSPA1B, PLEC, RDX, VCL
COMPARTMENTS	GOCC:0043229	Intracellular organelle	23	9609	0.23	0.24	0.022	AHNAK, ALDH2, ALDH7A1, C3, DPYSL3, ENO1, EZR, GFAP, GLO1, GSN, HADHA, HSPA1B, HSPD1, MACROH2A1, NNT, PEPD, PHB2, PLEC, PRDX1, RDX, SERPINA1, TPI1, VCL
COMPARTMENTS	GOCC:0070013	Intracellular organelle lumen	12	2902	0.46	0.33	0.024	ALDH2, ALDH7A1, C3, EZR, GSN, HADHA, HSPA1B, HSPD1, MACROH2A1, PHB2, SERPINA1, VCL
COMPARTMENTS	GOCC:0034774	Secretory granule lumen	4	241	1.07	0.53	0.025	C3, GSN, SERPINA1, VCL
COMPARTMENTS	GOCC:0043227	Membrane-bounded organelle	22	9083	0.23	0.24	0.029	AHNAK, ALDH2, ALDH7A1, ANXA5, C3, ENO1, EZR, GLO1, GSN, HADHA, HSPA1B, HSPD1, MACROH2A1, NNT, NPEPPS, PEPD, PHB2, PRDX1, RDX, SERPINA1, TPI1, VCL
COMPARTMENTS	GOCC:0002102	Podosome	2	25	1.75	0.56	0.040	GSN, VCL
COMPARTMENTS	GOCC:0032991	Protein-containing complex	16	5325	0.33	0.26	0.045	ALDH7A1, ANXA5, C3, DPYSL3, ENO1, EZR, HADHA, HSPA1B, HSPD1, MACROH2A1, PEPD, PHB2, PLEC, SERPINA1, TPI1, VCL

Table S6: Cerebellar vermis pathways enriched in upregulated proteins (FDR-adjusted p < 0.05) of children with idiopathic autism *(continued)*

Category	Term ID	Term description	Gene count	Back-ground	Strength	Signal	FDR	Matching proteins in network
UniProt Keywords	KW-0007	Acetylation	21	3362	0.64	0.75	1.8e-08	AHNAK, ALDH1L1, ALDH2, ALDH7A1, ANXA5, ENO1, EZR, GLO1, GSN, HADHA, HSPA1B, HSPD1, MACROH2A1, NNT, PEPD, PHB2, PLEC, PRDX1, PSAT1, TPI1, VCL
UniProt Keywords	KW-0597	Phosphoprotein	23	8122	0.30	0.31	3.9e-03	AHNAK, ALDH1L1, ANXA5, C3, DPYSL3, ENO1, EZR, GFAP, GLO1, GSN, HADHA, HSPA1B, HSPD1, MACROH2A1, PEPD, PHB2, PLEC, PRDX1, PSAT1, RDX, SERPINA1, TPI1, VCL
UniProt Keywords	KW-0456	Lyase	4	157	1.25	0.63	0.017	ENO1, GLO1, HADHA, TPI1
UniProt Keywords	KW-0520	NAD	4	184	1.18	0.57	0.023	ALDH2, ALDH7A1, HADHA, NNT
UniProt Keywords	KW-0117	Actin capping	2	19	1.87	0.54	0.046	GSN, RDX
UniProt Keywords	KW-0488	Methylation	7	973	0.70	0.37	0.046	AHNAK, GFAP, HADHA, HSPA1B, MACROH2A1, PLEC, TPI1
UniProt Keywords	KW-0963	Cytoplasm	16	5095	0.34	0.26	0.046	ALDH1L1, ALDH7A1, DPYSL3, ENO1, EZR, GDI1, GFAP, GSN, HSPA1B, NPEPPS, PHB2, PLEC, PRDX1, RDX, TPI1, VCL
UniProt Keywords	KW-0009	Actin-binding	4	271	1.02	0.44	0.049	GSN, PLEC, RDX, VCL
Pfam	PF00171	Aldehyde dehydrogenase family	3	20	2.02	0.81	0.010	ALDH1L1, ALDH2, ALDH7A1
InterPro	IPR015590	Aldehyde dehydrogenase domain	3	20	2.02	0.71	0.018	ALDH1L1, ALDH2, ALDH7A1

Table S6: Cerebellar vermis pathways enriched in upregulated proteins (FDR-adjusted $p < 0.05$) of children with idiopathic autism *(continued)*

Category	Term ID	Term description	Gene count	Back-ground	Strength	Signal	FDR	Matching proteins in network
InterPro	IPR016161	Aldehyde/histidinol dehydrogenase	3	20	2.02	0.71	0.018	ALDH1L1, ALDH2, ALDH7A1
InterPro	IPR016162	Aldehyde dehydrogenase, N-terminal	3	20	2.02	0.71	0.018	ALDH1L1, ALDH2, ALDH7A1
InterPro	IPR016163	Aldehyde dehydrogenase, C-terminal	3	19	2.05	0.72	0.018	ALDH1L1, ALDH2, ALDH7A1
InterPro	IPR029510	Aldehyde dehydrogenase, glutamic acid active site	3	16	2.12	0.72	0.018	ALDH1L1, ALDH2, ALDH7A1
InterPro	IPR008954	Moesin tail domain superfamily	2	5	2.45	0.61	0.035	EZR, RDX
InterPro	IPR011174	Ezrin/radixin/moesin	2	4	2.55	0.61	0.035	EZR, RDX
InterPro	IPR011259	Ezrin/radixin/moesin, C-terminal	2	4	2.55	0.61	0.035	EZR, RDX
InterPro	IPR041789	ERM family, FERM domain C-lobe	2	4	2.55	0.61	0.035	EZR, RDX
InterPro	IPR046810	Ezrin/radixin/moesin, alpha-helical domain	2	4	2.55	0.61	0.035	EZR, RDX

Table S7: Cerebellar vermis pathways enriched in downregulated proteins (FDR-adjusted $p < 0.05$) of adults with idiopathic autism

Category	Term ID	Term description	Gene count	Back-ground	Strength	Signal	FDR	Matching proteins in network
GO Process	GO:0051641	Cellular localization	23	2677	0.63	0.72	1.6e-06	ANK2, AP3B2, DNM1, DNM3, DYNC1H1, GRID2, HK1, HSP90AA1, ITSN1, MACF1, MAP1B, NSF, SEPTIN11, SEPTIN3, SEPTIN4, SEPTIN6, SEPTIN9, SPTBN1, STXBP1, SYN1, SYN2, SYNE1, XRCC5
GO Process	GO:0099504	Synaptic vesicle cycle	7	125	1.44	1.30	6.2e-05	AP3B2, DNM1, DNM3, ITSN1, STXBP1, SYN1, SYN2
GO Process	GO:0008104	Protein localization	17	1943	0.63	0.60	3.1e-04	ANK2, AP3B2, GRID2, HK1, HSP90AA1, ITSN1, MACF1, NSF, SEPTIN11, SEPTIN3, SEPTIN4, SEPTIN6, SEPTIN9, SPTBN1, STXBP1, SYNE1, XRCC5
GO Process	GO:0051179	Localization	25	4512	0.44	0.46	3.1e-04	AFG3L2, ANK2, AP3B2, DNM1, DNM3, DYNC1H1, GRID2, HK1, HSP90AA1, ITSN1, MACF1, MAP1B, NSF, SEPTIN11, SEPTIN3, SEPTIN4, SEPTIN6, SEPTIN9, SGIP1, SPTBN1, STXBP1, SYN1, SYN2, SYNE1, XRCC5
GO Process	GO:0061640	Cytoskeleton-dependent cytokinesis	6	112	1.42	1.11	3.1e-04	SEPTIN11, SEPTIN3, SEPTIN4, SEPTIN6, SEPTIN9, SPTBN1
GO Process	GO:0097479	Synaptic vesicle localization	4	40	1.69	0.90	2.9e-03	AP3B2, DNM1, SYN1, SYN2
GO Process	GO:0044087	Regulation of cellular component biogenesis	11	971	0.75	0.56	3.8e-03	CEP76, DNM3, DYNC1H1, GRID2, HSP90AA1, MACF1, MAP1B, SEPTIN9, SPTBN1, STXBP1, XRCC5

Table S7: Cerebellar vermis pathways enriched in downregulated proteins (FDR-adjusted $p < 0.05$) of adults with idiopathic autism *(continued)*

Category	Term ID	Term description	Gene count	Back-ground	Strength	Signal	FDR	Matching proteins in network
GO Process	GO:0016043	Cellular component organization	25	5436	0.36	0.34	5.7e-03	AFG3L2, ANK2, AP3B2, DNM1, DNM3, DYNC1H1, FARSB, GRID2, HSP90AA1, KRAS, MACF1, MAP1B, MPP6, NSF, SEPTIN6, SGIP1, SPTBN1, STXBP1, SYN1, SYNE1, TUBB2A, TUBB3, WDR1, XRCC5, XRCC6
GO Process	GO:0016192	Vesicle-mediated transport	12	1298	0.66	0.47	7.8e-03	ANK2, AP3B2, DNM1, DNM3, ITSN1, MACF1, NSF, SGIP1, SPTBN1, STXBP1, SYN1, SYN2
GO Process	GO:0048488	Synaptic vesicle endocytosis	4	56	1.55	0.74	7.8e-03	AP3B2, DNM1, DNM3, ITSN1
GO Process	GO:0002218	Activation of innate immune response	4	65	1.48	0.69	0.011	HSP90AA1, PSPC1, XRCC5, XRCC6
GO Process	GO:0007399	Nervous system development	15	2188	0.53	0.40	0.011	AFG3L2, ANK2, DNM3, GRID2, HSP90AA1, KRAS, MAP1B, SEPTIN4, SPTBN1, STXBP1, SYN1, TAGLN3, TUBB2A, TUBB3, XRCC5
GO Process	GO:0051648	Vesicle localization	5	180	1.14	0.51	0.027	AP3B2, DNM1, DYNC1H1, SYN1, SYN2
GO Process	GO:0022008	Neurogenesis	11	1290	0.62	0.38	0.027	AFG3L2, GRID2, HSP90AA1, KRAS, MAP1B, SEPTIN4, STXBP1, SYN1, TUBB2A, TUBB3, XRCC5
GO Process	GO:0051130	Positive regulation of cellular component organization	10	1049	0.67	0.40	0.027	DNM3, DYNC1H1, GRID2, HSP90AA1, MACF1, MAP1B, SEPTIN9, SGIP1, WDR1, XRCC5
GO Process	GO:0043001	Golgi to plasma membrane protein transport	3	30	1.69	0.57	0.028	MACF1, NSF, SPTBN1

Table S7: Cerebellar vermis pathways enriched in downregulated proteins (FDR-adjusted $p < 0.05$) of adults with idiopathic autism *(continued)*

Category	Term ID	Term description	Gene count	Back-ground	Strength	Signal	FDR	Matching proteins in network
GO Process	GO:0006996	Organelle organization	18	3470	0.41	0.30	0.036	AFG3L2, ANK2, AP3B2, DNM1, DYNC1H1, HSP90AA1, KRAS, MACF1, MAP1B, SEPTIN6, SPTBN1, STXBP1, SYNE1, TUBB2A, TUBB3, WDR1, XRCC5, XRCC6
GO Process	GO:0022607	Cellular component assembly	15	2467	0.48	0.32	0.036	AFG3L2, ANK2, DNM3, DYNC1H1, FARSB, GRID2, HSP90AA1, MAP1B, MPP6, SEPTIN6, SGIP1, SPTBN1, STXBP1, WDR1, XRCC5
GO Process	GO:0034330	Cell junction organization	7	492	0.85	0.42	0.036	AFG3L2, ANK2, DNM3, GRID2, MAP1B, SYN1, WDR1
GO Process	GO:0072659	Protein localization to plasma membrane	5	200	1.09	0.47	0.036	ANK2, MACF1, NSF, SPTBN1, STXBP1
GO Process	GO:0051640	Organelle localization	7	514	0.83	0.40	0.042	AP3B2, DNM1, DYNC1H1, MAP1B, STXBP1, SYN1, SYN2
GO Process	GO:0051301	Cell division	7	527	0.82	0.39	0.047	DYNC1H1, SEPTIN11, SEPTIN3, SEPTIN4, SEPTIN6, SEPTIN9, SPTBN1
GO Function	GO:0035639	Purine ribonucleoside triphosphate binding	21	1834	0.75	0.96	2.7e-08	AFG3L2, DNM1, DNM3, DYNC1H1, EEF1A2, FARSB, HK1, HSP90AA1, KRAS, NSF, SEPTIN11, SEPTIN3, SEPTIN4, SEPTIN6, SEPTIN9, SYN1, SYN2, TUBB2A, TUBB3, XRCC5, XRCC6
GO Function	GO:0005525	GTP binding	12	381	1.19	1.66	2.7e-08	DNM1, DNM3, EEF1A2, HSP90AA1, KRAS, SEPTIN11, SEPTIN3, SEPTIN4, SEPTIN6, SEPTIN9, TUBB2A, TUBB3

Table S7: Cerebellar vermis pathways enriched in downregulated proteins (FDR-adjusted $p < 0.05$) of adults with idiopathic autism *(continued)*

Category	Term ID	Term description	Gene count	Back-ground	Strength	Signal	FDR	Matching proteins in network
GO Function	GO:0032555	Purine ribonucleotide binding	21	1903	0.74	0.93	2.7e-08	AFG3L2, DNM1, DNM3, DYNC1H1, EEF1A2, FARSB, HK1, HSP90AA1, KRAS, NSF, SEPTIN11, SEPTIN3, SEPTIN4, SEPTIN6, SEPTIN9, SYN1, SYN2, TUBB2A, TUBB3, XRCC5, XRCC6
GO Function	GO:0017111	Nucleoside-triphosphatase activity	12	650	0.96	1.13	1.6e-06	AFG3L2, DNM1, DNM3, EEF1A2, HSP90AA1, KRAS, NSF, SEPTIN11, SEPTIN3, SEPTIN4, SEPTIN6, SEPTIN9
GO Function	GO:0003924	GTPase activity	9	317	1.15	1.30	4.1e-06	DNM1, DNM3, EEF1A2, KRAS, SEPTIN11, SEPTIN3, SEPTIN4, SEPTIN6, SEPTIN9
GO Function	GO:0060090	Molecular adaptor activity	9	385	1.06	1.11	2.0e-05	ANK2, ITSN1, SEPTIN11, SEPTIN3, SEPTIN4, SEPTIN6, SEPTIN9, SYN1, SYNE1
GO Function	GO:1901363	Heterocyclic compound binding	27	5977	0.35	0.39	3.8e-04	AFG3L2, DNM1, DNM3, DYNC1H1, EEF1A2, FARSB, HK1, HSP90AA1, KRAS, MACF1, MDH2, NSF, PSPC1, SEPTIN11, SEPTIN3, SEPTIN4, SEPTIN6, SEPTIN9, SPTBN1, STXBP1, SYN1, SYN2, SYNE1, TUBB2A, TUBB3, XRCC5, XRCC6

Table S7: Cerebellar vermis pathways enriched in downregulated proteins (FDR-adjusted $p < 0.05$) of adults with idiopathic autism *(continued)*

Category	Term ID	Term description	Gene count	Back-ground	Strength	Signal	FDR	Matching proteins in network
GO Function	GO:0097159	Organic cyclic compound binding	27	6050	0.34	0.39	4.6e-04	AFG3L2, DNM1, DNM3, DYNC1H1, EEF1A2, FARSB, HK1, HSP90AA1, KRAS, MACF1, MDH2, NSF, PSPC1, SEPTIN11, SEPTIN3, SEPTIN4, SEPTIN6, SEPTIN9, SPTBN1, STXBP1, SYN1, SYN2, SYNE1, TUBB2A, TUBB3, XRCC5, XRCC6
GO Function	GO:0008092	Cytoskeletal protein binding	11	1002	0.73	0.63	8.5e-04	ANK2, DNM1, DNM3, HSP90AA1, MACF1, MAP1B, SGIP1, SPTBN1, SYN1, SYNE1, WDR1
GO Function	GO:0005515	Protein binding	28	7242	0.28	0.31	4.6e-03	AFG3L2, ANK2, DNM1, DNM3, DYNC1H1, EEF1A2, GRID2, HSP90AA1, ITSN1, KRAS, MACF1, MAP1B, MDH2, MPP1, NSF, SEPTIN3, SEPTIN4, SEPTIN9, SGIP1, SPTBN1, STXBP1, SYN1, SYN2, SYNE1, TUBB3, WDR1, XRCC5, XRCC6
GO Function	GO:0016787	Hydrolase activity	15	2347	0.50	0.41	6.5e-03	AFG3L2, DNM1, DNM3, EEF1A2, HSP90AA1, KRAS, LRRC40, NSF, SEPTIN11, SEPTIN3, SEPTIN4, SEPTIN6, SEPTIN9, XRCC5, XRCC6
GO Function	GO:0044877	Protein-containing complex binding	11	1261	0.63	0.47	6.5e-03	ANK2, HSP90AA1, KRAS, MACF1, MAP1B, NSF, SPTBN1, SYNE1, WDR1, XRCC5, XRCC6
GO Function	GO:0005200	Structural constituent of cytoskeleton	4	107	1.27	0.61	0.015	ANK2, SPTBN1, TUBB2A, TUBB3

Table S7: Cerebellar vermis pathways enriched in downregulated proteins (FDR-adjusted $p < 0.05$) of adults with idiopathic autism *(continued)*

Category	Term ID	Term description	Gene count	Back-ground	Strength	Signal	FDR	Matching proteins in network
GO Function	GO:0003723	RNA binding	12	1672	0.55	0.39	0.016	DNM1, DYNC1H1, FARSB, HSP90AA1, MACF1, MDH2, PSPC1, SPTBN1, STXBP1, SYNE1, XRCC5, XRCC6
GO Function	GO:0043167	Ion binding	24	6033	0.29	0.28	0.021	AFG3L2, DNM1, DNM3, DYNC1H1, EEF1A2, FARSB, FAT2, HK1, HSP90AA1, ITSN1, KRAS, MACF1, NSF, SEPTIN11, SEPTIN3, SEPTIN4, SEPTIN6, SEPTIN9, SYN1, SYN2, TUBB2A, TUBB3, XRCC5, XRCC6
GO Function	GO:0005198	Structural molecule activity	8	776	0.71	0.41	0.026	ANK2, DNM3, MACF1, MAP1B, SEPTIN4, SPTBN1, TUBB2A, TUBB3
GO Function	GO:0008017	Microtubule binding	5	269	0.96	0.44	0.037	DNM1, DNM3, MACF1, MAP1B, SGIP1
GO Function	GO:0003779	Actin binding	6	448	0.82	0.39	0.047	MACF1, MAP1B, SPTBN1, SYN1, SYNE1, WDR1
GO Component	GO:0030054	Cell junction	24	2115	0.75	1.03	1.4e-10	ANK2, AP3B2, DNM1, DNM3, EEF1A2, FAT2, GRID2, ITSN1, KRAS, MACF1, MAP1B, MPP1, MPP6, NSF, SEPTIN11, SEPTIN3, SEPTIN4, SEPTIN6, SPTBN1, STXBP1, SYN1, SYN2, SYNE1, WDR1
GO Component	GO:0045202	Synapse	18	1350	0.82	1.05	3.3e-08	ANK2, AP3B2, DNM1, DNM3, EEF1A2, GRID2, ITSN1, MAP1B, NSF, SEPTIN11, SEPTIN3, SEPTIN4, SEPTIN6, SPTBN1, STXBP1, SYN1, SYN2, SYNE1

Table S7: Cerebellar vermis pathways enriched in downregulated proteins (FDR-adjusted $p < 0.05$) of adults with idiopathic autism *(continued)*

Category	Term ID	Term description	Gene count	Back-ground	Strength	Signal	FDR	Matching proteins in network
GO Component	GO:0043005	Neuron projection	18	1391	0.80	1.03	3.5e-08	ANK2, AP3B2, DNM3, DYNC1H1, GRID2, HSP90AA1, ITSN1, MAP1B, MPP1, NSF, SEPTIN11, SEPTIN3, SEPTIN4, SEPTIN6, SPTBN1, STXBP1, SYN1, TUBB3
GO Component	GO:0005940	Septin ring	5	14	2.25	2.45	1.5e-07	SEPTIN11, SEPTIN3, SEPTIN4, SEPTIN6, SEPTIN9
GO Component	GO:0031105	Septin complex	5	14	2.25	2.45	1.5e-07	SEPTIN11, SEPTIN3, SEPTIN4, SEPTIN6, SEPTIN9
GO Component	GO:0005856	Cytoskeleton	21	2369	0.64	0.77	2.0e-07	ANK2, CEP76, DNM1, DNM3, DYNC1H1, MACF1, MAP1B, MPP1, SEPTIN11, SEPTIN3, SEPTIN4, SEPTIN6, SEPTIN9, SPTBN1, STXBP1, SYN1, SYNE1, TAGLN3, TUBB2A, TUBB3, WDR1
GO Component	GO:0042995	Cell projection	21	2379	0.64	0.77	2.0e-07	ANK2, AP3B2, DNM3, DYNC1H1, GRID2, HSP90AA1, ITSN1, MACF1, MAP1B, MPP1, NSF, SEPTIN11, SEPTIN3, SEPTIN4, SEPTIN6, SEPTIN9, SPTBN1, STXBP1, SYN1, TUBB3, WDR1
GO Component	GO:0120025	Plasma membrane bounded cell projection	20	2268	0.64	0.75	5.5e-07	ANK2, AP3B2, DNM3, DYNC1H1, GRID2, HSP90AA1, ITSN1, MACF1, MAP1B, MPP1, NSF, SEPTIN11, SEPTIN3, SEPTIN4, SEPTIN6, SEPTIN9, SPTBN1, STXBP1, SYN1, TUBB3

Table S7: Cerebellar vermis pathways enriched in downregulated proteins (FDR-adjusted $p < 0.05$) of adults with idiopathic autism *(continued)*

Category	Term ID	Term description	Gene count	Back-ground	Strength	Signal	FDR	Matching proteins in network
GO Component	GO:0030424	Axon	12	651	0.96	1.15	9.0e-07	AP3B2, DNM3, DYNC1H1, HSP90AA1, MAP1B, SEPTIN11, SEPTIN4, SEPTIN6, SPTBN1, STXBP1, SYN1, TUBB3
GO Component	GO:0005938	Cell cortex	9	304	1.16	1.37	1.9e-06	DYNC1H1, MPP1, SEPTIN11, SEPTIN3, SEPTIN4, SEPTIN6, SEPTIN9, SPTBN1, WDR1
GO Component	GO:0098794	Postsynapse	11	621	0.94	1.05	5.6e-06	ANK2, DNM3, GRID2, MAP1B, NSF, SEPTIN11, SPTBN1, STXBP1, SYN1, SYN2, SYNE1
GO Component	GO:0098793	Presynapse	10	525	0.97	1.04	1.3e-05	AP3B2, DNM1, DNM3, ITSN1, SEPTIN3, SEPTIN4, SEPTIN6, STXBP1, SYN1, SYN2
GO Component	GO:0070161	Anchoring junction	14	1325	0.72	0.75	2.6e-05	ANK2, FAT2, GRID2, ITSN1, KRAS, MAP1B, MPP1, MPP6, SEPTIN11, SEPTIN3, SEPTIN4, SYN1, SYN2, WDR1
GO Component	GO:0015630	Microtubule cytoskeleton	14	1355	0.71	0.74	3.2e-05	CEP76, DNM1, DNM3, DYNC1H1, MACF1, MAP1B, MPP1, SEPTIN11, SEPTIN3, SEPTIN4, SEPTIN6, SEPTIN9, TUBB2A, TUBB3
GO Component	GO:0032153	Cell division site	5	70	1.55	1.38	5.3e-05	SEPTIN11, SEPTIN3, SEPTIN4, SEPTIN6, SEPTIN9
GO Component	GO:0031982	Vesicle	22	3957	0.44	0.47	1.4e-04	ANK2, AP3B2, DNM1, DNM3, DYNC1H1, FAT2, HSP90AA1, ITSN1, MDH2, MPP6, SEPTIN4, SEPTIN6, SGIP1, SPTBN1, STXBP1, SYN1, SYN2, TUBB2A, TUBB3, WDR1, XRCC5, XRCC6

Table S7: Cerebellar vermis pathways enriched in downregulated proteins (FDR-adjusted p < 0.05) of adults with idiopathic autism *(continued)*

Category	Term ID	Term description	Gene count	Back-ground	Strength	Signal	FDR	Matching proteins in network
GO Component	GO:0071944	Cell periphery	27	6015	0.34	0.40	1.9e-04	ANK2, DNM1, DNM3, DYNC1H1, FAT2, GRID2, HSP90AA1, ITSN1, KRAS, MACF1, MAP1B, MPP1, MPP6, NSF, SEPTIN11, SEPTIN3, SEPTIN4, SEPTIN6, SEPTIN9, SGIP1, SPTBN1, STXBP1, SYN2, SYNE1, TUBB3, WDR1, XRCC5
GO Component	GO:0005874	Microtubule	8	453	0.94	0.82	3.6e-04	DNM1, DNM3, DYNC1H1, MACF1, MAP1B, SEPTIN9, TUBB2A, TUBB3
GO Component	GO:0099512	Supramolecular fiber	11	1000	0.73	0.67	3.6e-04	ANK2, DNM1, DNM3, DYNC1H1, MACF1, MAP1B, SEPTIN9, SPTBN1, SYNE1, TUBB2A, TUBB3
GO Component	GO:0014069	Postsynaptic density	7	324	1.03	0.88	3.7e-04	DNM3, GRID2, MAP1B, NSF, SPTBN1, SYN1, SYN2
GO Component	GO:0030425	Dendrite	9	624	0.85	0.76	3.7e-04	DNM3, GRID2, HSP90AA1, MAP1B, NSF, SEPTIN11, SEPTIN4, SYN1, TUBB3
GO Component	GO:0098590	Plasma membrane region	12	1237	0.68	0.63	3.7e-04	ANK2, DNM1, DNM3, GRID2, HSP90AA1, ITSN1, MACF1, SEPTIN6, SGIP1, SPTBN1, STXBP1, SYNE1
GO Component	GO:0043232	Intracellular non-membrane-bounded organelle	24	5191	0.36	0.39	5.5e-04	ANK2, CEP76, DNM1, DNM3, DYNC1H1, MACF1, MAP1B, MPP1, PSPC1, SEPTIN11, SEPTIN3, SEPTIN4, SEPTIN6, SEPTIN9, SPTBN1, STXBP1, SYN1, SYNE1, TAGLN3, TUBB2A, TUBB3, WDR1, XRCC5, XRCC6

Table S7: Cerebellar vermis pathways enriched in downregulated proteins (FDR-adjusted $p < 0.05$) of adults with idiopathic autism *(continued)*

Category	Term ID	Term description	Gene count	Back-ground	Strength	Signal	FDR	Matching proteins in network
GO Component	GO:0005737	Cytoplasm	37	12056	0.18	0.29	5.7e-04	AFG3L2, ANK2, AP3B2, CEP76, DNM1, DNM3, DYNC1H1, EEF1A2, FARSB, FAT2, HK1, HSP90AA1, ITSN1, KRAS, LRRC40, MACF1, MAP1B, MDH2, MPP1, NSF, PSPC1, SEPTIN11, SEPTIN3, SEPTIN4, SEPTIN6, SEPTIN9, SGIP1, SPTBN1, STXBP1, SYN1, SYN2, SYNE1, TUBB2A, TUBB3, WDR1, XRCC5, XRCC6
GO Component	GO:0099080	Supramolecular complex	12	1366	0.64	0.57	7.3e-04	ANK2, DNM1, DNM3, DYNC1H1, MACF1, MAP1B, SEPTIN6, SEPTIN9, SPTBN1, SYNE1, TUBB2A, TUBB3
GO Component	GO:0043226	Organelle	39	14017	0.14	0.26	1.3e-03	AFG3L2, ANK2, AP3B2, CEP76, DNM1, DNM3, DYNC1H1, EEF1A2, FAT2, GRID2, HK1, HSP90AA1, ITSN1, KRAS, LRRC40, MACF1, MAP1B, MDH2, MPP1, MPP6, NSF, PSPC1, SEPTIN11, SEPTIN3, SEPTIN4, SEPTIN6, SEPTIN9, SGIP1, SPTBN1, STXBP1, SYN1, SYN2, SYNE1, TAGLN3, TUBB2A, TUBB3, WDR1, XRCC5, XRCC6
GO Component	GO:0043564	Ku70:Ku80 complex	2	2	2.69	1.08	1.4e-03	XRCC5, XRCC6

Table S7: Cerebellar vermis pathways enriched in downregulated proteins (FDR-adjusted $p < 0.05$) of adults with idiopathic autism *(continued)*

Category	Term ID	Term description	Gene count	Back-ground	Strength	Signal	FDR	Matching proteins in network
GO Component	GO:0016020	Membrane	32	9523	0.22	0.30	2.3e-03	AFG3L2, ANK2, AP3B2, DNM1, DNM3, DYNC1H1, EEF1A2, FARSB, FAT2, GRID2, HK1, HSP90AA1, ITSN1, KRAS, LRRC40, MACF1, MAP1B, MDH2, MPP1, MPP6, NSF, SEPTIN4, SEPTIN6, SGIP1, SPTBN1, STXBP1, SYN1, SYN2, SYNE1, WDR1, XRCC5, XRCC6
GO Component	GO:0070418	DNA-dependent protein kinase complex	2	4	2.39	0.93	3.2e-03	XRCC5, XRCC6
GO Component	GO:0098978	Glutamatergic synapse	6	334	0.95	0.67	3.2e-03	DNM1, DNM3, GRID2, SPTBN1, STXBP1, SYN2
GO Component	GO:0032991	Protein-containing complex	23	5506	0.31	0.33	4.4e-03	AFG3L2, AP3B2, CEP76, DNM1, DNM3, DYNC1H1, EEF1A2, FARSB, GRID2, HSP90AA1, MAP1B, SEPTIN11, SEPTIN3, SEPTIN4, SEPTIN6, SEPTIN9, SGIP1, SPTBN1, STXBP1, SYN2, SYNE1, XRCC5, XRCC6
GO Component	GO:0097227	Sperm annulus	2	5	2.29	0.88	4.4e-03	SEPTIN4, SEPTIN6
GO Component	GO:0097060	Synaptic membrane	6	375	0.90	0.60	5.5e-03	ANK2, DNM1, DNM3, GRID2, STXBP1, SYNE1

Table S7: Cerebellar vermis pathways enriched in downregulated proteins (FDR-adjusted $p < 0.05$) of adults with idiopathic autism *(continued)*

Category	Term ID	Term description	Gene count	Back-ground	Strength	Signal	FDR	Matching proteins in network
GO Component	GO:0043229	Intracellular organelle	37	13231	0.14	0.24	8.0e-03	AFG3L2, ANK2, AP3B2, CEP76, DNM1, DNM3, DYNC1H1, EEF1A2, FAT2, HK1, HSP90AA1, ITSN1, KRAS, LRRC40, MACF1, MAP1B, MDH2, MPP1, NSF, PSPC1, SEPTIN11, SEPTIN3, SEPTIN4, SEPTIN6, SEPTIN9, SGIP1, SPTBN1, STXBP1, SYN1, SYN2, SYNE1, TAGLN3, TUBB2A, TUBB3, WDR1, XRCC5, XRCC6
GO Component	GO:0099503	Secretory vesicle	9	1047	0.63	0.45	9.8e-03	DYNC1H1, HSP90AA1, SEPTIN4, SEPTIN6, STXBP1, SYN1, SYN2, XRCC5, XRCC6
GO Component	GO:0070419	Nonhomologous end joining complex	2	9	2.04	0.74	9.9e-03	XRCC5, XRCC6
GO Component	GO:0000783	Nuclear telomere cap complex	2	13	1.88	0.64	0.018	XRCC5, XRCC6
GO Component	GO:0043197	Dendritic spine	4	176	1.05	0.53	0.020	DNM3, GRID2, MAP1B, SEPTIN11
GO Component	GO:0070062	Extracellular exosome	12	2096	0.45	0.32	0.027	DNM1, DNM3, DYNC1H1, FAT2, HSP90AA1, MDH2, MPP6, SPTBN1, STXBP1, TUBB2A, TUBB3, WDR1
GO Component	GO:0008021	Synaptic vesicle	4	212	0.97	0.45	0.033	SEPTIN4, SEPTIN6, SYN1, SYN2
GO Component	GO:0030117	Membrane coat	3	91	1.21	0.50	0.033	AP3B2, DNM1, SGIP1
GO Component	GO:0031410	Cytoplasmic vesicle	13	2482	0.41	0.30	0.033	ANK2, AP3B2, DYNC1H1, HSP90AA1, ITSN1, SEPTIN4, SEPTIN6, SGIP1, STXBP1, SYN1, SYN2, XRCC5, XRCC6

Table S7: Cerebellar vermis pathways enriched in downregulated proteins (FDR-adjusted $p < 0.05$) of adults with idiopathic autism *(continued)*

Category	Term ID	Term description	Gene count	Back-ground	Strength	Signal	FDR	Matching proteins in network
GO Component	GO:0005886	Plasma membrane	21	5544	0.27	0.26	0.034	ANK2, DNM1, DNM3, FAT2, GRID2, HSP90AA1, ITSN1, KRAS, MACF1, MAP1B, MPP1, MPP6, NSF, SEPTIN6, SGIP1, SPTBN1, STXBP1, SYN2, SYNE1, WDR1, XRCC5
GO Component	GO:0012505	Endomembrane system	19	4721	0.30	0.26	0.034	ANK2, AP3B2, DNM3, DYNC1H1, FAT2, HSP90AA1, ITSN1, KRAS, MACF1, NSF, SEPTIN4, SEPTIN6, SGIP1, STXBP1, SYN1, SYN2, SYNE1, XRCC5, XRCC6
GO Component	GO:0030863	Cortical cytoskeleton	3	102	1.16	0.46	0.041	MPP1, SPTBN1, WDR1
GO Component	GO:0031430	M band	2	24	1.61	0.50	0.041	ANK2, SPTBN1
GO Component	GO:0005622	Intracellular anatomical structure	38	14891	0.10	0.20	0.042	AFG3L2, ANK2, AP3B2, CEP76, DNM1, DNM3, DYNC1H1, EEF1A2, FARSB, FAT2, HK1, HSP90AA1, ITSN1, KRAS, LRRC40, MACF1, MAP1B, MDH2, MPP1, NSF, PSPC1, SEPTIN11, SEPTIN3, SEPTIN4, SEPTIN6, SEPTIN9, SGIP1, SPTBN1, STXBP1, SYN1, SYN2, SYNE1, TAGLN3, TUBB2A, TUBB3, WDR1, XRCC5, XRCC6
GO Component	GO:0031968	Organelle outer membrane	4	236	0.92	0.42	0.043	HK1, KRAS, SEPTIN4, SYNE1
STRING clusters	CL:30787	Septin ring	5	10	2.39	2.36	3.6e-07	SEPTIN11, SEPTIN3, SEPTIN4, SEPTIN6, SEPTIN9
STRING clusters	CL:30790	Sperm annulus, and Neuritis	3	5	2.47	1.33	2.8e-04	SEPTIN11, SEPTIN6, SEPTIN9

Table S7: Cerebellar vermis pathways enriched in downregulated proteins (FDR-adjusted $p < 0.05$) of adults with idiopathic autism *(continued)*

Category	Term ID	Term description	Gene count	Back-ground	Strength	Signal	FDR	Matching proteins in network
STRING clusters	CL:13769	Clathrin coat, and Presynaptic endocytosis	5	73	1.53	1.16	2.9e-04	AP3B2, DNM1, DNM3, ITSN1, SGIP1
STRING clusters	CL:13770	Clathrin coat, and Presynaptic endocytosis	4	48	1.61	0.96	1.7e-03	DNM1, DNM3, ITSN1, SGIP1
STRING clusters	CL:26209	Mixed, incl. Golgi-to-ER retrograde transport, and Cytoplasmic dynein complex	5	157	1.20	0.69	6.4e-03	DYNC1H1, MACF1, TAGLN3, TUBB2A, TUBB3
STRING clusters	CL:26373	Mixed, incl. Post-chaperonin tubulin folding pathway, and Microtubule end	4	75	1.42	0.74	6.4e-03	MACF1, TAGLN3, TUBB2A, TUBB3
STRING clusters	CL:26380	Post-chaperonin tubulin folding pathway, and Calponin repeat	3	39	1.58	0.59	0.022	TAGLN3, TUBB2A, TUBB3
STRING clusters	CL:6250	Nonhomologous end joining complex	2	5	2.29	0.62	0.022	XRCC5, XRCC6
STRING clusters	CL:25027	Spectrin, and Ankyrin, UPA domain	2	6	2.22	0.59	0.027	ANK2, SPTBN1
STRING clusters	CL:26390	Microtubule-dependent trafficking of connexons from Golgi to the plasma membrane	2	6	2.22	0.59	0.027	TUBB2A, TUBB3
KEGG	hsa05100	Bacterial invasion of epithelial cells	6	70	1.63	1.75	3.1e-06	DNM1, DNM3, SEPTIN11, SEPTIN3, SEPTIN6, SEPTIN9
KEGG	hsa04721	Synaptic vesicle cycle	4	72	1.44	0.85	2.8e-03	DNM1, DNM3, NSF, STXBP1

Table S7: Cerebellar vermis pathways enriched in downregulated proteins (FDR-adjusted p < 0.05) of adults with idiopathic autism *(continued)*

Category	Term ID	Term description	Gene count	Back-ground	Strength	Signal	FDR	Matching proteins in network
KEGG	hsa05131	Shigellosis	5	218	1.05	0.61	9.4e-03	HK1, SEPTIN11, SEPTIN3, SEPTIN6, SEPTIN9
KEGG	hsa03450	Non-homologous end-joining	2	12	1.91	0.56	0.030	XRCC5, XRCC6
Reactome	HSA-373760	L1CAM interactions	6	120	1.39	1.18	1.5e-04	ANK2, DNM1, DNM3, SPTBN1, TUBB2A, TUBB3
Reactome	HSA-5653656	Vesicle-mediated transport	11	666	0.91	0.85	1.5e-04	ANK2, DNM1, DNM3, DYNC1H1, HSP90AA1, ITSN1, NSF, SGIP1, SPTBN1, TUBB2A, TUBB3
Reactome	HSA-6807878	COPI-mediated anterograde transport	6	101	1.47	1.22	1.5e-04	ANK2, DYNC1H1, NSF, SPTBN1, TUBB2A, TUBB3
Reactome	HSA-199991	Membrane Trafficking	10	626	0.90	0.82	2.3e-04	ANK2, DNM1, DNM3, DYNC1H1, ITSN1, NSF, SGIP1, SPTBN1, TUBB2A, TUBB3
Reactome	HSA-112315	Transmission across Chemical Synapses	7	268	1.11	0.92	4.5e-04	KRAS, NSF, STXBP1, SYN1, SYN2, TUBB2A, TUBB3
Reactome	HSA-380320	Recruitment of NuMA to mitotic centrosomes	5	93	1.42	1.07	4.5e-04	CEP76, DYNC1H1, HSP90AA1, TUBB2A, TUBB3
Reactome	HSA-422475	Axon guidance	9	551	0.91	0.78	4.5e-04	ANK2, DNM1, DNM3, HSP90AA1, ITSN1, KRAS, SPTBN1, TUBB2A, TUBB3
Reactome	HSA-9646399	Aggrephagy	4	42	1.67	1.13	5.1e-04	DYNC1H1, HSP90AA1, TUBB2A, TUBB3
Reactome	HSA-437239	Recycling pathway of L1	4	48	1.61	1.08	6.9e-04	DNM1, DNM3, TUBB2A, TUBB3
Reactome	HSA-2132295	MHC class II antigen presentation	5	122	1.30	0.94	9.8e-04	DNM1, DNM3, DYNC1H1, TUBB2A, TUBB3
Reactome	HSA-3371497	HSP90 chaperone cycle for steroid hormone receptors (SHR) in the presence of ligand	4	55	1.55	1.01	9.9e-04	DYNC1H1, HSP90AA1, TUBB2A, TUBB3

Table S7: Cerebellar vermis pathways enriched in downregulated proteins (FDR-adjusted $p < 0.05$) of adults with idiopathic autism *(continued)*

Category	Term ID	Term description	Gene count	Back-ground	Strength	Signal	FDR	Matching proteins in network
Reactome	HSA-181429	Serotonin Neurotransmitter Release Cycle	3	18	1.91	1.02	1.5e-03	STXBP1, SYN1, SYN2
Reactome	HSA-212676	Dopamine Neurotransmitter Release Cycle	3	23	1.81	0.93	2.6e-03	STXBP1, SYN1, SYN2
Reactome	HSA-69275	G2/M Transition	5	195	1.10	0.67	5.7e-03	CEP76, DYNC1H1, HSP90AA1, TUBB2A, TUBB3
Reactome	HSA-5617833	Cilium Assembly	5	200	1.09	0.67	5.8e-03	CEP76, DYNC1H1, HSP90AA1, TUBB2A, TUBB3
Reactome	HSA-164843	2-LTR circle formation	2	7	2.15	0.70	0.014	XRCC5, XRCC6
Reactome	HSA-190828	Gap junction trafficking	3	48	1.49	0.65	0.014	DNM1, TUBB2A, TUBB3
Reactome	HSA-6811436	COPI-independent Golgi-to-ER retrograde traffic	3	52	1.45	0.64	0.015	DYNC1H1, TUBB2A, TUBB3
Reactome	HSA-8856688	Golgi-to-ER retrograde transport	4	133	1.17	0.59	0.015	DYNC1H1, NSF, TUBB2A, TUBB3
Reactome	HSA-8856828	Clathrin-mediated endocytosis	4	145	1.13	0.57	0.017	DNM1, DNM3, ITSN1, SGIP1
Reactome	HSA-9634285	Constitutive Signaling by Overexpressed ERBB2	2	11	1.95	0.62	0.021	HSP90AA1, KRAS
Reactome	HSA-3270619	IRF3-mediated induction of type I IFN	2	13	1.88	0.59	0.025	XRCC5, XRCC6
Reactome	HSA-380259	Loss of Nlp from mitotic centrosomes	3	69	1.33	0.55	0.025	CEP76, DYNC1H1, HSP90AA1
Reactome	HSA-177504	Retrograde neurotrophin signalling	2	14	1.85	0.58	0.027	DNM1, DNM3
Reactome	HSA-8854518	AURKA Activation by TPX2	3	72	1.31	0.54	0.027	CEP76, DYNC1H1, HSP90AA1

Table S7: Cerebellar vermis pathways enriched in downregulated proteins (FDR-adjusted $p < 0.05$) of adults with idiopathic autism *(continued)*

Category	Term ID	Term description	Gene count	Back-ground	Strength	Signal	FDR	Matching proteins in network
Reactome	HSA-5637810	Constitutive Signaling by EGFRvIII	2	15	1.82	0.57	0.028	HSP90AA1, KRAS
Reactome	HSA-8852276	The role of GTSE1 in G2/M progression after G2 checkpoint	3	77	1.28	0.52	0.028	HSP90AA1, TUBB2A, TUBB3
Reactome	HSA-9659379	Sensory processing of sound	3	76	1.29	0.53	0.028	MPP1, SPTBN1, SYN1
Reactome	HSA-9665348	Signaling by ERBB2 ECD mutants	2	16	1.79	0.56	0.029	HSP90AA1, KRAS
Reactome	HSA-380270	Recruitment of mitotic centrosome proteins and complexes	3	80	1.27	0.52	0.029	CEP76, DYNC1H1, HSP90AA1
Reactome	HSA-438064	Post NMDA receptor activation events	3	80	1.27	0.52	0.029	KRAS, TUBB2A, TUBB3
Reactome	HSA-112314	Neurotransmitter receptors and postsynaptic signal transmission	4	204	0.98	0.45	0.035	KRAS, NSF, TUBB2A, TUBB3
Reactome	HSA-1236382	Constitutive Signaling by Ligand-Responsive EGFR Cancer Variants	2	19	1.71	0.53	0.035	HSP90AA1, KRAS
Reactome	HSA-190840	Microtubule-dependent trafficking of connexons from Golgi to the plasma membrane	2	19	1.71	0.53	0.035	TUBB2A, TUBB3
Reactome	HSA-2565942	Regulation of PLK1 Activity at G2/M Transition	3	87	1.23	0.49	0.035	CEP76, DYNC1H1, HSP90AA1
Reactome	HSA-9703465	Signaling by FLT3 fusion proteins	2	19	1.71	0.53	0.035	KRAS, SPTBN1

Table S7: Cerebellar vermis pathways enriched in downregulated proteins (FDR-adjusted p < 0.05) of adults with idiopathic autism *(continued)*

Category	Term ID	Term description	Gene count	Back-ground	Strength	Signal	FDR	Matching proteins in network
Reactome	HSA-389977	Post-chaperonin tubulin folding pathway	2	22	1.65	0.51	0.040	TUBB2A, TUBB3
Reactome	HSA-5620912	Anchoring of the basal body to the plasma membrane	3	97	1.18	0.47	0.040	CEP76, DYNC1H1, HSP90AA1
Reactome	HSA-9665686	Signaling by ERBB2 TMD/JMD mutants	2	22	1.65	0.51	0.040	HSP90AA1, KRAS
Reactome	HSA-6811434	COPI-dependent Golgi-to-ER retrograde traffic	3	99	1.17	0.46	0.041	NSF, TUBB2A, TUBB3
Reactome	HSA-9609690	HCMV Early Events	3	102	1.16	0.45	0.043	DYNC1H1, TUBB2A, TUBB3
Reactome	HSA-389960	Formation of tubulin folding intermediates by CCT/TriC	2	25	1.60	0.48	0.046	TUBB2A, TUBB3
Reactome	HSA-9664565	Signaling by ERBB2 KD Mutants	2	25	1.60	0.48	0.046	HSP90AA1, KRAS
WikiPathways	WP2267	Synaptic vesicle pathway	6	51	1.76	1.95	1.2e-06	DNM1, DNM3, NSF, STXBP1, SYN1, SYN2
Monarch	HP:0002079	Hypoplasia of the corpus callosum	11	462	1.07	1.12	2.0e-05	AP3B2, DNM1, DYNC1H1, EEF1A2, HK1, MACF1, MAP1B, MDH2, STXBP1, TUBB2A, TUBB3
Monarch	HP:0002500	Abnormal cerebral white matter morphology	13	914	0.85	0.84	5.8e-05	AP3B2, DNM1, DYNC1H1, EEF1A2, HK1, KRAS, MACF1, MAP1B, MDH2, STXBP1, SYNE1, TUBB2A, TUBB3
Monarch	HP:0007370	Aplasia/Hypoplasia of the corpus callosum	12	698	0.93	0.92	5.8e-05	AP3B2, DNM1, DYNC1H1, EEF1A2, HK1, KRAS, MACF1, MAP1B, MDH2, STXBP1, TUBB2A, TUBB3

Table S7: Cerebellar vermis pathways enriched in downregulated proteins (FDR-adjusted $p < 0.05$) of adults with idiopathic autism *(continued)*

Category	Term ID	Term description	Gene count	Back-ground	Strength	Signal	FDR	Matching proteins in network
Monarch	HP:0012638	Abnormal nervous system physiology	22	3154	0.54	0.57	5.8e-05	AFG3L2, ANK2, AP3B2, DNM1, DYNC1H1, EEF1A2, FARSB, FAT2, GRID2, HK1, KRAS, MACF1, MAP1B, MDH2, NSF, SEPTIN9, STXBP1, SYN1, SYNE1, TUBB2A, TUBB3, WDR1
Monarch	HP:0033725	Thin corpus callosum	12	709	0.92	0.91	5.8e-05	AP3B2, DNM1, DYNC1H1, EEF1A2, HK1, KRAS, MACF1, MAP1B, MDH2, STXBP1, TUBB2A, TUBB3
Monarch	HP:0000764	Peripheral axonal degeneration	7	174	1.30	1.22	5.9e-05	AFG3L2, DYNC1H1, HK1, KRAS, SEPTIN9, SYNE1, TUBB3
Monarch	HP:0001250	Seizure	16	1607	0.69	0.70	5.9e-05	AFG3L2, ANK2, AP3B2, DNM1, DYNC1H1, EEF1A2, FARSB, HK1, KRAS, MACF1, MAP1B, MDH2, STXBP1, SYN1, TUBB2A, TUBB3
Monarch	HP:0001265	Hyporeflexia	9	353	1.10	1.07	5.9e-05	AP3B2, DNM1, DYNC1H1, EEF1A2, HK1, MDH2, SEPTIN9, STXBP1, SYNE1
Monarch	HP:0001288	Gait disturbance	13	951	0.83	0.83	5.9e-05	AFG3L2, AP3B2, DNM1, DYNC1H1, EEF1A2, FAT2, GRID2, HK1, MAP1B, MDH2, STXBP1, SYNE1, TUBB3
Monarch	HP:0001315	Reduced tendon reflexes	10	516	0.98	0.97	5.9e-05	AP3B2, DNM1, DYNC1H1, EEF1A2, HK1, KRAS, MDH2, SEPTIN9, STXBP1, SYNE1
Monarch	HP:0001317	Abnormal cerebellum morphology	12	788	0.88	0.87	5.9e-05	AFG3L2, AP3B2, DYNC1H1, FAT2, GRID2, HK1, KRAS, MACF1, MDH2, STXBP1, SYNE1, TUBB3

Table S7: Cerebellar vermis pathways enriched in downregulated proteins (FDR-adjusted $p < 0.05$) of adults with idiopathic autism *(continued)*

Category	Term ID	Term description	Gene count	Back-ground	Strength	Signal	FDR	Matching proteins in network
Monarch	HP:0002167	Neurological speech impairment	15	1354	0.74	0.74	5.9e-05	AFG3L2, AP3B2, DNM1, DYNC1H1, EEF1A2, FAT2, GRID2, HK1, KRAS, MACF1, MAP1B, MDH2, STXBP1, SYNE1, TUBB3
Monarch	HP:0010993	Abnormal cerebral subcortex morphology	13	999	0.81	0.81	5.9e-05	AP3B2, DNM1, DYNC1H1, EEF1A2, HK1, KRAS, MACF1, MAP1B, MDH2, STXBP1, SYNE1, TUBB2A, TUBB3
Monarch	HP:0001324	Muscle weakness	12	861	0.84	0.83	6.3e-05	AFG3L2, AP3B2, DNM1, DYNC1H1, EEF1A2, HK1, KRAS, MDH2, SEPTIN9, STXBP1, SYNE1, TUBB3
Monarch	HP:0031826	Abnormal reflex	13	1046	0.79	0.79	6.3e-05	AFG3L2, AP3B2, DNM1, DYNC1H1, EEF1A2, GRID2, HK1, KRAS, MDH2, SEPTIN9, STXBP1, SYNE1, TUBB3
Monarch	HP:0001311	Abnormal nervous system electrophysiology	10	545	0.96	0.94	7.0e-05	AFG3L2, AP3B2, DNM1, DYNC1H1, EEF1A2, HK1, KRAS, MAP1B, STXBP1, TUBB2A
Monarch	HP:0012443	Abnormality of brain morphology	18	2254	0.59	0.61	7.0e-05	AFG3L2, AP3B2, DNM1, DYNC1H1, EEF1A2, FARSB, FAT2, GRID2, HK1, KRAS, MACF1, MAP1B, MDH2, NSF, STXBP1, SYNE1, TUBB2A, TUBB3
Monarch	HP:0012639	Abnormal nervous system morphology	19	2546	0.57	0.59	7.0e-05	AFG3L2, AP3B2, DNM1, DYNC1H1, EEF1A2, FARSB, FAT2, GRID2, HK1, KRAS, MACF1, MAP1B, MDH2, NSF, SEPTIN9, STXBP1, SYNE1, TUBB2A, TUBB3

Table S7: Cerebellar vermis pathways enriched in downregulated proteins (FDR-adjusted $p < 0.05$) of adults with idiopathic autism *(continued)*

Category	Term ID	Term description	Gene count	Back-ground	Strength	Signal	FDR	Matching proteins in network
Monarch	HP:0100022	Abnormality of movement	16	1758	0.65	0.66	7.0e-05	AFG3L2, AP3B2, DNM1, DYNC1H1, EEF1A2, FAT2, GRID2, HK1, KRAS, MACF1, MAP1B, MDH2, SEPTIN9, STXBP1, SYNE1, TUBB3
Monarch	HP:0012759	Neurodevelopmental abnormality	18	2323	0.58	0.59	8.8e-05	AFG3L2, AP3B2, DNM1, DYNC1H1, EEF1A2, FARSB, GRID2, HK1, KRAS, MACF1, MAP1B, MDH2, STXBP1, SYN1, SYNE1, TUBB2A, TUBB3, WDR1
Monarch	HP:0001272	Cerebellar atrophy	8	346	1.06	0.94	2.2e-04	AFG3L2, AP3B2, FAT2, GRID2, HK1, MDH2, STXBP1, SYNE1
Monarch	HP:0003477	Peripheral axonal neuropathy	6	145	1.31	1.08	2.5e-04	AFG3L2, DYNC1H1, HK1, KRAS, SYNE1, TUBB3
Monarch	HP:0002060	Abnormal cerebral morphology	15	1786	0.62	0.57	4.5e-04	AP3B2, DNM1, DYNC1H1, EEF1A2, FARSB, HK1, KRAS, MACF1, MAP1B, MDH2, NSF, STXBP1, SYNE1, TUBB2A, TUBB3
Monarch	HP:0002977	Aplasia/Hypoplasia involving the central nervous system	14	1534	0.65	0.60	4.5e-04	AFG3L2, AP3B2, DNM1, DYNC1H1, EEF1A2, FARSB, HK1, KRAS, MACF1, MAP1B, MDH2, STXBP1, TUBB2A, TUBB3
Monarch	HP:0007359	Focal-onset seizure	7	265	1.11	0.92	4.5e-04	DNM1, DYNC1H1, EEF1A2, MAP1B, STXBP1, SYN1, TUBB3
Monarch	HP:0007364	Aplasia/Hypoplasia of the cerebrum	13	1300	0.69	0.63	4.5e-04	AP3B2, DNM1, DYNC1H1, EEF1A2, FARSB, HK1, KRAS, MACF1, MAP1B, MDH2, STXBP1, TUBB2A, TUBB3

Table S7: Cerebellar vermis pathways enriched in downregulated proteins (FDR-adjusted $p < 0.05$) of adults with idiopathic autism *(continued)*

Category	Term ID	Term description	Gene count	Back-ground	Strength	Signal	FDR	Matching proteins in network
Monarch	HP:0011804	Abnormal muscle physiology	16	2088	0.58	0.54	5.0e-04	AFG3L2, AP3B2, DNM1, DYNC1H1, EEF1A2, FARSB, GRID2, HK1, KRAS, MACF1, MDH2, SEPTIN9, STXBP1, SYNE1, TUBB2A, TUBB3
Monarch	HP:0000708	Behavioral abnormality	15	1839	0.60	0.56	5.5e-04	AFG3L2, AP3B2, DNM1, DYNC1H1, EEF1A2, GRID2, HK1, KRAS, MAP1B, MDH2, NSF, STXBP1, SYN1, SYNE1, TUBB3
Monarch	HP:0000639	Nystagmus	11	936	0.76	0.66	6.5e-04	AFG3L2, AP3B2, DNM1, EEF1A2, FAT2, GRID2, HK1, KRAS, STXBP1, SYNE1, TUBB3
Monarch	HP:0011443	Abnormality of coordination	11	945	0.76	0.66	6.8e-04	AFG3L2, AP3B2, DNM1, DYNC1H1, EEF1A2, FAT2, GRID2, HK1, STXBP1, SYNE1, TUBB3
Monarch	HP:0012547	Abnormal involuntary eye movements	11	943	0.76	0.66	6.8e-04	AFG3L2, AP3B2, DNM1, EEF1A2, FAT2, GRID2, HK1, KRAS, STXBP1, SYNE1, TUBB3
Monarch	HP:0200134	Epileptic encephalopathy	5	100	1.39	1.00	7.1e-04	AP3B2, DNM1, EEF1A2, MDH2, STXBP1
Monarch	HP:0010844	EEG with multifocal slow activity	4	43	1.66	1.08	7.6e-04	AP3B2, DNM1, EEF1A2, STXBP1
Monarch	HP:0002353	EEG abnormality	8	441	0.95	0.77	8.3e-04	AFG3L2, AP3B2, DNM1, EEF1A2, KRAS, MAP1B, STXBP1, TUBB2A
Monarch	HP:0000750	Delayed speech and language development	10	790	0.79	0.67	9.4e-04	AP3B2, DNM1, EEF1A2, GRID2, HK1, MACF1, MAP1B, MDH2, STXBP1, TUBB3

Table S7: Cerebellar vermis pathways enriched in downregulated proteins (FDR-adjusted $p < 0.05$) of adults with idiopathic autism *(continued)*

Category	Term ID	Term description	Gene count	Back-ground	Strength	Signal	FDR	Matching proteins in network
Monarch	HP:0025270	Abnormal esophagus physiology	9	619	0.85	0.70	1.0e-03	AFG3L2, AP3B2, DNM1, DYNC1H1, EEF1A2, FARSB, MACF1, MAP1B, STXBP1
Monarch	HP:0000729	Autistic behavior	8	460	0.93	0.74	1.1e-03	AP3B2, DNM1, DYNC1H1, EEF1A2, MAP1B, STXBP1, SYN1, TUBB3
Monarch	HP:0002463	Language impairment	10	809	0.78	0.65	1.1e-03	AP3B2, DNM1, EEF1A2, GRID2, HK1, MACF1, MAP1B, MDH2, STXBP1, TUBB3
Monarch	HP:0002509	Limb hypertonia	5	112	1.34	0.94	1.1e-03	AP3B2, DNM1, EEF1A2, HK1, STXBP1
Monarch	HP:0030178	Abnormality of central nervous system electrophysiology	8	464	0.93	0.74	1.1e-03	AFG3L2, AP3B2, DNM1, EEF1A2, KRAS, MAP1B, STXBP1, TUBB2A
Monarch	HP:0000496	Abnormality of eye movement	13	1491	0.63	0.55	1.2e-03	AFG3L2, AP3B2, DNM1, EEF1A2, FAT2, GRID2, HK1, KRAS, MACF1, MDH2, STXBP1, SYNE1, TUBB3
Monarch	HP:0002020	Gastroesophageal reflux	7	331	1.02	0.78	1.2e-03	AP3B2, DNM1, DYNC1H1, EEF1A2, FARSB, MAP1B, STXBP1
Monarch	HP:0000508	Ptosis	9	647	0.84	0.67	1.3e-03	AFG3L2, AP3B2, DNM1, EEF1A2, KRAS, SEPTIN9, STXBP1, SYNE1, TUBB3
Monarch	HP:0001252	Hypotonia	13	1505	0.63	0.54	1.3e-03	AP3B2, DNM1, DYNC1H1, EEF1A2, FARSB, GRID2, HK1, KRAS, MACF1, STXBP1, SYNE1, TUBB2A, TUBB3
Monarch	HP:0001332	Dystonia	8	480	0.91	0.72	1.3e-03	AFG3L2, DYNC1H1, EEF1A2, HK1, MDH2, STXBP1, SYNE1, TUBB3

Table S7: Cerebellar vermis pathways enriched in downregulated proteins (FDR-adjusted $p < 0.05$) of adults with idiopathic autism *(continued)*

Category	Term ID	Term description	Gene count	Back-ground	Strength	Signal	FDR	Matching proteins in network
Monarch	HP:0003808	Abnormal muscle tone	14	1773	0.59	0.52	1.3e-03	AFG3L2, AP3B2, DNM1, DYNC1H1, EEF1A2, FARSB, GRID2, HK1, KRAS, MACF1, STXBP1, SYNE1, TUBB2A, TUBB3
Monarch	HP:0009127	Abnormality of the musculature of the limbs	8	481	0.91	0.72	1.3e-03	AFG3L2, AP3B2, DNM1, DYNC1H1, EEF1A2, HK1, STXBP1, SYNE1
Monarch	HP:0012719	Functional abnormality of the gastrointestinal tract	10	840	0.77	0.63	1.3e-03	AFG3L2, AP3B2, DNM1, DYNC1H1, EEF1A2, FARSB, KRAS, MACF1, MAP1B, STXBP1
Monarch	HP:0025032	Abnormality of digestive system physiology	14	1763	0.59	0.52	1.3e-03	AFG3L2, AP3B2, DNM1, DYNC1H1, EEF1A2, FARSB, HK1, KRAS, MACF1, MAP1B, MDH2, STXBP1, TUBB3, WDR1
Monarch	HP:0001251	Ataxia	10	854	0.76	0.62	1.4e-03	AFG3L2, AP3B2, DNM1, EEF1A2, FAT2, GRID2, HK1, STXBP1, SYNE1, TUBB3
Monarch	HP:0011805	Abnormal skeletal muscle morphology	13	1537	0.62	0.54	1.4e-03	AFG3L2, AP3B2, DNM1, DYNC1H1, EEF1A2, GRID2, HK1, KRAS, MDH2, SEPTIN9, STXBP1, SYNE1, TUBB3
Monarch	HP:0001263	Global developmental delay	13	1559	0.61	0.53	1.6e-03	AP3B2, DNM1, DYNC1H1, EEF1A2, GRID2, HK1, KRAS, MACF1, MAP1B, MDH2, STXBP1, TUBB2A, TUBB3
Monarch	HP:0025100	Abnormal hippocampus morphology	4	59	1.52	0.94	1.6e-03	DYNC1H1, EEF1A2, STXBP1, TUBB3

Table S7: Cerebellar vermis pathways enriched in downregulated proteins (FDR-adjusted p < 0.05) of adults with idiopathic autism *(continued)*

Category	Term ID	Term description	Gene count	Back-ground	Strength	Signal	FDR	Matching proteins in network
Monarch	HP:0002493	Upper motor neuron dysfunction	11	1100	0.69	0.57	1.7e-03	AFG3L2, AP3B2, DNM1, DYNC1H1, EEF1A2, GRID2, MACF1, MDH2, STXBP1, SYNE1, TUBB3
Monarch	HP:0000736	Short attention span	7	371	0.97	0.72	1.8e-03	AP3B2, DNM1, EEF1A2, MAP1B, STXBP1, SYNE1, TUBB3
Monarch	HP:0002363	Abnormal brainstem morphology	6	239	1.09	0.78	1.8e-03	DNM1, DYNC1H1, MACF1, SYNE1, TUBB2A, TUBB3
Monarch	HP:0000759	Abnormal peripheral nervous system morphology	8	530	0.87	0.67	1.9e-03	AFG3L2, DYNC1H1, HK1, KRAS, MDH2, SEPTIN9, SYNE1, TUBB3
Monarch	HP:0002538	Abnormal cerebral cortex morphology	9	704	0.80	0.63	1.9e-03	DYNC1H1, EEF1A2, FARSB, KRAS, MACF1, MAP1B, STXBP1, TUBB2A, TUBB3
Monarch	HP:0002536	Abnormal cortical gyration	7	384	0.95	0.70	2.2e-03	DYNC1H1, EEF1A2, MACF1, MAP1B, STXBP1, TUBB2A, TUBB3
Monarch	HP:0002123	Generalized myoclonic seizure	5	146	1.23	0.81	2.3e-03	AFG3L2, DNM1, DYNC1H1, EEF1A2, STXBP1
Monarch	HP:0004305	Involuntary movements	10	933	0.72	0.57	2.4e-03	AFG3L2, AP3B2, DNM1, DYNC1H1, EEF1A2, MACF1, MDH2, STXBP1, SYNE1, TUBB3
Monarch	HP:0000587	Abnormality of the optic nerve	9	737	0.78	0.60	2.5e-03	AFG3L2, AP3B2, DNM1, EEF1A2, GRID2, HK1, KRAS, STXBP1, TUBB3
Monarch	HP:0001276	Hypertonia	10	949	0.72	0.56	2.5e-03	AFG3L2, AP3B2, DNM1, DYNC1H1, EEF1A2, HK1, MACF1, STXBP1, SYNE1, TUBB3
Monarch	HP:0002355	Difficulty walking	6	260	1.06	0.74	2.5e-03	AP3B2, DNM1, EEF1A2, GRID2, HK1, STXBP1

Table S7: Cerebellar vermis pathways enriched in downregulated proteins (FDR-adjusted $p < 0.05$) of adults with idiopathic autism *(continued)*

Category	Term ID	Term description	Gene count	Back-ground	Strength	Signal	FDR	Matching proteins in network
Monarch	HP:0004302	Functional motor deficit	7	396	0.94	0.68	2.5e-03	AP3B2, DNM1, DYNC1H1, EEF1A2, GRID2, HK1, STXBP1
Monarch	HP:0007367	Atrophy/Degeneration affecting the central nervous system	9	744	0.78	0.60	2.5e-03	AP3B2, DNM1, DYNC1H1, EEF1A2, FARSB, GRID2, KRAS, STXBP1, SYNE1
Monarch	HP:0011968	Feeding difficulties	10	947	0.72	0.57	2.5e-03	AP3B2, DNM1, DYNC1H1, EEF1A2, FARSB, HK1, KRAS, MDH2, STXBP1, TUBB3
Monarch	HP:0012758	Neurodevelopmental delay	14	1926	0.55	0.47	2.5e-03	AP3B2, DNM1, DYNC1H1, EEF1A2, GRID2, HK1, KRAS, MACF1, MAP1B, MDH2, STXBP1, SYNE1, TUBB2A, TUBB3
Monarch	HP:0033127	Abnormality of the musculoskeletal system	18	3173	0.45	0.41	2.6e-03	AFG3L2, AP3B2, DNM1, DYNC1H1, EEF1A2, FARSB, GRID2, HK1, KRAS, MACF1, MAP1B, MDH2, SEPTIN9, STXBP1, SYN1, SYNE1, TUBB2A, TUBB3
Monarch	HP:0010818	Generalized tonic seizure	4	73	1.43	0.85	2.7e-03	DNM1, DYNC1H1, EEF1A2, STXBP1
Monarch	HP:0011442	Abnormal central motor function	13	1689	0.58	0.48	2.8e-03	AFG3L2, AP3B2, DNM1, DYNC1H1, EEF1A2, FAT2, GRID2, HK1, MACF1, MDH2, STXBP1, SYNE1, TUBB3
Monarch	HP:0009830	Peripheral neuropathy	7	416	0.92	0.66	3.0e-03	AFG3L2, DYNC1H1, HK1, KRAS, SEPTIN9, SYNE1, TUBB3
Monarch	HP:0011182	Interictal epileptiform activity	6	276	1.03	0.71	3.1e-03	AP3B2, DNM1, EEF1A2, MAP1B, STXBP1, TUBB2A
Monarch	HP:0000494	Downslanted palpebral fissures	7	427	0.91	0.65	3.3e-03	AP3B2, DNM1, DYNC1H1, EEF1A2, KRAS, STXBP1, TUBB3

Table S7: Cerebellar vermis pathways enriched in downregulated proteins (FDR-adjusted $p < 0.05$) of adults with idiopathic autism *(continued)*

Category	Term ID	Term description	Gene count	Back-ground	Strength	Signal	FDR	Matching proteins in network
Monarch	HP:0001344	Absent speech	6	282	1.02	0.70	3.3e-03	AP3B2, DNM1, EEF1A2, MACF1, MDH2, STXBP1
Monarch	HP:0002070	Limb ataxia	4	78	1.40	0.82	3.3e-03	AFG3L2, FAT2, GRID2, SYNE1
Monarch	HP:0002194	Delayed gross motor development	6	284	1.02	0.69	3.3e-03	AP3B2, DYNC1H1, HK1, STXBP1, SYNE1, TUBB3
Monarch	HP:0002365	Hypoplasia of the brainstem	4	79	1.40	0.82	3.3e-03	DYNC1H1, MACF1, TUBB2A, TUBB3
Monarch	HP:0006956	Lateral ventricle dilatation	4	79	1.40	0.82	3.3e-03	DYNC1H1, EEF1A2, STXBP1, TUBB3
Monarch	HP:0200006	Slanting of the palpebral fissure	8	599	0.82	0.60	3.3e-03	AP3B2, DNM1, DYNC1H1, EEF1A2, KRAS, SEPTIN9, STXBP1, TUBB3
Monarch	HP:0000118	Phenotypic abnormality	23	5129	0.34	0.35	3.4e-03	AFG3L2, ANK2, AP3B2, DNM1, DYNC1H1, EEF1A2, FARSB, FAT2, GRID2, HK1, KRAS, MACF1, MAP1B, MDH2, NSF, SEPTIN9, SPTBN1, STXBP1, SYN1, SYNE1, TUBB2A, TUBB3, WDR1
Monarch	HP:0002063	Rigidity	5	169	1.16	0.75	3.4e-03	AFG3L2, AP3B2, DNM1, EEF1A2, STXBP1
Monarch	HP:0001558	Decreased fetal movement	5	171	1.16	0.74	3.5e-03	AP3B2, DNM1, EEF1A2, STXBP1, SYNE1
Monarch	HP:0032794	Myoclonic seizure	5	172	1.16	0.74	3.6e-03	AFG3L2, DNM1, DYNC1H1, EEF1A2, STXBP1
Monarch	HP:0002521	Hypsarrhythmia	5	175	1.15	0.73	3.8e-03	AP3B2, DNM1, EEF1A2, STXBP1, TUBB2A
Monarch	HP:0002133	Status epilepticus	5	176	1.15	0.73	3.9e-03	AP3B2, DNM1, DYNC1H1, EEF1A2, STXBP1
Monarch	HP:0025336	Delayed ability to sit	3	28	1.72	0.85	4.0e-03	AP3B2, HK1, TUBB3
Monarch	HP:0032677	Generalized-onset motor seizure	5	178	1.14	0.72	4.0e-03	AFG3L2, DNM1, DYNC1H1, EEF1A2, STXBP1

Table S7: Cerebellar vermis pathways enriched in downregulated proteins (FDR-adjusted p < 0.05) of adults with idiopathic autism *(continued)*

Category	Term ID	Term description	Gene count	Back-ground	Strength	Signal	FDR	Matching proteins in network
Monarch	HP:0001557	Prenatal movement abnormality	5	180	1.14	0.72	4.1e-03	AP3B2, DNM1, EEF1A2, STXBP1, SYNE1
Monarch	HP:0000492	Abnormal eyelid morphology	10	1058	0.67	0.51	4.5e-03	AP3B2, DNM1, DYNC1H1, EEF1A2, HK1, KRAS, MAP1B, SEPTIN9, STXBP1, TUBB3
Monarch	HP:0002421	Poor head control	5	184	1.13	0.71	4.5e-03	AP3B2, DNM1, EEF1A2, MDH2, STXBP1
Monarch	HP:0010819	Atonic seizure	4	90	1.34	0.77	4.5e-03	DNM1, DYNC1H1, EEF1A2, STXBP1
Monarch	HP:0020219	Motor seizure	6	309	0.98	0.65	4.5e-03	AFG3L2, DNM1, DYNC1H1, EEF1A2, HK1, STXBP1
Monarch	HP:0001336	Myoclonus	6	313	0.97	0.65	4.6e-03	AFG3L2, AP3B2, DNM1, DYNC1H1, EEF1A2, STXBP1
Monarch	HP:0012373	Abnormal eye physiology	15	2405	0.49	0.42	4.6e-03	AFG3L2, AP3B2, DNM1, DYNC1H1, EEF1A2, FAT2, GRID2, HK1, KRAS, MACF1, MDH2, SEPTIN9, STXBP1, SYNE1, TUBB3
Monarch	HP:0000315	Abnormality of the orbital region	11	1319	0.61	0.47	5.2e-03	AP3B2, DNM1, DYNC1H1, EEF1A2, HK1, KRAS, MAP1B, MDH2, SEPTIN9, STXBP1, TUBB3
Monarch	HP:0002066	Gait ataxia	5	192	1.11	0.69	5.2e-03	AFG3L2, FAT2, GRID2, STXBP1, SYNE1
Monarch	HP:0000240	Abnormality of skull size	11	1333	0.61	0.47	5.6e-03	AP3B2, DNM1, DYNC1H1, EEF1A2, FARSB, KRAS, MACF1, MAP1B, STXBP1, SYN1, TUBB3
Monarch	HP:0012795	Abnormal optic disc morphology	8	670	0.77	0.54	5.8e-03	AFG3L2, AP3B2, DNM1, EEF1A2, GRID2, HK1, KRAS, STXBP1

Table S7: Cerebellar vermis pathways enriched in downregulated proteins (FDR-adjusted $p < 0.05$) of adults with idiopathic autism *(continued)*

Category	Term ID	Term description	Gene count	Back-ground	Strength	Signal	FDR	Matching proteins in network
Monarch	HP:0012444	Brain atrophy	8	675	0.77	0.54	6.0e-03	AP3B2, DNM1, DYNC1H1, EEF1A2, FARSB, GRID2, KRAS, STXBP1
Monarch	HP:0032410	Bilateral generalized polymicrogyria	3	34	1.64	0.78	6.0e-03	DYNC1H1, EEF1A2, STXBP1
Monarch	HP:0001257	Spasticity	9	897	0.69	0.50	6.7e-03	AFG3L2, AP3B2, DNM1, DYNC1H1, EEF1A2, MACF1, STXBP1, SYNE1, TUBB3
Monarch	HP:0012447	Abnormal myelination	7	502	0.84	0.56	6.7e-03	AFG3L2, AP3B2, DNM1, EEF1A2, HK1, MDH2, STXBP1
Monarch	HP:0410008	Abnormality of the peripheral nervous system	6	343	0.94	0.60	6.8e-03	ANK2, DYNC1H1, HK1, KRAS, SEPTIN9, TUBB3
Monarch	HP:0002650	Scoliosis	9	908	0.69	0.49	7.2e-03	DYNC1H1, EEF1A2, FARSB, HK1, KRAS, MAP1B, STXBP1, SYNE1, TUBB3
Monarch	HP:0000234	Abnormality of the head	16	2865	0.44	0.37	7.5e-03	AP3B2, DNM1, DYNC1H1, EEF1A2, FARSB, HK1, KRAS, MACF1, MAP1B, MDH2, SEPTIN9, STXBP1, SYN1, SYNE1, TUBB3, WDR1
Monarch	HP:0001098	Abnormal fundus morphology	10	1143	0.63	0.46	7.5e-03	AFG3L2, AP3B2, DNM1, EEF1A2, GRID2, HK1, KRAS, MDH2, STXBP1, TUBB3
Monarch	HP:0001249	Intellectual disability	12	1656	0.55	0.43	7.5e-03	AFG3L2, AP3B2, DNM1, DYNC1H1, EEF1A2, GRID2, HK1, KRAS, MAP1B, STXBP1, SYNE1, TUBB3
Monarch	HP:0001328	Specific learning disability	5	214	1.06	0.63	7.5e-03	FARSB, MAP1B, SYN1, TUBB3, WDR1

Table S7: Cerebellar vermis pathways enriched in downregulated proteins (FDR-adjusted $p < 0.05$) of adults with idiopathic autism *(continued)*

Category	Term ID	Term description	Gene count	Back-ground	Strength	Signal	FDR	Matching proteins in network
Monarch	HP:0000478	Abnormality of the eye	16	2876	0.44	0.37	7.7e-03	AFG3L2, AP3B2, DNM1, DYNC1H1, EEF1A2, FARSB, FAT2, GRID2, HK1, KRAS, MACF1, MDH2, SEPTIN9, STXBP1, SYNE1, TUBB3
Monarch	HP:0004322	Short stature	10	1152	0.63	0.46	7.7e-03	AP3B2, DNM1, DYNC1H1, EEF1A2, FARSB, HK1, KRAS, MACF1, SEPTIN9, STXBP1
Monarch	HP:0000290	Abnormality of the forehead	9	926	0.68	0.48	7.8e-03	AP3B2, DNM1, DYNC1H1, EEF1A2, FARSB, HK1, KRAS, MAP1B, STXBP1
Monarch	HP:0002540	Inability to walk	5	218	1.05	0.63	7.8e-03	DNM1, HK1, MDH2, STXBP1, TUBB3
Monarch	HP:0010821	Focal emotional seizure with laughing	3	39	1.58	0.74	7.8e-03	DYNC1H1, EEF1A2, STXBP1
Monarch	HP:0025097	Eyelid myoclonus	3	39	1.58	0.74	7.8e-03	DYNC1H1, EEF1A2, STXBP1
Monarch	HP:0100660	Dyskinesia	5	221	1.05	0.62	8.0e-03	AP3B2, DNM1, DYNC1H1, EEF1A2, STXBP1
Monarch	HP:0002352	Leukoencephalopathy	5	226	1.04	0.61	8.8e-03	DNM1, DYNC1H1, EEF1A2, HK1, STXBP1
Monarch	HP:0001290	Generalized hypotonia	9	956	0.67	0.47	9.4e-03	AP3B2, DNM1, EEF1A2, FARSB, GRID2, MACF1, STXBP1, SYNE1, TUBB2A
Monarch	HP:0011458	Abdominal symptom	11	1448	0.57	0.43	9.4e-03	AP3B2, DNM1, DYNC1H1, EEF1A2, FARSB, HK1, KRAS, MDH2, STXBP1, TUBB3, WDR1
Monarch	HP:0000648	Optic atrophy	7	548	0.80	0.52	9.6e-03	AFG3L2, AP3B2, DNM1, EEF1A2, HK1, KRAS, STXBP1

Table S7: Cerebellar vermis pathways enriched in downregulated proteins (FDR-adjusted $p < 0.05$) of adults with idiopathic autism *(continued)*

Category	Term ID	Term description	Gene count	Back-ground	Strength	Signal	FDR	Matching proteins in network
Monarch	HP:0000271	Abnormality of the face	15	2641	0.45	0.37	0.010	AP3B2, DNM1, DYNC1H1, EEF1A2, FARSB, HK1, KRAS, MACF1, MAP1B, MDH2, SEPTIN9, STXBP1, SYNE1, TUBB3, WDR1
Monarch	HP:0000546	Retinal degeneration	5	235	1.02	0.59	0.010	AP3B2, DNM1, EEF1A2, HK1, STXBP1
Monarch	HP:0010553	Oculogyric crisis	3	45	1.52	0.69	0.011	DYNC1H1, EEF1A2, STXBP1
Monarch	HP:0002126	Polymicrogyria	5	240	1.01	0.58	0.011	DYNC1H1, EEF1A2, MAP1B, STXBP1, TUBB3
Monarch	HP:0031466	Impairment in personality functioning	7	566	0.78	0.50	0.011	AFG3L2, AP3B2, DNM1, EEF1A2, MDH2, STXBP1, TUBB3
Monarch	HP:0000348	High forehead	5	249	1.00	0.56	0.013	AP3B2, DNM1, EEF1A2, KRAS, STXBP1
Monarch	HP:0034057	Fetal anomaly	6	401	0.87	0.52	0.013	AP3B2, DNM1, EEF1A2, KRAS, STXBP1, SYNE1
Monarch	HP:0000002	Abnormality of body height	10	1253	0.59	0.42	0.013	AP3B2, DNM1, DYNC1H1, EEF1A2, FARSB, HK1, KRAS, MACF1, SEPTIN9, STXBP1
Monarch	EFO:0004784	Self reported educational attainment	9	1012	0.64	0.44	0.013	AP3B2, GRID2, ITSN1, MPP6, SEPTIN3, SGIP1, STXBP1, TUBB3, XRCC6
Monarch	HP:0000717	Autism	4	132	1.17	0.60	0.013	AP3B2, DNM1, EEF1A2, STXBP1
Monarch	HP:0001197	Abnormality of prenatal development or birth	8	786	0.70	0.46	0.013	ANK2, AP3B2, DNM1, EEF1A2, FARSB, KRAS, STXBP1, SYNE1
Monarch	HP:0011147	Typical absence seizure	3	50	1.47	0.65	0.013	DYNC1H1, EEF1A2, STXBP1
Monarch	HP:0002015	Dysphagia	6	413	0.85	0.51	0.014	AFG3L2, DNM1, DYNC1H1, EEF1A2, MACF1, STXBP1

Table S7: Cerebellar vermis pathways enriched in downregulated proteins (FDR-adjusted $p < 0.05$) of adults with idiopathic autism *(continued)*

Category	Term ID	Term description	Gene count	Back-ground	Strength	Signal	FDR	Matching proteins in network
Monarch	HP:0002059	Cerebral atrophy	7	603	0.76	0.47	0.015	AP3B2, DNM1, DYNC1H1, EEF1A2, FARSB, KRAS, STXBP1
Monarch	HP:0002069	Bilateral tonic-clonic seizure	5	263	0.97	0.54	0.015	AFG3L2, DNM1, DYNC1H1, EEF1A2, STXBP1
Monarch	HP:0000752	Hyperactivity	6	423	0.84	0.50	0.016	AP3B2, DNM1, EEF1A2, KRAS, MAP1B, STXBP1
Monarch	HP:0002119	Ventriculomegaly	7	612	0.75	0.46	0.016	DYNC1H1, EEF1A2, HK1, KRAS, STXBP1, TUBB2A, TUBB3
Monarch	HP:0007369	Atrophy/Degeneration affecting the cerebrum	7	619	0.75	0.45	0.017	AP3B2, DNM1, DYNC1H1, EEF1A2, FARSB, KRAS, STXBP1
Monarch	HP:0000543	Optic disc pallor	4	146	1.13	0.56	0.017	AFG3L2, AP3B2, GRID2, HK1
Monarch	HP:0001507	Growth abnormality	13	2182	0.47	0.35	0.018	AP3B2, DNM1, DYNC1H1, EEF1A2, FARSB, HK1, KRAS, MACF1, MDH2, SEPTIN9, STXBP1, SYNE1, TUBB3
Monarch	HP:0100710	Impulsivity	4	149	1.12	0.55	0.018	AP3B2, DNM1, EEF1A2, STXBP1
Monarch	HP:0000252	Microcephaly	9	1080	0.61	0.40	0.019	AP3B2, DNM1, DYNC1H1, EEF1A2, FARSB, MACF1, MAP1B, STXBP1, TUBB3
Monarch	HP:0000286	Epicanthus	6	447	0.82	0.47	0.020	EEF1A2, HK1, KRAS, MAP1B, SEPTIN9, TUBB3
Monarch	HP:0002317	Unsteady gait	4	153	1.11	0.54	0.020	AP3B2, DNM1, EEF1A2, STXBP1
Monarch	HP:0007018	Attention deficit hyperactivity disorder	5	284	0.94	0.50	0.020	AP3B2, DNM1, EEF1A2, MAP1B, STXBP1
Monarch	HP:0009121	Abnormal axial skeleton morphology	13	2206	0.46	0.34	0.020	AP3B2, DNM1, DYNC1H1, EEF1A2, FARSB, HK1, KRAS, MACF1, MAP1B, STXBP1, SYN1, SYNE1, TUBB3

Table S7: Cerebellar vermis pathways enriched in downregulated proteins (FDR-adjusted p < 0.05) of adults with idiopathic autism *(continued)*

Category	Term ID	Term description	Gene count	Back-ground	Strength	Signal	FDR	Matching proteins in network
Monarch	HP:0040195	Decreased head circumference	9	1088	0.61	0.40	0.020	AP3B2, DNM1, DYNC1H1, EEF1A2, FARSB, MACF1, MAP1B, STXBP1, TUBB3
Monarch	HP:0002121	Generalized non-motor (absence) seizure	4	155	1.10	0.54	0.020	DNM1, DYNC1H1, EEF1A2, STXBP1
Monarch	HP:0200136	Oral-pharyngeal dysphagia	3	61	1.38	0.58	0.020	DYNC1H1, EEF1A2, STXBP1
Monarch	HP:0001339	Lissencephaly	4	157	1.10	0.53	0.021	DYNC1H1, MACF1, STXBP1, TUBB3
Monarch	HP:0000668	Hypodontia	4	159	1.09	0.53	0.022	AP3B2, DNM1, EEF1A2, STXBP1
Monarch	EFO:0006842	Diabetes mellitus biomarker	7	658	0.72	0.43	0.023	GRID2, HK1, MACF1, NSF, SEPTIN9, SPTBN1, SYN2
Monarch	EFO:0009270	Heel bone mineral density	8	878	0.65	0.40	0.023	DNM3, MACF1, SEPTIN11, SEPTIN9, SGIP1, SPTBN1, SYN2, SYNE1
Monarch	EFO:0007660	Neuroticism measurement	5	298	0.92	0.48	0.023	DNM3, MPP6, NSF, SYN2, WDR1
Monarch	HP:0011842	Abnormal skeletal morphology	14	2573	0.43	0.32	0.023	AP3B2, DNM1, DYNC1H1, EEF1A2, FARSB, GRID2, HK1, KRAS, MACF1, MAP1B, STXBP1, SYN1, SYNE1, TUBB3
Monarch	HP:0000929	Abnormal skull morphology	12	1954	0.48	0.34	0.023	AP3B2, DNM1, DYNC1H1, EEF1A2, FARSB, HK1, KRAS, MACF1, MAP1B, STXBP1, SYN1, TUBB3
Monarch	HP:0025101	Dysgenesis of the hippocampus	2	12	1.91	0.60	0.023	STXBP1, TUBB3
Monarch	HP:0100716	Self-injurious behavior	4	163	1.08	0.52	0.023	DNM1, DYNC1H1, EEF1A2, STXBP1
Monarch	HP:0033259	Non-motor seizure	4	166	1.07	0.51	0.025	DNM1, DYNC1H1, EEF1A2, STXBP1

Table S7: Cerebellar vermis pathways enriched in downregulated proteins (FDR-adjusted $p < 0.05$) of adults with idiopathic autism *(continued)*

Category	Term ID	Term description	Gene count	Back-ground	Strength	Signal	FDR	Matching proteins in network
Monarch	HP:0001260	Dysarthria	6	480	0.79	0.43	0.026	AFG3L2, FAT2, GRID2, KRAS, SYNE1, TUBB3
Monarch	HP:0000486	Strabismus	8	903	0.64	0.39	0.026	GRID2, HK1, KRAS, MACF1, MDH2, STXBP1, SYNE1, TUBB3
Monarch	HP:0003202	Skeletal muscle atrophy	6	483	0.79	0.43	0.027	AFG3L2, DYNC1H1, HK1, MDH2, SEPTIN9, SYNE1
Monarch	HP:0006919	Abnormal aggressive, impulsive or violent behavior	5	313	0.90	0.46	0.027	DNM1, DYNC1H1, EEF1A2, STXBP1, SYN1
Monarch	HP:0000504	Abnormality of vision	9	1161	0.58	0.37	0.028	AFG3L2, AP3B2, DNM1, DYNC1H1, EEF1A2, HK1, KRAS, STXBP1, TUBB3
Monarch	HP:0001337	Tremor	6	492	0.78	0.42	0.029	AFG3L2, AP3B2, DNM1, EEF1A2, MDH2, STXBP1
Monarch	HP:0034353	Appendicular spasticity	5	324	0.88	0.44	0.031	AFG3L2, DYNC1H1, STXBP1, SYNE1, TUBB3
Monarch	HP:0002497	Spastic ataxia	2	15	1.82	0.55	0.032	AFG3L2, TUBB3
Monarch	HP:0011097	Epileptic spasm	4	182	1.03	0.47	0.032	DYNC1H1, EEF1A2, HK1, STXBP1
Monarch	HP:0001508	Failure to thrive	8	949	0.62	0.36	0.034	AP3B2, DNM1, EEF1A2, FARSB, KRAS, MDH2, STXBP1, TUBB3
Monarch	HP:0011146	Dialeptic seizure	4	186	1.02	0.46	0.035	DNM1, DYNC1H1, EEF1A2, STXBP1
Monarch	HP:0000570	Abnormal saccadic eye movements	3	79	1.27	0.49	0.036	AFG3L2, SYNE1, TUBB3
Monarch	HP:0012372	Abnormal eye morphology	12	2077	0.45	0.31	0.037	AFG3L2, AP3B2, DNM1, EEF1A2, FARSB, GRID2, HK1, KRAS, MDH2, SEPTIN9, STXBP1, TUBB3
Monarch	EFO:0004337	Intelligence	7	738	0.67	0.37	0.039	GRID2, LRRC40, MPP6, NSF, SEPTIN3, SEPTIN4, XRCC6

Table S7: Cerebellar vermis pathways enriched in downregulated proteins (FDR-adjusted p < 0.05) of adults with idiopathic autism *(continued)*

Category	Term ID	Term description	Gene count	Back-ground	Strength	Signal	FDR	Matching proteins in network
Monarch	HP:0002186	Apraxia	4	197	1.00	0.43	0.042	AFG3L2, GRID2, STXBP1, TUBB3
Monarch	HP:0002311	Incoordination	4	197	1.00	0.43	0.042	DYNC1H1, GRID2, STXBP1, SYNE1
Monarch	EFO:0005431	Illegal drug consumption	2	18	1.74	0.50	0.043	HSP90AA1, SEPTIN9
Monarch	HP:0000657	Oculomotor apraxia	3	85	1.24	0.46	0.043	AFG3L2, GRID2, TUBB3
Monarch	HP:0008956	Proximal lower limb amyotrophy	2	18	1.74	0.50	0.043	DYNC1H1, SYNE1
Monarch	HP:0000733	Motor stereotypy	4	201	0.99	0.43	0.044	DYNC1H1, EEF1A2, STXBP1, TUBB3
Monarch	HP:0000722	Obsessive-compulsive behavior	3	87	1.23	0.46	0.045	GRID2, MAP1B, STXBP1
Monarch	HP:0001321	Cerebellar hypoplasia	5	358	0.84	0.40	0.045	AFG3L2, DYNC1H1, KRAS, MACF1, TUBB3
Monarch	HP:0100704	Cerebral visual impairment	4	203	0.99	0.42	0.045	DYNC1H1, EEF1A2, HK1, STXBP1
Monarch	HP:0001441	Abnormality of the musculature of the thigh	2	19	1.71	0.49	0.045	DYNC1H1, SYNE1
Monarch	HP:0002118	Abnormal cerebral ventricle morphology	7	765	0.65	0.35	0.045	DYNC1H1, EEF1A2, HK1, KRAS, STXBP1, TUBB2A, TUBB3
Monarch	HP:0002384	Focal impaired awareness seizure	3	90	1.22	0.45	0.048	DYNC1H1, EEF1A2, STXBP1
Monarch	HP:0004325	Decreased body weight	9	1274	0.54	0.32	0.048	AP3B2, DNM1, EEF1A2, FARSB, KRAS, MDH2, STXBP1, SYNE1, TUBB3
Monarch	HP:0011153	Focal motor seizure	3	90	1.22	0.45	0.048	DYNC1H1, EEF1A2, STXBP1
Monarch	HP:0100491	Abnormality of lower limb joint	6	558	0.72	0.37	0.048	HK1, KRAS, MAP1B, STXBP1, SYNE1, TUBB3
Monarch	HP:0012704	Widened subarachnoid space	3	91	1.21	0.44	0.049	DYNC1H1, EEF1A2, STXBP1

Table S7: Cerebellar vermis pathways enriched in downregulated proteins (FDR-adjusted $p < 0.05$) of adults with idiopathic autism *(continued)*

Category	Term ID	Term description	Gene count	Back-ground	Strength	Signal	FDR	Matching proteins in network
DISEASES	DOID:331	Central nervous system disease	14	1199	0.76	0.71	2.4e-04	AFG3L2, AP3B2, DNM1, DYNC1H1, EEF1A2, FAT2, GRID2, HK1, MAP1B, MDH2, NSF, STXBP1, SYN1, SYNE1
DISEASES	DOID:863	Nervous system disease	18	2275	0.59	0.57	2.9e-04	AFG3L2, AP3B2, DNM1, DYNC1H1, EEF1A2, FAT2, GRID2, HK1, MACF1, MAP1B, MDH2, NSF, SEPTIN9, STXBP1, SYN1, SYNE1, TUBB2A, TUBB3
DISEASES	DOID:936	Brain disease	11	806	0.83	0.71	6.7e-04	AFG3L2, AP3B2, DNM1, EEF1A2, FAT2, GRID2, MDH2, NSF, STXBP1, SYN1, SYNE1
DISEASES	DOID:7	Disease of anatomical entity	24	4798	0.39	0.39	2.0e-03	AFG3L2, ANK2, AP3B2, DNM1, DYNC1H1, EEF1A2, FAT2, GRID2, HK1, KRAS, MACF1, MAP1B, MDH2, NSF, SEPTIN3, SEPTIN6, SEPTIN9, STXBP1, SYN1, SYNE1, TUBB2A, TUBB3, XRCC5, XRCC6
DISEASES	DOID:1826	Epilepsy	7	306	1.05	0.73	2.6e-03	AP3B2, DNM1, EEF1A2, MDH2, NSF, STXBP1, SYN1
DISEASES	DOID:1289	Neurodegenerative disease	7	481	0.86	0.42	0.038	AFG3L2, DYNC1H1, FAT2, GRID2, HK1, MAP1B, SYNE1
DISEASES	DOID:0050753	Cerebellar ataxia	4	108	1.26	0.46	0.046	AFG3L2, FAT2, GRID2, SYNE1
DISEASES	DOID:4	Disease	25	6291	0.29	0.25	0.046	AFG3L2, ANK2, AP3B2, DNM1, DYNC1H1, EEF1A2, FAT2, GRID2, HK1, KRAS, MACF1, MAP1B, MDH2, NSF, SEPTIN3, SEPTIN6, SEPTIN9, STXBP1, SYN1, SYN2, SYNE1, TUBB2A, TUBB3, XRCC5, XRCC6

Table S7: Cerebellar vermis pathways enriched in downregulated proteins (FDR-adjusted p < 0.05) of adults with idiopathic autism *(continued)*

Category	Term ID	Term description	Gene count	Back-ground	Strength	Signal	FDR	Matching proteins in network
TISSUES	BTO:0000142	Brain	35	5733	0.48	0.64	5.6e-11	AFG3L2, ANK2, AP3B2, DNM1, DNM3, DYNC1H1, EEF1A2, FARSB, FAT2, GRID2, HK1, HSP90AA1, ITSN1, KRAS, MACF1, MAP1B, MDH2, MPP1, NSF, SEPTIN11, SEPTIN3, SEPTIN4, SEPTIN6, SEPTIN9, SGIP1, SPTBN1, STXBP1, SYN1, SYN2, SYNE1, TAGLN3, TUBB2A, WDR1, XRCC5, XRCC6
TISSUES	BTO:0000058	Alimentary canal	18	2021	0.64	0.70	9.8e-06	AP3B2, CEP76, DYNC1H1, EEF1A2, HSP90AA1, KRAS, MACF1, MAP1B, MDH2, SEPTIN11, SEPTIN9, SPTBN1, TAGLN3, TUBB2A, TUBB3, WDR1, XRCC5, XRCC6
TISSUES	BTO:0000445	Cerebral lobe	12	759	0.89	0.97	9.8e-06	AFG3L2, AP3B2, DYNC1H1, EEF1A2, MACF1, MAP1B, MPP1, SEPTIN3, SEPTIN4, SGIP1, STXBP1, SYN1
TISSUES	BTO:0000233	Cerebral cortex	13	1118	0.76	0.77	4.8e-05	AFG3L2, AP3B2, DYNC1H1, EEF1A2, FARSB, MACF1, MAP1B, MPP1, SEPTIN3, SEPTIN4, SGIP1, STXBP1, SYN1
TISSUES	BTO:0000478	Forebrain	15	1534	0.68	0.70	4.8e-05	AFG3L2, AP3B2, DYNC1H1, EEF1A2, FARSB, MACF1, MAP1B, MPP1, NSF, SEPTIN3, SEPTIN4, SGIP1, STXBP1, SYN1, TAGLN3
TISSUES	BTO:0000484	Frontal lobe	6	113	1.42	1.32	4.8e-05	AFG3L2, DYNC1H1, MACF1, MAP1B, STXBP1, SYN1

Table S7: Cerebellar vermis pathways enriched in downregulated proteins (FDR-adjusted $p < 0.05$) of adults with idiopathic autism *(continued)*

Category	Term ID	Term description	Gene count	Back-ground	Strength	Signal	FDR	Matching proteins in network
TISSUES	BTO:0000511	Gastrointestinal tract	16	1816	0.64	0.66	4.8e-05	AP3B2, CEP76, DYNC1H1, EEF1A2, HSP90AA1, KRAS, MACF1, MAP1B, MDH2, SEPTIN11, SPTBN1, TAGLN3, TUBB2A, TUBB3, WDR1, XRCC6
TISSUES	BTO:0003914	Interstitial cell of Cajal	7	193	1.25	1.21	4.8e-05	HSP90AA1, MAP1B, SEPTIN11, TAGLN3, TUBB2A, TUBB3, WDR1
TISSUES	BTO:0001489	Whole body	39	13099	0.17	0.29	2.8e-04	AFG3L2, ANK2, AP3B2, CEP76, DNM1, DNM3, DYNC1H1, EEF1A2, FARSB, FAT2, GRID2, HK1, HSP90AA1, ITSN1, KRAS, LRRC40, MACF1, MAP1B, MDH2, MPP1, NSF, PSPC1, SEPTIN11, SEPTIN3, SEPTIN4, SEPTIN6, SEPTIN9, SGIP1, SPTBN1, STXBP1, SYN1, SYN2, SYNE1, TAGLN3, TUBB2A, TUBB3, WDR1, XRCC5, XRCC6
TISSUES	BTO:0000439	Eye	10	835	0.77	0.66	7.8e-04	AFG3L2, ANK2, DYNC1H1, HSP90AA1, MACF1, SEPTIN9, SPTBN1, TUBB2A, WDR1, XRCC6
TISSUES	BTO:0000132	Blood platelet	7	363	0.98	0.76	1.2e-03	DNM1, HSP90AA1, MAP1B, SEPTIN11, SEPTIN6, SPTBN1, WDR1
TISSUES	BTO:0000202	Sense organ	11	1124	0.68	0.57	1.4e-03	AFG3L2, ANK2, DYNC1H1, HSP90AA1, MACF1, MDH2, SEPTIN9, SPTBN1, TUBB2A, WDR1, XRCC6

Table S7: Cerebellar vermis pathways enriched in downregulated proteins (FDR-adjusted p < 0.05) of adults with idiopathic autism *(continued)*

Category	Term ID	Term description	Gene count	Back-ground	Strength	Signal	FDR	Matching proteins in network
TISSUES	BTO:0001491	Viscus	24	5378	0.34	0.36	1.9e-03	ANK2, AP3B2, CEP76, DYNC1H1, EEF1A2, HSP90AA1, ITSN1, KRAS, MACF1, MAP1B, MDH2, NSF, PSPC1, SEPTIN11, SEPTIN4, SEPTIN9, SPTBN1, SYNE1, TAGLN3, TUBB2A, TUBB3, WDR1, XRCC5, XRCC6
TISSUES	BTO:0000042	Animal	40	15148	0.11	0.24	3.2e-03	AFG3L2, ANK2, AP3B2, CEP76, DNM1, DNM3, DYNC1H1, EEF1A2, FARSB, FAT2, GRID2, HK1, HSP90AA1, ITSN1, KRAS, LRRC40, MACF1, MAP1B, MDH2, MPP1, MPP6, NSF, PSPC1, SEPTIN11, SEPTIN3, SEPTIN4, SEPTIN6, SEPTIN9, SGIP1, SPTBN1, STXBP1, SYN1, SYN2, SYNE1, TAGLN3, TUBB2A, TUBB3, WDR1, XRCC5, XRCC6
TISSUES	BTO:0000293	Occipital lobe	3	36	1.61	0.76	7.1e-03	EEF1A2, MAP1B, STXBP1
TISSUES	BTO:0000203	Respiratory system	12	1707	0.54	0.41	0.010	DYNC1H1, EEF1A2, HSP90AA1, ITSN1, KRAS, MACF1, MAP1B, MDH2, SEPTIN9, SPTBN1, XRCC5, XRCC6
TISSUES	BTO:0000421	Connective tissue	9	954	0.67	0.46	0.010	HSP90AA1, MAP1B, SEPTIN11, TAGLN3, TUBB2A, TUBB3, WDR1, XRCC5, XRCC6
TISSUES	BTO:0000562	Heart	8	738	0.73	0.48	0.010	DYNC1H1, HSP90AA1, MACF1, MDH2, SPTBN1, SYNE1, XRCC5, XRCC6

Table S7: Cerebellar vermis pathways enriched in downregulated proteins (FDR-adjusted p < 0.05) of adults with idiopathic autism *(continued)*

Category	Term ID	Term description	Gene count	Back-ground	Strength	Signal	FDR	Matching proteins in network
TISSUES	BTO:0001490	Other source	3	43	1.54	0.70	0.010	DYNC1H1, MACF1, SYNE1
TISSUES	BTO:0000759	Liver	13	2125	0.48	0.36	0.017	ANK2, DYNC1H1, HSP90AA1, MACF1, MAP1B, MDH2, NSF, PSPC1, SEPTIN9, SPTBN1, WDR1, XRCC5, XRCC6
TISSUES	BTO:0000379	Embryo	8	824	0.68	0.43	0.019	DYNC1H1, HK1, ITSN1, MACF1, MAP1B, PSPC1, SEPTIN11, SEPTIN6
TISSUES	BTO:0001374	Thymus	5	271	0.96	0.51	0.019	HSP90AA1, MPP1, SEPTIN6, SEPTIN9, XRCC6
TISSUES	BTO:0000648	Intestine	10	1349	0.56	0.37	0.023	AP3B2, DYNC1H1, EEF1A2, HSP90AA1, KRAS, MACF1, SEPTIN11, SPTBN1, WDR1, XRCC6
TISSUES	BTO:0000763	Lung	10	1395	0.55	0.35	0.029	DYNC1H1, EEF1A2, HSP90AA1, ITSN1, KRAS, MACF1, MAP1B, MDH2, SPTBN1, XRCC6
TISSUES	BTO:0000180	Cervical carcinoma cell	5	322	0.88	0.43	0.036	HSP90AA1, KRAS, MAP1B, XRCC5, XRCC6
TISSUES	BTO:0000174	Embryonic structure	13	2369	0.43	0.30	0.041	DYNC1H1, HK1, HSP90AA1, ITSN1, MACF1, MAP1B, MPP1, PSPC1, SEPTIN11, SEPTIN6, SYNE1, WDR1, XRCC6
TISSUES	BTO:0000255	Brain cell line	4	188	1.02	0.44	0.042	HSP90AA1, MAP1B, TUBB2A, TUBB3
TISSUES	BTO:0000580	Blood cancer cell	9	1234	0.56	0.32	0.047	HSP90AA1, MACF1, MAP1B, MDH2, MPP1, SEPTIN9, WDR1, XRCC5, XRCC6
TISSUES	BTO:0003092	Urinary system	9	1249	0.55	0.32	0.050	DYNC1H1, HSP90AA1, ITSN1, MACF1, MDH2, PSPC1, SYNE1, WDR1, XRCC6

Table S7: Cerebellar vermis pathways enriched in downregulated proteins (FDR-adjusted $p < 0.05$) of adults with idiopathic autism *(continued)*

Category	Term ID	Term description	Gene count	Back-ground	Strength	Signal	FDR	Matching proteins in network
COMPARTMENTS	GOCC:0005940	Septin ring	5	14	2.25	2.23	6.9e-07	SEPTIN11, SEPTIN3, SEPTIN4, SEPTIN6, SEPTIN9
COMPARTMENTS	GOCC:0031105	Septin complex	5	14	2.25	2.23	6.9e-07	SEPTIN11, SEPTIN3, SEPTIN4, SEPTIN6, SEPTIN9
COMPARTMENTS	GOCC:0005856	Cytoskeleton	17	1575	0.73	0.83	2.0e-06	ANK2, CEP76, DYNC1H1, MACF1, MAP1B, MPP1, SEPTIN11, SEPTIN3, SEPTIN4, SEPTIN6, SEPTIN9, SPTBN1, SYN1, SYNE1, TUBB2A, TUBB3, WDR1
COMPARTMENTS	GOCC:0030054	Cell junction	13	1053	0.78	0.80	4.4e-05	ANK2, DNM1, DNM3, FAT2, GRID2, ITSN1, KRAS, MACF1, SEPTIN3, SYN1, SYN2, SYNE1, WDR1
COMPARTMENTS	GOCC:0005938	Cell cortex	7	190	1.26	1.21	4.8e-05	ANK2, SEPTIN11, SEPTIN3, SEPTIN4, SEPTIN6, SEPTIN9, SPTBN1
COMPARTMENTS	GOCC:0030424	Axon	7	267	1.11	0.93	3.8e-04	GRID2, HSP90AA1, MAP1B, SEPTIN4, SPTBN1, SYN1, TUBB3
COMPARTMENTS	GOCC:0043232	Intracellular non-membrane-bounded organelle	20	3309	0.47	0.48	4.1e-04	ANK2, CEP76, DYNC1H1, MACF1, MAP1B, MPP1, PSPC1, SEPTIN11, SEPTIN3, SEPTIN4, SEPTIN6, SEPTIN9, SPTBN1, SYN1, SYNE1, TUBB2A, TUBB3, WDR1, XRCC5, XRCC6
COMPARTMENTS	GOCC:0045202	Synapse	8	493	0.90	0.71	1.4e-03	DNM1, DNM3, GRID2, ITSN1, SEPTIN3, SYN1, SYN2, SYNE1
COMPARTMENTS	GOCC:0005874	Microtubule	6	233	1.10	0.79	1.7e-03	DYNC1H1, MACF1, MAP1B, SEPTIN9, TUBB2A, TUBB3
COMPARTMENTS	GOCC:0099512	Supramolecular fiber	8	555	0.85	0.63	2.7e-03	ANK2, DYNC1H1, MACF1, MAP1B, SEPTIN9, SYNE1, TUBB2A, TUBB3

Table S7: Cerebellar vermis pathways enriched in downregulated proteins (FDR-adjusted $p < 0.05$) of adults with idiopathic autism *(continued)*

Category	Term ID	Term description	Gene count	Back-ground	Strength	Signal	FDR	Matching proteins in network
COMPARTMENTS	GOCC:0043005	Neuron projection	8	579	0.83	0.61	3.2e-03	DNM3, GRID2, HSP90AA1, MAP1B, SEPTIN4, SPTBN1, SYN1, TUBB3
COMPARTMENTS	GOCC:0043564	Ku70:Ku80 complex	2	2	2.69	0.92	3.7e-03	XRCC5, XRCC6
COMPARTMENTS	GOCC:0120025	Plasma membrane bounded cell projection	11	1285	0.62	0.48	5.3e-03	DNM3, GRID2, HSP90AA1, MACF1, MAP1B, SEPTIN4, SEPTIN6, SEPTIN9, SPTBN1, SYN1, TUBB3
COMPARTMENTS	GOCC:0043226	Organelle	33	10113	0.21	0.28	5.8e-03	AFG3L2, ANK2, CEP76, DNM1, DNM3, DYNC1H1, EEF1A2, HK1, HSP90AA1, ITSN1, KRAS, MACF1, MAP1B, MDH2, MPP1, NSF, PSPC1, SEPTIN11, SEPTIN3, SEPTIN4, SEPTIN6, SEPTIN9, SGIP1, SPTBN1, STXBP1, SYN1, SYN2, SYNE1, TUBB2A, TUBB3, WDR1, XRCC5, XRCC6
COMPARTMENTS	GOCC:0098793	Presynapse	5	189	1.11	0.68	5.8e-03	DNM1, ITSN1, SEPTIN3, SYN1, SYN2
COMPARTMENTS	GOCC:0098590	Plasma membrane region	8	676	0.77	0.53	6.6e-03	ANK2, DNM3, ITSN1, MACF1, SEPTIN6, SGIP1, SPTBN1, SYNE1

Table S7: Cerebellar vermis pathways enriched in downregulated proteins (FDR-adjusted $p < 0.05$) of adults with idiopathic autism *(continued)*

Category	Term ID	Term description	Gene count	Back-ground	Strength	Signal	FDR	Matching proteins in network
COMPARTMENTS	GOCC:0005622	Intracellular	35	11512	0.18	0.26	7.6e-03	AFG3L2, ANK2, AP3B2, CEP76, DNM3, DYNC1H1, EEF1A2, FARSB, HK1, HSP90AA1, ITSN1, KRAS, MACF1, MAP1B, MDH2, MPP1, NSF, PSPC1, SEPTIN11, SEPTIN3, SEPTIN4, SEPTIN6, SEPTIN9, SGIP1, SPTBN1, STXBP1, SYN1, SYN2, SYNE1, TAGLN3, TUBB2A, TUBB3, WDR1, XRCC5, XRCC6
COMPARTMENTS	GOCC:0097227	Sperm annulus	2	5	2.29	0.77	8.7e-03	SEPTIN4, SEPTIN6
COMPARTMENTS	GOCC:0015629	Actin cytoskeleton	6	363	0.91	0.56	9.2e-03	ANK2, MACF1, SEPTIN11, SEPTIN9, SPTBN1, WDR1
COMPARTMENTS	GOCC:0032807	DNA ligase IV complex	2	7	2.15	0.70	0.013	XRCC5, XRCC6
COMPARTMENTS	GOCC:0043229	Intracellular organelle	31	9609	0.20	0.25	0.017	AFG3L2, ANK2, CEP76, DYNC1H1, EEF1A2, HK1, HSP90AA1, ITSN1, KRAS, MACF1, MAP1B, MDH2, MPP1, NSF, PSPC1, SEPTIN11, SEPTIN3, SEPTIN4, SEPTIN6, SEPTIN9, SGIP1, SPTBN1, STXBP1, SYN1, SYN2, SYNE1, TUBB2A, TUBB3, WDR1, XRCC5, XRCC6

Table S7: Cerebellar vermis pathways enriched in downregulated proteins (FDR-adjusted $p < 0.05$) of adults with idiopathic autism *(continued)*

Category	Term ID	Term description	Gene count	Back-ground	Strength	Signal	FDR	Matching proteins in network
COMPARTMENTS	GOCC:0110165	Cellular anatomical entity	38	14060	0.12	0.22	0.017	AFG3L2, ANK2, AP3B2, CEP76, DNM1, DNM3, DYNC1H1, EEF1A2, FARSB, FAT2, GRID2, HK1, HSP90AA1, ITSN1, KRAS, MACF1, MAP1B, MDH2, MPP1, MPP6, NSF, PSPC1, SEPTIN11, SEPTIN3, SEPTIN4, SEPTIN6, SEPTIN9, SGIP1, SPTBN1, STXBP1, SYN1, SYN2, SYNE1, TUBB2A, TUBB3, WDR1, XRCC5, XRCC6
COMPARTMENTS	GOCC:0070418	DNA-dependent protein kinase complex	2	9	2.04	0.65	0.018	XRCC5, XRCC6
COMPARTMENTS	GOCC:0005737	Cytoplasm	28	8195	0.23	0.26	0.021	AFG3L2, ANK2, AP3B2, DNM3, DYNC1H1, EEF1A2, FARSB, HK1, HSP90AA1, ITSN1, KRAS, MACF1, MAP1B, MDH2, NSF, SEPTIN11, SEPTIN3, SEPTIN4, SEPTIN6, SEPTIN9, SGIP1, SPTBN1, STXBP1, SYN1, SYN2, SYNE1, WDR1, XRCC6
COMPARTMENTS	GOCC:0008091	Spectrin	2	12	1.91	0.58	0.027	ANK2, SPTBN1
COMPARTMENTS	GOCC:0000783	Nuclear telomere cap complex	2	13	1.88	0.56	0.030	XRCC5, XRCC6

Table S7: Cerebellar vermis pathways enriched in downregulated proteins (FDR-adjusted $p < 0.05$) of adults with idiopathic autism *(continued)*

Category	Term ID	Term description	Gene count	Back-ground	Strength	Signal	FDR	Matching proteins in network
COMPARTMENTS	GOCC:0016020	Membrane	22	5715	0.28	0.26	0.034	AFG3L2, ANK2, AP3B2, DNM3, EEF1A2, GRID2, HSP90AA1, ITSN1, KRAS, MACF1, MDH2, MPP6, NSF, SEPTIN4, SEPTIN6, SGIP1, SPTBN1, STXBP1, SYN1, SYN2, SYNE1, XRCC5
COMPARTMENTS	GOCC:0032991	Protein-containing complex	21	5325	0.29	0.26	0.036	AFG3L2, ANK2, AP3B2, CEP76, DNM1, DNM3, DYNC1H1, EEF1A2, FARSB, GRID2, HSP90AA1, SEPTIN11, SEPTIN3, SEPTIN4, SEPTIN6, SEPTIN9, SGIP1, SPTBN1, SYNE1, XRCC5, XRCC6
COMPARTMENTS	GOCC:0070419	Nonhomologous end joining complex	2	16	1.79	0.52	0.037	XRCC5, XRCC6
COMPARTMENTS	GOCC:0015630	Microtubule cytoskeleton	8	973	0.61	0.35	0.041	CEP76, DYNC1H1, MACF1, MAP1B, MPP1, SEPTIN9, TUBB2A, TUBB3
UniProt Keywords	KW-0206	Cytoskeleton	16	1235	0.80	0.96	6.1e-07	ANK2, CEP76, DNM1, DNM3, DYNC1H1, MACF1, MAP1B, SEPTIN11, SEPTIN3, SEPTIN6, SEPTIN9, SPTBN1, SYNE1, TUBB2A, TUBB3, WDR1
UniProt Keywords	KW-0342	GTP-binding	10	342	1.16	1.43	6.1e-07	DNM1, DNM3, EEF1A2, KRAS, SEPTIN11, SEPTIN3, SEPTIN6, SEPTIN9, TUBB2A, TUBB3

Table S7: Cerebellar vermis pathways enriched in downregulated proteins (FDR-adjusted p < 0.05) of adults with idiopathic autism *(continued)*

Category	Term ID	Term description	Gene count	Back-ground	Strength	Signal	FDR	Matching proteins in network
UniProt Keywords	KW-0547	Nucleotide-binding	18	1776	0.70	0.82	6.1e-07	AFG3L2, DNM1, DNM3, DYNC1H1, EEF1A2, FARSB, HK1, HSP90AA1, KRAS, NSF, SEPTIN11, SEPTIN3, SEPTIN6, SEPTIN9, TUBB2A, TUBB3, XRCC5, XRCC6
UniProt Keywords	KW-0597	Phosphoprotein	35	8122	0.33	0.44	6.1e-07	ANK2, AP3B2, CEP76, DNM1, DNM3, DYNC1H1, EEF1A2, GRID2, HK1, HSP90AA1, ITSN1, LRRC40, MACF1, MAP1B, MDH2, MPP1, MPP6, NSF, PSPC1, SEPTIN11, SEPTIN3, SEPTIN6, SEPTIN9, SGIP1, SPTBN1, STXBP1, SYN1, SYN2, SYNE1, TAGLN3, TUBB2A, TUBB3, WDR1, XRCC5, XRCC6
UniProt Keywords	KW-0007	Acetylation	20	3362	0.47	0.49	2.1e-04	DNM3, DYNC1H1, EEF1A2, HK1, HSP90AA1, KRAS, MACF1, MAP1B, MDH2, MPP1, NSF, PSPC1, SEPTIN11, SEPTIN6, SEPTIN9, SPTBN1, TUBB2A, WDR1, XRCC5, XRCC6
UniProt Keywords	KW-0493	Microtubule	7	280	1.09	0.97	2.1e-04	DNM1, DNM3, DYNC1H1, MACF1, MAP1B, TUBB2A, TUBB3
UniProt Keywords	KW-0770	Synapse	8	461	0.93	0.82	3.6e-04	ANK2, GRID2, ITSN1, MAP1B, SEPTIN11, SEPTIN3, SYN1, SYN2
UniProt Keywords	KW-0887	Epilepsy	6	212	1.14	0.96	3.6e-04	AP3B2, DNM1, EEF1A2, MDH2, STXBP1, SYN1

Table S7: Cerebellar vermis pathways enriched in downregulated proteins (FDR-adjusted $p < 0.05$) of adults with idiopathic autism *(continued)*

Category	Term ID	Term description	Gene count	Back-ground	Strength	Signal	FDR	Matching proteins in network
UniProt Keywords	KW-0965	Cell junction	10	809	0.78	0.71	3.6e-04	ANK2, FAT2, GRID2, ITSN1, MAP1B, SEPTIN11, SEPTIN3, SYN1, SYN2, WDR1
UniProt Keywords	KW-0963	Cytoplasm	24	5095	0.37	0.41	3.7e-04	ANK2, CEP76, DNM1, DNM3, DYNC1H1, FARSB, HK1, HSP90AA1, ITSN1, KRAS, MACF1, MAP1B, NSF, PSPC1, SEPTIN11, SEPTIN3, SEPTIN6, SEPTIN9, SPTBN1, STXBP1, SYNE1, TUBB2A, TUBB3, WDR1
UniProt Keywords	KW-0254	Endocytosis	5	128	1.28	1.02	4.0e-04	ANK2, DNM1, DNM3, ITSN1, SGIP1
UniProt Keywords	KW-0523	Neurodegeneration	7	349	0.99	0.85	4.0e-04	AFG3L2, DYNC1H1, FARSB, FAT2, GRID2, HK1, SYNE1
UniProt Keywords	KW-0009	Actin-binding	5	271	0.96	0.56	0.012	MACF1, SPTBN1, SYN1, SYNE1, WDR1
UniProt Keywords	KW-0772	Systemic lupus erythematosus	2	21	1.67	0.49	0.045	XRCC5, XRCC6
UniProt Keywords	KW-0966	Cell projection	8	1024	0.59	0.34	0.045	ITSN1, MACF1, MAP1B, MPP1, SEPTIN11, SEPTIN6, TUBB3, WDR1
UniProt Keywords	KW-0132	Cell division	5	384	0.81	0.39	0.045	DYNC1H1, SEPTIN11, SEPTIN3, SEPTIN6, SEPTIN9
UniProt Keywords	KW-0728	SH3 domain	4	222	0.95	0.42	0.045	ITSN1, MACF1, MPP1, MPP6
Pfam	PF01926	50S ribosome-binding GTPase	4	43	1.66	0.80	5.6e-03	SEPTIN11, SEPTIN3, SEPTIN6, SEPTIN9
Pfam	PF03730	Ku70/Ku80 C-terminal arm	2	2	2.69	0.59	0.028	XRCC5, XRCC6
Pfam	PF02078	Synapsin, N-terminal domain	2	3	2.52	0.57	0.031	SYN1, SYN2

Table S7: Cerebellar vermis pathways enriched in downregulated proteins (FDR-adjusted $p < 0.05$) of adults with idiopathic autism *(continued)*

Category	Term ID	Term description	Gene count	Back-ground	Strength	Signal	FDR	Matching proteins in network
Pfam	PF02750	Synapsin, ATP binding domain	2	3	2.52	0.57	0.031	SYN1, SYN2
Pfam	PF10581	Synapsin N-terminal	2	3	2.52	0.57	0.031	SYN1, SYN2
InterPro	IPR027417	P-loop containing nucleoside triphosphate hydrolase	13	872	0.87	0.85	7.6e-05	AFG3L2, DNM1, DNM3, DYNC1H1, EEF1A2, KRAS, MPP1, MPP6, NSF, SEPTIN11, SEPTIN3, SEPTIN6, SEPTIN9
InterPro	IPR016491	Septin	4	12	2.22	1.48	9.3e-05	SEPTIN11, SEPTIN3, SEPTIN6, SEPTIN9
InterPro	IPR030379	Septin-type guanine nucleotide-binding (G) domain	4	12	2.22	1.48	9.3e-05	SEPTIN11, SEPTIN3, SEPTIN6, SEPTIN9
InterPro	IPR001359	Synapsin	2	3	2.52	0.57	0.031	SYN1, SYN2
InterPro	IPR001589	Actinin-type actin-binding domain, conserved site	3	23	1.81	0.55	0.031	MACF1, SPTBN1, SYNE1
InterPro	IPR001715	Calponin homology domain	4	80	1.39	0.53	0.031	MACF1, SPTBN1, SYNE1, TAGLN3
InterPro	IPR002017	Spectrin repeat	3	22	1.83	0.56	0.031	MACF1, SPTBN1, SYNE1
InterPro	IPR005160	Ku70/Ku80 C-terminal arm	2	2	2.69	0.57	0.031	XRCC5, XRCC6
InterPro	IPR005161	Ku70/Ku80, N-terminal alpha/beta	2	2	2.69	0.57	0.031	XRCC5, XRCC6
InterPro	IPR006164	Ku70/Ku80 beta-barrel domain	2	2	2.69	0.57	0.031	XRCC5, XRCC6
InterPro	IPR018159	Spectrin/alpha-actinin	3	30	1.69	0.55	0.031	MACF1, SPTBN1, SYNE1
InterPro	IPR019735	Synapsin, conserved site	2	3	2.52	0.57	0.031	SYN1, SYN2
InterPro	IPR019736	Synapsin, phosphorylation site	2	3	2.52	0.57	0.031	SYN1, SYN2
InterPro	IPR020897	Synapsin, pre-ATP-grasp domain	2	3	2.52	0.57	0.031	SYN1, SYN2

Table S7: Cerebellar vermis pathways enriched in downregulated proteins (FDR-adjusted $p < 0.05$) of adults with idiopathic autism *(continued)*

Category	Term ID	Term description	Gene count	Back-ground	Strength	Signal	FDR	Matching proteins in network
InterPro	IPR020898	Synapsin, ATP-binding domain	2	3	2.52	0.57	0.031	SYN1, SYN2
InterPro	IPR036872	CH domain superfamily	4	81	1.39	0.53	0.031	MACF1, SPTBN1, SYNE1, TAGLN3
InterPro	IPR016194	SPOC-like, C-terminal domain superfamily	2	5	2.29	0.55	0.035	XRCC5, XRCC6
InterPro	IPR000375	Dynamin stalk domain	2	6	2.22	0.51	0.044	DNM1, DNM3
InterPro	IPR003130	Dynamin GTPase effector	2	6	2.22	0.51	0.044	DNM1, DNM3
InterPro	IPR019762	Dynamin, GTPase region, conserved site	2	6	2.22	0.51	0.044	DNM1, DNM3
InterPro	IPR020850	GTPase effector domain	2	6	2.22	0.51	0.044	DNM1, DNM3
InterPro	IPR001401	Dynamin, GTPase domain	2	7	2.15	0.50	0.047	DNM1, DNM3
InterPro	IPR022812	Dynamin	2	7	2.15	0.50	0.047	DNM1, DNM3
SMART	SM00033	Calponin homology domain	4	65	1.48	0.70	9.3e-03	MACF1, SPTBN1, SYNE1, TAGLN3
SMART	SM00559	Ku70 and Ku80 are 70kDa and 80kDa subunits of the Lupus Ku autoantigen	2	2	2.69	0.76	9.8e-03	XRCC5, XRCC6
SMART	SM00150	Spectrin repeats	3	33	1.65	0.66	0.014	MACF1, SPTBN1, SYNE1
SMART	SM00302	Dynamin GTPase effector domain	2	6	2.22	0.62	0.023	DNM1, DNM3
SMART	SM00053	Dynamin, GTPase	2	7	2.15	0.61	0.024	DNM1, DNM3

Table S8: Cerebellar vermis pathways enriched in upregulated proteins (FDR-adjusted $p < 0.05$) of adults with idiopathic autism

Category	Term ID	Term description	Gene count	Back-ground	Strength	Signal	FDR	Matching proteins in network
GO Process	GO:0045109	Intermediate filament organization	13	69	2.11	6.29	4.1e-20	DSP, KRT1, KRT14, KRT16, KRT17, KRT2, KRT5, KRT6A, KRT6B, KRT77, KRT78, KRT80, KRT9
GO Process	GO:0030216	Keratinocyte differentiation	13	138	1.81	4.63	3.5e-17	CSTA, DSP, KRT1, KRT10, KRT16, KRT17, KRT2, KRT5, KRT6A, KRT6B, KRT77, KRT78, KRT80
GO Process	GO:0043588	Skin development	15	266	1.58	3.81	3.5e-17	CSTA, DSP, JUP, KRT1, KRT10, KRT16, KRT17, KRT2, KRT5, KRT6A, KRT6B, KRT77, KRT78, KRT80, KRT9
GO Process	GO:0008544	Epidermis development	15	320	1.50	3.38	3.4e-16	CSTA, DSP, KRT1, KRT10, KRT14, KRT16, KRT17, KRT2, KRT5, KRT6A, KRT6B, KRT77, KRT78, KRT80, KRT9
GO Process	GO:0031424	Keratinization	10	83	1.91	4.16	1.2e-13	KRT1, KRT16, KRT17, KRT2, KRT5, KRT6A, KRT6B, KRT77, KRT78, KRT80
GO Process	GO:0030855	Epithelial cell differentiation	15	617	1.22	2.09	2.9e-12	CSTA, DSP, KRT1, KRT10, KRT14, KRT16, KRT17, KRT2, KRT5, KRT6A, KRT6B, KRT77, KRT78, KRT80, KRT9
GO Process	GO:0097435	Supramolecular fiber organization	14	570	1.22	2.02	2.7e-11	ANXA2, DSP, KRT1, KRT14, KRT16, KRT17, KRT2, KRT5, KRT6A, KRT6B, KRT77, KRT78, KRT80, KRT9
GO Process	GO:0060429	Epithelium development	16	1069	1.01	1.45	3.1e-10	CSTA, DSP, KRT1, KRT10, KRT14, KRT16, KRT17, KRT2, KRT5, KRT6A, KRT6B, KRT77, KRT78, KRT80, KRT9, VCL
GO Process	GO:0018149	Peptide cross-linking	5	37	1.96	2.00	4.7e-06	CSTA, DSP, KRT1, KRT10, KRT2

Table S8: Cerebellar vermis pathways enriched in upregulated proteins (FDR-adjusted $p < 0.05$) of adults with idiopathic autism *(continued)*

Category	Term ID	Term description	Gene count	Back-ground	Strength	Signal	FDR	Matching proteins in network
GO Process	GO:0048513	Animal organ development	19	3246	0.60	0.63	5.7e-06	ANXA2, ARG1, CSTA, DSP, JUP, KRT1, KRT10, KRT16, KRT17, KRT2, KRT5, KRT6A, KRT6B, KRT77, KRT78, KRT80, KRT9, MBP, PRDX3
GO Process	GO:0030154	Cell differentiation	19	3507	0.57	0.58	1.8e-05	ANXA2, CSTA, DSP, KRT1, KRT10, KRT14, KRT16, KRT17, KRT2, KRT5, KRT6A, KRT6B, KRT77, KRT78, KRT80, KRT9, PRDX3, VCAN, VCL
GO Process	GO:0048856	Anatomical structure development	22	5117	0.47	0.49	2.5e-05	ANXA2, ARG1, CSTA, DSP, JUP, KRT1, KRT10, KRT14, KRT16, KRT17, KRT2, KRT5, KRT6A, KRT6B, KRT77, KRT78, KRT80, KRT9, MBP, PRDX3, VCAN, VCL
GO Process	GO:0016043	Cellular component organization	22	5436	0.44	0.45	7.8e-05	ANXA2, CTSD, DSG1, DSP, GGCT, JUP, KRT1, KRT10, KRT14, KRT16, KRT17, KRT2, KRT5, KRT6A, KRT6B, KRT77, KRT78, KRT80, KRT9, MBP, PRDX3, VCL
GO Process	GO:0006996	Organelle organization	17	3470	0.52	0.48	6.5e-04	ANXA2, CTSD, DSP, GGCT, KRT1, KRT14, KRT16, KRT17, KRT2, KRT5, KRT6A, KRT6B, KRT77, KRT78, KRT80, KRT9, PRDX3
GO Process	GO:0032501	Multicellular organismal process	22	6490	0.36	0.35	1.9e-03	ALB, ANXA2, ARG1, DSG1, DSP, JUP, KRT1, KRT14, KRT16, KRT17, KRT2, KRT5, KRT6A, KRT6B, KRT77, KRT78, KRT80, KRT9, MBP, PRDX3, VCAN, VCL

Table S8: Cerebellar vermis pathways enriched in upregulated proteins (FDR-adjusted $p < 0.05$) of adults with idiopathic autism *(continued)*

Category	Term ID	Term description	Gene count	Back-ground	Strength	Signal	FDR	Matching proteins in network
GO Process	GO:0090136	Epithelial cell-cell adhesion	3	18	2.05	1.04	2.4e-03	DSP, JUP, VCL
GO Process	GO:0052548	Regulation of endopeptidase activity	6	414	0.99	0.55	0.018	ANXA2, CSTA, CTSD, MBP, PRDX3, SERPINB3
GO Process	GO:0051546	Keratinocyte migration	2	6	2.35	0.60	0.034	KRT16, KRT2
GO Process	GO:0086073	Bundle of His cell-Purkinje myocyte adhesion involved in cell communication	2	6	2.35	0.60	0.034	DSP, JUP
GO Process	GO:0030162	Regulation of proteolysis	7	739	0.81	0.41	0.042	ANXA2, CSTA, CTSD, ENO1, MBP, PRDX3, SERPINB3
GO Process	GO:0010756	Positive regulation of plasminogen activation	2	8	2.23	0.55	0.047	ANXA2, ENO1
GO Process	GO:0060135	Maternal process involved in female pregnancy	3	57	1.55	0.52	0.047	ARG1, DSG1, PRDX3
GO Function	GO:0005198	Structural molecule activity	18	776	1.20	2.21	7.2e-15	DSP, JUP, KRT1, KRT10, KRT14, KRT16, KRT17, KRT2, KRT5, KRT6A, KRT6B, KRT77, KRT78, KRT80, KRT9, MBP, VCAN, VCL
GO Function	GO:0030280	Structural constituent of skin epidermis	9	37	2.22	4.93	2.1e-14	KRT1, KRT10, KRT2, KRT5, KRT6A, KRT6B, KRT77, KRT78, KRT80
GO Function	GO:0005200	Structural constituent of cytoskeleton	8	107	1.71	2.62	6.6e-09	DSP, KRT14, KRT16, KRT2, KRT5, KRT6A, KRT6B, KRT9
GO Component	GO:0005882	Intermediate filament	15	207	1.69	4.50	5.0e-19	DSP, JUP, KRT1, KRT10, KRT14, KRT16, KRT17, KRT2, KRT5, KRT6A, KRT6B, KRT77, KRT78, KRT80, KRT9

Table S8: Cerebellar vermis pathways enriched in upregulated proteins (FDR-adjusted $p < 0.05$) of adults with idiopathic autism *(continued)*

Category	Term ID	Term description	Gene count	Back-ground	Strength	Signal	FDR	Matching proteins in network
GO Component	GO:0001533	Cornified envelope	11	59	2.10	5.65	1.1e-17	ANXA2, CSTA, DSG1, DSP, JUP, KRT1, KRT10, KRT16, KRT17, KRT2, KRT77
GO Component	GO:0045095	Keratin filament	12	100	1.91	5.06	1.5e-17	KRT1, KRT10, KRT14, KRT17, KRT2, KRT5, KRT6A, KRT6B, KRT77, KRT78, KRT80, KRT9
GO Component	GO:0099512	Supramolecular fiber	17	1000	1.06	1.70	1.3e-12	DSP, ENO1, JUP, KRT1, KRT10, KRT14, KRT16, KRT17, KRT2, KRT5, KRT6A, KRT6B, KRT77, KRT78, KRT80, KRT9, VCL
GO Component	GO:0070062	Extracellular exosome	21	2096	0.83	1.16	2.1e-12	ALB, ANXA2, CTSD, DSP, ENO1, GGCT, JUP, KRT1, KRT10, KRT14, KRT16, KRT2, KRT5, KRT6A, KRT6B, KRT77, KRT78, KRT9, PKM, SERPINB3, VCL
GO Component	GO:0005615	Extracellular space	24	3247	0.70	0.91	2.2e-12	ALB, ANXA2, ARG1, CSTA, CTSD, DSP, ENO1, GGCT, JUP, KRT1, KRT10, KRT14, KRT16, KRT2, KRT5, KRT6A, KRT6B, KRT77, KRT78, KRT9, PKM, SERPINB3, VCAN, VCL
GO Component	GO:0031982	Vesicle	24	3957	0.61	0.75	1.2e-10	ALB, ANXA2, ARG1, CTSD, DSG1, DSP, ENO1, GGCT, JUP, KRT1, KRT10, KRT14, KRT16, KRT2, KRT5, KRT6A, KRT6B, KRT77, KRT78, KRT9, PKM, PRDX3, SERPINB3, VCL

Table S8: Cerebellar vermis pathways enriched in upregulated proteins (FDR-adjusted $p < 0.05$) of adults with idiopathic autism *(continued)*

Category	Term ID	Term description	Gene count	Back-ground	Strength	Signal	FDR	Matching proteins in network
GO Component	GO:0005829	Cytosol	24	5438	0.48	0.54	1.3e-07	ANXA2, ARG1, CSTA, DSG1, ENO1, GGCT, JUP, KRT1, KRT10, KRT14, KRT16, KRT17, KRT2, KRT5, KRT6A, KRT6B, KRT77, KRT78, KRT80, KRT9, MBP, PKM, PRDX3, VCL
GO Component	GO:0101002	ficolin-1-rich granule	7	185	1.41	1.73	1.2e-06	CTSD, DSG1, DSP, JUP, KRT1, PKM, VCL
GO Component	GO:0034774	Secretory granule lumen	8	321	1.23	1.46	2.0e-06	ALB, ANXA2, ARG1, CTSD, JUP, PKM, SERPINB3, VCL
GO Component	GO:0030141	Secretory granule	11	873	0.93	1.06	2.2e-06	ALB, ANXA2, ARG1, CTSD, DSG1, DSP, JUP, KRT1, PKM, SERPINB3, VCL
GO Component	GO:0005856	Cytoskeleton	16	2369	0.66	0.71	2.7e-06	DSP, JUP, KRT1, KRT10, KRT14, KRT16, KRT17, KRT2, KRT5, KRT6A, KRT6B, KRT77, KRT78, KRT80, KRT9, VCL
GO Component	GO:0030057	Desmosome	4	25	2.04	1.95	7.8e-06	DSG1, DSP, JUP, VCL
GO Component	GO:0005916	Fascia adherens	3	9	2.35	1.72	5.1e-05	DSP, JUP, VCL
GO Component	GO:0005737	Cytoplasm	29	12056	0.21	0.30	5.2e-05	ALB, ANXA2, ARG1, CSTA, CTSD, DSG1, DSP, ENO1, GGCT, JUP, KRT1, KRT10, KRT14, KRT16, KRT17, KRT2, KRT5, KRT6A, KRT6B, KRT77, KRT78, KRT80, KRT9, MBP, PKM, PRDX3, SERPINB3, VCAN, VCL
GO Component	GO:1904813	ficolin-1-rich granule lumen	5	124	1.44	1.32	8.8e-05	CTSD, JUP, KRT1, PKM, VCL
GO Component	GO:0035580	Specific granule lumen	4	62	1.64	1.34	1.9e-04	ARG1, CTSD, JUP, VCL
GO Component	GO:0005775	Vacuolar lumen	5	175	1.29	1.06	4.2e-04	ANXA2, ARG1, CTSD, SERPINB3, VCAN

Table S8: Cerebellar vermis pathways enriched in upregulated proteins (FDR-adjusted $p < 0.05$) of adults with idiopathic autism *(continued)*

Category	Term ID	Term description	Gene count	Back-ground	Strength	Signal	FDR	Matching proteins in network
GO Component	GO:0043226	Organelle	29	14017	0.15	0.24	3.6e-03	ALB, ANXA2, ARG1, CSTA, CTSD, DSG1, DSP, ENO1, GGCT, JUP, KRT1, KRT10, KRT14, KRT16, KRT17, KRT2, KRT5, KRT6A, KRT6B, KRT77, KRT78, KRT80, KRT9, MBP, PKM, PRDX3, SERPINB3, VCAN, VCL
GO Component	GO:0043232	Intracellular non-membrane-bounded organelle	18	5191	0.37	0.35	3.8e-03	ANXA2, DSP, ENO1, JUP, KRT1, KRT10, KRT14, KRT16, KRT17, KRT2, KRT5, KRT6A, KRT6B, KRT77, KRT78, KRT80, KRT9, VCL
GO Component	GO:0031410	Cytoplasmic vesicle	12	2482	0.52	0.41	6.1e-03	ALB, ANXA2, ARG1, CTSD, DSG1, DSP, JUP, KRT1, PKM, PRDX3, SERPINB3, VCL
GO Component	GO:0005915	Zonula adherens	2	9	2.18	0.89	6.3e-03	JUP, VCL
GO Component	GO:0043229	Intracellular organelle	28	13231	0.16	0.24	8.0e-03	ALB, ANXA2, ARG1, CSTA, CTSD, DSG1, DSP, ENO1, JUP, KRT1, KRT10, KRT14, KRT16, KRT17, KRT2, KRT5, KRT6A, KRT6B, KRT77, KRT78, KRT80, KRT9, MBP, PKM, PRDX3, SERPINB3, VCAN, VCL
GO Component	GO:0043218	Compact myelin	2	13	2.02	0.79	0.011	ANXA2, MBP
GO Component	GO:0062023	Collagen-containing extracellular matrix	5	407	0.92	0.54	0.015	ANXA2, CTSD, KRT1, PKM, VCAN
GO Component	GO:0035578	Azurophil granule lumen	3	91	1.35	0.64	0.017	ANXA2, ARG1, SERPINB3
GO Component	GO:0005911	Cell-cell junction	5	499	0.83	0.43	0.035	ANXA2, DSG1, DSP, JUP, VCL

Table S8: Cerebellar vermis pathways enriched in upregulated proteins (FDR-adjusted $p < 0.05$) of adults with idiopathic autism *(continued)*

Category	Term ID	Term description	Gene count	Back-ground	Strength	Signal	FDR	Matching proteins in network
GO Component	GO:0043227	Membrane-bounded organelle	27	13188	0.14	0.20	0.046	ALB, ANXA2, ARG1, CSTA, CTSD, DSG1, DSP, ENO1, GGCT, JUP, KRT1, KRT10, KRT14, KRT16, KRT2, KRT5, KRT6A, KRT6B, KRT77, KRT78, KRT9, MBP, PKM, PRDX3, SERPINB3, VCAN, VCL
STRING clusters	CL:34117	Formation of the cornified envelope, and Serpin, conserved site	15	105	1.99	6.46	7.7e-23	DSG1, DSP, JUP, KRT1, KRT10, KRT14, KRT16, KRT17, KRT2, KRT5, KRT6A, KRT6B, KRT77, KRT78, SERPINB3
STRING clusters	CL:34247	Mixed, incl. Pachyonychia congenita, and Epidermolysis bullosa simplex Dowling-Meara type	7	15	2.50	4.66	1.0e-12	KRT14, KRT16, KRT17, KRT5, KRT6A, KRT6B, SERPINB3
STRING clusters	CL:34245	Mixed, incl. Pachyonychia congenita, and Netherton syndrome	8	41	2.12	4.15	2.5e-12	KRT14, KRT16, KRT17, KRT5, KRT6A, KRT6B, KRT78, SERPINB3
STRING clusters	CL:34187	Desmosome, and Ichthyosis vulgaris	7	27	2.25	3.98	2.3e-11	DSG1, DSP, JUP, KRT1, KRT10, KRT2, KRT77
STRING clusters	CL:34253	Pachyonychia congenita, and Epidermolysis bullosa simplex Dowling-Meara type	5	5	2.83	3.74	6.1e-10	KRT14, KRT16, KRT5, KRT6A, KRT6B

Table S8: Cerebellar vermis pathways enriched in upregulated proteins (FDR-adjusted p < 0.05) of adults with idiopathic autism *(continued)*

Category	Term ID	Term description	Gene count	Back-ground	Strength	Signal	FDR	Matching proteins in network
STRING clusters	CL:34189	Mixed, incl. Ichthyosis vulgaris, and Bullous congenital ichthyosiform erythroderma	5	11	2.49	3.21	7.9e-09	DSG1, KRT1, KRT10, KRT2, KRT77
STRING clusters	CL:34218	Cell adhesive protein binding involved in bundle of His cell-Purkinje myocyte communication	2	5	2.43	0.75	0.015	DSP, JUP
STRING clusters	CL:34191	Ichthyosis vulgaris, and Epidermolytic acanthoma	2	6	2.35	0.71	0.019	KRT1, KRT10
KEGG	hsa05150	Staphylococcus aureus infection	6	86	1.68	1.99	1.3e-06	DSG1, KRT10, KRT14, KRT16, KRT17, KRT9
KEGG	hsa04915	Estrogen signaling pathway	6	133	1.49	1.62	7.7e-06	CTSD, KRT10, KRT14, KRT16, KRT17, KRT9
KEGG	hsa01230	Biosynthesis of amino acids	3	73	1.45	0.63	0.021	ARG1, ENO1, PKM
KEGG	hsa05146	Amoebiasis	3	101	1.30	0.52	0.040	ARG1, SERPINB3, VCL
Reactome	HSA-6809371	Formation of the cornified envelope	17	128	1.96	6.98	2.2e-26	CSTA, DSG1, DSP, JUP, KRT1, KRT10, KRT14, KRT16, KRT17, KRT2, KRT5, KRT6A, KRT6B, KRT77, KRT78, KRT80, KRT9
Reactome	HSA-1266738	Developmental Biology	18	1108	1.04	1.67	5.2e-13	CSTA, DSG1, DSP, JUP, KRT1, KRT10, KRT14, KRT16, KRT17, KRT2, KRT5, KRT6A, KRT6B, KRT77, KRT78, KRT80, KRT9, MBP
Reactome	HSA-6798695	Neutrophil degranulation	10	476	1.15	1.43	5.6e-07	ANXA2, ARG1, CTSD, DSG1, DSP, JUP, KRT1, PKM, SERPINB3, VCL

Table S8: Cerebellar vermis pathways enriched in upregulated proteins (FDR-adjusted $p < 0.05$) of adults with idiopathic autism *(continued)*

Category	Term ID	Term description	Gene count	Back-ground	Strength	Signal	FDR	Matching proteins in network
Monarch	HP:0000982	Palmoplantar keratoderma	15	145	1.85	5.39	1.9e-20	CSTA, DSG1, DSP, JUP, KRT1, KRT10, KRT14, KRT16, KRT17, KRT2, KRT5, KRT6A, KRT6B, KRT9, VCL
Monarch	HP:0008066	Abnormal blistering of the skin	13	76	2.07	6.23	2.0e-20	CSTA, DSG1, DSP, JUP, KRT1, KRT10, KRT14, KRT16, KRT17, KRT2, KRT5, KRT6A, KRT6B
Monarch	HP:0007446	Palmoplantar blistering	8	11	2.69	5.95	7.7e-16	DSG1, KRT1, KRT14, KRT16, KRT17, KRT5, KRT6A, KRT6B
Monarch	HP:0008404	Nail dystrophy	11	113	1.82	4.17	1.1e-14	CSTA, DSG1, DSP, JUP, KRT1, KRT14, KRT16, KRT17, KRT5, KRT6A, KRT6B
Monarch	HP:0033800	Blistering by anatomical location	8	20	2.43	5.22	1.9e-14	DSG1, KRT1, KRT14, KRT16, KRT17, KRT5, KRT6A, KRT6B
Monarch	HP:0000975	Hyperhidrosis	10	120	1.75	3.54	1.5e-12	DSG1, JUP, KRT1, KRT14, KRT16, KRT17, KRT5, KRT6A, KRT6B, KRT9
Monarch	HP:0007550	Hypohidrosis or hyperhidrosis	10	179	1.58	2.83	6.3e-11	DSG1, JUP, KRT1, KRT14, KRT16, KRT17, KRT5, KRT6A, KRT6B, KRT9
Monarch	HP:0001597	Abnormality of the nail	12	392	1.32	2.19	1.1e-10	CSTA, DSG1, DSP, JUP, KRT1, KRT14, KRT16, KRT17, KRT5, KRT6A, KRT6B, KRT9
Monarch	HP:0010783	Erythema	8	99	1.74	2.84	1.4e-09	CSTA, DSP, KRT1, KRT10, KRT14, KRT2, KRT5, KRT9
Monarch	HP:0001805	Onychogryposis	6	24	2.23	3.31	2.0e-09	KRT14, KRT16, KRT17, KRT5, KRT6A, KRT6B
Monarch	HP:0011124	Abnormal epidermal morphology	7	70	1.83	2.75	8.4e-09	CSTA, DSG1, DSP, JUP, KRT1, KRT10, KRT2
Monarch	HP:0012514	Lower limb pain	6	32	2.11	3.00	8.4e-09	KRT14, KRT16, KRT17, KRT5, KRT6A, KRT6B

Table S8: Cerebellar vermis pathways enriched in upregulated proteins (FDR-adjusted p < 0.05) of adults with idiopathic autism *(continued)*

Category	Term ID	Term description	Gene count	Back-ground	Strength	Signal	FDR	Matching proteins in network
Monarch	HP:0000695	Natal tooth	6	35	2.07	2.92	1.3e-08	DSP, JUP, KRT16, KRT17, KRT6A, KRT6B
Monarch	HP:0002814	Abnormality of the lower limb	16	1506	0.86	1.08	1.8e-08	ALB, CSTA, DSG1, DSP, JUP, KRT1, KRT10, KRT14, KRT16, KRT17, KRT2, KRT5, KRT6A, KRT6B, KRT9, VCL
Monarch	HP:0002164	Nail dysplasia	8	145	1.57	2.32	1.9e-08	DSP, KRT1, KRT14, KRT16, KRT17, KRT5, KRT6A, KRT6B
Monarch	HP:0025092	Epidermal acanthosis	6	39	2.02	2.82	2.0e-08	CSTA, DSG1, DSP, JUP, KRT1, KRT10
Monarch	HP:0001231	Abnormal fingernail morphology	8	156	1.54	2.24	2.8e-08	KRT1, KRT14, KRT16, KRT17, KRT5, KRT6A, KRT6B, KRT9
Monarch	HP:0008069	Neoplasm of the skin	8	175	1.49	2.09	6.3e-08	KRT1, KRT14, KRT16, KRT17, KRT5, KRT6A, KRT6B, KRT9
Monarch	HP:0011355	Localized skin lesion	12	738	1.04	1.34	8.6e-08	CSTA, DSP, JUP, KRT1, KRT10, KRT14, KRT16, KRT17, KRT2, KRT5, KRT6A, KRT6B
Monarch	HP:0012035	Steatocystoma multiplex	4	4	2.83	2.88	8.9e-08	KRT16, KRT17, KRT6A, KRT6B
Monarch	HP:0025248	Eruptive vellus hair cyst	4	4	2.83	2.88	8.9e-08	KRT16, KRT17, KRT6A, KRT6B
Monarch	HP:0200041	Skin erosion	5	21	2.21	2.70	9.9e-08	CSTA, DSP, JUP, KRT14, KRT5
Monarch	HP:0001596	Alopecia	8	193	1.45	1.98	1.1e-07	DSP, JUP, KRT14, KRT16, KRT17, KRT5, KRT6A, KRT6B
Monarch	HP:0007490	Linear arrays of macular hyperkeratoses in flexural areas	4	5	2.74	2.78	1.4e-07	KRT16, KRT17, KRT6A, KRT6B
Monarch	HP:0100792	Acantholysis	4	6	2.66	2.68	2.3e-07	DSG1, DSP, JUP, KRT2
Monarch	HP:0008401	Onychogryposis of toenails	4	7	2.59	2.60	3.6e-07	KRT16, KRT17, KRT6A, KRT6B

Table S8: Cerebellar vermis pathways enriched in upregulated proteins (FDR-adjusted $p < 0.05$) of adults with idiopathic autism *(continued)*

Category	Term ID	Term description	Gene count	Back-ground	Strength	Signal	FDR	Matching proteins in network
Monarch	HP:0030318	Angular cheilitis	4	7	2.59	2.60	3.6e-07	KRT16, KRT17, KRT6A, KRT6B
Monarch	HP:0001609	Hoarse voice	6	72	1.75	2.20	3.9e-07	KRT14, KRT16, KRT17, KRT5, KRT6A, KRT6B
Monarch	HP:0011362	Abnormal hair quantity	11	682	1.04	1.27	4.9e-07	DSG1, DSP, JUP, KRT1, KRT10, KRT14, KRT16, KRT17, KRT5, KRT6A, KRT6B
Monarch	HP:0030268	Hyperplastic callus formation	4	8	2.53	2.54	4.9e-07	KRT16, KRT17, KRT6A, KRT6B
Monarch	HP:0040036	Onychogryposis of fingernail	4	9	2.48	2.47	6.8e-07	KRT16, KRT17, KRT6A, KRT6B
Monarch	HP:0011138	Abnormality of skin adnexa morphology	13	1165	0.88	1.02	7.7e-07	CSTA, DSG1, DSP, JUP, KRT1, KRT10, KRT14, KRT16, KRT17, KRT5, KRT6A, KRT6B, KRT9
Monarch	HP:0001818	Paronychia	4	11	2.39	2.35	1.2e-06	KRT16, KRT17, KRT6A, KRT6B
Monarch	HP:0007410	Palmoplantar hyperhidrosis	4	12	2.35	2.30	1.6e-06	KRT16, KRT17, KRT6A, KRT6B
Monarch	HP:0200040	Epidermoid cyst	4	12	2.35	2.30	1.6e-06	KRT16, KRT17, KRT6A, KRT6B
Monarch	HP:0001802	Absent toenail	4	15	2.26	2.16	3.2e-06	DSP, JUP, KRT14, KRT5
Monarch	HP:0030766	Ear pain	4	15	2.26	2.16	3.2e-06	KRT16, KRT17, KRT6A, KRT6B
Monarch	HP:0011123	Inflammatory abnormality of the skin	9	479	1.11	1.25	4.4e-06	CSTA, DSG1, DSP, KRT1, KRT10, KRT14, KRT17, KRT5, KRT9
Monarch	HP:0100798	Fingernail dysplasia	4	17	2.20	2.09	4.6e-06	KRT16, KRT17, KRT6A, KRT6B
Monarch	HP:0011122	Abnormality of skin physiology	9	524	1.07	1.17	8.6e-06	CSTA, DSG1, DSP, KRT1, KRT10, KRT14, KRT17, KRT5, KRT9

Table S8: Cerebellar vermis pathways enriched in upregulated proteins (FDR-adjusted $p < 0.05$) of adults with idiopathic autism *(continued)*

Category	Term ID	Term description	Gene count	Back-ground	Strength	Signal	FDR	Matching proteins in network
Monarch	HP:0008064	Ichthyosis	6	133	1.49	1.61	8.7e-06	CSTA, DSP, KRT1, KRT10, KRT2, KRT9
Monarch	HP:0002745	Oral leukoplakia	4	21	2.11	1.96	8.7e-06	KRT16, KRT17, KRT6A, KRT6B
Monarch	HP:0007502	Follicular hyperkeratosis	4	21	2.11	1.96	8.7e-06	KRT16, KRT17, KRT6A, KRT6B
Monarch	HP:0025125	White lesion of the oral mucosa	4	21	2.11	1.96	8.7e-06	KRT16, KRT17, KRT6A, KRT6B
Monarch	HP:0007475	Congenital bullous ichthyosiform erythroderma	3	3	2.83	2.05	1.0e-05	KRT1, KRT10, KRT2
Monarch	HP:0001057	Aplasia cutis congenita	4	30	1.96	1.73	2.9e-05	DSP, JUP, KRT14, KRT5
Monarch	HP:0001030	Fragile skin	4	34	1.90	1.64	4.5e-05	DSP, JUP, KRT14, KRT5
Monarch	HP:0011830	Abnormal oral mucosa morphology	7	305	1.19	1.19	4.5e-05	DSP, KRT14, KRT16, KRT17, KRT5, KRT6A, KRT6B
Monarch	HP:0046506	Pain in head and neck region	4	35	1.89	1.63	4.9e-05	KRT16, KRT17, KRT6A, KRT6B
Monarch	HP:0001581	Recurrent skin infections	5	98	1.54	1.43	6.1e-05	DSG1, DSP, KRT1, KRT14, KRT5
Monarch	HP:0011793	Neoplasm by anatomical site	9	732	0.92	0.88	1.1e-04	KRT1, KRT10, KRT14, KRT16, KRT17, KRT5, KRT6A, KRT6B, KRT9
Monarch	HP:0004325	Decreased body weight	11	1274	0.77	0.72	1.5e-04	ALB, ARG1, DSP, KRT1, KRT10, KRT14, KRT16, KRT17, KRT5, KRT6A, KRT6B
Monarch	HP:0040162	Orthokeratosis	3	11	2.27	1.52	1.6e-04	CSTA, DSG1, DSP
Monarch	HP:0001019	Erythroderma	4	52	1.72	1.36	2.0e-04	DSG1, DSP, KRT1, KRT10
Monarch	HP:0002086	Abnormality of the respiratory system	12	1636	0.70	0.64	2.2e-04	ALB, CTSD, DSG1, DSP, JUP, KRT14, KRT16, KRT17, KRT5, KRT6A, KRT6B, VCL
Monarch	HP:0011354	Generalized abnormality of skin	9	821	0.87	0.79	2.7e-04	CSTA, DSP, JUP, KRT1, KRT10, KRT14, KRT2, KRT5, KRT9

Table S8: Cerebellar vermis pathways enriched in upregulated proteins (FDR-adjusted p < 0.05) of adults with idiopathic autism *(continued)*

Category	Term ID	Term description	Gene count	Back-ground	Strength	Signal	FDR	Matching proteins in network
Monarch	HP:0025142	Constitutional symptom	9	906	0.83	0.71	5.9e-04	ALB, DSP, JUP, KRT14, KRT16, KRT17, KRT5, KRT6A, KRT6B
Monarch	HP:0001507	Growth abnormality	13	2182	0.61	0.54	6.6e-04	ALB, ARG1, DSG1, DSP, JUP, KRT1, KRT10, KRT14, KRT16, KRT17, KRT5, KRT6A, KRT6B
Monarch	HP:0000992	Cutaneous photosensitivity	4	75	1.56	1.13	7.2e-04	KRT1, KRT10, KRT14, KRT5
Monarch	HP:0008065	Aplasia/Hypoplasia of the skin	5	170	1.30	1.01	7.2e-04	DSP, JUP, KRT14, KRT2, KRT5
Monarch	HP:0000118	Phenotypic abnormality	19	5129	0.40	0.39	1.2e-03	ALB, ARG1, CSTA, CTSD, DSG1, DSP, JUP, KRT1, KRT10, KRT14, KRT16, KRT17, KRT2, KRT5, KRT6A, KRT6B, KRT9, VCAN, VCL
Monarch	HP:0007438	Mottled pigmentation of the trunk and proximal extremities	2	2	2.83	1.15	1.6e-03	KRT14, KRT5
Monarch	HP:0007494	Discrete 2 to 5-mm hyper- and hypopigmented macules	2	2	2.83	1.15	1.6e-03	KRT14, KRT5
Monarch	HP:0010978	Abnormality of immune system physiology	10	1347	0.70	0.57	1.7e-03	ALB, CSTA, DSG1, DSP, KRT1, KRT10, KRT14, KRT17, KRT5, KRT9
Monarch	HP:0002715	Abnormality of the immune system	11	1682	0.65	0.53	1.8e-03	ALB, CSTA, DSG1, DSP, KRT1, KRT10, KRT14, KRT17, KRT5, KRT9, VCL
Monarch	HP:0010701	Abnormal immunoglobulin level	5	211	1.21	0.87	1.8e-03	ALB, KRT1, KRT14, KRT5, KRT9
Monarch	EFO:0003765	Sign or symptom	7	573	0.92	0.70	1.9e-03	ALB, KRT14, KRT16, KRT17, KRT5, KRT6A, KRT6B

Table S8: Cerebellar vermis pathways enriched in upregulated proteins (FDR-adjusted $p < 0.05$) of adults with idiopathic autism *(continued)*

Category	Term ID	Term description	Gene count	Back-ground	Strength	Signal	FDR	Matching proteins in network
Monarch	HP:0000164	Abnormality of the dentition	8	821	0.82	0.64	2.1e-03	DSP, JUP, KRT14, KRT16, KRT17, KRT5, KRT6A, KRT6B
Monarch	HP:0040189	Scaling skin	3	32	1.80	1.03	2.1e-03	CSTA, KRT1, KRT10
Monarch	HP:0006670	Impaired myocardial contractility	2	3	2.66	1.09	2.3e-03	DSP, JUP
Monarch	HP:0007497	Focal friction-related palmoplantar hyperkeratosis	2	3	2.66	1.09	2.3e-03	KRT14, KRT5
Monarch	HP:0007559	Localized epidermolytic hyperkeratosis	2	3	2.66	1.09	2.3e-03	KRT1, KRT9
Monarch	HP:0007599	Generalized reticulate brown pigmentation	2	3	2.66	1.09	2.3e-03	KRT14, KRT5
Monarch	HP:0005597	Congenital alopecia totalis	2	4	2.53	1.02	3.3e-03	DSP, JUP
Monarch	HP:0007530	Punctate palmoplantar hyperkeratosis	2	4	2.53	1.02	3.3e-03	KRT14, KRT5
Monarch	HP:0007585	Skin fragility with non-scarring blistering	2	4	2.53	1.02	3.3e-03	KRT14, KRT5
Monarch	HP:0008094	Widely spaced toes	2	4	2.53	1.02	3.3e-03	DSP, JUP
Monarch	HP:0010298	Smooth tongue	2	4	2.53	1.02	3.3e-03	KRT14, KRT5
Monarch	HP:0010705	4-5 finger syndactyly	2	4	2.53	1.02	3.3e-03	DSP, JUP
Monarch	HP:0025238	Foot pain	2	4	2.53	1.02	3.3e-03	KRT14, KRT5
Monarch	HP:0031464	Genital blistering	2	4	2.53	1.02	3.3e-03	KRT14, KRT5
Monarch	HP:0045059	Hyperkeratotic papule	2	4	2.53	1.02	3.3e-03	KRT14, KRT5
Monarch	HP:0003489	Acute episodes of neuropathic symptoms	2	5	2.43	0.97	4.3e-03	KRT14, KRT5
Monarch	HP:0007427	Reticulated skin pigmentation	2	5	2.43	0.97	4.3e-03	KRT14, KRT5
Monarch	HP:0007483	Depigmentation/hyperpigmentation of skin	2	5	2.43	0.97	4.3e-03	KRT14, KRT5
Monarch	HP:0007589	Aplasia cutis congenita on trunk or limbs	2	5	2.43	0.97	4.3e-03	KRT14, KRT5

Table S8: Cerebellar vermis pathways enriched in upregulated proteins (FDR-adjusted $p < 0.05$) of adults with idiopathic autism *(continued)*

Category	Term ID	Term description	Gene count	Back-ground	Strength	Signal	FDR	Matching proteins in network
Monarch	HP:0009719	Hypomelanotic macule	2	5	2.43	0.97	4.3e-03	KRT14, KRT5
Monarch	HP:0009884	Tapered distal phalanges of finger	2	5	2.43	0.97	4.3e-03	DSP, JUP
Monarch	HP:0031274	Hypovolemic shock	2	5	2.43	0.97	4.3e-03	DSP, JUP
Monarch	HP:0001508	Failure to thrive	8	949	0.76	0.55	4.7e-03	ARG1, DSP, KRT14, KRT16, KRT17, KRT5, KRT6A, KRT6B
Monarch	HP:0033127	Abnormality of the musculoskeletal system	14	3173	0.48	0.39	5.1e-03	ALB, ARG1, CTSD, DSP, JUP, KRT1, KRT10, KRT14, KRT16, KRT17, KRT5, KRT6A, KRT6B, VCL
Monarch	HP:0006097	3-4 finger syndactyly	2	6	2.35	0.93	5.3e-03	DSP, JUP
Monarch	HP:0030350	Erythematous papule	2	6	2.35	0.93	5.3e-03	KRT14, KRT5
Monarch	HP:0040111	Obsolete Bilateral external ear deformity	2	6	2.35	0.93	5.3e-03	DSP, JUP
Monarch	HP:0025354	Abnormal cellular phenotype	6	490	0.92	0.61	6.3e-03	ALB, DSP, KRT1, KRT14, KRT5, KRT9
Monarch	HP:0003228	Hypernatremia	2	7	2.29	0.89	6.6e-03	DSG1, DSP
Monarch	HP:0012513	Upper limb pain	2	7	2.29	0.89	6.6e-03	KRT14, KRT5
Monarch	HP:0000535	Obsolete Sparse and thin eyebrow	3	55	1.57	0.80	7.3e-03	DSP, JUP, KRT17
Monarch	HP:0011843	Abnormal musculoskeletal physiology	9	1330	0.66	0.48	7.4e-03	DSP, JUP, KRT1, KRT14, KRT16, KRT17, KRT5, KRT6A, KRT6B
Monarch	HP:0001626	Abnormality of the cardiovascular system	12	2438	0.52	0.40	7.6e-03	ALB, CSTA, DSG1, DSP, JUP, KRT1, KRT10, KRT14, KRT2, KRT5, KRT9, VCL
Monarch	HP:0005585	Spotty hyperpigmentation	2	8	2.23	0.85	8.0e-03	KRT14, KRT5
Monarch	HP:0005590	Spotty hypopigmentation	2	8	2.23	0.85	8.0e-03	KRT14, KRT5
Monarch	HP:0007447	Diffuse palmoplantar hyperkeratosis	2	8	2.23	0.85	8.0e-03	KRT1, KRT14

Table S8: Cerebellar vermis pathways enriched in upregulated proteins (FDR-adjusted $p < 0.05$) of adults with idiopathic autism *(continued)*

Category	Term ID	Term description	Gene count	Back-ground	Strength	Signal	FDR	Matching proteins in network
Monarch	HP:0011663	Right ventricular cardiomyopathy	2	8	2.23	0.85	8.0e-03	DSP, JUP
Monarch	HP:0031446	Erosion of oral mucosa	2	8	2.23	0.85	8.0e-03	KRT14, KRT5
Monarch	HP:0003341	Lamina lucida cleavage	2	9	2.18	0.82	9.3e-03	KRT14, KRT5
Monarch	HP:0004057	Mitten deformity	2	9	2.18	0.82	9.3e-03	DSP, JUP
Monarch	HP:0012531	Pain	6	539	0.88	0.56	9.3e-03	KRT14, KRT16, KRT17, KRT5, KRT6A, KRT6B
Monarch	HP:0032449	Abnormal dermoepidermal hemidesmosome morphology	2	9	2.18	0.82	9.3e-03	DSP, JUP
Monarch	HP:0200034	Papule	3	65	1.50	0.74	0.010	CSTA, KRT14, KRT5
Monarch	HP:0003073	Hypoalbuminemia	3	66	1.49	0.73	0.011	ALB, KRT14, KRT5
Monarch	HP:0012116	Abnormal circulating albumin concentration	3	67	1.48	0.72	0.011	ALB, KRT14, KRT5
Monarch	HP:0011842	Abnormal skeletal morphology	12	2573	0.50	0.38	0.011	ALB, CTSD, DSP, JUP, KRT1, KRT10, KRT14, KRT16, KRT17, KRT5, KRT6A, KRT6B
Monarch	HP:0001939	Abnormality of metabolism/homeostasis	11	2168	0.54	0.39	0.011	ALB, ARG1, CTSD, DSG1, DSP, KRT1, KRT14, KRT2, KRT5, KRT9, VCL
Monarch	HP:0002094	Dyspnea	5	350	0.99	0.59	0.011	KRT16, KRT17, KRT6A, KRT6B, VCL
Monarch	HP:0002098	Respiratory distress	4	183	1.17	0.65	0.011	KRT16, KRT17, KRT6A, KRT6B
Monarch	HP:0010702	Increased circulating antibody level	3	68	1.48	0.72	0.011	ALB, KRT1, KRT9
Monarch	HP:0002289	Alopecia universalis	2	11	2.09	0.78	0.012	DSP, JUP
Monarch	HP:0100780	Conjunctival hamartoma	2	11	2.09	0.78	0.012	KRT1, KRT10
Monarch	HP:0200097	Oral mucosal blisters	2	11	2.09	0.78	0.012	KRT14, KRT5

Table S8: Cerebellar vermis pathways enriched in upregulated proteins (FDR-adjusted $p < 0.05$) of adults with idiopathic autism *(continued)*

Category	Term ID	Term description	Gene count	Back-ground	Strength	Signal	FDR	Matching proteins in network
Monarch	HP:0011458	Abdominal symptom	9	1448	0.63	0.43	0.012	ARG1, KRT1, KRT10, KRT14, KRT16, KRT17, KRT5, KRT6A, KRT6B
Monarch	HP:0000271	Abnormality of the face	12	2641	0.49	0.37	0.013	ALB, CTSD, DSP, JUP, KRT1, KRT10, KRT14, KRT16, KRT17, KRT5, KRT6A, KRT6B
Monarch	HP:0001233	2-3 finger syndactyly	2	12	2.05	0.76	0.013	DSP, JUP
Monarch	HP:0002795	Abnormal respiratory system physiology	8	1151	0.67	0.45	0.014	CTSD, DSP, JUP, KRT16, KRT17, KRT6A, KRT6B, VCL
Monarch	HP:0006682	Premature ventricular contraction	2	13	2.02	0.73	0.015	DSP, JUP
Monarch	HP:0001806	Onycholysis	2	14	1.99	0.71	0.017	DSG1, JUP
Monarch	HP:0001836	Camptodactyly of toe	2	14	1.99	0.71	0.017	DSP, JUP
Monarch	HP:0002793	Abnormal pattern of respiration	6	624	0.82	0.49	0.018	CTSD, KRT16, KRT17, KRT6A, KRT6B, VCL
Monarch	HP:0025114	Hypergranulosis	2	17	1.90	0.65	0.024	DSG1, DSP
Monarch	HP:0002046	Heat intolerance	2	18	1.88	0.63	0.026	KRT14, KRT5
Monarch	HP:0006739	Squamous cell carcinoma of the skin	2	18	1.88	0.63	0.026	KRT14, KRT5
Monarch	HP:0100643	Abnormality of nail color	2	18	1.88	0.63	0.026	DSG1, DSP
Monarch	HP:0002780	Bronchomalacia	2	19	1.85	0.62	0.028	KRT14, KRT5
Monarch	HP:0000989	Pruritus	3	99	1.31	0.56	0.029	DSP, KRT14, KRT5
Monarch	HP:0001056	Milia	2	20	1.83	0.61	0.030	KRT14, KRT5
Monarch	HP:0002224	Woolly hair	2	20	1.83	0.61	0.030	DSP, JUP
Monarch	HP:0002597	Abnormality of the vasculature	9	1673	0.56	0.36	0.031	ALB, CSTA, DSP, KRT1, KRT10, KRT14, KRT2, KRT5, KRT9
Monarch	HP:0009125	Lipodystrophy	3	102	1.30	0.55	0.031	ALB, DSP, VCL
Monarch	HP:0005483	Abnormal epiglottis morphology	2	21	1.81	0.59	0.033	KRT14, KRT5

Table S8: Cerebellar vermis pathways enriched in upregulated proteins (FDR-adjusted $p < 0.05$) of adults with idiopathic autism *(continued)*

Category	Term ID	Term description	Gene count	Back-ground	Strength	Signal	FDR	Matching proteins in network
Monarch	HP:0032180	Abnormal circulating metabolite concentration	7	1010	0.67	0.39	0.034	ALB, ARG1, DSG1, DSP, KRT14, KRT5, VCL
Monarch	HP:0001807	Ridged nail	2	22	1.79	0.58	0.035	KRT14, KRT5
Monarch	HP:0002719	Recurrent infections	6	721	0.75	0.41	0.035	ALB, DSG1, DSP, KRT1, KRT14, KRT5
Monarch	HP:0003765	Psoriasiform dermatitis	2	22	1.79	0.58	0.035	DSG1, DSP
Monarch	HP:0010876	Abnormal circulating protein concentration	5	466	0.86	0.44	0.035	ALB, DSP, KRT14, KRT5, VCL
Monarch	HP:0001810	Dystrophic toenail	2	23	1.77	0.57	0.037	KRT14, KRT5
Monarch	HP:0200043	Verrucae	2	23	1.77	0.57	0.037	KRT1, KRT9
Monarch	HP:0032101	Unusual infection	6	748	0.74	0.39	0.041	ALB, DSG1, DSP, KRT1, KRT14, KRT5
Monarch	HP:0025032	Abnormality of digestive system physiology	9	1763	0.54	0.33	0.042	ARG1, KRT1, KRT10, KRT14, KRT16, KRT17, KRT5, KRT6A, KRT6B
Monarch	HP:0000561	Absent eyelashes	2	26	1.72	0.53	0.046	DSP, JUP
Monarch	HP:0001036	Parakeratosis	2	26	1.72	0.53	0.046	DSP, KRT5
Monarch	HP:0000502	Abnormal conjunctiva morphology	3	122	1.22	0.48	0.047	KRT1, KRT10, KRT14
DISEASES	DOID:161	Keratosis	12	59	2.14	6.13	4.0e-19	DSG1, DSP, JUP, KRT1, KRT14, KRT16, KRT17, KRT2, KRT5, KRT6A, KRT6B, KRT9
DISEASES	DOID:3390	Palmoplantar keratosis	11	46	2.21	6.06	3.2e-18	DSG1, DSP, JUP, KRT1, KRT14, KRT16, KRT17, KRT2, KRT6A, KRT6B, KRT9
DISEASES	DOID:37	Skin disease	16	518	1.32	2.63	5.0e-15	ALB, CSTA, DSG1, DSP, JUP, KRT1, KRT10, KRT14, KRT16, KRT17, KRT2, KRT5, KRT6A, KRT6B, KRT77, KRT9

Table S8: Cerebellar vermis pathways enriched in upregulated proteins (FDR-adjusted p < 0.05) of adults with idiopathic autism *(continued)*

Category	Term ID	Term description	Gene count	Back-ground	Strength	Signal	FDR	Matching proteins in network
DISEASES	DOID:0050428	Nonepidermolytic palmoplantar keratoderma	5	10	2.53	3.14	1.3e-08	DSP, JUP, KRT16, KRT6A, KRT6B
DISEASES	DOID:4603	Epidermolytic hyperkeratosis	4	4	2.83	2.73	2.0e-07	KRT1, KRT10, KRT2, KRT9
DISEASES	DOID:0050449	Pachyonychia congenita	4	5	2.74	2.65	3.1e-07	KRT16, KRT17, KRT6A, KRT6B
DISEASES	DOID:174	Acanthoma	4	9	2.48	2.33	1.5e-06	KRT1, KRT10, KRT14, KRT5
DISEASES	DOID:4159	Skin cancer	5	63	1.73	1.67	2.1e-05	KRT1, KRT14, KRT17, KRT5, KRT77
DISEASES	DOID:0060877	Bullous congenital ichthyosiform erythroderma	3	3	2.83	1.91	2.2e-05	KRT1, KRT10, KRT2
DISEASES	DOID:0080223	Epidermolytic palmoplantar keratoderma	3	3	2.83	1.91	2.2e-05	KRT1, KRT2, KRT9
DISEASES	DOID:0111556	Steatocystoma multiplex	3	3	2.83	1.91	2.2e-05	KRT17, KRT6A, KRT6B
DISEASES	DOID:2730	Epidermolysis bullosa	4	23	2.07	1.81	2.2e-05	DSG1, DSP, KRT14, KRT5
DISEASES	DOID:7039	Borst-Jadassohn intraepidermal carcinoma	3	3	2.83	1.91	2.2e-05	KRT1, KRT14, KRT5
DISEASES	DOID:2513	Basal cell carcinoma	4	27	2.00	1.74	2.9e-05	KRT1, KRT14, KRT17, KRT5
DISEASES	DOID:6498	Seborrheic keratosis	3	6	2.53	1.72	5.8e-05	KRT1, KRT14, KRT5
DISEASES	DOID:8691	Mycosis fungoides	3	6	2.53	1.72	5.8e-05	KRT1, KRT14, KRT5
DISEASES	DOID:7	Disease of anatomical entity	20	4798	0.45	0.46	1.0e-04	ALB, CSTA, CTSD, DSG1, DSP, JUP, KRT1, KRT10, KRT14, KRT16, KRT17, KRT2, KRT5, KRT6A, KRT6B, KRT77, KRT9, MBP, VCAN, VCL
DISEASES	DOID:8502	Bullous skin disease	4	40	1.83	1.50	1.0e-04	DSG1, DSP, KRT14, KRT5

Table S8: Cerebellar vermis pathways enriched in upregulated proteins (FDR-adjusted $p < 0.05$) of adults with idiopathic autism *(continued)*

Category	Term ID	Term description	Gene count	Back-ground	Strength	Signal	FDR	Matching proteins in network
DISEASES	DOID:0050736	Autosomal dominant disease	11	1386	0.73	0.65	3.9e-04	ALB, DSP, KRT1, KRT10, KRT14, KRT16, KRT17, KRT2, KRT5, KRT6A, KRT6B
DISEASES	DOID:0050639	Primary cutaneous amyloidosis	3	19	2.03	1.25	6.8e-04	KRT1, KRT14, KRT5
DISEASES	DOID:305	Carcinoma	6	307	1.12	0.90	7.8e-04	ALB, KRT1, KRT14, KRT17, KRT5, KRT77
DISEASES	DOID:9120	Amyloidosis	4	75	1.56	1.11	7.8e-04	ALB, KRT1, KRT14, KRT5
DISEASES	DOID:4	Disease	21	6291	0.36	0.36	1.3e-03	ALB, ARG1, CSTA, CTSD, DSG1, DSP, JUP, KRT1, KRT10, KRT14, KRT16, KRT17, KRT2, KRT5, KRT6A, KRT6B, KRT77, KRT9, MBP, VCAN, VCL
DISEASES	DOID:0060735	Epidermolysis bullosa simplex Dowling-Meara type	2	2	2.83	1.14	1.7e-03	KRT14, KRT5
DISEASES	DOID:0111346	Epidermolysis bullosa simplex with mottled pigmentation	2	2	2.83	1.14	1.7e-03	KRT14, KRT5
DISEASES	DOID:0111708	Focal nonepidermolytic palmoplantar keratoderma	2	2	2.83	1.14	1.7e-03	KRT16, KRT6A
DISEASES	DOID:4323	Epidermolytic acanthoma	2	2	2.83	1.14	1.7e-03	KRT1, KRT10
DISEASES	DOID:0080551	Naxos disease	2	6	2.35	0.90	6.4e-03	DSP, JUP
DISEASES	DOID:0050431	Arrhythmogenic right ventricular cardiomyopathy	2	15	1.96	0.63	0.028	DSP, JUP
DISEASES	DOID:9182	Pemphigus	2	15	1.96	0.63	0.028	DSG1, DSP

Table S8: Cerebellar vermis pathways enriched in upregulated proteins (FDR-adjusted $p < 0.05$) of adults with idiopathic autism *(continued)*

Category	Term ID	Term description	Gene count	Back-ground	Strength	Signal	FDR	Matching proteins in network
DISEASES	DOID:0050739	Autosomal genetic disease	12	2802	0.46	0.32	0.032	ALB, CTSD, DSP, KRT1, KRT10, KRT14, KRT16, KRT17, KRT2, KRT5, KRT6A, KRT6B
DISEASES	DOID:0050177	Monogenic disease	13	3266	0.43	0.31	0.033	ALB, CTSD, DSP, KRT1, KRT10, KRT14, KRT16, KRT17, KRT2, KRT5, KRT6A, KRT6B, VCAN
DISEASES	DOID:630	Genetic disease	14	3778	0.40	0.29	0.035	ALB, ARG1, CTSD, DSP, KRT1, KRT10, KRT14, KRT16, KRT17, KRT2, KRT5, KRT6A, KRT6B, VCAN
DISEASES	DOID:2914	Immune system disease	6	675	0.78	0.42	0.036	DSG1, DSP, KRT1, KRT14, KRT5, MBP
DISEASES	DOID:0060036	Intrinsic cardiomyopathy	3	109	1.27	0.48	0.050	DSP, JUP, VCL
TISSUES	BTO:0001253	Skin	19	1151	1.05	1.71	1.2e-13	ANXA2, CSTA, CTSD, DSG1, DSP, ENO1, GGCT, KRT1, KRT10, KRT14, KRT16, KRT17, KRT2, KRT5, KRT6A, KRT6B, KRT80, MBP, PKM
TISSUES	BTO:0000634	Integument	21	2112	0.83	1.14	1.0e-11	ALB, ANXA2, CSTA, CTSD, DSG1, DSP, ENO1, GGCT, KRT1, KRT10, KRT14, KRT16, KRT17, KRT2, KRT5, KRT6A, KRT6B, KRT80, MBP, PKM, VCL
TISSUES	BTO:0000420	Neck	9	264	1.36	1.86	1.0e-07	ALB, ANXA2, ENO1, KRT14, KRT17, KRT5, KRT6A, KRT6B, PKM
TISSUES	BTO:0000574	Hematopoietic cell	13	1019	0.94	1.12	3.8e-07	ALB, ANXA2, ARG1, ENO1, JUP, KRT1, KRT10, KRT14, KRT2, KRT5, KRT9, PKM, VCL

Table S8: Cerebellar vermis pathways enriched in upregulated proteins (FDR-adjusted p < 0.05) of adults with idiopathic autism *(continued)*

Category	Term ID	Term description	Gene count	Back-ground	Strength	Signal	FDR	Matching proteins in network
TISSUES	BTO:0000828	Throat	8	234	1.37	1.71	7.6e-07	ANXA2, ENO1, KRT14, KRT17, KRT5, KRT6A, KRT6B, PKM
TISSUES	BTO:0000203	Respiratory system	15	1707	0.78	0.87	9.3e-07	ALB, ANXA2, CTSD, DSP, ENO1, GGCT, JUP, KRT14, KRT17, KRT5, MBP, PKM, PRDX3, SERPINB3, VCAN
TISSUES	BTO:0000751	Leukocyte	12	924	0.95	1.11	9.3e-07	ALB, ANXA2, ENO1, JUP, KRT1, KRT10, KRT14, KRT2, KRT5, KRT9, PKM, VCL
TISSUES	BTO:0000763	Lung	14	1395	0.83	0.95	9.3e-07	ALB, ANXA2, CTSD, DSP, ENO1, GGCT, JUP, KRT14, KRT5, MBP, PKM, PRDX3, SERPINB3, VCAN
TISSUES	BTO:0000775	Lymphocyte	11	698	1.03	1.23	9.3e-07	ALB, ANXA2, ENO1, KRT1, KRT10, KRT14, KRT2, KRT5, KRT9, PKM, VCL
TISSUES	BTO:0001090	Mouth	10	532	1.11	1.34	9.3e-07	ANXA2, CSTA, DSG1, ENO1, KRT1, KRT14, KRT17, KRT5, KRT6A, KRT78
TISSUES	BTO:0001208	Larynx	5	37	1.96	2.24	9.3e-07	KRT14, KRT17, KRT5, KRT6A, KRT6B
TISSUES	BTO:0000089	Blood	15	1824	0.75	0.83	1.3e-06	ALB, ANXA2, ARG1, CTSD, ENO1, JUP, KRT1, KRT10, KRT14, KRT2, KRT5, KRT9, PKM, PRDX3, VCL
TISSUES	BTO:0000404	Epidermis	8	293	1.27	1.53	1.5e-06	CSTA, DSG1, KRT10, KRT14, KRT16, KRT2, KRT5, KRT6B
TISSUES	BTO:0001491	Viscus	23	5378	0.46	0.51	1.5e-06	ALB, ANXA2, ARG1, CSTA, CTSD, DSP, ENO1, GGCT, JUP, KRT1, KRT10, KRT14, KRT16, KRT17, KRT5, KRT6A, KRT78, MBP, PKM, PRDX3, SERPINB3, VCAN, VCL

Table S8: Cerebellar vermis pathways enriched in upregulated proteins (FDR-adjusted $p < 0.05$) of adults with idiopathic autism *(continued)*

Category	Term ID	Term description	Gene count	Back-ground	Strength	Signal	FDR	Matching proteins in network
TISSUES	BTO:0001470	Epidermal cell	7	197	1.38	1.64	2.2e-06	CSTA, DSG1, KRT10, KRT14, KRT16, KRT5, KRT6B
TISSUES	BTO:0002074	Stratified epithelium	3	3	2.83	2.09	8.1e-06	KRT10, KRT14, KRT5
TISSUES	BTO:0001049	Pharynx	5	75	1.66	1.68	1.4e-05	ANXA2, ENO1, KRT14, KRT5, KRT6A
TISSUES	BTO:0000988	Pancreas	9	626	0.99	1.02	2.5e-05	ANXA2, CTSD, DSP, ENO1, KRT14, KRT17, KRT5, KRT6A, PKM
TISSUES	BTO:0005810	Immune system	13	1664	0.72	0.73	2.6e-05	ALB, ANXA2, ARG1, CTSD, ENO1, KRT1, KRT10, KRT14, KRT2, KRT5, KRT9, PKM, VCL
TISSUES	BTO:0000345	Digestive gland	16	2881	0.58	0.57	5.2e-05	ALB, ANXA2, ARG1, CTSD, DSP, ENO1, JUP, KRT10, KRT14, KRT17, KRT5, KRT6A, PKM, PRDX3, VCAN, VCL
TISSUES	BTO:0004680	Stratum basale	3	8	2.41	1.73	5.2e-05	KRT10, KRT14, KRT5
TISSUES	BTO:0000667	Keratinocyte	5	113	1.48	1.36	7.8e-05	CSTA, DSG1, KRT10, KRT16, KRT6B
TISSUES	BTO:0000753	Lymphoid tissue	12	1600	0.71	0.67	1.1e-04	ALB, ANXA2, CTSD, ENO1, KRT1, KRT10, KRT14, KRT2, KRT5, KRT9, PKM, VCL
TISSUES	BTO:0001493	Trunk	10	1014	0.83	0.79	1.1e-04	ALB, ANXA2, ENO1, KRT14, KRT16, KRT17, KRT5, KRT6A, KRT6B, PKM
TISSUES	BTO:0000058	Alimentary canal	13	2021	0.64	0.60	1.7e-04	ALB, ANXA2, CTSD, DSP, ENO1, KRT14, KRT16, KRT5, KRT6A, KRT78, PKM, PRDX3, VCL
TISSUES	BTO:0002860	Oral mucosa	3	14	2.16	1.50	1.7e-04	DSG1, KRT1, KRT5
TISSUES	BTO:0000123	Bladder	6	282	1.16	1.02	2.7e-04	ALB, ANXA2, GGCT, KRT14, PRDX3, VCL

Table S8: Cerebellar vermis pathways enriched in upregulated proteins (FDR-adjusted p < 0.05) of adults with idiopathic autism *(continued)*

Category	Term ID	Term description	Gene count	Back-ground	Strength	Signal	FDR	Matching proteins in network
TISSUES	BTO:0000759	Liver	13	2125	0.62	0.57	2.7e-04	ALB, ANXA2, ARG1, CTSD, DSP, ENO1, JUP, KRT10, KRT14, KRT5, PKM, PRDX3, VCL
TISSUES	BTO:0001129	Prostate gland	7	476	1.00	0.87	3.7e-04	ANXA2, ENO1, KRT5, KRT6A, KRT80, PKM, VCL
TISSUES	BTO:0000886	Mucosa	6	340	1.08	0.88	6.9e-04	DSG1, ENO1, KRT1, KRT10, KRT14, KRT5
TISSUES	BTO:0000522	Gland	22	7004	0.33	0.35	8.2e-04	ALB, ANXA2, ARG1, CSTA, CTSD, DSP, ENO1, GGCT, JUP, KRT1, KRT10, KRT14, KRT16, KRT17, KRT5, KRT6A, KRT80, MBP, PKM, PRDX3, VCAN, VCL
TISSUES	BTO:0001488	Endocrine gland	21	6403	0.35	0.36	8.8e-04	ALB, ANXA2, ARG1, CSTA, CTSD, DSP, ENO1, JUP, KRT1, KRT10, KRT14, KRT16, KRT17, KRT5, KRT6A, KRT80, MBP, PKM, PRDX3, VCAN, VCL
TISSUES	BTO:0003099	Internal female genital organ	14	2804	0.53	0.48	8.9e-04	ALB, ANXA2, CTSD, ENO1, JUP, KRT10, KRT14, KRT17, KRT5, KRT6A, PKM, PRDX3, VCAN, VCL
TISSUES	BTO:0000959	Esophagus	4	109	1.40	0.98	1.4e-03	ENO1, KRT16, KRT5, KRT78
TISSUES	BTO:0000020	Abdomen	4	113	1.38	0.96	1.5e-03	ALB, ANXA2, ENO1, PKM
TISSUES	BTO:0000202	Sense organ	9	1124	0.74	0.60	1.5e-03	ANXA2, CSTA, ENO1, KRT1, KRT17, KRT5, KRT78, PKM, VCL
TISSUES	BTO:0001085	Vascular system	6	420	0.99	0.74	1.9e-03	ALB, ANXA2, ENO1, PKM, VCAN, VCL
TISSUES	BTO:0001422	Uterine endometrium	4	124	1.34	0.91	2.1e-03	ENO1, KRT10, KRT14, KRT5
TISSUES	BTO:0000551	Lung cancer cell	4	131	1.32	0.88	2.5e-03	JUP, KRT6A, KRT6B, PKM
TISSUES	BTO:0000939	Basal cell	2	5	2.43	1.06	2.5e-03	KRT14, KRT5

Table S8: Cerebellar vermis pathways enriched in upregulated proteins (FDR-adjusted $p < 0.05$) of adults with idiopathic autism *(continued)*

Category	Term ID	Term description	Gene count	Back-ground	Strength	Signal	FDR	Matching proteins in network
TISSUES	BTO:0003092	Urinary system	9	1249	0.69	0.54	2.9e-03	ALB, ANXA2, CTSD, ENO1, GGCT, MBP, PKM, PRDX3, VCL
TISSUES	BTO:0000416	Epithelium	7	697	0.83	0.62	3.0e-03	ALB, ANXA2, KRT10, KRT14, KRT5, KRT80, VCL
TISSUES	BTO:0001244	Urinary tract	9	1258	0.69	0.53	3.0e-03	ALB, ANXA2, CTSD, ENO1, GGCT, MBP, PKM, PRDX3, VCL
TISSUES	BTO:0001113	Prepuce	3	51	1.60	0.92	3.5e-03	DSG1, DSP, KRT10
TISSUES	BTO:0001486	Skeletal system	9	1307	0.67	0.51	3.8e-03	ALB, ANXA2, ARG1, CSTA, CTSD, ENO1, GGCT, PKM, PRDX3
TISSUES	BTO:0002309	Myoepithelial cell	2	7	2.29	0.98	3.8e-03	KRT14, KRT5
TISSUES	BTO:0000671	Kidney	8	1039	0.72	0.53	4.4e-03	ALB, ANXA2, CTSD, ENO1, GGCT, MBP, PKM, PRDX3
TISSUES	BTO:0001385	Tongue	4	161	1.23	0.78	4.4e-03	CSTA, KRT1, KRT17, KRT78
TISSUES	BTO:0000091	Ascites	3	61	1.52	0.86	4.6e-03	ANXA2, ENO1, PKM
TISSUES	BTO:0000140	Bone	4	165	1.22	0.77	4.6e-03	ALB, ANXA2, ENO1, PKM
TISSUES	BTO:0000180	Cervical carcinoma cell	5	322	1.02	0.69	4.6e-03	ANXA2, ENO1, KRT17, PKM, VCL
TISSUES	BTO:0000431	Excretory gland	9	1385	0.64	0.49	4.6e-03	ALB, ANXA2, CTSD, ENO1, GGCT, KRT16, MBP, PKM, PRDX3
TISSUES	BTO:0000887	Muscle	8	1070	0.71	0.52	4.6e-03	ALB, ARG1, ENO1, GGCT, KRT17, PKM, PRDX3, VCAN
TISSUES	BTO:0000975	Ovary	6	528	0.89	0.62	4.6e-03	ALB, ANXA2, ENO1, KRT6A, PKM, VCL

Table S8: Cerebellar vermis pathways enriched in upregulated proteins (FDR-adjusted $p < 0.05$) of adults with idiopathic autism *(continued)*

Category	Term ID	Term description	Gene count	Back-ground	Strength	Signal	FDR	Matching proteins in network
TISSUES	BTO:0001489	Whole body	28	13099	0.16	0.24	4.6e-03	ALB, ANXA2, ARG1, CSTA, CTSD, DSG1, DSP, ENO1, GGCT, JUP, KRT1, KRT10, KRT14, KRT16, KRT17, KRT2, KRT5, KRT6A, KRT6B, KRT78, KRT80, KRT9, MBP, PKM, PRDX3, SERPINB3, VCAN, VCL
TISSUES	BTO:0001702	Left atrium	3	60	1.53	0.86	4.6e-03	ALB, PKM, VCL
TISSUES	BTO:0001703	Right atrium	3	61	1.52	0.86	4.6e-03	ALB, PKM, VCL
TISSUES	BTO:0002922	Bronchial epithelial cell	2	9	2.18	0.94	4.6e-03	KRT14, KRT5
TISSUES	BTO:0001158	Rectum	3	65	1.50	0.84	5.0e-03	ALB, DSP, VCL
TISSUES	BTO:0001424	Uterus	8	1117	0.69	0.50	5.9e-03	ENO1, KRT10, KRT14, KRT17, KRT5, PKM, PRDX3, VCL
TISSUES	BTO:0000255	Brain cell line	4	188	1.16	0.72	6.1e-03	ALB, ENO1, PRDX3, VCL
TISSUES	BTO:0000132	Blood platelet	5	363	0.97	0.64	6.3e-03	ALB, CTSD, KRT1, PKM, VCL
TISSUES	BTO:0003914	Interstitial cell of Cajal	4	193	1.15	0.71	6.5e-03	ALB, ENO1, PKM, PRDX3
TISSUES	BTO:0001629	Left ventricle	3	78	1.42	0.77	7.6e-03	ALB, PKM, VCL
TISSUES	BTO:0001418	Urinary bladder	4	217	1.10	0.65	9.5e-03	ANXA2, GGCT, PRDX3, VCL
TISSUES	BTO:0003091	Urogenital system	20	7090	0.28	0.28	0.010	ALB, ANXA2, CTSD, DSG1, DSP, ENO1, GGCT, JUP, KRT10, KRT14, KRT16, KRT17, KRT5, KRT6A, KRT80, MBP, PKM, PRDX3, VCAN, VCL
TISSUES	BTO:0001243	Shoot	3	107	1.28	0.63	0.017	ALB, DSG1, MBP
TISSUES	BTO:0000586	Colonic cancer cell	4	280	0.99	0.52	0.022	ALB, ANXA2, ENO1, PKM
TISSUES	BTO:0000081	Reproductive system	18	6444	0.28	0.25	0.033	ALB, ANXA2, CTSD, DSG1, DSP, ENO1, JUP, KRT10, KRT14, KRT16, KRT17, KRT5, KRT6A, KRT80, PKM, PRDX3, VCAN, VCL

Table S8: Cerebellar vermis pathways enriched in upregulated proteins (FDR-adjusted $p < 0.05$) of adults with idiopathic autism *(continued)*

Category	Term ID	Term description	Gene count	Back-ground	Strength	Signal	FDR	Matching proteins in network
TISSUES	BTO:0000082	Male reproductive system	11	2838	0.42	0.29	0.045	ANXA2, DSG1, DSP, ENO1, KRT10, KRT5, KRT6A, KRT80, PKM, PRDX3, VCL
TISSUES	BTO:0000282	Head	18	6642	0.27	0.24	0.047	ALB, ANXA2, CSTA, CTSD, DSG1, ENO1, KRT1, KRT14, KRT17, KRT5, KRT6A, KRT78, KRT80, MBP, PKM, PRDX3, VCAN, VCL
TISSUES	BTO:0000771	Macroglia	2	39	1.54	0.52	0.047	MBP, PKM
TISSUES	BTO:0001102	Blood vessel	4	354	0.89	0.41	0.049	ALB, ANXA2, VCAN, VCL
COMPARTMENTS	GOCC:0005882	Intermediate filament	13	81	2.04	6.06	4.0e-20	KRT1, KRT10, KRT14, KRT16, KRT17, KRT2, KRT5, KRT6A, KRT6B, KRT77, KRT80, KRT9, MBP
COMPARTMENTS	GOCC:0045095	Keratin filament	10	29	2.37	6.36	3.9e-18	KRT1, KRT10, KRT14, KRT17, KRT2, KRT5, KRT6A, KRT6B, KRT77, KRT9
COMPARTMENTS	GOCC:0099512	Supramolecular fiber	14	555	1.23	2.11	5.6e-12	KRT1, KRT10, KRT14, KRT16, KRT17, KRT2, KRT5, KRT6A, KRT6B, KRT77, KRT80, KRT9, MBP, VCL
COMPARTMENTS	GOCC:0001533	Cornified envelope	7	43	2.04	3.46	2.1e-10	CSTA, DSG1, DSP, JUP, KRT1, KRT10, KRT2
COMPARTMENTS	GOCC:0005737	Cytoplasm	28	8195	0.37	0.44	9.9e-08	ALB, ANXA2, ARG1, CSTA, CTSD, DSG1, DSP, ENO1, GGCT, JUP, KRT1, KRT10, KRT14, KRT16, KRT17, KRT2, KRT5, KRT6A, KRT6B, KRT77, KRT80, KRT9, MBP, PKM, PRDX3, SERPINB3, VCAN, VCL

Table S8: Cerebellar vermis pathways enriched in upregulated proteins (FDR-adjusted $p < 0.05$) of adults with idiopathic autism *(continued)*

Category	Term ID	Term description	Gene count	Back-ground	Strength	Signal	FDR	Matching proteins in network
COMPARTMENTS	GOCC:0005856	Cytoskeleton	15	1575	0.81	0.95	2.2e-07	ALB, KRT1, KRT10, KRT14, KRT16, KRT17, KRT2, KRT5, KRT6A, KRT6B, KRT77, KRT80, KRT9, MBP, VCL
COMPARTMENTS	GOCC:0030141	Secretory granule	11	719	1.02	1.22	6.5e-07	ALB, ANXA2, ARG1, CTSD, DSG1, DSP, JUP, KRT1, PKM, SERPINB3, VCL
COMPARTMENTS	GOCC:0005829	Cytosol	18	3054	0.60	0.65	3.0e-06	ALB, CSTA, ENO1, GGCT, KRT1, KRT10, KRT14, KRT16, KRT17, KRT2, KRT5, KRT6B, KRT77, KRT80, KRT9, MBP, PKM, VCL
COMPARTMENTS	GOCC:0034774	Secretory granule lumen	7	241	1.30	1.42	9.2e-06	ALB, ANXA2, ARG1, CTSD, JUP, SERPINB3, VCL
COMPARTMENTS	GOCC:0030057	Desmosome	4	23	2.07	1.93	9.6e-06	DSG1, DSP, JUP, VCL
COMPARTMENTS	GOCC:0043226	Organelle	28	10113	0.27	0.35	1.5e-05	ALB, ANXA2, ARG1, CSTA, CTSD, DSG1, DSP, ENO1, GGCT, JUP, KRT1, KRT10, KRT14, KRT16, KRT17, KRT2, KRT5, KRT6A, KRT6B, KRT77, KRT80, KRT9, MBP, PKM, PRDX3, SERPINB3, VCAN, VCL
COMPARTMENTS	GOCC:0005622	Intracellular	29	11512	0.23	0.32	2.1e-05	ALB, ANXA2, ARG1, CSTA, CTSD, DSG1, DSP, ENO1, GGCT, JUP, KRT1, KRT10, KRT14, KRT16, KRT17, KRT2, KRT5, KRT6A, KRT6B, KRT77, KRT78, KRT80, KRT9, MBP, PKM, PRDX3, SERPINB3, VCAN, VCL
COMPARTMENTS	GOCC:0005916	Fascia adherens	3	6	2.53	1.85	2.7e-05	DSP, JUP, VCL

Table S8: Cerebellar vermis pathways enriched in upregulated proteins (FDR-adjusted $p < 0.05$) of adults with idiopathic autism *(continued)*

Category	Term ID	Term description	Gene count	Back-ground	Strength	Signal	FDR	Matching proteins in network
COMPARTMENTS	GOCC:0043229	Intracellular organelle	27	9609	0.28	0.35	4.9e-05	ALB, ANXA2, ARG1, CSTA, CTSD, DSG1, DSP, ENO1, JUP, KRT1, KRT10, KRT14, KRT16, KRT17, KRT2, KRT5, KRT6A, KRT6B, KRT77, KRT80, KRT9, MBP, PKM, PRDX3, SERPINB3, VCAN, VCL
COMPARTMENTS	GOCC:0031982	Vesicle	14	2125	0.65	0.64	5.0e-05	ALB, ANXA2, ARG1, CTSD, DSG1, DSP, ENO1, JUP, KRT1, KRT5, PKM, PRDX3, SERPINB3, VCL
COMPARTMENTS	GOCC:0005615	Extracellular space	10	1027	0.82	0.78	1.2e-04	ALB, ANXA2, ARG1, CSTA, CTSD, ENO1, KRT1, KRT5, PKM, VCL
COMPARTMENTS	GOCC:1904813	ficolin-1-rich granule lumen	5	124	1.44	1.29	1.2e-04	CTSD, JUP, KRT1, PKM, VCL
COMPARTMENTS	GOCC:0035580	Specific granule lumen	4	62	1.64	1.31	2.3e-04	ARG1, CTSD, JUP, VCL
COMPARTMENTS	GOCC:0031410	Cytoplasmic vesicle	12	1738	0.67	0.62	2.4e-04	ALB, ANXA2, ARG1, CTSD, DSG1, DSP, JUP, KRT1, PKM, PRDX3, SERPINB3, VCL
COMPARTMENTS	GOCC:0043232	Intracellular non-membrane-bounded organelle	16	3309	0.52	0.49	2.4e-04	ALB, ANXA2, KRT1, KRT10, KRT14, KRT16, KRT17, KRT2, KRT5, KRT6A, KRT6B, KRT77, KRT80, KRT9, MBP, VCL
COMPARTMENTS	GOCC:0005775	Vacuolar lumen	5	168	1.31	1.09	3.5e-04	ANXA2, ARG1, CTSD, SERPINB3, VCAN
COMPARTMENTS	GOCC:0065010	Extracellular membrane-bounded organelle	7	473	1.00	0.88	3.5e-04	ALB, ANXA2, CTSD, ENO1, KRT5, PKM, VCL
COMPARTMENTS	GOCC:1903561	Extracellular vesicle	7	500	0.98	0.84	4.7e-04	ALB, ANXA2, CTSD, ENO1, KRT5, PKM, VCL

Table S8: Cerebellar vermis pathways enriched in upregulated proteins (FDR-adjusted $p < 0.05$) of adults with idiopathic autism *(continued)*

Category	Term ID	Term description	Gene count	Back-ground	Strength	Signal	FDR	Matching proteins in network
COMPARTMENTS	GOCC:0070062	Extracellular exosome	6	428	0.98	0.73	2.2e-03	ALB, ANXA2, CTSD, ENO1, PKM, VCL
COMPARTMENTS	GOCC:0012505	Endomembrane system	14	3156	0.48	0.41	3.1e-03	ALB, ANXA2, ARG1, CTSD, DSG1, DSP, ENO1, JUP, KRT1, PKM, PRDX3, SERPINB3, VCAN, VCL
COMPARTMENTS	GOCC:0005915	Zonula adherens	2	8	2.23	0.92	5.4e-03	JUP, VCL
COMPARTMENTS	GOCC:0005576	Extracellular region	11	2079	0.56	0.43	5.8e-03	ALB, ANXA2, ARG1, CSTA, CTSD, ENO1, KRT1, KRT5, PKM, VCAN, VCL
COMPARTMENTS	GOCC:0062023	Collagen-containing extracellular matrix	4	198	1.14	0.64	0.011	ANXA2, KRT1, PKM, VCAN
COMPARTMENTS	GOCC:0035578	Azurophil granule lumen	3	91	1.35	0.63	0.019	ANXA2, ARG1, SERPINB3
COMPARTMENTS	GOCC:1904090	Peptidase inhibitor complex	2	17	1.90	0.69	0.019	ALB, CSTA
COMPARTMENTS	GOCC:0030312	External encapsulating structure	4	259	1.02	0.50	0.029	ALB, KRT1, PKM, VCAN
UniProt Keywords	KW-0403	Intermediate filament	13	75	2.07	6.40	4.7e-21	KRT1, KRT10, KRT14, KRT16, KRT17, KRT2, KRT5, KRT6A, KRT6B, KRT77, KRT78, KRT80, KRT9
UniProt Keywords	KW-0416	Keratin	13	144	1.79	4.70	6.3e-18	KRT1, KRT10, KRT14, KRT16, KRT17, KRT2, KRT5, KRT6A, KRT6B, KRT77, KRT78, KRT80, KRT9
UniProt Keywords	KW-1007	Palmoplantar keratoderma	10	36	2.28	6.10	7.2e-18	DSG1, DSP, JUP, KRT1, KRT14, KRT16, KRT17, KRT6A, KRT6B, KRT9
UniProt Keywords	KW-0038	Ectodermal dysplasia	5	50	1.83	2.01	2.7e-06	KRT14, KRT16, KRT17, KRT6A, KRT6B

Table S8: Cerebellar vermis pathways enriched in upregulated proteins (FDR-adjusted p < 0.05) of adults with idiopathic autism *(continued)*

Category	Term ID	Term description	Gene count	Back-ground	Strength	Signal	FDR	Matching proteins in network
UniProt Keywords	KW-0175	Coiled coil	14	2166	0.64	0.62	8.1e-05	DSP, KRT1, KRT10, KRT14, KRT16, KRT17, KRT2, KRT5, KRT6A, KRT6B, KRT77, KRT78, KRT80, KRT9
UniProt Keywords	KW-0977	Ichthyosis	4	44	1.79	1.52	8.1e-05	CSTA, KRT1, KRT10, KRT2
UniProt Keywords	KW-0263	Epidermolysis bullosa	3	16	2.11	1.42	2.6e-04	DSP, KRT14, KRT5
UniProt Keywords	KW-0164	Citrullination	3	66	1.49	0.72	0.012	KRT1, KRT77, MBP
UniProt Keywords	KW-0122	Cardiomyopathy	3	87	1.37	0.60	0.023	DSP, JUP, VCL
UniProt Keywords	KW-0488	Methylation	7	973	0.69	0.40	0.029	ALB, DSP, KRT1, KRT2, KRT77, MBP, PKM
UniProt Keywords	KW-0597	Phosphoprotein	21	8122	0.24	0.23	0.041	ALB, ANXA2, ARG1, DSG1, DSP, ENO1, GGCT, JUP, KRT1, KRT10, KRT14, KRT17, KRT2, KRT5, KRT80, KRT9, MBP, PKM, PRDX3, VCAN, VCL
Pfam	PF16208	Keratin type II head	7	25	2.28	3.89	5.1e-11	KRT1, KRT2, KRT5, KRT6A, KRT6B, KRT77, KRT78
InterPro	IPR039008	Intermediate filament, rod domain	13	73	2.08	6.23	3.7e-20	KRT1, KRT10, KRT14, KRT16, KRT17, KRT2, KRT5, KRT6A, KRT6B, KRT77, KRT78, KRT80, KRT9
InterPro	IPR018039	Intermediate filament protein, conserved site	12	62	2.12	6.04	5.4e-19	KRT1, KRT10, KRT14, KRT16, KRT17, KRT2, KRT5, KRT6A, KRT6B, KRT77, KRT78, KRT9
InterPro	IPR003054	Keratin, type II	8	26	2.32	4.68	3.2e-13	KRT1, KRT2, KRT5, KRT6A, KRT6B, KRT77, KRT78, KRT80
InterPro	IPR032444	Keratin type II head	7	25	2.28	3.93	4.0e-11	KRT1, KRT2, KRT5, KRT6A, KRT6B, KRT77, KRT78

Table S8: Cerebellar vermis pathways enriched in upregulated proteins (FDR-adjusted $p < 0.05$) of adults with idiopathic autism *(continued)*

Category	Term ID	Term description	Gene count	Back-ground	Strength	Signal	FDR	Matching proteins in network
InterPro	IPR002957	Keratin, type I	5	32	2.03	2.10	2.9e-06	KRT10, KRT14, KRT16, KRT17, KRT9
SMART	SM01391	Intermediate filament protein	12	71	2.06	5.86	5.4e-19	KRT1, KRT10, KRT14, KRT16, KRT17, KRT2, KRT5, KRT6A, KRT6B, KRT78, KRT80, KRT9

Table S9: Non-significant synaptic proteins of cerebellar vermis in children with idiopathic autism (FDR-adjusted p > 0.05)

Gene name	FDR-adjusted p-value	log2(fold-change)	Number of peptides
A0A2R8YDQ0	0.9482	-0.1110	3
A0A3B3ISG8	0.7152	0.1908	5
A0A3B3ISV5	0.8345	0.1208	3
A0A3B3ITX4	0.8012	0.1217	5
A1BG	0.8908	0.1679	2
A2M	0.5191	0.3014	3
A2ML1	0.9736	0.0601	2
AAK1	0.4874	-0.4909	3
AAMP	0.4589	-0.3249	1
AARS	0.9819	0.0132	8
AARS2	0.5858	-0.2796	1
AARSD1	0.5145	-0.4977	1
AASS	0.9839	0.0318	1
ABAT	0.5264	0.2762	5
ABCA2	0.9555	0.0924	1
ABCA7	0.8400	-0.2425	1
ABCA8	0.5653	-0.4734	1
ABCB6	0.8820	-0.1012	1
ABCB7	0.9555	-0.1480	1
ABCB8	0.9217	-0.2564	2
ABCD2	0.7022	-0.4325	2
ABCD3	0.9838	0.0336	1
ABCE1	0.8480	-0.1912	3
ABCF1	0.9211	-0.2861	1
ABCF2	0.9405	-0.0646	1
ABCF3	0.9891	-0.0162	3
ABHD10	0.3765	-0.4047	2
ABHD11	0.6258	0.3620	1
ABHD14B	0.8378	-0.4270	1
ABHD6	0.9283	-0.1912	1
ABI1	0.1921	-0.3165	1
ABI2	0.8400	-0.1480	3
ABLIM1	0.8512	0.1408	3
ABR	0.9484	-0.0817	3
ACAA1	0.7640	-0.2213	3
ACAA2	0.1921	0.5322	7
ACACA	0.7319	-0.5477	1
ACAD8	0.9819	-0.0652	1
ACADM	0.7938	-0.2652	1
ACADS	0.6795	0.4468	2
ACAP2	0.9404	-0.1107	1
ACAT2	0.6375	0.3183	2
ACBD5	0.9217	-0.1216	2
ACLY	0.3202	-0.2053	11

Table S9: Non-significant synaptic proteins of cerebellar vermis in children with idiopathic autism (FDR-adjusted p > 0.05) *(continued)*

Gene name	FDR-adjusted p-value	log2(fold-change)	Number of peptides
ACO1	0.9596	-0.0896	1
ACO2	0.5826	0.2411	5
ACOT11	0.9805	0.0233	2
ACOT13	0.5134	-0.2786	4
ACOT7	0.3765	0.3581	4
ACOT9	0.6565	-0.3237	3
ACOX1	0.9847	-0.0297	1
ACSBG1	0.7251	-0.2424	3
ACSF2	0.4874	0.6517	1
ACSL1	0.9217	-0.0922	1
ACSL3	0.4504	-0.3637	2
ACSL4	0.9708	0.0768	1
ACSL6	0.9053	-0.1277	1
ACSM5	0.9947	0.0067	1
ACSS1	0.5400	0.6675	1
ACSS2	0.7586	0.2400	1
ACSS3	0.5996	0.4129	1
ACTB	0.9450	-0.0199	16
ACTBL2	0.9819	-0.0231	1
ACTG1	0.9753	0.0402	1
ACTN4	0.6902	0.2209	2
ACTR10	0.7000	-0.4428	1
ACTR1A	0.0633	-0.3392	7
ACTR1B	0.7354	-0.4028	1
ACTR2	0.7000	-0.2462	3
ACTR3	0.2894	-0.2458	3
ACTR3B	0.7491	-0.2144	1
ADAM10	0.5826	0.6431	1
ADAM11	0.4258	0.3318	1
ADAM22	0.3713	-0.2301	5
ADAP1	0.8841	0.1686	1
ADD1	0.8480	0.1075	6
ADD2	0.9839	0.0123	4
ADD3	0.6375	0.2416	6
ADGRG1	0.9971	0.0031	1
ADGRL3	0.8278	-0.4530	1
ADH5	0.1216	0.5913	5
ADPRHL2	0.6257	0.3134	1
AFDN	0.8833	0.1606	1
AFG1L	0.6767	0.1776	1
AFG3L2	0.9555	0.0428	8
AFTPH	0.9819	0.0311	1
AGAP1	0.4874	-0.5610	2
AGAP2	0.6418	-0.2388	4

Table S9: Non-significant synaptic proteins of cerebellar vermis in children with idiopathic autism (FDR-adjusted p > 0.05) *(continued)*

Gene name	FDR-adjusted p-value	log2(fold-change)	Number of peptides
AGAP3	0.5776	-0.3399	1
AGFG1	0.9819	0.0448	1
AGK	0.9366	-0.0691	1
AGL	0.7479	0.1954	3
AGPAT3	0.9805	0.0246	4
AGPAT4	0.8989	-0.1479	1
AGPS	0.5826	-0.1824	1
AHCY	0.2701	0.3019	5
AHCYL1	0.8333	-0.0817	7
AHCYL2	0.8345	-0.3207	1
AHNAK2	0.8110	-0.1565	4
AHSA1	0.9695	-0.0477	2
AIDA	0.9555	-0.2162	1
AIF1L	0.6540	-0.3923	1
AIFM3	0.9376	-0.0819	2
AIMP2	0.5188	-0.2742	4
AK1	0.8278	0.2042	6
AK3	0.2960	0.3957	3
AK4	0.9819	0.0283	5
AK5	0.7354	0.2951	1
AKAP12	0.4473	0.2768	7
AKAP9	0.9145	-0.2073	1
AKR1A1	0.5653	0.4214	4
AKR1B1	0.5253	0.4302	3
AKR1C1	0.8335	0.2470	3
AKR7A2	0.7822	0.3621	1
AKT1	0.8121	-0.3539	1
ALAD	0.7834	0.1422	3
ALB	0.1161	0.3195	13
ALDH18A1	0.8694	-0.1618	3
ALDH1A1	0.8833	-0.0645	12
ALDH1B1	0.9819	-0.0293	1
ALDH3A2	0.6795	0.3364	1
ALDH4A1	0.7832	0.1821	5
ALDH5A1	0.8380	0.1306	4
ALDH6A1	0.3202	0.2081	15
ALDH8A1	0.8894	-0.1282	1
ALDH9A1	0.9217	0.0636	5
ALDOA	0.9482	-0.0327	10
ALDOC	0.4152	-0.1411	16
ALS2	0.5543	-0.3277	2
AMER2	0.6423	-0.3020	2
AMOT	0.9947	-0.0114	1
AMPH	0.9646	0.0441	4

Table S9: Non-significant synaptic proteins of cerebellar vermis in children with idiopathic autism (FDR-adjusted p > 0.05) *(continued)*

Gene name	FDR-adjusted p-value	log2(fold-change)	Number of peptides
AMT	0.6364	-0.1675	2
AMY1B	0.8558	-0.2817	1
ANK2	0.8989	-0.0405	24
ANK3	0.6215	-0.1480	16
ANKFY1	0.7989	-0.5183	1
ANKMY2	0.4231	-0.4873	2
ANKRD29	0.9320	-0.1064	3
ANKRD44	0.5134	-0.4005	3
ANKS1B	0.9736	0.0492	2
ANLN	0.9482	0.0480	3
ANP32A	0.3713	-0.3086	3
ANXA1	0.4178	0.4988	2
ANXA11	0.9555	0.0797	1
ANXA2	0.6858	0.2415	6
ANXA6	0.4479	0.2788	11
ANXA7	0.7822	-0.0938	2
AP1B1	0.5253	-0.1748	9
AP1G1	0.4358	-0.3600	3
AP1M1	0.9555	-0.0777	3
AP1S2	0.6339	-0.5080	1
AP2A2	0.6177	-0.1954	6
AP2M1	0.8780	-0.1771	3
AP2S1	0.7928	-0.1742	1
AP3B1	0.8989	-0.1234	3
AP3B2	0.2831	-0.2614	8
AP3D1	0.4231	-0.2101	4
AP3M2	0.5253	-0.2933	5
AP4S1	0.9217	-0.1137	2
APBA2	0.9554	-0.0955	1
APBB1	0.9482	0.1118	1
APC	0.4243	-0.6258	1
APEH	0.7112	0.2722	2
APEX1	0.9649	0.0591	1
APMAP	0.7586	0.2371	2
APOA1	0.9171	0.0975	3
APOB	0.4231	0.2635	6
APOD	0.3348	0.9840	1
APOE	0.9053	-0.1698	1
APOOL	0.9311	-0.1778	1
APPL1	0.6177	0.4901	2
APPL2	0.7354	0.2387	2
APRT	0.6375	0.3514	1
ARCN1	0.4364	-0.4174	4
ARF3	0.5253	0.2313	6

Table S9: Non-significant synaptic proteins of cerebellar vermis in children with idiopathic autism (FDR-adjusted p > 0.05) *(continued)*

Gene name	FDR-adjusted p-value	log2(fold-change)	Number of peptides
ARF4	0.9709	-0.0415	3
ARF6	0.9819	-0.0191	5
ARFGAP1	0.9847	-0.0509	1
ARFGEF1	0.9484	-0.0792	1
ARFGEF2	0.5311	-0.3499	1
ARFGEF3	0.8693	-0.1637	5
ARG1	0.9468	0.0740	5
ARHGAP1	0.9819	-0.0875	1
ARHGAP12	0.6490	-0.3201	1
ARHGAP17	0.8480	0.1485	1
ARHGAP21	0.9053	-0.1018	4
ARHGAP35	0.7354	-0.1889	3
ARHGAP44	0.7672	-0.2602	3
ARHGAP5	0.8333	-0.1777	3
ARHGEF1	0.9211	0.2489	1
ARHGEF10L	0.9610	-0.0860	2
ARHGEF11	0.6423	-0.3830	1
ARHGEF12	0.8578	-0.3664	1
ARHGEF17	0.8670	-0.3189	1
ARHGEF2	0.9376	0.1782	1
ARHGEF3	0.9555	-0.1942	1
ARHGEF33	0.3135	-0.4530	5
ARHGEF6	0.7817	-0.2081	1
ARHGEF7	0.9353	-0.0814	4
ARID3B	0.7992	-0.4903	1
ARL1	0.9171	-0.1339	1
ARL2	0.9047	-0.1163	2
ARL3	0.9507	-0.0890	3
ARL6	0.8281	-0.1870	1
ARL8A	0.9819	-0.0705	1
ARMC1	0.9376	0.1031	1
ARMC10	0.6902	-0.3959	2
ARMC6	0.9555	-0.1147	1
ARMC8	0.9171	-0.0591	3
ARMT1	0.9676	-0.1213	1
ARPC1A	0.6452	-0.1383	4
ARPC2	0.9217	-0.0650	4
ARPC4	0.8833	-0.0795	6
ARPC5	0.9561	-0.0565	1
ARPC5L	0.1640	-0.3356	4
ARRB1	0.9217	0.0773	2
ARVCF	0.9366	-0.1341	2
ASAH1	0.8464	0.1821	2
ASAP1	0.3197	-0.4320	2

Table S9: Non-significant synaptic proteins of cerebellar vermis in children with idiopathic autism (FDR-adjusted p > 0.05) *(continued)*

Gene name	FDR-adjusted p-value	log2(fold-change)	Number of peptides
ASAP2	0.7000	-0.3066	1
ASCC3	0.5543	-0.2199	1
ASNA1	0.7980	-0.1832	3
ASNS	0.9217	-0.1698	2
ASPH	0.9819	-0.0169	5
ASRGL1	0.7251	-0.3579	1
ASS1	0.8871	-0.1307	1
ATAD1	0.8940	-0.3366	1
ATAD3A	0.9652	-0.0677	2
ATAT1	0.9610	-0.1260	1
ATG16L1	0.9555	0.0599	1
ATG2B	0.6260	-0.4043	1
ATG3	0.9443	0.1468	2
ATG4C	0.9885	0.0382	1
ATG9A	0.8093	-0.3241	1
ATIC	0.8833	0.2178	4
ATL1	0.9211	-0.1980	4
ATP1A1	0.7454	-0.1549	6
ATP1A2	0.7931	-0.1297	4
ATP1A3	0.4364	-0.1282	18
ATP1B1	0.9555	0.0467	5
ATP2A2	0.9555	-0.0319	12
ATP2A3	0.9047	-0.1104	3
ATP2B1	0.9211	0.0807	6
ATP2B2	0.6215	0.4769	2
ATP2B3	0.9610	0.0806	1
ATP5F1A	0.5559	0.1278	21
ATP5F1B	0.2951	0.2117	14
ATP5F1C	0.8207	0.1128	9
ATP5F1D	0.9966	-0.0018	2
ATP5F1E	0.9839	0.0175	1
ATP5IF1	0.9053	0.2764	2
ATP5MC1	0.7930	0.2082	1
ATP5ME	0.8513	0.1482	3
ATP5MF	0.8843	-0.1733	1
ATP5MPL	0.9217	-0.1536	1
ATP5PB	0.6423	0.3450	2
ATP5PD	0.2497	0.4210	3
ATP5PF	0.8989	0.1677	1
ATP5PO	0.7772	0.1305	8
ATP6AP1	0.5844	-0.2806	3
ATP6AP2	0.6850	-0.5170	1
ATP6V0A1	0.3900	-0.3539	3
ATP6V0D1	0.9217	-0.0932	5

Table S9: Non-significant synaptic proteins of cerebellar vermis in children with idiopathic autism (FDR-adjusted p > 0.05) *(continued)*

Gene name	FDR-adjusted p-value	log2(fold-change)	Number of peptides
ATP6V1A	0.6490	-0.1556	5
ATP6V1B2	0.4243	-0.2014	9
ATP6V1C1	0.3197	-0.2512	9
ATP6V1D	0.8207	-0.2462	1
ATP6V1E1	0.9736	-0.0235	6
ATP6V1H	0.9053	-0.0926	3
ATP8A1	0.7156	0.2945	2
ATP9A	0.7938	-0.5770	1
ATPAF1	0.9217	0.1281	2
ATXN10	0.9217	-0.0707	4
ATXN2L	0.5826	-0.2513	1
AUH	0.3793	-0.1655	10
AZGP1	0.9353	0.2251	1
B4DLN1	0.4241	0.6647	1
BABAM1	0.9468	0.0522	2
BAG6	0.9819	-0.0370	1
BAIAP2	0.7000	-0.1825	5
BASP1	0.6565	-0.1880	6
BBOX1	0.7474	0.5673	1
BBS2	0.9555	-0.0314	1
BBS9	0.9482	-0.1010	1
BCAN	0.9784	-0.0409	2
BCAP29	0.6384	-0.5131	1
BCAP31	0.6540	-0.3342	1
BCAR1	0.9483	-0.1575	1
BCAS1	0.9482	-0.0548	10
BCAS3	0.8993	-0.2644	1
BCCIP	0.7518	0.2496	1
BCR	0.4231	-0.7187	1
BDH2	0.7461	0.2163	2
BECN1	0.8989	-0.1009	1
BIN1	0.5495	0.2089	6
BLMH	0.6953	0.2231	4
BLVRA	0.8947	-0.0974	3
BLVRB	0.6903	0.3581	1
BNIP1	0.4487	-0.2582	2
BOLA2	0.9927	-0.0100	2
BPIFA2	0.7822	-0.3773	1
BPIFB1	0.9217	-0.0941	2
BRAF	0.9217	-0.2524	1
BRK1	0.5635	-0.3578	2
BRPF3	0.8634	-0.5114	1
BRSK1	0.4931	-0.2958	1
BSN	0.6907	0.1506	10

Table S9: Non-significant synaptic proteins of cerebellar vermis in children with idiopathic autism (FDR-adjusted p > 0.05) *(continued)*

Gene name	FDR-adjusted p-value	log2(fold-change)	Number of peptides
BTBD17	0.7834	-0.2547	1
BTN3A2	0.5145	-0.3515	1
BUB3	0.7541	-0.1554	2
BZW1	0.9569	0.0395	5
C11orf54	0.9838	0.0264	1
C15orf38	0.9366	0.1413	1
C2CD2L	0.9784	-0.0901	1
C2CD5	0.9283	-0.1739	1
C2orf81	0.5826	0.6659	1
C4A	0.4269	0.2875	6
CA1	0.4868	0.5320	1
CA10	0.8738	-0.2750	1
CA2	0.1664	0.7893	3
CA4	0.4487	-0.1951	6
CA8	0.8340	-0.4685	1
CAB39	0.4883	-0.3460	2
CAB39L	0.7333	-0.2685	1
CACNA1A	0.6575	0.4221	1
CACNA2D1	0.7928	0.1783	2
CACNB1	0.7772	-0.2869	1
CACNB2	0.6795	-0.1437	4
CACNB3	0.9695	0.0429	1
CACNB4	0.8288	-0.1181	1
CACNG2	0.8926	-0.1928	1
CACYBP	0.9217	-0.1017	1
CAD	0.9736	0.0311	1
CADPS	0.3713	-0.5019	6
CADPS2	0.1666	-0.2622	18
CALB1	0.1773	-0.5308	5
CALB2	0.9217	-0.0612	6
CALML5	0.9482	-0.1479	2
CALR	0.7548	0.2341	5
CAMK1	0.8989	-0.2866	1
CAMK1D	0.8493	-0.2253	3
CAMK2A	0.8833	-0.1088	8
CAMK2B	0.9353	-0.0665	4
CAMK2D	0.7473	-0.2032	8
CAMK2G	0.8949	-0.1144	6
CAMK4	0.9784	-0.0444	4
CAMKK2	0.9999	-0.0001	2
CAMSAP1	0.9217	0.1329	1
CAMSAP2	0.6177	-0.2242	2
CAND1	0.7621	-0.0709	15
CAND2	0.9555	-0.0547	2

Table S9: Non-significant synaptic proteins of cerebellar vermis in children with idiopathic autism (FDR-adjusted p > 0.05) *(continued)*

Gene name	FDR-adjusted p-value	log2(fold-change)	Number of peptides
CANX	0.9819	0.0355	2
CAP1	0.9597	-0.0274	6
CAP2	0.9053	-0.1445	2
CAPG	0.1559	0.5232	3
CAPN1	0.9819	0.0130	5
CAPN2	0.6423	0.7291	1
CAPN5	0.9480	0.0864	1
CAPNS1	0.9217	0.1132	2
CAPRIN1	0.3713	-0.2554	5
CAPZA1	0.6922	-0.2161	3
CAPZB	0.9396	-0.0437	6
CARD16	0.9916	-0.0404	1
CARM1	0.9819	0.0515	1
CARMIL1	0.6381	-0.4945	1
CARNS1	0.9819	-0.0573	1
CARS	0.8345	0.1144	3
CASK	0.5996	-0.1620	7
CASKIN1	0.9784	0.0226	7
CASP14	0.8715	0.2862	2
CASQ2	0.5498	0.6227	1
CAST	0.9484	0.0694	3
CASTOR2	0.5639	0.4733	1
CAT	0.9646	-0.0367	6
CAVIN2	0.9482	-0.1218	1
CBLN1	0.6423	-0.2686	1
CBLN3	0.5543	-0.5678	1
CBR1	0.6375	0.5878	3
CBR3	0.8043	0.3442	2
CBR4	0.9876	0.0199	1
CCAR1	0.7354	-0.2476	1
CCAR2	0.5451	-0.4418	3
CCDC127	0.7910	-0.2094	1
CCDC183	0.5923	-0.2853	1
CCDC22	0.7853	0.2537	1
CCDC6	0.6565	-0.3699	1
CCDC65	0.8110	0.7373	1
CCDC91	0.6585	-0.1786	2
CCDC93	0.7403	-0.3029	1
CCZ1B	0.9838	-0.0240	2
CD2AP	0.7251	-0.1884	2
CD47	0.2343	-0.3218	4
CD59	0.9211	0.0935	1
CD9	0.9610	-0.1805	1
CDC34	0.9217	-0.2303	1

Table S9: Non-significant synaptic proteins of cerebellar vermis in children with idiopathic autism (FDR-adjusted p > 0.05) *(continued)*

Gene name	FDR-adjusted p-value	log2(fold-change)	Number of peptides
CDC37	0.5579	-0.2948	3
CDC42	0.9784	0.0297	1
CDC42BPA	0.9217	-0.0643	5
CDC42BPB	0.8144	-0.2582	2
CDC42EP4	0.4487	-0.4402	2
CDH15	0.6490	0.3992	1
CDH2	0.4868	0.4872	1
CDK16	0.7354	0.2585	2
CDK18	0.9784	-0.0557	1
CDK5	0.5653	-0.3810	3
CDK5RAP3	0.9482	-0.1751	2
CDSN	0.9482	0.0715	4
CELF1	0.3814	-0.5732	3
CELF2	0.9217	-0.1757	1
CELF5	0.3197	-0.4508	1
CEMIP2	0.9916	-0.0243	1
CEND1	0.9217	-0.1836	2
CENPE	0.9211	-0.0759	1
CEP170	0.7621	-0.1593	4
CEP76	0.4416	-0.2996	5
CEPT1	0.9146	-0.0800	1
CETN2	0.8051	0.1828	1
CFL1	0.8345	0.1623	4
CFL2	0.9783	0.0344	3
CGGBP1	0.9819	0.0343	1
CHCHD3	0.9217	0.0509	7
CHDH	0.9555	0.0462	6
CHID1	0.8833	-0.1624	1
CHL1	0.9652	-0.0424	1
CHM	0.9999	-0.0007	1
CHMP1A	0.6177	-0.2278	5
CHMP1B	0.9555	-0.0484	3
CHMP2A	0.8300	-0.2109	2
CHMP2B	0.3713	-0.3576	4
CHMP4B	0.4077	-0.4028	3
CHMP5	0.9708	0.0467	1
CHN2	0.7928	-0.2615	2
CHORDC1	0.9482	0.0851	1
CHP1	0.5336	0.3724	1
CIAPIN1	0.6795	-0.1969	1
CISD2	0.3197	-0.4420	2
CIT	0.6585	0.4332	1
CKAP4	0.5336	-0.1955	6
CKAP5	0.3382	-0.1488	13

Table S9: Non-significant synaptic proteins of cerebellar vermis in children with idiopathic autism (FDR-adjusted p > 0.05) *(continued)*

Gene name	FDR-adjusted p-value	log2(fold-change)	Number of peptides
CKB	0.9819	-0.0255	11
CKMT1A	0.1921	-0.3087	7
CLASP1	0.9014	-0.4052	1
CLASP2	0.4548	-0.2712	3
CLCN4	0.9217	-0.2084	1
CLCN7	0.8898	0.1427	1
CLDN11	0.3197	0.3764	4
CLDND1	0.9784	-0.0455	1
CLEC16A	0.6490	0.5862	1
CLIC4	0.9422	0.2590	1
CLIP1	0.1382	-0.2384	8
CLIP2	0.6953	-0.2346	6
CLMN	0.3860	-0.5781	1
CLSTN1	0.9482	0.1054	1
CLTA	0.8270	-0.1471	4
CLTB	0.5900	-0.2659	5
CLTC	0.3956	-0.1981	20
CLU	0.9555	0.0285	5
CLVS2	0.9999	-0.0004	2
CMAS	0.6423	-0.3332	1
CMBL	0.9709	0.2547	1
CMC1	0.6601	-0.3908	1
CMPK1	0.9422	-0.1670	2
CNDP2	0.2644	0.4715	7
CNN1	0.9555	0.1348	1
CNN3	0.9652	-0.0365	2
CNNM2	0.9482	-0.0459	1
CNNM4	0.9604	0.0600	1
CNOT1	0.8738	-0.0879	5
CNOT2	0.8513	-0.1101	1
CNOT3	0.7938	-0.4508	1
CNOT9	0.3957	-0.8659	1
CNP	0.8288	0.0927	21
CNRIP1	0.6423	0.2288	4
CNTFR	0.3713	-0.4161	1
CNTN1	0.0559	-0.2362	9
CNTN2	0.4154	-0.2597	2
CNTNAP1	0.5543	-0.4372	2
CNTNAP2	0.6375	-0.2456	1
COA7	0.7938	-0.1916	1
COG2	0.9784	-0.0295	2
COG3	0.9555	0.0339	2
COG5	0.8002	-0.2177	1
COG6	0.9819	0.0265	1

Table S9: Non-significant synaptic proteins of cerebellar vermis in children with idiopathic autism (FDR-adjusted p > 0.05) *(continued)*

Gene name	FDR-adjusted p-value	log2(fold-change)	Number of peptides
COL1A2	0.8288	0.2226	1
COL6A3	0.9529	0.0684	4
COL9A1	0.9911	0.0467	1
COLGALT2	0.8400	-0.1432	1
COMMD5	0.8042	0.1953	1
COMMD7	0.9652	-0.0730	1
COMT	0.9927	-0.0151	2
COPA	0.7938	-0.1538	5
COPB1	0.6423	-0.2273	3
COPB2	0.0755	-0.5218	4
COPG1	0.7844	-0.2361	3
COPG2	0.8578	-0.1563	2
COPS2	0.9819	0.0285	2
COPS3	0.7603	-0.3102	1
COPS5	0.9593	-0.0475	2
COPS6	0.6452	0.3530	1
COPS7A	0.9802	0.0340	2
COQ8A	0.9217	0.2385	1
COQ9	0.2951	0.6298	2
CORO1A	0.3197	-0.2504	6
CORO1B	0.9366	-0.1335	3
CORO1C	0.9217	-0.0550	4
CORO2A	0.4339	-0.2732	3
CORO2B	0.0674	-0.3898	3
CORO7	0.7951	-0.1527	2
COTL1	0.9482	0.0564	4
COX4I1	0.9217	-0.1055	2
COX5A	0.9610	0.0504	1
COX5B	0.5588	0.2925	3
COX6B1	0.7822	-0.2033	2
COX6C	0.6907	0.3379	1
COX7A2	0.8743	0.3438	1
COX7A2L	0.9353	0.1187	1
COX7C	0.9480	0.1130	2
CPA4	0.9927	0.0150	2
CPE	0.4248	-0.3977	2
CPLX1	0.7938	0.3209	1
CPLX2	0.9298	0.1156	2
CPLX4	0.3197	0.5296	1
CPM	0.7548	-0.2699	1
CPNE3	0.9217	0.0898	2
CPNE6	0.8144	0.3263	2
CPO	0.9217	-0.1391	1
CPSF6	0.8144	-0.2161	1

Table S9: Non-significant synaptic proteins of cerebellar vermis in children with idiopathic autism (FDR-adjusted p > 0.05) *(continued)*

Gene name	FDR-adjusted p-value	log2(fold-change)	Number of peptides
CPSF7	0.2276	-0.6135	3
CPT1A	0.9916	-0.0080	4
CPT2	0.9569	-0.1018	1
CRAT	0.9628	0.0366	7
CRELD1	0.4479	-0.3256	3
CRIP2	0.9217	0.1045	1
CRKL	0.8606	-0.2133	2
CRMP1	0.5826	0.1697	7
CRTC1	0.7643	0.2147	1
CRYAB	0.9217	0.1080	4
CRYL1	0.5789	0.6514	2
CRYM	0.9966	-0.0025	3
CRYZ	0.6795	0.1878	5
CSDC2	0.9555	-0.0613	2
CSDE1	0.6071	-0.2887	5
CSE1L	0.9784	0.0412	3
CSK	0.9053	-0.1479	1
CSN1S1	0.2436	-0.4391	1
CSN1S2	0.9819	-0.0337	1
CSN2	0.9966	0.0065	1
CSNK1A1	0.9736	-0.0472	2
CSNK2B	0.9217	-0.1751	1
CSRP1	0.4258	-0.2309	2
CST3	0.4989	-0.5404	1
CST6	0.9819	-0.0632	1
CSTA	0.9927	0.0128	2
CSTB	0.6452	0.6819	1
CSTF3	0.9283	-0.1260	1
CT45A2	0.9217	-0.4873	1
CTBP1	0.8144	0.1707	2
CTH	0.9784	-0.0653	1
CTIF	0.9376	-0.2007	1
CTNNA1	0.7822	0.4979	1
CTNNA2	0.8841	0.1038	7
CTNNA3	0.9211	0.0729	1
CTNNB1	0.9422	0.0528	6
CTPS1	0.8989	-0.1735	2
CTSB	0.8871	0.3210	1
CTSD	0.5495	0.1627	13
CTSH	0.9784	0.0824	1
CTSV	0.7643	0.4694	1
CTTN	0.5495	-0.2484	3
CTTNBP2	0.3202	-0.5267	1
CUL1	0.9819	0.0196	3

Table S9: Non-significant synaptic proteins of cerebellar vermis in children with idiopathic autism (FDR-adjusted p > 0.05) *(continued)*

Gene name	FDR-adjusted p-value	log2(fold-change)	Number of peptides
CUL2	0.9482	0.0933	1
CUL3	0.7251	0.1781	1
CUL4B	0.7678	0.1674	2
CUL5	0.9736	-0.0433	2
CUX1	0.4702	0.3758	1
CYB5R1	0.8300	-0.1175	4
CYB5R3	0.6595	0.6350	1
CYC1	0.9784	-0.0461	1
CYCS	0.7671	-0.2445	5
CYFIP1	0.4487	-0.2333	9
CYFIP2	0.6126	-0.3235	2
CYTH2	0.8833	-0.4898	1
DAAM1	0.6767	-0.1999	3
DAAM2	0.8578	-0.2010	2
DAB2IP	0.7000	-0.3763	2
DAGLA	0.5829	-0.2057	5
DAP3	0.5134	-0.5317	1
DAPK3	0.9784	0.0586	1
DARS	0.9468	-0.0563	6
DBN1	0.9819	-0.0325	2
DBT	0.9927	-0.0054	7
DCD	0.9936	-0.0057	7
DCLK1	0.9610	0.0350	6
DCLK2	0.8715	0.1165	5
DCLRE1A	0.9217	-0.2045	1
DCTN1	0.9079	-0.1245	2
DCTN2	0.1495	-0.1963	12
DCTN3	0.4269	-0.2500	3
DCTN4	0.9211	0.1093	3
DCTN5	0.9283	-0.1022	1
DCUN1D3	0.5543	0.2150	1
DCXR	0.7518	-0.1951	3
DDAH1	0.6585	0.1689	3
DDAH2	0.2052	0.5763	4
DDB1	0.9211	-0.1055	3
DDOST	0.7479	-0.1564	4
DDT	0.9784	0.0299	2
DDX18	0.9555	-0.0709	1
DDX19B	0.8480	-0.2157	3
DDX23	0.7328	-0.3161	1
DDX39A	0.3335	-0.5194	2
DDX39B	0.8110	-0.1707	2
DDX3X	0.4479	-0.3357	4
DDX5	0.4269	-0.7221	1

Table S9: Non-significant synaptic proteins of cerebellar vermis in children with idiopathic autism (FDR-adjusted p > 0.05) *(continued)*

Gene name	FDR-adjusted p-value	log2(fold-change)	Number of peptides
DDX6	0.3316	-0.3807	3
DECR1	0.6126	-0.1446	11
DECR2	0.5191	-0.4046	1
DEK	0.6177	-0.2632	1
DENND5B	0.9482	-0.0989	1
DENR	0.5451	-0.2004	1
DESI1	0.9555	-0.1024	1
DFFA	0.9147	0.3311	1
DGKH	0.5543	-0.3268	1
DGKZ	0.9482	-0.1507	1
DHRS11	0.9217	-0.2175	1
DHRS4	0.9709	-0.0352	3
DHX15	0.8898	-0.1351	4
DHX16	0.9589	-0.1291	1
DHX29	0.6315	-0.4311	1
DHX9	0.8400	-0.2225	2
DIAPH1	0.9454	0.1246	1
DIP2C	0.7354	-0.2707	3
DIRAS1	0.8303	-0.1272	4
DIRAS2	0.8989	-0.1491	3
DIS3L2	0.8841	-0.2042	1
DLAT	0.6601	0.1113	14
DLD	0.9784	0.0160	7
DLG1	0.9555	-0.1012	1
DLG2	0.7834	-0.2414	1
DLG3	0.4358	-0.3482	1
DLG4	0.9784	-0.0350	1
DLGAP1	0.8783	-0.1109	3
DLGAP3	0.9555	0.0709	1
DLGAP4	0.9593	-0.0949	1
DLST	0.8400	-0.0699	7
DMAC2L	0.6006	0.4016	2
DMD	0.9819	0.0269	5
DMKN	0.8871	0.2683	2
DMTN	0.6540	-0.2287	6
DMXL1	0.1377	-0.3615	6
DMXL2	0.0725	-0.3638	10
DNAJA1	0.9482	-0.0617	3
DNAJA3	0.9211	0.2488	1
DNAJA4	0.9311	-0.1890	1
DNAJB1	0.5559	0.5359	1
DNAJB4	0.1383	-0.4896	3
DNAJC11	0.9217	-0.0903	3
DNAJC12	0.8841	0.3220	1

Table S9: Non-significant synaptic proteins of cerebellar vermis in children with idiopathic autism (FDR-adjusted p > 0.05) *(continued)*

Gene name	FDR-adjusted p-value	log2(fold-change)	Number of peptides
DNAJC13	0.5831	-0.2881	2
DNAJC19	0.9482	-0.0682	1
DNAJC3	0.8606	-0.2454	1
DNAJC6	0.4269	-0.3356	4
DNAJC8	0.4154	-0.3753	4
DNM1	0.0786	-0.2608	30
DNM1L	0.9670	-0.0222	11
DNM2	0.7616	-0.2202	3
DNM3	0.2326	-0.3989	11
DNPEP	0.9482	-0.1030	1
DOCK10	0.9708	0.0584	1
DOCK3	0.6052	-0.3827	1
DOCK5	0.9555	-0.0690	1
DOCK7	0.9053	-0.1713	1
DOCK9	0.6490	-0.2401	2
DPP6	0.7671	-0.1535	4
DPP9	0.8480	0.1573	1
DPYSL2	0.1033	0.1939	17
DPYSL4	0.9646	-0.0399	5
DPYSL5	0.8890	-0.0605	6
DRAP1	0.6177	-0.3731	1
DRG1	0.6071	-0.3073	1
DRG2	0.9646	-0.0777	2
DROSHA	0.8838	0.3418	1
DSC2	0.7822	-0.1864	1
DSC3	0.9405	0.1306	1
DSG1	0.8989	-0.0811	9
DSP	0.9927	-0.0021	54
DST	0.6490	-0.1141	9
DSTN	0.9353	-0.0954	4
DTD2	0.6163	-0.4224	1
DTNA	0.9555	0.0493	4
DTX3	0.9011	-0.1513	1
DUSP3	0.9404	0.2601	1
DVL1P1	0.6902	-0.2552	1
DYM	0.7817	-0.3205	1
DYNC1I2	0.6113	-0.2869	3
DYNC2H1	0.9784	-0.0467	1
DYNLL2	0.5191	-0.3050	1
DYRK1A	0.7822	0.4540	1
DYSF	0.5900	-0.3760	1
E9PLD3	0.7822	-0.2541	1
ECH1	0.2850	-0.2676	5
ECHDC1	0.9482	0.1031	1

Table S9: Non-significant synaptic proteins of cerebellar vermis in children with idiopathic autism (FDR-adjusted p > 0.05) *(continued)*

Gene name	FDR-adjusted p-value	log2(fold-change)	Number of peptides
ECHDC2	0.9217	0.0903	1
ECHS1	0.7070	0.1395	5
ECI2	0.0892	-0.3466	5
EDC4	0.5996	-0.2069	6
EDF1	0.7076	-0.2889	2
EDIL3	0.6423	-0.2676	2
EEA1	0.6452	-0.0947	12
EEF1A1	0.8989	-0.2902	2
EEF1B2	0.0674	-0.5668	4
EEF1D	0.6850	-0.3184	1
EEF1G	0.3698	-0.3256	6
EEF2	0.1917	-0.2058	10
EEFSEC	0.7333	-0.3385	1
EEPD1	0.9347	-0.1592	1
EFHD1	0.9832	-0.0256	1
EFL1	0.9690	-0.0276	1
EFR3A	0.9366	-0.0841	2
EFR3B	0.4315	-0.3687	5
EGFR	0.9544	-0.0817	3
EGLN1	0.6540	0.2710	1
EHD1	0.9916	0.0083	8
EHD2	0.9982	-0.0015	2
EHD3	0.9047	0.1701	1
EHD4	0.9283	0.0982	2
EIF2A	0.9217	-0.1088	2
EIF2AK2	0.7603	-0.2094	3
EIF2S1	0.7671	0.2013	2
EIF2S2	0.4823	-0.2180	2
EIF2S3	0.9927	-0.0061	2
EIF3A	0.6071	0.3348	1
EIF3CL	0.4255	-0.4201	3
EIF3E	0.8480	-0.2112	2
EIF3F	0.9024	-0.0948	4
EIF3H	0.1816	-0.3606	4
EIF3I	0.7931	-0.1574	2
EIF3J	0.5892	-0.2957	2
EIF3K	0.6907	-0.2837	1
EIF4A1	0.8871	-0.4014	1
EIF4A3	0.6375	-0.2348	3
EIF4G1	0.8088	-0.1499	5
EIF4G2	0.2883	-0.2541	7
EIF4G3	0.8333	0.3197	1
EIF5A	0.3150	-0.2517	4
EIPR1	0.1495	-0.5083	2

Table S9: Non-significant synaptic proteins of cerebellar vermis in children with idiopathic autism (FDR-adjusted p > 0.05) *(continued)*

Gene name	FDR-adjusted p-value	log2(fold-change)	Number of peptides
ELAVL1	0.3713	-0.2532	4
ELAVL2	0.3956	-0.2602	8
ELAVL3	0.7601	-0.2931	2
ELOB	0.9819	-0.0211	5
ELOC	0.9177	0.1277	1
ELP1	0.6902	-0.2325	4
ELP2	0.5954	0.3565	1
ELP3	0.8122	-0.2152	1
EMC1	0.6903	-0.2948	2
EML2	0.9821	-0.0195	1
EML4	0.9784	-0.0495	1
ENO2	0.6795	0.2091	3
ENO3	0.9819	0.0367	2
ENOPH1	0.9784	0.1562	1
ENPP6	0.9053	-0.1262	1
ENSA	0.9966	0.0057	1
EPB41	0.9171	-0.1223	1
EPB41L1	0.7460	-0.1280	8
EPB41L2	0.9217	-0.0971	6
EPB41L3	0.2883	-0.3024	7
EPDR1	0.3684	-0.4826	2
EPHX1	0.9468	0.1062	1
EPM2A	0.8634	0.1656	1
EPN2	0.6286	0.2245	1
EPPK1	0.3698	-0.3289	4
EPRS	0.3083	-0.3340	3
EPS15	0.9217	0.1409	2
EPS15L1	0.3432	0.3086	4
EPS8L2	0.9217	-0.1443	1
ERBIN	0.9217	-0.0952	2
ERC1	0.9211	-0.0616	5
ERC2	0.3197	-0.4544	1
ERGIC1	0.9555	0.0563	1
ERH	0.0832	-1.4240	1
ERLIN2	0.9649	-0.0405	3
ERMN	0.6490	0.4365	2
ERMP1	0.9864	-0.0213	1
ERP29	0.4285	-0.2766	4
ERP44	0.9482	0.1247	1
ESD	0.9217	0.1374	1
ESYT2	0.6671	-0.4317	1
ETF1	0.6490	-0.2148	1
ETFA	0.9718	0.0608	2
ETFB	0.9376	0.0551	4

Table S9: Non-significant synaptic proteins of cerebellar vermis in children with idiopathic autism (FDR-adjusted p > 0.05) *(continued)*

Gene name	FDR-adjusted p-value	log2(fold-change)	Number of peptides
ETFDH	0.6423	0.4529	2
EXOC1	0.2951	-0.3406	2
EXOC2	0.8513	-0.2200	2
EXOC3	0.5543	-0.2401	3
EXOC4	0.8833	-0.1983	1
EXOC5	0.9217	-0.0626	6
EXOC6	0.7799	-0.1857	1
EXOC6B	0.5635	-0.1693	3
EXOC7	0.4548	-0.6207	2
EXOC8	0.8281	-0.2107	2
F13A1	0.7499	0.3706	1
FABP3	0.5253	0.3024	2
FABP5	0.9784	0.0835	1
FABP7	0.6601	0.2929	2
FADS6	0.8397	0.3240	1
FAHD1	0.7834	0.2763	1
FAHD2A	0.6418	0.4563	2
FAM126B	0.3965	-0.4413	3
FAM129B	0.8578	-0.1306	2
FAM131B	0.8309	0.3346	1
FAM136A	0.2411	-0.6029	2
FAM13A	0.4745	0.5789	1
FAM169A	0.7191	-0.4784	1
FAM171B	0.9802	-0.0989	1
FAM177A1	0.9366	0.0845	1
FAM45A	0.8124	-0.2878	1
FAM81A	0.9129	0.3475	1
FAM98B	0.6850	-0.6799	1
FAR1	0.9610	0.1158	1
FARP1	0.9966	-0.0028	4
FARSA	0.7487	-0.2290	4
FASN	0.9405	0.0306	28
FAT1	0.9819	0.0199	1
FAT2	0.1630	-0.2674	9
FAU	0.6423	-0.3319	2
FBL	0.9784	-0.0956	1
FBXO41	0.4594	-0.4323	1
FCHO2	0.7491	-0.2299	1
FDX1	0.7938	0.2892	1
FDXR	0.9610	0.0690	1
FECH	0.6540	-0.2033	3
FERMT2	0.9736	0.0402	1
FGA	0.1340	0.5769	3
FGF1	0.5543	0.3986	1

Table S9: Non-significant synaptic proteins of cerebellar vermis in children with idiopathic autism (FDR-adjusted p > 0.05) *(continued)*

Gene name	FDR-adjusted p-value	log2(fold-change)	Number of peptides
FGF12	0.9053	-0.1657	1
FGF13	0.6391	0.2440	1
FGG	0.8110	0.3336	1
FH	0.3168	0.3507	3
FHL1	0.9646	0.0543	6
FIBP	0.9839	0.0236	1
FKBP15	0.7698	-0.2674	2
FKBP3	0.5826	-0.8247	1
FKBP4	0.9885	0.0114	5
FKBP8	0.7728	-0.3417	1
FLG2	0.9217	0.2051	2
FLNA	0.9366	0.0357	9
FLNB	0.3083	0.2328	7
FLNC	0.9217	0.1881	1
FLOT1	0.9376	0.0535	3
FLOT2	0.7938	-0.1124	6
FLYWCH2	0.3956	-0.7832	1
FMN2	0.9555	-0.0904	1
FMNL2	0.7991	-0.1317	4
FMR1	0.9971	0.0033	2
FN3K	0.9555	-0.0500	3
FNBP1	0.4717	-0.1986	4
FNTB	0.8345	0.3100	1
FOCAD	0.4238	-0.3149	1
FRY	0.3702	-0.5491	2
FSCN1	0.4594	0.4645	4
FSD1	0.9934	-0.0081	1
FSD1L	0.8781	-0.1123	3
FTH1	0.9214	-0.1051	2
FTL	0.9602	0.0665	2
FTO	0.7584	-0.2180	2
FUNDC2	0.9482	-0.0854	2
FUS	0.5954	-0.2942	2
FXR1	0.9555	-0.0373	2
GABARAPL2	0.7522	-0.1447	4
GABBR1	0.8781	-0.1922	1
GABBR2	0.2644	-0.4204	3
GABRA1	0.3967	-0.5458	2
GABRA6	0.8400	-0.2026	4
GABRB2	0.7979	-0.5367	1
GABRG2	0.8367	-0.1591	1
GAD1	0.4548	-0.2913	4
GAK	0.5826	-0.2349	4
GALK1	0.9784	-0.0576	1

Table S9: Non-significant synaptic proteins of cerebellar vermis in children with idiopathic autism (FDR-adjusted p > 0.05) *(continued)*

Gene name	FDR-adjusted p-value	log2(fold-change)	Number of peptides
GANAB	0.8843	0.2539	2
GAP43	0.5313	0.3542	4
GAPDH	0.9468	-0.0153	29
GAPVD1	0.9864	-0.0311	1
GAREM1	0.8529	-0.2303	1
GARS	0.5653	0.2010	5
GART	0.9454	0.0559	3
GATB	0.9911	-0.0124	1
GATD1	0.6423	-0.2599	3
GATD3B	0.6113	0.4633	3
GBA	0.9838	-0.0209	3
GBE1	0.5253	0.5108	1
GBF1	0.7938	-0.3204	1
GCDH	0.9555	0.0665	1
GCLC	0.5996	0.2740	4
GCLM	0.9784	-0.0612	1
GCN1	0.5543	-0.2753	3
GCOM2	0.6795	-0.3687	1
GDI2	0.1909	0.3996	3
GDPD3	0.5543	0.3680	1
GEMIN5	0.8163	-0.1581	1
GET4	0.8303	-0.4672	1
GFM1	0.9708	-0.0489	2
GGA1	0.9217	-0.1135	1
GGCT	0.7354	0.2357	5
GGH	0.9482	-0.1248	1
GGT7	0.9927	0.0124	1
GID8	0.9484	-0.1223	1
GIGYF2	0.5814	-0.3981	1
GIPC1	0.8067	-0.1185	1
GIT1	0.9217	-0.1319	1
GIT2	0.9321	-0.1902	1
GJA1	0.6423	-0.3265	1
GK	0.7834	-0.1990	3
GKAP1	0.9832	0.0177	1
GLB1L3	0.9893	0.0191	1
GLG1	0.6126	-0.3389	1
GLIPR2	0.3202	-0.2781	1
GLOD4	0.3083	0.5050	2
GLRX	0.9819	0.0570	1
GLRX3	0.8162	-0.1491	1
GLS	0.8869	-0.1836	4
GLUD1	0.6565	0.1497	12
GLUL	0.1167	-0.5488	5

Table S9: Non-significant synaptic proteins of cerebellar vermis in children with idiopathic autism (FDR-adjusted p > 0.05) *(continued)*

Gene name	FDR-adjusted p-value	log2(fold-change)	Number of peptides
GM2A	0.7291	0.5991	1
GMFB	0.3240	-0.2692	4
GMPPA	0.7480	0.4861	1
GMPS	0.9819	-0.0172	3
GNA11	0.9555	-0.0974	2
GNAI1	0.9246	-0.0838	1
GNAI2	0.7603	0.3925	2
GNAO1	0.8715	0.1059	5
GNAQ	0.9566	-0.0541	6
GNAS	0.6565	0.1504	10
GNAZ	0.9238	-0.0580	6
GNB1	0.5777	0.2712	5
GNB5	0.7274	0.2163	3
GNE	0.9819	0.0487	1
GNG12	0.7548	0.4099	1
GNG7	0.9217	-0.2189	1
GNL1	0.8510	-0.3005	2
GNPDA1	0.9992	-0.0009	2
GOLGA2	0.9652	0.0724	1
GOLGA3	0.5097	-0.3518	2
GOLGA4	0.5495	-0.2779	2
GOPC	0.9283	-0.0930	1
GORASP2	0.8110	-0.2839	1
GOT1	0.8871	-0.0624	7
GOT2	0.5044	-0.2131	7
GPD1	0.7208	0.3587	1
GPD1L	0.7834	0.1596	1
GPHN	0.7938	0.1917	1
GPI	0.6595	0.1649	14
GPM6A	0.9885	-0.0150	1
GPNMB	0.6850	0.3325	1
GPR158	0.9217	-0.0619	3
GPR180	0.5954	-0.4665	1
GPRC5B	0.9819	-0.0307	1
GPRIN1	0.6795	0.1500	7
GPRIN3	0.9482	-0.1850	1
GPS1	0.9692	0.0290	4
GPSM1	0.9819	0.0524	1
GPX4	0.8871	-0.0938	3
GRB2	0.9819	0.0343	1
GRHPR	0.3288	0.8302	1
GRIA1	0.5485	-0.5470	1
GRIA2	0.5543	-0.1865	5
GRIA3	0.4616	-0.1918	4

Table S9: Non-significant synaptic proteins of cerebellar vermis in children with idiopathic autism (FDR-adjusted p > 0.05) *(continued)*

Gene name	FDR-adjusted p-value	log2(fold-change)	Number of peptides
GRIA4	0.8989	-0.0730	6
GRID2	0.7934	-0.3780	3
GRID2IP	0.8513	-0.4089	1
GRIN1	0.9217	-0.1208	2
GRIPAP1	0.9347	0.2092	1
GRK2	0.9561	0.0439	2
GRK5	0.9708	0.0859	1
GRM1	0.6835	-0.2134	4
GRM2	0.4077	-0.3108	3
GRM4	0.6795	0.2690	1
GRPEL1	0.8989	0.2218	2
GSDMA	0.9819	0.0956	1
GSK3B	0.4702	-0.3526	3
GSPT1	0.9649	-0.0336	5
GSPT2	0.9211	-0.1165	1
GSR	0.9555	-0.1302	1
GSS	0.7998	0.1385	4
GSTA4	0.1816	0.6731	1
GSTM2	0.4508	0.5617	3
GSTM3	0.8832	0.2661	3
GSTM5	0.6575	0.4383	2
GSTO1	0.5577	0.3461	1
GSTP1	0.8989	0.1904	1
GSTT2B	0.4616	0.4625	2
GTF2F2	0.9341	-0.1191	1
GTF2I	0.9217	-0.1931	1
GTPBP1	0.7938	-0.1897	3
GTPBP10	0.8095	-0.1779	1
GUCY1A1	0.9819	-0.0394	1
GUCY1B1	0.8093	-0.1856	1
GUF1	0.9966	-0.0069	1
GUK1	0.7454	-0.2375	1
GYG1	0.8056	0.2790	1
GYS1	0.9211	-0.1182	2
H1F0	0.0671	0.5515	5
H1FX	0.8512	0.1898	3
H2AFV	0.9927	0.0156	1
H2AFX	0.9966	0.0030	1
H2AFY2	0.8833	0.1692	2
H3BN98	0.3112	-0.6334	3
H3F3A	0.6177	0.6819	2
H7C2G1	0.9966	0.0028	1
HACD3	0.4364	-0.4155	2
HADH	0.8400	0.2381	2

Table S9: Non-significant synaptic proteins of cerebellar vermis in children with idiopathic autism (FDR-adjusted p > 0.05) *(continued)*

Gene name	FDR-adjusted p-value	log2(fold-change)	Number of peptides
HADHB	0.3449	0.2670	13
HAGH	0.4487	-0.2657	2
HAL	0.9652	0.0632	3
HAPLN1	0.7403	-0.3413	2
HAPLN2	0.4154	-0.7466	1
HARS	0.5653	-0.2120	5
HARS2	0.5653	-0.1698	4
HBA1	0.5826	0.3581	7
HBB	0.5653	0.4187	6
HBD	0.7417	0.2081	4
HBS1L	0.7499	-0.1628	1
HCFC1	0.2134	-0.5000	4
HDAC11	0.4154	-0.3663	2
HDAC6	0.5495	-0.5577	1
HDGFRP3	0.9217	0.2496	1
HDLBP	0.9211	-0.1358	2
HEBP2	0.8541	0.1358	2
HECTD4	0.6163	-0.5625	1
HECW2	0.9555	-0.1723	1
HEPACAM	0.4487	0.2536	2
HERC1	0.2642	-0.4223	2
HEXB	0.7671	0.1944	2
HIBCH	0.4487	0.3555	2
HID1	0.7354	-0.4519	1
HINT2	0.7822	0.5711	1
HIP1	0.8600	-0.1149	4
HIP1R	0.7491	-0.2221	2
HIST1H1B	0.1968	0.3869	8
HIST1H1C	0.3857	0.5195	1
HIST1H1D	0.2052	0.3725	10
HIST1H1E	0.2392	0.7060	3
HIST1H4A	0.5451	0.5449	6
HIST2H2AC	0.7938	0.5722	2
HIST2H2BE	0.2224	0.4528	6
HIST2H2BF	0.6595	0.7796	2
HK1	0.3197	-0.2146	23
HMGB1	0.9947	0.0093	1
HMGCS1	0.7211	-0.2945	1
HMGN2	0.9482	0.2316	1
HMGN4	0.9217	0.1833	1
HMOX2	0.9484	-0.0605	3
HNRNPA0	0.9555	-0.2492	1
HNRNPA1	0.4154	-0.5533	2
HNRNPA2B1	0.8144	-0.2892	2

Table S9: Non-significant synaptic proteins of cerebellar vermis in children with idiopathic autism (FDR-adjusted p > 0.05) *(continued)*

Gene name	FDR-adjusted p-value	log2(fold-change)	Number of peptides
HNRNPAB	0.5789	-0.3651	1
HNRNPC	0.4504	-0.1893	9
HNRNPDL	0.8715	-0.3439	1
HNRNPH1	0.6682	-0.3848	2
HNRNPH3	0.9854	-0.0326	2
HNRNPL	0.1161	-0.3743	2
HNRNPLL	0.7834	-0.2920	1
HNRNPR	0.1350	-0.4282	5
HNRNPU	0.4004	0.4527	5
HNRNPUL1	0.9736	0.0413	1
HOMER1	0.9785	-0.0365	1
HOMER3	0.4874	-0.2124	4
HP	0.1167	0.6634	4
HP1BP3	0.8270	0.1286	5
HPCA	0.9839	-0.0249	2
HPCAL4	0.9610	0.0526	2
HPRT1	0.7354	-0.1608	2
HPX	0.9927	-0.0198	1
HRG	0.9211	0.3350	1
HSD11B1	0.9819	0.0325	1
HSD17B10	0.1816	0.4894	3
HSD17B11	0.8833	-0.0725	4
HSD17B12	0.8345	-0.2225	1
HSD17B4	0.2906	-0.1363	10
HSD17B8	0.5809	-0.1979	5
HSDL2	0.8291	0.1220	6
HSP90AA1	0.8513	0.0798	26
HSP90AB1	0.9555	-0.0596	7
HSP90B1	0.2556	0.3022	10
HSPA12A	0.5253	-0.4650	6
HSPA13	0.9217	-0.1734	1
HSPA2	0.2556	0.2423	9
HSPA4	0.1371	0.2852	6
HSPA4L	0.7643	-0.1586	5
HSPA5	0.8207	-0.0711	9
HSPA6	0.9916	-0.0074	3
HSPA8	0.6850	0.0790	16
HSPA9	0.1444	0.2733	11
HSPB1	0.7772	0.1421	8
HSPB8	0.9376	0.1252	1
HSPE1	0.6795	0.1512	7
HSPH1	0.7251	0.2250	2
HTRA1	0.9053	-0.3349	1
HTT	0.3086	-0.3906	4

Table S9: Non-significant synaptic proteins of cerebellar vermis in children with idiopathic autism (FDR-adjusted p > 0.05) *(continued)*

Gene name	FDR-adjusted p-value	log2(fold-change)	Number of peptides
HUWE1	0.4339	-0.2041	9
HYOU1	0.4874	-0.2620	3
I3L4J1	0.9784	-0.0614	2
IARS	0.3965	-0.4588	4
IARS2	0.9819	0.0242	4
IBA57	0.9142	-0.1745	1
ICMT	0.7934	0.3413	1
IDE	0.8843	0.1051	3
IDH1	0.0892	0.3889	3
IDH2	0.8076	0.2001	6
IDH3A	0.6177	0.2038	8
IDH3B	0.9839	-0.0263	2
IDH3G	0.9916	0.0276	1
IFT27	0.9610	0.1019	1
IGBP1	0.5044	-0.6219	1
IGHA1	0.9652	0.0524	3
IGHG1	0.7537	0.2921	4
IGHG2	0.7822	-0.2235	2
IGHM	0.8288	0.1739	2
IGHV4	0.9366	0.1935	1
IGKC	0.8051	0.2033	3
IGKV2	0.5078	0.4474	1
IGKV3D	0.9211	0.1825	1
IGLC2	0.9934	-0.0335	1
IGLL5	0.9966	-0.0047	2
IGSF21	0.9819	-0.0341	2
IGSF8	0.1189	-0.2544	11
IGSF9B	0.7251	-0.1751	2
IKBKB	0.9792	0.0698	1
IL36G	0.9966	0.0048	1
IL37	0.9784	0.1195	1
ILF2	0.5577	-0.3961	2
ILF3	0.5543	-0.3923	2
ILK	0.8345	-0.3251	2
ILKAP	0.9708	0.0581	1
IMMT	0.9047	0.0554	10
IMPA2	0.9053	0.2018	2
IMPDH2	0.4874	-0.3897	2
INA	0.2901	-0.2572	16
INO80B	0.1785	0.4381	1
INPP1	0.8871	0.3095	1
INPP4A	0.9819	0.0306	2
INPP5F	0.7938	-0.1382	4
INPPL1	0.8989	-0.1869	1

Table S9: Non-significant synaptic proteins of cerebellar vermis in children with idiopathic autism (FDR-adjusted p > 0.05) *(continued)*

Gene name	FDR-adjusted p-value	log2(fold-change)	Number of peptides
IPO5	0.9646	0.0652	2
IPO7	0.9014	-0.1525	2
IPO9	0.6795	-0.4830	2
IQGAP1	0.4285	0.3632	3
IQGAP3	0.0698	-0.6070	1
IQSEC1	0.9482	-0.0914	1
IQSEC2	0.7653	-0.3973	2
IQSEC3	0.9927	0.0312	1
IRF2BP2	0.4487	0.5141	1
ISCA1	0.8095	-0.2323	2
IST1	0.1559	-0.3608	5
ISYNA1	0.8783	-0.2080	1
ITCH	0.9938	-0.0135	1
ITGB1	0.9555	0.1205	1
ITGB8	0.7570	0.1998	1
ITM2B	0.9217	-0.1754	1
ITPKB	0.7643	-0.7312	1
ITPR2	0.3713	-0.3377	2
ITSN1	0.8333	-0.0961	7
J3QQQ9	0.8291	-0.1456	3
JAK1	0.8833	-0.1677	1
KANK1	0.9217	-0.2214	1
KANK2	0.9736	-0.0716	1
KARS	0.6595	-0.5096	3
KATNAL1	0.3560	-0.5545	1
KAZN	0.7155	-0.3041	1
KBTBD11	0.5495	0.2242	4
KCNA1	0.6706	-0.8017	1
KCNAB1	0.9895	-0.0218	1
KCNAB2	0.7938	-0.1696	2
KCNC1	0.8833	-0.1837	1
KCND3	0.9595	0.0864	1
KCNIP4	0.4487	-0.4878	1
KCNK9	0.9652	-0.0778	1
KCTD12	0.9647	-0.0620	1
KHDRBS1	0.4339	-0.5888	2
KIAA0513	0.9482	0.1479	2
KIAA1107	0.9819	0.0224	3
KIAA1109	0.0674	-0.2895	4
KIAA1217	0.9569	-0.0568	1
KIF1A	0.9217	-0.0926	6
KIF1C	0.7938	-0.1655	2
KIF21A	0.7022	-0.2948	4
KIF21B	0.5892	-0.2792	1

Table S9: Non-significant synaptic proteins of cerebellar vermis in children with idiopathic autism (FDR-adjusted p > 0.05) *(continued)*

Gene name	FDR-adjusted p-value	log2(fold-change)	Number of peptides
KIF2A	0.9211	-0.0748	4
KIF3A	0.9934	-0.0057	2
KIF3B	0.9555	-0.1335	1
KIF5A	0.6540	-0.2007	5
KIF5B	0.7621	-0.1551	4
KIF5C	0.1371	-0.4269	4
KIT	0.8820	-0.1421	3
KLC1	0.4231	-0.2443	5
KLC2	0.6585	-0.2989	2
KLK8	0.8989	0.2430	1
KNDC1	0.7934	-0.2405	2
KPNA1	0.6113	-0.1901	2
KPNA2	0.9652	-0.0679	1
KPNA3	0.9366	-0.1092	1
KPNA5	0.9482	-0.1459	1
KPNB1	0.9708	-0.0250	8
KRAS	0.9217	-0.0922	3
KRT1	0.3197	-0.1450	52
KRT10	0.7772	0.0968	26
KRT13	0.9784	0.0661	3
KRT14	0.7354	-0.1041	24
KRT16	0.9561	-0.0417	6
KRT17	0.9422	0.0724	7
KRT2	0.7822	-0.0786	41
KRT23	0.9376	0.0958	2
KRT31	0.9610	0.0995	2
KRT4	0.9709	0.0708	3
KRT5	0.9217	0.0433	22
KRT6A	0.9819	0.0224	5
KRT6B	0.9610	0.0272	18
KRT71	0.9784	-0.0488	2
KRT77	0.8947	0.0855	16
KRT78	0.7156	-0.1649	8
KRT80	0.9283	-0.0787	6
KRT82	0.9916	-0.0120	3
KTN1	0.3197	-0.2400	8
L1CAM	0.9838	0.0123	6
L3HYPDH	0.9829	-0.0467	1
LACRT	0.8871	0.2345	2
LACTB	0.6795	0.2241	1
LAMB2	0.9366	-0.1919	1
LAMP1	0.7872	0.2414	2
LAMP2	0.9819	0.0314	1
LAMTOR1	0.6375	-0.5157	1

Table S9: Non-significant synaptic proteins of cerebellar vermis in children with idiopathic autism (FDR-adjusted p > 0.05) *(continued)*

Gene name	FDR-adjusted p-value	log2(fold-change)	Number of peptides
LANCL1	0.7350	-0.1898	2
LAP3	0.6423	0.3134	2
LARP4B	0.9211	-0.1655	2
LARS	0.6126	-0.2947	5
LARS2	0.7251	0.2545	1
LASP1	0.8843	-0.1896	1
LCN1	0.9422	0.2517	1
LDHA	0.7938	0.1198	5
LDHB	0.2435	0.2460	9
LETM1	0.6230	-0.2366	2
LETMD1	0.9053	-0.1741	1
LGALS1	0.8051	-0.1368	4
LGALS3BP	0.6423	-0.2323	2
LGALS7	0.7566	0.2339	2
LGALSL	0.9555	0.1546	1
LGI1	0.3202	-0.2103	4
LIMA1	0.9396	-0.2381	1
LIMCH1	0.9217	-0.0579	1
LIMK1	0.7913	-0.1901	1
LIMS1	0.8144	-0.1454	1
LIN7A	0.9736	0.0865	1
LLGL1	0.9217	-0.1373	2
LMAN1	0.4583	-0.4234	1
LMAN2	0.9468	0.2277	1
LMCD1	0.9589	-0.0985	1
LMF2	0.2436	-0.4900	1
LMNA	0.9217	-0.0880	3
LMNB1	0.7339	-0.2023	3
LMNB2	0.3689	-0.3633	5
LNPEP	0.6375	-0.3347	1
LONP1	0.9217	0.0760	9
LONRF2	0.4874	-0.2944	1
LPP	0.7518	0.4354	1
LRBA	0.9347	-0.1231	1
LRCH3	0.9885	-0.0227	1
LRP1	0.6423	-0.1921	4
LRPPRC	0.5653	-0.2045	12
LRRC31	0.5635	1.2006	1
LRRC40	0.9996	-0.0007	3
LRRC46	0.8942	0.1093	1
LRRC47	0.3197	-0.2719	6
LRRC4B	0.8345	0.0585	1
LRRC59	0.7603	-0.2554	1
LRRC8A	0.9555	0.0582	1

Table S9: Non-significant synaptic proteins of cerebellar vermis in children with idiopathic autism (FDR-adjusted p > 0.05) *(continued)*

Gene name	FDR-adjusted p-value	log2(fold-change)	Number of peptides
LRSAM1	0.9784	0.0781	1
LSAMP	0.6903	-0.1383	5
LSM4	0.6394	-0.3293	1
LSM6	0.9966	0.0049	1
LSM7	0.4616	-0.4376	1
LTA4H	0.5639	0.2905	3
LTF	0.7938	0.1809	3
LYPLA1	0.9405	-0.0596	2
LYPLA2	0.9838	-0.0155	3
LYPLAL1	0.9217	0.1980	2
LYSMD2	0.7938	0.2741	1
LYZ	0.9819	-0.0951	1
LZTFL1	0.9934	0.0100	1
M0QYV0	0.7541	-0.2066	3
MAB21L2	0.5996	-0.4239	1
MACF1	0.6375	-0.0935	18
MADD	0.7643	-0.2730	1
MAG	0.6741	-0.4065	2
MAGED2	0.5134	-0.2739	2
MAGI2	0.9819	0.0231	1
MAGOH	0.9555	-0.1171	1
MAIP1	0.9405	0.1456	2
MAL2	0.9966	0.0083	1
MAOA	0.9366	-0.1016	1
MAOB	0.9937	-0.0114	1
MAP11	0.7354	-0.4949	1
MAP1A	0.7354	-0.0540	30
MAP1B	0.7454	0.0622	33
MAP1LC3A	0.9171	-0.0602	4
MAP1S	0.8335	-0.1252	2
MAP2	0.2644	-0.2645	10
MAP2K1	0.9217	-0.1006	3
MAP2K2	0.9819	-0.0318	2
MAP2K4	0.9952	0.0054	2
MAP2K6	0.9217	-0.0758	2
MAP3K5	0.9690	-0.0317	6
MAP4	0.5777	0.1465	13
MAP4K2	0.9927	-0.0159	1
MAP4K3	0.6490	-0.6393	1
MAP6	0.4874	-0.2088	7
MAP7D1	0.9405	0.1711	1
MAPK1	0.9217	-0.0513	9
MAPK13	0.9708	-0.1055	1
MAPK3	0.9555	0.0864	2

Table S9: Non-significant synaptic proteins of cerebellar vermis in children with idiopathic autism (FDR-adjusted p > 0.05) *(continued)*

Gene name	FDR-adjusted p-value	log2(fold-change)	Number of peptides
MAPK8IP3	0.7251	-0.2886	1
MAPKAP1	0.7834	-0.2492	1
MAPRE2	0.7772	-0.2145	3
MAPRE3	0.9847	-0.0148	4
MAPT	0.8989	0.0545	17
MARC1	0.9911	0.0139	1
MARC2	0.9555	-0.0572	2
MARCKS	0.6490	-0.2238	4
MARCKSL1	0.8004	-0.3356	1
MARK1	0.7333	-0.1852	4
MARK2	0.4510	-0.5355	2
MARS	0.9211	-0.1497	3
MATR3	0.5321	-0.2990	3
MBOAT7	0.6585	-0.3890	1
MBP	0.9555	0.0418	15
MCAT	0.9555	-0.1514	1
MCCC1	0.9819	-0.0310	2
MCCC2	0.1057	-0.3074	5
MCEE	0.9482	-0.0939	1
MCF2L	0.9217	0.1236	1
MCTP1	0.2411	-0.4489	1
MCTS1	0.9482	0.1877	1
MDH1	0.7436	0.1582	5
MDH2	0.0892	-0.2389	14
ME2	0.8051	-0.2032	2
ME3	0.7970	-0.1508	2
MECP2	0.9819	-0.0173	5
METAP1	0.8833	-0.2450	1
MFN1	0.6953	-0.3239	2
MFN2	0.9610	0.0765	1
MGMT	0.9217	-0.0903	1
MGST3	0.8291	-0.2818	1
MICAL3	0.6903	-0.1657	7
MICU3	0.8270	-0.3789	1
MIGA1	0.9555	-0.0888	2
MKRN2	0.5635	-0.4378	1
MLF1	0.6767	-0.3633	1
MLYCD	0.9652	0.0528	1
MMAA	0.9217	-0.1116	3
MMS19	0.9784	-0.0674	1
MMUT	0.9893	0.0112	4
MOB2	0.7980	-0.4150	1
MOBP	0.8634	0.2444	1
MOCS1	0.6113	-0.2181	3

Table S9: Non-significant synaptic proteins of cerebellar vermis in children with idiopathic autism (FDR-adjusted p > 0.05) *(continued)*

Gene name	FDR-adjusted p-value	log2(fold-change)	Number of peptides
MOCS2	0.8088	0.2442	1
MOG	0.8871	0.3421	1
MOGS	0.5789	-0.3025	2
MON2	0.8833	-0.1835	1
MPHOSPH9	0.9927	0.0175	1
MPI	0.9927	0.0228	1
MPP1	0.9484	-0.1376	2
MPP2	0.7221	-0.2440	8
MPP5	0.9784	-0.0311	1
MPP6	0.7473	-0.1440	10
MPRIP	0.9792	-0.0389	2
MRAS	0.9736	0.0412	3
MRM3	0.8833	-0.1582	1
MROH1	0.5653	-0.3139	2
MRPL1	0.9908	0.0133	2
MRPL12	0.9211	0.1150	3
MRPL19	0.7621	-0.2672	2
MRPL43	0.8124	0.1922	1
MRPL44	0.6903	0.2916	1
MRPL53	0.7070	0.4937	1
MRPS16	0.6505	-0.3984	1
MRPS23	0.9574	-0.0723	2
MRPS27	0.4238	-0.3213	3
MRPS31	0.9217	-0.1628	2
MRPS5	0.9670	0.0760	1
MRPS6	0.9966	-0.0085	1
MRRF	0.8989	-0.0894	2
MSN	0.6071	0.3088	3
MSRA	0.5653	-0.3609	1
MT	0.9482	0.0722	3
MTCH1	0.9555	0.0608	2
MTCL1	0.9646	-0.0654	1
MTDH	0.9484	-0.1469	1
MTHFD1	0.6375	-0.1709	11
MTHFD1L	0.8833	-0.1922	3
MTMR10	0.9366	-0.1084	2
MTMR9	0.9366	-0.2117	1
MTR	0.9736	-0.0291	1
MTREX	0.6375	-0.4072	1
MTSS1	0.9711	-0.0543	1
MTSS2	0.9047	0.0864	3
MTURN	0.9482	0.2484	1
MTX2	0.9784	0.0285	2
MVD	0.9927	0.0164	1

Table S9: Non-significant synaptic proteins of cerebellar vermis in children with idiopathic autism (FDR-adjusted p > 0.05) *(continued)*

Gene name	FDR-adjusted p-value	log2(fold-change)	Number of peptides
MVP	0.9966	0.0017	3
MYBPC2	0.9047	0.1976	1
MYCBP	0.9053	-0.2300	1
MYCBP2	0.4339	-0.2545	3
MYH10	0.1230	-0.2670	16
MYH14	0.4154	-0.1773	7
MYH9	0.3965	0.1243	19
MYL6	0.4874	-0.2232	3
MYL6B	0.4315	-0.5730	1
MYO18A	0.6795	-0.1623	7
MYO1C	0.9482	0.1007	2
MYO1D	0.9217	-0.0901	4
MYO5A	0.0890	-0.2700	12
MYO6	0.9966	-0.0019	2
MYOF	0.5783	0.3863	2
NAA11	0.7354	-0.2737	1
NAA15	0.9047	0.1568	1
NAA35	0.9217	-0.1377	1
NACA2	0.5543	-0.3519	1
NAMPT	0.7758	0.1830	5
NANS	0.9366	0.2500	1
NAP1L1	0.8908	-0.3845	1
NAPA	0.9482	-0.1091	2
NAPB	0.8095	0.1744	4
NAPG	0.9908	-0.0089	5
NARS	0.7274	-0.1655	1
NASP	0.7853	-0.4270	1
NAT14	0.9784	0.0464	1
NAV1	0.9784	-0.0308	1
NAXD	0.8781	-0.1590	3
NAXE	0.8051	-0.1367	1
NBAS	0.9015	-0.1456	2
NBEA	0.7274	-0.2490	3
NCAM1	0.4487	-0.1503	8
NCAM2	0.9453	-0.0517	3
NCAN	0.5253	-0.5003	3
NCBP2	0.7928	-0.2260	1
NCDN	0.1391	0.3631	7
NCEH1	0.8022	-0.1987	1
NCK2	0.9217	-0.1567	3
NCKAP1L	0.6055	-0.6535	1
NCOA7	0.9483	-0.0958	2
NDRG2	0.6423	0.3449	2
NDRG3	0.9555	0.1027	1

Table S9: Non-significant synaptic proteins of cerebellar vermis in children with idiopathic autism (FDR-adjusted p > 0.05) *(continued)*

Gene name	FDR-adjusted p-value	log2(fold-change)	Number of peptides
NDRG4	0.6795	-0.2380	3
NDUFA10	0.8871	0.2128	2
NDUFA12	0.9819	-0.0536	1
NDUFA13	0.9709	-0.0443	1
NDUFA2	0.9819	0.0115	5
NDUFA3	0.7022	-0.2300	2
NDUFA4	0.2924	-0.2859	4
NDUFA5	0.9211	-0.1921	1
NDUFA6	0.9047	0.0848	3
NDUFA7	0.6257	-0.3920	1
NDUFAF7	0.8380	-0.2110	1
NDUFB10	0.9555	-0.0574	2
NDUFB11	0.9777	-0.0476	2
NDUFB3	0.5826	-0.2861	2
NDUFB4	0.9401	-0.0905	1
NDUFB6	0.9911	-0.0188	1
NDUFB9	0.9217	-0.1253	2
NDUFC2	0.9555	-0.0433	1
NDUFS1	0.5321	0.1750	6
NDUFS2	0.9482	0.2327	1
NDUFS3	0.9217	0.0972	3
NDUFS4	0.9353	0.0783	3
NDUFS6	0.9847	0.0192	1
NDUFS7	0.7865	0.1686	4
NDUFV1	0.8634	0.2327	2
NDUFV2	0.9366	-0.0610	2
NECAP1	0.8270	-0.2244	1
NEDD4L	0.9818	-0.0598	1
NEFH	0.9217	0.0941	3
NEFL	0.7541	0.1724	15
NEFM	0.5451	0.2228	12
NEGR1	0.5653	-0.1875	3
NEK7	0.7938	-0.1946	1
NEMF	0.9555	-0.0877	2
NENF	0.7904	0.6632	1
NEO1	0.9443	-0.1456	1
NF1	0.2224	-0.4526	3
NFASC	0.9579	0.0491	4
NFKB1	0.9819	0.0305	1
NFU1	0.8781	0.3043	1
NHP2	0.9555	-0.0689	1
NIPSNAP2	0.9652	-0.0653	1
NIT2	0.9709	0.0342	3
NKIRAS1	0.9217	-0.0832	2

Table S9: Non-significant synaptic proteins of cerebellar vermis in children with idiopathic autism (FDR-adjusted p > 0.05) *(continued)*

Gene name	FDR-adjusted p-value	log2(fold-change)	Number of peptides
NKIRAS2	0.3202	-0.3280	2
NLGN4Y	0.4269	-0.8848	1
NMD3	0.7938	-0.1783	1
NME1	0.9484	0.0347	5
NMT2	0.9197	-0.2180	1
NOMO2	0.7729	-0.1393	4
NONO	0.7350	-0.5394	1
NOS1	0.9211	-0.1220	3
NOVA2	0.8989	0.2051	2
NPEPL1	0.8738	-0.2543	1
NPM1	0.1216	-0.4026	8
NQO1	0.8869	0.2627	1
NR1D1	0.8447	0.3503	2
NRBP2	0.8871	-0.2327	1
NRCAM	0.8820	-0.0785	5
NRXN1	0.9947	0.0112	1
NRXN2	0.6565	-0.2538	3
NRXN3	0.2960	-0.3070	4
NSF	0.4154	-0.1835	25
NSFL1C	0.9966	-0.0074	1
NT5E	0.7417	0.1681	2
NTM	0.9610	-0.0494	2
NTPCR	0.9482	-0.1488	1
NUBP1	0.9802	-0.0351	2
NUBPL	0.6113	0.3067	2
NUCB2	0.9555	0.0671	1
NUCKS1	0.9652	0.0927	1
NUDC	0.7672	0.1343	4
NUDT16L1	0.9482	-0.0828	2
NUDT21	0.5134	-0.4335	3
NUDT5	0.7354	-0.2761	1
NUMA1	0.8088	-0.3245	1
NUMB	0.9847	0.0160	2
NUP205	0.8833	-0.1977	1
NUP210	0.4874	-0.2939	2
NUP43	0.9484	0.0972	1
NUP54	0.9197	-0.2016	1
NUP88	0.9652	-0.0590	2
NUP93	0.4989	-0.2933	3
NUTF2	0.7189	0.2149	1
NXF1	0.7671	-0.2963	1
NXPH3	0.8163	-0.2862	1
NXPH4	0.9171	-0.1370	1
OAS3	0.0634	-0.8352	1

Table S9: Non-significant synaptic proteins of cerebellar vermis in children with idiopathic autism (FDR-adjusted p > 0.05) *(continued)*

Gene name	FDR-adjusted p-value	log2(fold-change)	Number of peptides
OCIAD1	0.9372	-0.1707	1
OCRL	0.9347	0.0825	1
OGA	0.9784	0.0267	2
OGDHL	0.8163	-0.1188	5
OGT	0.9555	-0.0378	3
OLA1	0.5900	0.2081	4
OLFM1	0.8833	-0.1811	1
OLFM3	0.9514	-0.0654	2
OMG	0.9555	-0.0546	2
OPA1	0.6902	0.1914	7
OPCML	0.5858	-0.2023	5
OPHN1	0.6902	0.3499	1
OPLAH	0.8487	0.2279	1
OPTN	0.7928	-0.2510	2
OSBPL11	0.9966	0.0037	1
OSBPL2	0.9001	-0.4181	1
OSBPL9	0.9556	-0.0999	1
OSTF1	0.9353	-0.1058	1
OTUB1	0.9652	0.0962	1
OTUD7B	0.9555	-0.0584	2
OXCT1	0.0709	0.6410	5
OXR1	0.9053	-0.1847	2
OXSR1	0.6565	0.2023	3
P00761	0.9927	-0.0125	3
P4HB	0.3168	0.2594	4
PA2G4	0.6903	-0.1503	4
PABPC4	0.3202	-0.3388	4
PACS1	0.6071	-0.1602	5
PACS2	0.9482	0.0463	1
PACSIN1	0.9217	0.0478	8
PACSIN2	0.9211	-0.0625	6
PADI2	0.9555	0.0940	2
PAFAH1B1	0.9908	-0.0120	5
PAFAH1B3	0.9555	0.0871	3
PAICS	0.9480	0.0809	2
PAK1	0.9652	0.0345	2
PAK2	0.8278	0.1358	2
PALM	0.8345	0.1117	9
PANK4	0.8833	0.3580	1
PARK7	0.9053	0.1010	3
PARP1	0.8447	-0.1511	3
PARVA	0.6884	0.1937	2
PATJ	0.9947	0.0087	1
PATL1	0.4754	-0.2128	1

Table S9: Non-significant synaptic proteins of cerebellar vermis in children with idiopathic autism (FDR-adjusted p > 0.05) *(continued)*

Gene name	FDR-adjusted p-value	log2(fold-change)	Number of peptides
PAXX	0.8606	-0.3016	1
PBXIP1	0.9217	0.1244	2
PC	0.9784	-0.0331	4
PCBD2	0.9217	0.1280	1
PCBP1	0.9561	-0.0737	2
PCBP2	0.8587	-0.1488	4
PCBP3	0.2951	-0.2537	8
PCCA	0.9211	0.1231	2
PCCB	0.8989	-0.1307	3
PCDH1	0.4178	-0.3916	1
PCDHGB6	0.9819	0.0269	1
PCDHGC3	0.8333	-0.4449	1
PCK2	0.9819	0.0641	1
PCLO	0.7570	0.1346	9
PCMT1	0.4487	0.8795	1
PCNA	0.4995	-0.3575	1
PCNP	0.4874	-0.4030	2
PCNX4	0.4807	-0.4205	1
PCP4L1	0.9217	-0.1673	1
PCYOX1	0.7882	0.2535	1
PCYT2	0.9966	-0.0030	2
PDAP1	0.3197	-0.5657	1
PDCD10	0.8110	0.1700	2
PDCD4	0.5162	-0.5470	1
PDCD6IP	0.4989	0.1521	13
PDE12	0.9217	0.1838	1
PDE1A	0.9484	-0.0639	3
PDHA1	0.3168	0.2011	8
PDHB	0.6575	0.1156	11
PDHX	0.4798	0.1567	8
PDIA3	0.8832	-0.0974	8
PDIA4	0.8926	0.1483	3
PDIA5	0.9784	0.0592	1
PDIA6	0.6795	-0.2429	3
PDK3	0.9366	0.2114	1
PDLIM1	0.9217	-0.1425	1
PDLIM5	0.6902	-0.6360	1
PDP1	0.9217	-0.2751	1
PDPK1	0.9283	0.2353	1
PDPR	0.9784	0.0305	1
PDS5A	0.9555	-0.0651	1
PDS5B	0.7643	-0.2249	2
PDXP	0.9484	0.1331	1
PEA15	0.5954	0.9121	2

Table S9: Non-significant synaptic proteins of cerebellar vermis in children with idiopathic autism (FDR-adjusted p > 0.05) *(continued)*

Gene name	FDR-adjusted p-value	log2(fold-change)	Number of peptides
PEBP1	0.8345	-0.1332	5
PECR	0.9610	0.0434	1
PELI2	0.6565	-0.1908	1
PEX14	0.8043	-0.3003	1
PEX19	0.6163	-0.3677	1
PFDN1	0.9383	-0.0858	2
PFDN5	0.9911	0.0187	1
PFDN6	0.9646	0.0420	2
PFKFB2	0.9555	0.0828	1
PFKL	0.8989	-0.1555	5
PFKM	0.7643	-0.0966	11
PFKP	0.7670	-0.1613	8
PFN1	0.2163	0.5463	5
PFN2	0.8397	0.1624	6
PGAM1	0.6490	0.2844	5
PGAM5	0.9484	-0.1155	1
PGAP1	0.6575	-0.3280	1
PGD	0.2885	0.2494	5
PGGT1B	0.8841	0.2021	1
PGK1	0.1340	0.3925	8
PGLS	0.9298	0.1789	1
PGM1	0.4339	0.5193	2
PGM2L1	0.8695	-0.3174	1
PGM3	0.6902	0.2526	2
PGRMC1	0.9102	0.0870	4
PHAX	0.6423	-0.3173	1
PHB	0.0808	0.2491	10
PHF1	0.9366	-0.1649	1
PHF24	0.9422	0.1194	1
PHGDH	0.6177	0.1255	21
PHKA1	0.9018	-0.1818	1
PHKB	0.8300	-0.1683	4
PHLDA1	0.9555	-0.0690	1
PHLDB1	0.9555	-0.1596	1
PHPT1	0.9217	0.1938	2
PHYHIP	0.7436	-0.2937	4
PHYHIPL	0.9784	-0.0241	4
PI4KA	0.3197	-0.3246	5
PI4KB	0.8345	-0.1569	1
PICALM	0.9353	0.0976	2
PICK1	0.9211	-0.1995	1
PIGR	0.9784	-0.0672	1
PIGT	0.9555	-0.0598	1
PIH1D1	0.8095	0.3127	1

Table S9: Non-significant synaptic proteins of cerebellar vermis in children with idiopathic autism (FDR-adjusted p > 0.05) *(continued)*

Gene name	FDR-adjusted p-value	log2(fold-change)	Number of peptides
PIK3C2A	0.9217	-0.1591	1
PIK3CA	0.5208	-0.4775	1
PIK3R2	0.8207	-0.5269	1
PIK3R4	0.1426	-0.4662	1
PIN1	0.9376	-0.1884	1
PIP	0.9591	-0.0587	3
PIP4K2A	0.8125	-0.2830	2
PIP4K2C	0.9919	0.0186	2
PIP5K1C	0.8110	-0.2548	1
PIPOX	0.9261	-0.1544	1
PIPSL	0.4117	-0.2644	2
PITHD1	0.9777	-0.0708	1
PITPNA	0.9217	0.2141	1
PITPNC1	0.9555	-0.1696	1
PITPNM1	0.5495	-0.3558	1
PITPNM3	0.6850	-0.4630	1
PKM	0.6795	0.0803	25
PKP1	0.9555	-0.1093	1
PKP4	0.7834	-0.2882	2
PLA2G4D	0.7643	0.3952	1
PLAA	0.9839	0.0221	1
PLBD2	0.9610	-0.0963	1
PLCB3	0.9217	0.1072	1
PLCB4	0.9555	0.0407	4
PLCD1	0.9376	-0.0859	2
PLCD3	0.9217	0.2839	1
PLCH2	0.8833	0.1627	1
PLCL1	0.9819	0.0314	2
PLCL2	0.9217	-0.2367	1
PLD2	0.9454	-0.1134	1
PLD3	0.9839	-0.0347	1
PLEKHA5	0.7557	0.5447	1
PLEKHD1	0.5954	-0.3496	2
PLEKHH3	0.9482	-0.2048	1
PLEKHM2	0.9911	0.0199	1
PLG	0.9217	-0.1028	1
PLP1	0.5543	0.3132	11
PLPP3	0.6423	-0.2894	3
PLSCR4	0.5635	-0.2644	1
PLXNA1	0.7537	-0.1949	2
PLXNA4	0.8207	-0.2211	1
PLXNB2	0.9422	-0.1154	1
PLXNC1	0.7122	-0.2779	2
PMP2	0.9217	-0.2231	3

Table S9: Non-significant synaptic proteins of cerebellar vermis in children with idiopathic autism (FDR-adjusted p > 0.05) *(continued)*

Gene name	FDR-adjusted p-value	log2(fold-change)	Number of peptides
PMPCA	0.7333	-0.4303	1
PMVK	0.8833	-0.1925	2
PNP	0.7938	0.1923	3
PNPLA8	0.8144	0.2486	1
PNPT1	0.9784	-0.0320	3
POF1B	0.9217	-0.0895	3
POLB	0.8578	-0.2664	1
POLR2B	0.8940	-0.1647	1
POR	0.8464	-0.1023	3
PPA1	0.6574	0.5195	1
PPA2	0.9819	0.0327	1
PPCS	0.9966	0.0052	1
PPFIA1	0.7938	-0.2287	1
PPFIA3	0.9819	0.0317	3
PPIA	0.5253	0.3236	7
PPIB	0.9311	-0.0437	6
PPID	0.8989	-0.1985	2
PPIF	0.5985	0.3031	3
PPIH	0.8333	0.2475	2
PPIL1	0.9555	-0.1545	1
PPM1H	0.9555	0.0905	1
PPME1	0.8656	0.3154	1
PPP1CA	0.9819	0.0268	1
PPP1R12C	0.9736	-0.0439	1
PPP1R9B	0.5688	-0.5425	1
PPP2CA	0.9217	-0.1534	2
PPP2R1A	0.7354	-0.1278	6
PPP2R2A	0.9947	-0.0076	3
PPP2R5B	0.9784	-0.0367	1
PPP2R5D	0.9819	0.0240	2
PPP3CA	0.9819	0.0141	4
PPP3CB	0.9934	0.0110	1
PPP3CC	0.7822	0.2958	1
PPP5C	0.9404	0.0817	1
PPT1	0.6012	-0.4200	1
PRDX2	0.9217	0.0474	8
PRDX3	0.9805	0.0399	3
PRDX4	0.4364	0.3553	2
PRDX5	0.4178	0.3874	8
PRDX6	0.2263	0.3428	5
PREPL	0.8513	-0.1881	3
PREX1	0.9217	-0.1384	3
PREX2	0.9129	-0.1168	1
PRKACA	0.9947	0.0126	1

Table S9: Non-significant synaptic proteins of cerebellar vermis in children with idiopathic autism (FDR-adjusted p > 0.05) *(continued)*

Gene name	FDR-adjusted p-value	log2(fold-change)	Number of peptides
PRKACB	0.9947	-0.0060	3
PRKAG1	0.8447	-0.2260	1
PRKAG2	0.9819	0.0204	2
PRKAR1A	0.9197	-0.1481	1
PRKAR2A	0.6423	0.4769	1
PRKAR2B	0.9366	0.2803	1
PRKCA	0.9217	0.2055	2
PRKCB	0.9261	-0.1144	2
PRKCD	0.9819	-0.0240	3
PRKCE	0.8507	-0.4497	1
PRKCG	0.9911	0.0259	1
PRKCQ	0.8833	-0.0775	1
PRKG1	0.6902	-0.4732	1
PRKRA	0.5776	-0.2916	4
PRMT1	0.7938	-0.5171	1
PRMT5	0.5653	-0.5154	1
PRPF31	0.2642	-0.3679	1
PRPF40A	0.9652	-0.0380	1
PRPF4B	0.9217	0.2285	1
PRPF6	0.8898	-0.1450	1
PRPF8	0.3239	-0.4666	2
PRPS1	0.4241	-0.3582	3
PRPSAP1	0.1426	-0.4947	2
PRPSAP2	0.3990	-0.3973	3
PRTFDC1	0.3713	-0.3469	1
PRUNE1	0.9555	0.0604	2
PRXL2A	0.9911	-0.0190	1
PSAP	0.9555	-0.1141	1
PSAPL1	0.9274	0.1738	2
PSD2	0.8833	-0.1583	1
PSD3	0.7834	0.0998	13
PSIP1	0.9217	-0.2360	1
PSMA1	0.5543	-0.1521	6
PSMA2	0.9966	0.0018	4
PSMA3	0.9916	-0.0090	4
PSMA4	0.9483	-0.0524	4
PSMA5	0.6902	0.2252	2
PSMA6	0.9217	-0.0957	3
PSMA7	0.9555	0.0649	1
PSMB2	0.9211	0.1870	2
PSMB3	0.9819	-0.0271	2
PSMB4	0.9392	-0.0698	4
PSMB5	0.9468	-0.0582	3
PSMB6	0.4989	-0.2401	4

Table S9: Non-significant synaptic proteins of cerebellar vermis in children with idiopathic autism (FDR-adjusted p > 0.05) *(continued)*

Gene name	FDR-adjusted p-value	log2(fold-change)	Number of peptides
PSMB7	0.9171	0.1287	2
PSMB8	0.9646	0.0459	1
PSMB9	0.9422	-0.0660	1
PSMC1	0.8841	-0.1964	2
PSMC2	0.5145	-0.3058	5
PSMC3	0.9676	0.1188	1
PSMC4	0.9468	-0.0582	4
PSMC5	0.9217	-0.0952	3
PSMC6	0.7670	-0.1995	3
PSMD1	0.1903	-0.2712	7
PSMD10	0.9569	-0.1445	1
PSMD11	0.6230	-0.1998	5
PSMD12	0.9217	-0.1221	2
PSMD14	0.9652	-0.0434	2
PSMD2	0.4745	-0.2062	5
PSMD3	0.9211	-0.1312	2
PSMD5	0.9382	0.1283	1
PSMD7	0.7843	-0.1353	2
PSMD8	0.0573	-0.3410	6
PSME2	0.6903	-0.2525	1
PTBP1	0.4624	-0.3469	3
PTBP2	0.2224	-0.6782	2
PTEN	0.9555	-0.1094	1
PTGES2	0.9772	-0.0238	3
PTGES3	0.8400	0.2552	1
PTGR1	0.9694	0.0347	2
PTGR2	0.6313	0.4379	1
PTK2B	0.9709	0.0315	3
PTMS	0.8513	-0.1902	2
PTP4A2	0.5996	-0.2779	2
PTPMT1	0.9211	-0.1885	1
PTPN1	0.5495	-0.3513	2
PTPN11	0.9947	-0.0033	3
PTPN23	0.9482	0.0569	3
PTPN9	0.9792	0.0299	1
PTPRA	0.8871	-0.2304	1
PTPRD	0.9819	0.0441	1
PTPRE	0.9446	0.1194	1
PTPRG	0.7643	-0.3383	1
PTPRS	0.8051	-0.1466	1
PUF60	0.3956	-0.2370	3
PUM1	0.9482	-0.1078	1
PURA	0.0674	-0.4436	8
PURB	0.6869	-0.1350	4

Table S9: Non-significant synaptic proteins of cerebellar vermis in children with idiopathic autism (FDR-adjusted p > 0.05) *(continued)*

Gene name	FDR-adjusted p-value	log2(fold-change)	Number of peptides
PURG	0.8832	-0.3904	1
PXK	0.9934	-0.0070	2
PXN	0.9839	0.0121	2
PYCR1	0.6706	0.8039	1
PYGB	0.7980	0.1286	15
PYGM	0.8510	0.1139	10
QARS	0.6850	-0.5769	1
QDPR	0.7834	0.1293	7
QRSL1	0.9561	-0.0492	1
RAB10	0.6953	0.1777	4
RAB11A	0.3965	0.2665	3
RAB12	0.4358	-0.2184	1
RAB14	0.9217	-0.0670	4
RAB18	0.9311	-0.0973	3
RAB1A	0.9652	0.0561	3
RAB23	0.8345	0.1503	3
RAB24	0.9811	0.0548	2
RAB27A	0.9283	-0.1348	1
RAB28	0.8487	-0.1929	2
RAB29	0.6423	-0.6482	1
RAB2B	0.9784	-0.0398	2
RAB30	0.9555	-0.0888	2
RAB31	0.8841	-0.1635	1
RAB33A	0.9966	0.0057	1
RAB33B	0.9480	-0.2033	1
RAB35	0.9736	-0.0474	3
RAB39B	0.9366	-0.1462	2
RAB3A	0.9214	0.1304	1
RAB3C	0.8634	0.1521	3
RAB3GAP2	0.9217	-0.0778	6
RAB3IP	0.8578	-0.1617	2
RAB4A	0.9217	-0.1038	1
RAB4B	0.9839	-0.0244	1
RAB5A	0.7354	0.2562	1
RAB5B	0.9217	-0.0714	3
RAB5C	0.1866	0.3088	5
RAB6B	0.5451	0.1815	5
RAB7A	0.6490	0.2057	4
RAB8A	0.9211	-0.1866	1
RAB9A	0.8993	-0.3280	1
RABGAP1	0.8447	0.2937	1
RABGAP1L	0.9217	-0.1118	1
RABGEF1	0.9047	-0.0977	1
RABGGTA	0.8051	-0.2719	2

Table S9: Non-significant synaptic proteins of cerebellar vermis in children with idiopathic autism (FDR-adjusted p > 0.05) *(continued)*

Gene name	FDR-adjusted p-value	log2(fold-change)	Number of peptides
RABIF	0.7436	-0.5394	1
RABL3	0.9864	-0.0443	1
RAC1	0.9784	0.0143	5
RACK1	0.6113	-0.2968	1
RAD1	0.7951	-0.2747	1
RAD21	0.7156	-0.3118	1
RAD23A	0.8947	0.0984	5
RAD23B	0.4702	0.2676	1
RALA	0.9819	0.0116	4
RALGAPA1	0.2224	-0.3837	1
RALGAPB	0.7354	-0.3339	1
RAN	0.8303	0.2340	2
RANBP1	0.9482	0.0858	1
RANBP2	0.4487	-0.2501	2
RANBP6	0.9482	-0.0747	1
RANBP9	0.9819	0.0188	1
RAP1B	0.9555	0.0465	2
RAP1GAP	0.7586	-0.3125	1
RAP1GAP2	0.9283	-0.1139	3
RAP1GDS1	0.7991	0.1075	8
RAP2A	0.5705	-0.5104	2
RAPGEF4	0.8125	-0.1755	6
RARS	0.7499	-0.2814	2
RASA3	0.6575	-0.2957	2
RASGRF1	0.7000	-0.6297	1
RBBP5	0.9927	-0.0220	1
RBBP7	0.7436	-0.2087	1
RBFOX3	0.8419	-0.4270	1
RBM14	0.9785	-0.0380	1
RBM39	0.8728	0.2422	1
RBMXL1	0.6423	-0.7103	1
RCN1	0.9366	-0.1071	2
RDH11	0.8043	-0.2913	2
RECQL	0.3197	-0.3724	3
REELD1	0.8367	0.2413	1
REEP5	0.9555	-0.1361	1
RELCH	0.9885	0.0179	2
REPS1	0.7580	0.2063	2
REPS2	0.8093	0.1841	1
REXO2	0.8964	0.3130	1
RFTN2	0.9353	-0.0804	1
RGS17	0.9347	-0.3626	1
RHEB	0.9966	0.0029	4
RHOC	0.5191	0.4409	1

Table S9: Non-significant synaptic proteins of cerebellar vermis in children with idiopathic autism (FDR-adjusted p > 0.05) *(continued)*

Gene name	FDR-adjusted p-value	log2(fold-change)	Number of peptides
RHOT1	0.7938	-0.2590	1
RHOT2	0.9283	-0.1297	1
RIC8A	0.9819	0.0181	2
RICTOR	0.8670	-0.2495	2
RIDA	0.8989	0.0776	2
RIMS1	0.3702	-0.3443	3
RMDN3	0.9142	0.1559	3
RND2	0.9569	-0.2608	1
RNF123	0.6626	0.1560	1
RNF20	0.9966	-0.0042	1
RNF40	0.7928	-0.3268	1
RNH1	0.9422	0.0867	3
RNMT	0.4931	-0.4336	2
RO60	0.2995	0.4308	2
ROCK1	0.9885	0.0231	1
ROCK2	0.9736	0.0232	5
ROGDI	0.7580	0.2525	2
RP2	0.6113	0.4515	1
RPA1	0.5579	-0.5391	1
RPA2	0.8602	-0.5371	1
RPAP3	0.7122	-0.2362	1
RPH3A	0.7479	-0.2205	1
RPL10A	0.9555	-0.0650	2
RPL11	0.3713	-0.3052	5
RPL12	0.6795	-0.1454	5
RPL13	0.9819	0.0549	1
RPL13A	0.8871	-0.0996	4
RPL14	0.9784	-0.0698	1
RPL15	0.8447	-0.2489	1
RPL17	0.9217	0.2905	1
RPL18	0.9376	-0.1353	2
RPL18A	0.8270	0.1925	1
RPL19	0.8542	-0.1817	1
RPL21	0.8447	-0.2161	2
RPL22	0.8989	-0.2189	2
RPL23	0.9555	0.0532	5
RPL23A	0.9482	-0.0476	4
RPL24	0.5635	-0.2968	4
RPL27	0.5892	-0.2852	2
RPL28	0.9217	-0.2429	1
RPL29	0.7354	-0.5591	1
RPL3	0.6177	-0.2419	4
RPL30	0.7499	-0.4636	1
RPL31	0.8989	-0.4815	2

Table S9: Non-significant synaptic proteins of cerebellar vermis in children with idiopathic autism (FDR-adjusted p > 0.05) *(continued)*

Gene name	FDR-adjusted p-value	log2(fold-change)	Number of peptides
RPL34	0.6006	-0.4235	2
RPL35	0.7844	-0.4553	2
RPL36	0.5543	-0.5761	1
RPL38	0.2883	-0.3932	2
RPL4	0.6731	-0.2130	6
RPL5	0.5954	-0.3869	1
RPL6	0.7643	-0.1460	6
RPL7	0.7000	-0.1626	9
RPL7A	0.9561	-0.0475	5
RPL8	0.9844	-0.0321	1
RPL9	0.9405	-0.1390	2
RPN1	0.6490	-0.1065	7
RPN2	0.9482	-0.0979	1
RPP30	0.9217	-0.1231	1
RPRD1B	0.4594	-0.5592	1
RPS10	0.5120	-0.3256	2
RPS11	0.4487	-0.2244	6
RPS13	0.9784	-0.0680	1
RPS14	0.8480	-0.4075	1
RPS16	0.9075	-0.1275	4
RPS18	0.8871	-0.2097	1
RPS19	0.7251	-0.1409	7
RPS2	0.8989	-0.1120	3
RPS20	0.5954	-0.3780	1
RPS21	0.3194	-0.9661	1
RPS23	0.9999	-0.0003	1
RPS25	0.5639	-0.2253	4
RPS26	0.9934	0.0158	1
RPS28	0.0674	-0.6748	2
RPS3	0.2436	-0.2146	9
RPS3A	0.6423	-0.2353	3
RPS4X	0.9555	0.0719	3
RPS5	0.8885	-0.2227	1
RPS6KA1	0.9652	0.0931	1
RPS6KA2	0.7479	0.2616	1
RPS6KA3	0.8894	-0.1344	3
RPS7	0.9261	-0.1159	2
RPS8	0.8841	-0.2256	2
RPS9	0.9211	-0.1001	4
RPSA	0.2323	-0.6158	2
RPTOR	0.8276	-0.1977	2
RRAGC	0.7675	-0.2767	2
RRAS2	0.7586	0.2753	1
RRBP1	0.5145	-0.2569	5

Table S9: Non-significant synaptic proteins of cerebellar vermis in children with idiopathic autism (FDR-adjusted p > 0.05) *(continued)*

Gene name	FDR-adjusted p-value	log2(fold-change)	Number of peptides
RSL1D1	0.4471	-0.6659	1
RSU1	0.8201	-0.1495	3
RTCB	0.2702	-0.4380	5
RTN1	0.8841	0.0897	2
RTN4	0.7844	0.1786	5
RUFY1	0.8447	-0.1514	1
RUFY2	0.9283	-0.0764	3
RUFY3	0.7603	-0.1257	7
RUVBL1	0.7834	-0.1053	6
RUVBL2	0.3197	-0.4337	2
RYR2	0.5543	-0.2813	3
S100A13	0.9889	0.0125	3
S100A14	0.9709	-0.0702	1
S100A4	0.9482	0.0697	1
S100A6	0.5781	0.5046	1
S100A7	0.9819	-0.0519	2
S100A8	0.6375	0.2877	4
S100A9	0.8144	0.2019	3
S100P	0.9604	0.1047	1
SACM1L	0.7671	-0.2309	4
SAE1	0.3865	-0.4714	3
SAFB	0.9366	-0.0854	2
SAMM50	0.8989	0.1322	1
SAP18	0.9376	-0.2483	1
SAR1A	0.9610	-0.0701	3
SARDH	0.9819	0.0292	1
SARM1	0.8695	-0.1304	3
SARS	0.6903	-0.2036	4
SARS2	0.9217	0.1619	1
SBDS	0.9555	0.0432	4
SBF1	0.6850	-0.1783	6
SBF2	0.9589	0.1309	1
SBNO1	0.9602	-0.0813	1
SCAMP1	0.9652	-0.0507	1
SCAMP5	0.6461	-0.4700	1
SCCPDH	0.4385	-0.3308	2
SCFD1	0.9405	-0.0739	2
SCGB1D1	0.9589	0.1159	1
SCGB1D2	0.6423	-0.2652	2
SCGB2A2	0.9819	0.0585	1
SCGN	0.9468	0.0779	3
SCIN	0.4504	0.3519	2
SCN1A	0.6902	-0.1505	4
SCN2A	0.8110	-0.1872	2

Table S9: Non-significant synaptic proteins of cerebellar vermis in children with idiopathic autism (FDR-adjusted p > 0.05) *(continued)*

Gene name	FDR-adjusted p-value	log2(fold-change)	Number of peptides
SCNM1	0.9885	-0.0768	1
SCP2	0.8634	0.2337	1
SCPEP1	0.9480	0.1420	1
SCRN1	0.8110	0.1793	5
SDR39U1	0.9014	0.3186	1
SDS	0.8894	-0.1375	1
SDSL	0.9217	0.1362	2
SEC14L2	0.7070	0.2674	3
SEC16A	0.9757	0.0973	1
SEC22B	0.4548	-0.2372	8
SEC23A	0.7822	-0.1868	4
SEC24B	0.9610	-0.0927	1
SEC24C	0.4487	-0.3945	4
SEC31A	0.7834	-0.1864	3
SEC61A1	0.9217	-0.0671	1
SEC62	0.1816	-0.3971	1
SEH1L	0.8042	-0.1886	1
SEL1L	0.5635	-0.3853	1
SELENBP1	0.9555	-0.0864	1
SELENOH	0.9784	0.0393	1
SEMG1	0.9211	0.0864	9
SEMG2	0.9805	-0.0285	5
SEPHS1	0.6490	-0.3445	1
SEPSECS	0.7072	0.3121	1
SEPTIN10	0.7675	-0.1416	3
SEPTIN11	0.6423	-0.4379	2
SEPTIN2	0.4084	-0.1745	8
SEPTIN4	0.3297	-0.3242	5
SEPTIN5	0.6585	-0.1435	7
SEPTIN7	0.6452	-0.1351	7
SEPTIN8	0.5495	-0.2525	2
SEPTIN9	0.6310	-0.1370	7
SERPINA12	0.8949	0.0878	8
SERPINB1	0.8529	0.2683	1
SERPINB12	0.9217	-0.1210	4
SERPINB13	0.6423	0.3610	1
SERPINB14	0.9376	0.1620	1
SERPINB3	0.8040	0.2555	2
SERPINB4	0.4248	0.4262	7
SERPINB7	0.9758	-0.0643	1
SERPINE2	0.9708	-0.0296	2
SERPINF1	0.7487	0.3942	1
SESN1	0.8301	-0.3125	1
SESTD1	0.9966	-0.0066	1

Table S9: Non-significant synaptic proteins of cerebellar vermis in children with idiopathic autism (FDR-adjusted p > 0.05) *(continued)*

Gene name	FDR-adjusted p-value	log2(fold-change)	Number of peptides
SETD7	0.5543	0.5161	1
SF3A3	0.5145	-0.4056	2
SF3B3	0.6113	-0.1964	7
SFN	0.9916	0.0131	3
SFXN3	0.9891	-0.0179	2
SGCD	0.9652	-0.0605	1
SGIP1	0.9784	-0.0214	2
SGSM1	0.7938	-0.2332	1
SGTA	0.7583	0.2167	2
SH3BGRL	0.7589	-0.2428	1
SH3BGRL2	0.9217	-0.2304	1
SH3BGRL3	0.9484	0.0694	1
SH3GL1	0.1277	0.5434	3
SH3GL2	0.5191	0.1933	7
SH3GL3	0.3800	0.3155	6
SH3GLB1	0.9482	0.1025	1
SH3GLB2	0.9267	0.0925	3
SHANK1	0.7621	-0.1010	7
SHANK2	0.9376	-0.0628	1
SHF	0.9482	0.0672	1
SHFL	0.8869	-0.2146	1
SHISA6	0.9555	-0.1448	1
SHMT2	0.6423	-0.1696	5
SHTN1	0.9708	0.0668	2
SIPA1L3	0.5996	-0.3387	1
SIRPA	0.9468	-0.0796	1
SIRT2	0.6795	0.2854	4
SKAP2	0.9927	-0.0114	1
SKIV2L	0.7354	-0.1622	1
SKP1	0.9053	-0.1757	1
SLC12A2	0.7938	-0.1346	5
SLC12A4	0.4248	-0.5684	1
SLC12A5	0.6795	-0.2522	8
SLC12A6	0.6163	-0.2654	1
SLC12A9	0.9916	0.0145	1
SLC1A3	0.8964	-0.1679	1
SLC20A2	0.9217	-0.2166	1
SLC25A11	0.9468	0.0683	7
SLC25A12	0.6858	-0.2528	4
SLC25A20	0.9217	-0.0816	1
SLC25A22	0.9649	0.0805	2
SLC25A23	0.7333	-0.3511	2
SLC25A3	0.8051	-0.1464	7
SLC25A4	0.9211	-0.1328	3

Table S9: Non-significant synaptic proteins of cerebellar vermis in children with idiopathic autism (FDR-adjusted p > 0.05) *(continued)*

Gene name	FDR-adjusted p-value	log2(fold-change)	Number of peptides
SLC25A42	0.9217	0.0770	1
SLC25A5	0.9839	-0.0236	4
SLC25A6	0.9555	0.0352	18
SLC27A1	0.7834	-0.2056	1
SLC30A9	0.9171	0.2817	1
SLC39A10	0.6751	0.3397	1
SLC3A2	0.9652	0.0366	2
SLC43A2	0.7772	-0.3718	1
SLC4A1	0.7179	-0.3917	2
SLC4A10	0.9976	-0.0013	2
SLC4A4	0.9652	0.1053	1
SLC6A11	0.9217	0.1990	1
SLC8A1	0.9217	0.2159	1
SLC8A2	0.9821	0.0320	2
SLC9A3R1	0.8670	-0.2462	1
SLIRP	0.6575	-0.4243	1
SLITRK2	0.8738	-0.0648	1
SLK	0.9690	-0.0437	2
SLMAP	0.9217	-0.1451	1
SMAD4	0.9652	-0.1479	1
SMARCA1	0.6574	0.3409	1
SMARCC2	0.8940	-0.2190	1
SMC1A	0.8380	-0.1467	3
SMC3	0.8848	-0.1236	2
SMCHD1	0.6903	0.3109	1
SMCR8	0.9891	0.0304	1
SMS	0.9610	-0.0860	2
SMU1	0.9819	-0.0231	1
SNAP25	0.9211	0.1374	2
SNAP29	0.9695	0.0593	1
SNAP91	0.5543	-0.2653	4
SNCA	0.6375	0.2791	3
SNCB	0.8110	0.1103	5
SNCG	0.9652	-0.0422	3
SND1	0.3601	-0.3105	7
SNRNP200	0.9211	0.2355	1
SNRPA	0.9555	-0.0826	2
SNRPB	0.8936	-0.1297	1
SNRPB2	0.1057	-0.3912	1
SNRPC	0.8487	-0.3683	1
SNRPD2	0.9217	-0.1631	1
SNRPD3	0.9708	-0.0377	2
SNRPE	0.7208	-0.2804	1
SNTA1	0.9217	-0.1511	2

Table S9: Non-significant synaptic proteins of cerebellar vermis in children with idiopathic autism (FDR-adjusted p > 0.05) *(continued)*

Gene name	FDR-adjusted p-value	log2(fold-change)	Number of peptides
SNTB1	0.8916	0.0866	6
SNTB2	0.9482	0.0646	1
SNX1	0.9819	-0.0253	4
SNX12	0.5495	0.5099	1
SNX15	0.9211	0.2144	1
SNX18	0.9555	0.1025	1
SNX2	0.9482	-0.0663	4
SNX27	0.9261	0.1202	2
SNX3	0.9443	-0.1390	2
SNX5	0.6423	-0.1703	2
SNX6	0.7822	-0.2039	2
SOD2	0.8820	0.1480	2
SOGA1	0.7483	-0.1945	2
SOGA3	0.7222	-0.1699	5
SORBS1	0.9217	0.0998	2
SORBS2	0.8989	-0.1751	1
SORCS2	0.7931	-0.0961	3
SORD	0.5789	0.3761	1
SPAG1	0.9555	-0.0599	1
SPART	0.9484	-0.0637	2
SPATA20	0.9715	0.0305	4
SPRR3	0.9410	-0.0958	1
SPTA1	0.7951	-0.1607	7
SPTB	0.2047	-0.2309	11
SPTBN1	0.9211	-0.0272	43
SPTBN4	0.9217	-0.0682	4
SRC	0.8606	-0.1224	1
SRCIN1	0.1423	0.2706	11
SRGAP1	0.9053	-0.1954	1
SRGAP2	0.5378	-0.3135	4
SRGAP3	0.6540	-0.1998	3
SRI	0.7643	0.3714	1
SRP14	0.9819	0.0203	2
SRP54	0.5483	-0.1754	5
SRP72	0.4487	-0.3434	1
SRP9	0.1816	-0.3574	3
SRPK2	0.9350	-0.1824	1
SRPRA	0.7487	-0.3709	1
SRPRB	0.7601	0.2378	1
SRSF1	0.8989	-0.3804	1
SRSF11	0.4487	-0.3203	1
SRSF3	0.9934	-0.0124	1
SRSF4	0.7436	-0.5773	1
SRSF8	0.8270	0.2032	1

Table S9: Non-significant synaptic proteins of cerebellar vermis in children with idiopathic autism (FDR-adjusted p > 0.05) *(continued)*

Gene name	FDR-adjusted p-value	log2(fold-change)	Number of peptides
SSB	0.7621	-0.1639	3
SSBP1	0.6505	-0.2762	3
ST13	0.9014	0.0660	6
STAG1	0.5162	-0.5170	1
STAM	0.6260	0.3209	4
STAM2	0.5543	-0.1778	1
STAMBP	0.6540	-0.1905	2
STARD10	0.9555	0.1436	1
STAT1	0.8841	0.1417	1
STAT3	0.9555	-0.0990	1
STAU1	0.9966	0.0018	4
STAU2	0.8088	-0.2253	2
STIP1	0.8563	-0.0889	4
STK33	0.9836	0.0231	1
STK39	0.9784	-0.0705	2
STMN1	0.9353	-0.1148	2
STMN3	0.9819	0.0447	1
STOM	0.6902	-0.3561	2
STON2	0.6922	-0.1964	1
STRAP	0.5826	-0.4093	3
STRBP	0.7022	-0.4519	1
STRIP1	0.5253	-0.3432	2
STRN3	0.9839	0.0214	1
STT3A	0.5892	-0.4303	1
STUB1	0.7291	0.2447	3
STX12	0.9217	-0.0794	3
STX17	0.6071	0.4023	1
STX1A	0.9555	0.1296	1
STX1B	0.7148	0.2257	6
STX5	0.4931	-0.2589	3
STX7	0.9053	-0.1950	1
STXBP1	0.7350	-0.1480	18
STXBP3	0.8051	-0.1445	6
STXBP5	0.8871	0.1978	1
SUCLA2	0.8095	0.1055	18
SUCLG1	0.8634	0.1273	3
SUCLG2	0.7930	0.2896	3
SULT4A1	0.9376	-0.0901	2
SUMO3	0.9366	0.1620	1
SUN1	0.7189	-0.2593	1
SV2A	0.8783	-0.2968	1
SV2B	0.7189	0.1883	1
SVIP	0.6490	0.4916	1
SYN1	0.8110	-0.0662	17

Table S9: Non-significant synaptic proteins of cerebellar vermis in children with idiopathic autism (FDR-adjusted p > 0.05) *(continued)*

Gene name	FDR-adjusted p-value	log2(fold-change)	Number of peptides
SYN2	0.8670	-0.0695	9
SYN3	0.9053	-0.0794	3
SYNCRIP	0.9708	-0.0446	1
SYNGAP1	0.9784	-0.0295	2
SYNJ1	0.7000	-0.0899	13
SYNPO2	0.8464	-0.2392	1
SYP	0.2960	0.6330	1
SYT12	0.7834	-0.2094	4
SYT2	0.1167	-0.3002	11
SYT3	0.6744	-0.2299	2
SYT7	0.9646	0.0412	2
TAB1	0.8833	0.1531	1
TAB3	0.9555	0.1594	1
TACO1	0.9708	0.0568	1
TAGLN	0.9792	0.0524	2
TAGLN2	0.4243	0.6625	1
TAGLN3	0.7643	0.1364	8
TALDO1	0.4487	0.2749	7
TANC2	0.9966	-0.0035	1
TAOK1	0.9217	-0.1516	2
TARBP1	0.6423	-1.2044	1
TARDBP	0.9366	-0.3311	1
TARS	0.9555	-0.0399	4
TBC1D15	0.9819	-0.0324	2
TBC1D17	0.9604	0.0718	1
TBC1D9	0.9784	0.0583	1
TBC1D9B	0.9934	0.0049	4
TBCA	0.9217	-0.1519	1
TBCB	0.9646	-0.0644	3
TBCD	0.6585	-0.5311	2
TBCE	0.8838	0.1144	3
TBCEL	0.9366	-0.1266	1
TBL2	0.5653	-0.5326	1
TCAF1	0.5321	-0.4192	1
TCEA1	0.9405	-0.1509	1
TCEAL2	0.3288	-0.8909	1
TCEAL5	0.1968	-0.6703	1
TCERG1	0.9353	-0.0914	1
TCOF1	0.9784	-0.0883	1
TECPR2	0.8871	-0.1794	1
TFAM	0.3956	0.4820	3
TFAP2D	0.9555	-0.2573	1
TFCP2	0.9709	0.0770	1
TFE3	0.9927	-0.0162	1

Table S9: Non-significant synaptic proteins of cerebellar vermis in children with idiopathic autism (FDR-adjusted p > 0.05) *(continued)*

Gene name	FDR-adjusted p-value	log2(fold-change)	Number of peptides
TFG	0.9555	0.0684	2
TGM1	0.7621	0.2369	6
TGM3	0.9819	-0.0185	6
TGM5	0.9802	0.0518	1
THNSL1	0.9283	0.1175	2
THOC3	0.8783	-0.1642	1
THUMPD1	0.9482	-0.0504	3
THUMPD3	0.8400	-0.3845	2
THY1	0.5078	-0.3088	2
THYN1	0.9708	-0.0341	2
TIAM1	0.9958	-0.0089	1
TIMM13	0.9708	0.0361	2
TIMM44	0.7938	0.3058	2
TIMM8A	0.9211	0.2491	1
TIMM9	0.8993	-0.2307	1
TIPRL	0.5809	0.5851	1
TJP1	0.9911	-0.0098	2
TJP2	0.6953	0.2166	6
TKT	0.1377	0.2287	16
TLN1	0.7653	0.1021	12
TLN2	0.9217	-0.0383	17
TMA7	0.9555	0.1251	1
TMED4	0.9053	-0.2155	1
TMED8	0.7991	0.1991	1
TMEM109	0.4138	-0.6544	1
TMEM135	0.7000	-0.2926	1
TMEM163	0.7930	-0.3381	1
TMEM192	0.9217	-0.1574	1
TMEM33	0.6795	-0.3205	1
TMEM94	0.9699	0.0638	1
TMOD1	0.9832	-0.0265	1
TMOD2	0.9885	0.0088	6
TMOD3	0.7672	-0.1519	2
TMX2	0.2130	-0.3660	3
TMX3	0.8898	-0.1014	1
TNKS1BP1	0.1664	-0.5247	1
TNPO1	0.6596	-0.2175	6
TNPO2	0.9610	0.0501	1
TNR	0.6423	-0.1491	10
TNS3	0.8067	-0.1904	3
TOLLIP	0.8513	-0.1360	2
TOM1L2	0.9347	0.0827	3
TOMM40	0.9353	-0.0835	1
TOMM70	0.9652	-0.0307	5

Table S9: Non-significant synaptic proteins of cerebellar vermis in children with idiopathic autism (FDR-adjusted p > 0.05) *(continued)*

Gene name	FDR-adjusted p-value	log2(fold-change)	Number of peptides
TOP1	0.8207	0.3808	1
TOR1AIP1	0.8345	0.1858	1
TP53I11	0.7938	-0.1975	1
TP53RK	0.9482	-0.0477	2
TPD52	0.7834	-0.2420	1
TPD52L2	0.9484	0.0690	1
TPM1	0.3197	-0.2154	6
TPM3	0.9341	-0.1066	2
TPM4	0.8936	-0.0720	7
TPP2	0.1760	-0.5661	2
TPPP	0.9838	-0.0208	2
TPPP3	0.9847	-0.0138	5
TPR	0.2047	-0.4936	3
TPRG1L	0.8781	0.1655	2
TRA2B	0.9480	-0.2117	1
TRAF3	0.9709	-0.0322	2
TRAP1	0.9652	0.0558	4
TRAPPC10	0.9819	0.0612	1
TRAPPC11	0.9966	-0.0030	1
TRAPPC12	0.9404	-0.1025	1
TRAPPC13	0.9555	-0.1036	1
TRAPPC4	0.5089	-0.2149	2
TRAPPC6B	0.6953	-0.2910	1
TRAPPC9	0.9784	0.0258	2
TRIM2	0.5543	-0.2033	5
TRIM25	0.9966	-0.0088	2
TRIM28	0.5543	-0.4040	2
TRIM3	0.9482	-0.0687	2
TRIM32	0.8989	0.1347	2
TRIM67	0.6540	-0.4773	1
TRIM72	0.9405	-0.1209	1
TRIM9	0.7350	-0.2729	1
TRIO	0.8319	-0.1065	4
TRIR	0.3814	-0.6921	1
TRMT10C	0.9018	-0.1405	2
TRMU	0.9927	0.0146	1
TRNT1	0.7643	0.1207	1
TRPC3	0.3956	0.6401	1
TRPM3	0.9916	0.0187	1
TRPS1	0.9555	0.1167	1
TSC1	0.9784	0.0420	2
TSC2	0.9347	-0.0988	1
TSC22D4	0.6163	-0.2746	2
TSFM	0.9484	-0.0705	1

Table S9: Non-significant synaptic proteins of cerebellar vermis in children with idiopathic autism (FDR-adjusted p > 0.05) *(continued)*

Gene name	FDR-adjusted p-value	log2(fold-change)	Number of peptides
TSNAX	0.9376	-0.0697	1
TSTA3	0.7122	0.3476	1
TTC1	0.8339	0.2901	1
TTC37	0.8926	-0.1725	2
TTC7B	0.4154	-0.4978	1
TTC9	0.9927	-0.0164	1
TTC9B	0.9376	0.1676	1
TTI2	0.9484	-0.0729	1
TUBA1A	0.5635	-0.2352	16
TUBA4A	0.6601	-0.3098	5
TUBA8	0.6795	-0.2046	11
TUBAL3	0.7354	-0.2232	6
TUBB	0.9736	-0.0539	5
TUBB2A	0.4238	-0.2060	30
TUBB2B	0.9555	-0.1533	1
TUBB3	0.6364	-0.1970	19
TUBB4A	0.7834	-0.1885	13
TUBB4B	0.9999	0.0000	2
TUBB6	0.9468	-0.1046	2
TUBB8	0.9211	-0.3042	3
TUBGCP2	0.9833	-0.0362	1
TUFM	0.9784	-0.0172	15
TUSC2	0.4548	-0.2283	1
TWF1	0.9916	-0.0117	2
TXLNG	0.8051	-0.2502	1
TXN	0.9217	0.1937	2
TXNDC12	0.8144	0.4155	1
TXNDC17	0.7155	0.3661	2
TXNDC5	0.7834	-0.3899	1
TXNDC9	0.4176	-0.5420	1
TXNL1	0.9927	0.0096	2
TXNRD1	0.8447	0.1629	5
TXNRD2	0.9838	0.0273	1
U2AF1	0.9366	-0.2856	1
U2AF2	0.6682	-0.1973	1
U2SURP	0.8841	0.0987	1
UBA1	0.8848	0.1429	6
UBA2	0.9864	0.0168	2
UBA3	0.7970	0.4012	1
UBA52	0.4057	0.3006	3
UBA6	0.9819	0.0224	3
UBAP2L	0.6953	-0.3120	3
UBE2F	0.9934	0.0096	1
UBE2K	0.5543	0.5215	1

Table S9: Non-significant synaptic proteins of cerebellar vermis in children with idiopathic autism (FDR-adjusted p > 0.05) *(continued)*

Gene name	FDR-adjusted p-value	log2(fold-change)	Number of peptides
UBE2M	0.4874	0.3269	1
UBE2N	0.5097	0.3048	3
UBE2O	0.6565	0.3186	1
UBE2V1	0.9353	-0.1793	1
UBE2V2	0.9947	-0.0080	1
UBE3A	0.9916	0.0141	1
UBE4A	0.9480	-0.0959	3
UBL4A	0.8783	-0.1827	1
UBL5	0.9838	-0.0273	1
UBR4	0.6902	-0.1547	6
UBXN6	0.9217	-0.1500	1
UCHL1	0.4702	0.3964	4
UFC1	0.7909	0.2515	2
UFD1	0.9484	-0.0878	1
UFL1	0.9217	-0.1506	3
UFM1	0.9217	0.2492	1
UGGT1	0.8270	-0.0931	6
UGP2	0.6921	-0.1285	10
UHRF1BP1	0.8871	0.2090	1
UHRF1BP1L	0.7106	-0.4593	1
ULK3	0.7106	-0.2674	2
UMPS	0.7417	-0.2094	2
UNC13A	0.9482	-0.0641	4
UNC13C	0.9053	-0.0966	3
UNC45A	0.9610	-0.0778	2
UNC5C	0.8380	0.2044	1
UPF1	0.7822	-0.2049	4
UQCC1	0.8270	-0.3041	1
UQCRB	0.9971	-0.0025	3
UQCRC1	0.9482	0.1258	1
UQCRC2	0.9966	-0.0018	8
UQCRFS1P1	0.8783	-0.1514	3
URI1	0.9784	-0.0464	1
USE1	0.9555	-0.0752	1
USO1	0.9999	0.0004	2
USP10	0.9784	-0.0359	1
USP14	0.9217	-0.2767	1
USP15	0.9819	0.0169	2
USP24	0.7621	-0.1781	1
USP28	0.9652	0.0805	1
USP47	0.8750	-0.1049	5
USP5	0.6423	-0.4332	4
USP7	0.9847	-0.0353	1
USP9X	0.2326	-0.2237	3

Table S9: Non-significant synaptic proteins of cerebellar vermis in children with idiopathic autism (FDR-adjusted p > 0.05) *(continued)*

Gene name	FDR-adjusted p-value	log2(fold-change)	Number of peptides
UTP20	0.4874	-0.3489	1
UTRN	0.9217	0.1273	1
V9GYY5	0.9555	-0.0501	1
VAC14	0.6163	-0.4352	2
VAMP7	0.9376	0.1720	1
VAPA	0.9555	-0.1146	1
VAPB	0.9885	-0.0083	4
VARS	0.7461	-0.5218	1
VARS2	0.9555	0.1084	1
VAT1	0.3698	0.4470	2
VAT1L	0.9366	-0.0439	8
VBP1	0.9283	-0.0585	2
VCAN	0.5543	-0.1818	10
VCP	0.6902	-0.1413	6
VCPIP1	0.9484	-0.0799	1
VDAC1	0.1057	-0.5081	10
VDAC2	0.0605	-0.4204	11
VDAC3	0.3698	-0.4144	6
VIM	0.9211	0.1588	4
VPS13A	0.7417	-0.2602	2
VPS13C	0.9217	0.0634	3
VPS13D	0.7643	0.3107	1
VPS25	0.9784	0.0326	1
VPS29	0.6540	-0.3131	2
VPS33A	0.9217	-0.0955	3
VPS35	0.9347	-0.0873	6
VPS36	0.9891	0.0136	1
VPS39	0.6246	-0.6997	1
VPS41	0.9911	0.0228	1
VPS45	0.7179	-0.1913	4
VPS4B	0.7621	-0.6488	1
VPS50	0.2417	-0.4261	2
VPS51	0.3965	-0.6405	1
VPS53	0.7000	-0.2964	2
VPS8	0.9217	-0.2133	1
VSNL1	0.2642	-0.2528	5
VTA1	0.7436	-0.1591	2
VWA5A	0.9217	0.1430	1
VWA8	0.6423	-0.2117	2
WARS2	0.9353	-0.1501	1
WASF3	0.3665	-0.2607	2
WASH2P	0.9211	-0.1459	1
WASHC3	0.9217	0.1994	1
WASHC4	0.8871	-0.1020	2

Table S9: Non-significant synaptic proteins of cerebellar vermis in children with idiopathic autism (FDR-adjusted p > 0.05) *(continued)*

Gene name	FDR-adjusted p-value	log2(fold-change)	Number of peptides
WASHC5	0.6706	-0.1892	3
WASL	0.9819	0.0293	1
WDFY3	0.9211	-0.1877	2
WDR1	0.9217	-0.0740	6
WDR11	0.9784	0.0534	2
WDR13	0.7436	-0.2358	2
WDR17	0.2224	-0.4375	1
WDR37	0.9610	-0.0604	3
WDR4	0.9468	-0.1269	1
WDR45	0.9819	0.0134	3
WDR45B	0.7417	0.5063	1
WDR47	0.8894	-0.2868	2
WDR7	0.5078	-0.2963	4
WDR91	0.9784	-0.0496	1
WDR92	0.5023	-0.5765	1
WDTC1	0.2417	-0.3393	2
WIPF2	0.9708	-0.0336	3
WIPI2	0.9832	-0.0587	2
WNK2	0.8967	-0.1824	1
XKR7	0.9709	0.0478	1
XPNPEP1	0.7938	0.2426	2
XPO1	0.6423	-0.2770	6
XPO5	0.7938	0.2206	1
XPO7	0.7473	0.1885	2
XRCC4	0.8400	0.1652	1
XRCC6	0.0755	-0.5872	7
YARS	0.6858	-0.1355	8
YARS2	0.9555	-0.0707	2
YES1	0.8989	0.1685	1
YKT6	0.9819	0.0206	5
YWHAB	0.9482	0.0613	8
YWHAE	0.2995	0.2058	9
YWHAG	0.7643	-0.2200	7
YWHAH	0.8942	-0.0982	7
YWHAQ	0.9555	0.1374	1
YWHAZ	0.9482	0.0521	12
ZADH2	0.9819	-0.0318	1
ZC2HC1A	0.9891	-0.0093	5
ZC3H15	0.7822	-0.1968	1
ZC3HAV1	0.9555	-0.0923	1
ZG16B	0.9646	-0.0557	2
ZMIZ2	0.7999	-0.4011	1
ZNF516	0.9610	0.1678	1
ZPR1	0.7834	0.2499	1

Table S9: Non-significant synaptic proteins of cerebellar vermis in children with idiopathic autism (FDR-adjusted p > 0.05) *(continued)*

Gene name	FDR-adjusted p-value	log2(fold-change)	Number of peptides
ZRANB2	0.4137	-0.2991	2
ZW10	0.9484	-0.1355	1
ZZEF1	0.2148	-0.5751	1

Table S10: Non-significant synaptic proteins of cerebellar vermis in adults with idiopathic autism (FDR-adjusted p > 0.05)

Gene name	FDR-adjusted p-value	log2(fold-change)	Number of peptides
A0A2R8YDQ0	0.7098	0.4017	2
A0A3B3ISG8	0.1303	-0.5367	4
A0A3B3ISV5	0.9085	0.0517	5
A0A3B3ITX4	0.9926	-0.0048	3
A1BG	0.9669	0.0424	2
A2M	0.8273	0.1447	5
A2ML1	0.8690	0.1189	2
AAK1	0.5151	-0.3368	3
AARS	0.9630	-0.0234	9
AARSD1	0.8690	-0.1258	1
ABAT	0.7985	0.5603	1
ABCB7	0.9106	-0.0807	2
ABCC4	0.8797	-0.3729	1
ABCD2	0.8536	0.2524	1
ABCD3	0.8033	-0.2469	1
ABCF1	0.9957	0.0024	1
ABCF3	0.5691	-0.2471	2
ABHD10	0.8233	0.2206	2
ABHD11	0.7796	-0.1479	3
ABI1	0.9870	-0.0153	1
ABI2	0.8806	-0.0769	3
ABL2	0.7789	0.2094	1
ABLIM1	0.5005	-0.2010	2
ABR	0.7565	-0.1190	5
ACAA1	0.4187	-0.5085	2
ACAA2	0.6714	0.2173	3
ACAD8	0.8806	-0.2300	1
ACAD9	0.4104	-0.4056	2
ACADSB	0.4917	-0.7612	1
ACADVL	0.9270	-0.0345	13
ACAP2	0.5551	0.3476	2
ACAT1	0.8903	0.0550	11
ACIN1	0.8680	0.1011	2
ACLY	0.2468	-0.2112	14
ACO1	0.6867	0.2873	1
ACO2	0.9146	-0.0615	5
ACOT1	0.7949	-0.1664	1
ACOT13	0.2527	-0.4591	4
ACOT7	0.8273	-0.1485	3
ACOT9	0.8796	-0.0779	2
ACOX1	0.7590	0.1973	2
ACSBG1	0.4519	-0.5588	1
ACTB	0.7550	-0.1360	4
ACTBL2	0.8161	0.1479	2

Table S10: Non-significant synaptic proteins of cerebellar vermis in adults with idiopathic autism (FDR-adjusted p > 0.05) *(continued)*

Gene name	FDR-adjusted p-value	log2(fold-change)	Number of peptides
ACTG1	0.4837	0.5975	1
ACTG2	0.7058	-0.0833	17
ACTN4	0.1828	0.4680	3
ACTR10	0.5151	-0.6686	1
ACTR1A	0.4131	-0.2820	4
ACTR2	0.8033	-0.1188	4
ACTR3	0.6414	-0.1871	4
ACTR3B	0.8352	-0.1286	2
ADAM10	0.4100	0.8033	1
ADAM11	0.9500	-0.0711	1
ADAM22	0.7949	0.1443	3
ADAM23	0.8033	0.3728	1
ADAP1	0.5857	-0.4236	2
ADAR	0.9484	0.0771	3
ADARB1	0.9423	-0.0522	3
ADD1	0.4187	-0.2707	5
ADD2	0.0962	-0.4026	7
ADD3	0.1828	-0.3537	5
ADGRG1	0.6578	-0.2582	1
ADGRL3	0.1256	-1.0246	1
ADNP2	0.8690	-0.2976	1
ADO	0.6310	-0.5036	1
ADPRHL2	0.7742	-0.1823	1
AFDN	0.4735	-0.2323	3
AGAP2	0.8140	-0.1365	3
AGAP3	0.3688	-0.3126	4
AGFG1	0.5809	-0.5694	1
AGK	0.7796	0.3102	1
AGL	0.5144	0.3723	3
AGO1	0.9749	0.0425	1
AGPAT3	0.7556	-0.1026	2
AGPAT4	0.8905	0.1416	1
AGPS	0.7550	0.2027	1
AHCTF1	0.8220	-0.1282	2
AHCY	0.4180	0.3158	6
AHCYL1	0.8396	0.0701	10
AHNAK	0.0830	-0.2715	21
AHNAK2	0.8161	-0.2991	1
AHSA1	0.7532	-0.1862	2
AIFM1	0.6577	-0.4865	1
AIFM3	0.7550	0.3189	1
AIMP1	0.9630	-0.0420	1
AIMP2	0.8986	0.1401	3
AIP	0.9692	0.0491	1

Table S10: Non-significant synaptic proteins of cerebellar vermis in adults with idiopathic autism (FDR-adjusted p > 0.05) *(continued)*

Gene name	FDR-adjusted p-value	log2(fold-change)	Number of peptides
AK1	0.8768	0.0769	5
AK3	0.8986	0.0826	2
AK4	0.5780	-0.4048	3
AK5	0.5343	0.2937	1
AKAP12	0.9330	-0.0370	4
AKAP6	0.4285	0.5293	1
AKR1A1	0.8645	0.0820	4
AKR1B1	0.7565	0.2274	3
AKR1C1	0.6587	0.3285	1
AKT1	0.8726	-0.0863	2
ALAD	0.7098	0.3047	2
ALDH1A1	0.1303	0.4176	9
ALDH1L1	0.6079	0.2109	5
ALDH2	0.1019	0.4349	7
ALDH4A1	0.6192	0.2554	3
ALDH5A1	0.8797	0.1120	3
ALDH6A1	0.4693	-0.1775	17
ALDH7A1	0.2929	0.2303	11
ALDH8A1	0.5011	0.5288	1
ALDH9A1	0.9169	0.0422	7
ALDOA	0.7212	0.1433	12
ALDOC	0.5151	0.1857	17
ALS2	0.7173	-0.3091	3
AMER2	0.9761	0.0235	5
AMOT	0.6735	-0.1641	1
AMPH	0.7935	-0.0904	11
AMT	0.7611	0.2551	1
ANK1	0.6486	-0.1602	11
ANK3	0.4187	-0.1459	25
ANKFY1	0.8960	0.0654	4
ANKMY2	0.9946	0.0112	1
ANKRD13D	0.4210	0.3652	1
ANKRD28	0.8680	0.1222	2
ANKRD29	0.7173	0.2640	3
ANKRD44	0.5497	-0.2519	2
ANKS1B	0.8352	0.1814	2
ANLN	0.9484	-0.0377	3
ANP32A	0.8986	0.0710	5
ANP32B	0.8690	-0.1816	1
ANXA1	0.8153	0.2485	1
ANXA11	0.7053	0.2081	2
ANXA5	0.1728	0.3666	3
ANXA6	0.7789	0.0983	9
ANXA7	0.2593	0.4179	1

Table S10: Non-significant synaptic proteins of cerebellar vermis in adults with idiopathic autism (FDR-adjusted p > 0.05) *(continued)*

Gene name	FDR-adjusted p-value	log2(fold-change)	Number of peptides
AP1B1	0.8680	-0.0877	6
AP1G1	0.9843	-0.0092	4
AP1M1	0.7702	-0.1745	3
AP1S2	0.4426	0.5046	1
AP2A1	0.7161	0.0784	17
AP2A2	0.8960	-0.0618	3
AP2B1	0.7789	0.0710	22
AP2M1	0.8651	-0.0690	6
AP3B1	0.8054	-0.1011	3
AP3D1	0.4889	-0.1833	5
AP3M1	0.6038	-0.6870	1
AP3M2	0.3727	-0.3106	6
AP3S1	0.7789	0.3479	1
AP4S1	0.7984	-0.0796	1
AP5M1	0.8171	0.2569	1
AP5Z1	0.8020	0.2417	1
APBB1	0.3935	-0.6356	1
APC	0.8322	0.2510	1
APEX1	0.4269	-0.7727	1
API5	0.4352	-0.2234	7
APMAP	0.9578	0.0754	1
APOA1	0.5038	-0.3728	4
APOA2	0.9809	-0.0323	1
APOB	0.6542	0.2930	4
APOL2	0.7173	-0.2040	1
APPL2	0.9484	-0.0796	1
AQP4	0.7780	-0.2411	2
ARCN1	0.7565	-0.1375	5
ARF4	0.4543	0.2465	3
ARF5	0.3738	0.4125	2
ARF6	0.7590	-0.1591	3
ARFGAP1	0.6453	0.2913	2
ARFGEF2	0.4664	-0.3562	1
ARFGEF3	0.9259	-0.0508	4
ARHGAP1	0.9214	-0.1350	2
ARHGAP12	0.7556	0.3900	2
ARHGAP21	0.1256	-0.3099	4
ARHGAP26	0.0593	-0.2914	9
ARHGAP35	0.7565	0.1193	2
ARHGAP39	0.6714	-0.2829	1
ARHGAP44	0.8536	0.1981	2
ARHGAP5	0.1248	-0.4245	3
ARHGEF10L	0.8536	0.1087	1
ARHGEF11	0.2237	-0.3957	2

Table S10: Non-significant synaptic proteins of cerebellar vermis in adults with idiopathic autism (FDR-adjusted p > 0.05) *(continued)*

Gene name	FDR-adjusted p-value	log2(fold-change)	Number of peptides
ARHGEF12	0.7789	0.2057	2
ARHGEF2	0.9808	-0.0324	1
ARHGEF3	0.7565	0.2983	1
ARHGEF33	0.1303	-0.5664	2
ARHGEF37	0.9260	0.0451	1
ARHGEF7	0.1186	-0.5604	4
ARID1A	0.9596	-0.0352	1
ARID1B	0.9156	-0.0976	1
ARID2	0.7628	-0.2809	1
ARID3B	0.3210	-1.1205	1
ARL1	0.7949	-0.2425	1
ARL2	0.6272	-0.2418	2
ARL3	0.7550	0.1965	4
ARL6IP5	0.3935	-0.4825	2
ARL8B	0.7949	0.2757	1
ARMC10	0.7413	-0.2122	2
ARMC6	0.8542	0.0856	2
ARMC8	0.1806	-0.4334	2
ARNT	0.9669	0.0461	1
ARPC1A	0.7976	-0.0884	4
ARPC1B	0.7792	0.3178	1
ARPC2	0.8727	-0.0664	5
ARPC3	0.5593	0.5368	1
ARPC4	0.9106	-0.0460	5
ARPC5	0.9270	0.0570	3
ARPC5L	0.8493	-0.0703	5
ARRB1	0.8679	-0.2599	1
ARVCF	0.7789	-0.2747	2
ASAH1	0.3912	0.2999	1
ASAP2	0.9944	-0.0052	1
ASNA1	0.8504	-0.1022	2
ASNS	0.9260	-0.0457	3
ASPH	0.9761	0.0197	2
ASPM	0.8986	-0.1127	1
ASRGL1	0.5104	0.4901	1
ASS1	0.9801	-0.0154	2
ATAD1	0.5962	-0.2884	1
ATAD3A	0.7556	-0.2210	2
ATAT1	0.8679	0.3950	1
ATG16L1	0.9053	-0.1083	1
ATG2B	0.9509	-0.0892	1
ATG3	0.9178	-0.1124	1
ATG4C	0.3310	-0.6583	1
ATIC	0.2768	-0.3282	5

Table S10: Non-significant synaptic proteins of cerebellar vermis in adults with idiopathic autism (FDR-adjusted p > 0.05) *(continued)*

Gene name	FDR-adjusted p-value	log2(fold-change)	Number of peptides
ATP13A1	0.6578	-0.3010	2
ATP1A1	0.9946	0.0032	6
ATP1A2	0.6714	0.1980	6
ATP1A3	0.9870	0.0040	24
ATP1B1	0.7611	-0.2819	2
ATP2A2	0.7173	0.1072	9
ATP2B1	0.9166	0.0383	7
ATP2B2	0.7897	0.1554	3
ATP2B4	0.8493	-0.1211	2
ATP5F1A	0.2194	0.1963	18
ATP5F1B	0.9761	-0.0128	13
ATP5F1C	0.7797	0.0975	10
ATP5F1D	0.9509	-0.0538	3
ATP5F1E	0.7749	0.2331	2
ATP5IF1	0.8690	0.1716	3
ATP5MC1	0.7775	0.2935	1
ATP5ME	0.7912	-0.1330	3
ATP5MPL	0.2593	-1.0230	1
ATP5PB	0.8504	0.1863	3
ATP5PD	0.4210	0.4966	3
ATP5PF	0.5420	0.4507	2
ATP5PO	0.5470	0.1599	9
ATP6AP1	0.9891	-0.0098	3
ATP6AP2	0.8690	-0.1645	1
ATP6V0A1	0.8680	0.1100	3
ATP6V0D1	0.8433	-0.0942	6
ATP6V1A	0.2252	0.2729	8
ATP6V1B2	0.9086	-0.0334	12
ATP6V1C1	0.6828	-0.1504	8
ATP6V1D	0.9581	-0.0383	2
ATP6V1E1	0.4193	0.2290	4
ATP6V1F	0.9259	0.0957	1
ATP6V1H	0.7400	-0.2061	4
ATP8A1	0.7265	-0.2613	1
ATP9A	0.4889	-0.6907	1
ATXN10	0.8504	0.0649	2
ATXN2	0.9164	0.1109	1
ATXN2L	0.5497	-0.4078	2
AUH	0.2108	-0.2948	10
AZGP1	0.5005	0.9047	1
BABAM1	0.4567	0.5126	1
BAG3	0.9826	0.0233	1
BAG6	0.6714	0.2150	2
BAIAP2	0.1828	-0.3033	6

Table S10: Non-significant synaptic proteins of cerebellar vermis in adults with idiopathic autism (FDR-adjusted p > 0.05) *(continued)*

Gene name	FDR-adjusted p-value	log2(fold-change)	Number of peptides
BASP1	0.5630	0.2413	8
BAZ1B	0.8783	-0.2548	1
BBOX1	0.9141	-0.0778	1
BCAN	0.5218	0.2285	4
BCAP29	0.8161	-0.2017	1
BCAP31	0.8054	-0.1687	3
BCAS1	0.8680	-0.0612	11
BCAS3	0.6981	-0.1851	2
BCAT1	0.8017	-0.2224	1
BCLAF1	0.7532	0.2766	1
BCR	0.6578	-0.3084	1
BDH2	0.3573	0.5918	2
BIN1	0.7789	-0.1127	6
BLMH	0.1828	0.5472	3
BLVRA	0.5470	-0.6177	1
BOLA2	0.8903	0.1053	1
BORCS5	0.8273	-0.2912	1
BPIFB1	0.8368	0.2618	1
BPNT1	0.8690	0.2141	1
BRAF	0.2724	-0.4553	3
BRD3	0.5518	-0.5246	1
BRK1	0.9764	0.0168	3
BROX	0.9843	0.0134	2
BRSK1	0.6059	0.1900	2
BRSK2	0.8903	-0.2163	1
BSG	0.9132	0.1179	1
BSN	0.2252	-0.3043	14
BSPRY	0.9484	0.0525	1
BTBD17	0.6867	-0.2330	2
BUB3	0.2888	-0.9373	1
BZW1	0.9484	0.0388	2
C12orf4	0.9926	0.0126	1
C15orf38	0.9831	0.0199	1
C17orf49	0.7603	-0.3702	1
C17orf75	0.7118	-0.4573	1
C1orf198	0.7118	0.4272	1
C2CD5	0.6828	-0.2180	3
C2orf68	0.8986	-0.1086	1
C3	0.2014	0.3193	8
C4A	0.8628	0.0925	4
C9orf64	0.7382	0.3809	1
CA1	0.8690	-0.1372	2
CA10	0.4630	-0.6192	1
CA2	0.9893	-0.0117	1

Table S10: Non-significant synaptic proteins of cerebellar vermis in adults with idiopathic autism (FDR-adjusted p > 0.05) *(continued)*

Gene name	FDR-adjusted p-value	log2(fold-change)	Number of peptides
CA4	0.9661	-0.0175	6
CA8	0.6984	-0.2559	1
CAB39	0.7789	-0.1553	2
CAB39L	0.9619	-0.0519	1
CACNA1A	0.6578	-0.2126	2
CACNA2D1	0.9109	-0.0719	2
CACNB2	0.9322	0.0258	5
CACNB3	0.6890	-0.2350	1
CACNB4	0.9457	-0.0450	2
CACNG2	0.3278	-0.4495	2
CACYBP	0.5788	-0.3781	2
CAD	0.8433	-0.1285	3
CADPS	0.5562	-0.1478	8
CADPS2	0.4977	-0.1594	14
CALB1	0.4597	0.4309	4
CALB2	0.7780	0.1454	5
CALCOCO1	0.8330	0.1148	2
CALML5	0.1828	0.7602	3
CALR	0.9857	0.0068	4
CAMK1	0.5005	-0.2174	4
CAMK2A	0.6542	-0.2171	7
CAMK2B	0.5908	-0.2395	4
CAMK2D	0.8574	-0.0725	11
CAMK2G	0.6578	-0.1745	5
CAMK4	0.8106	-0.0873	9
CAMKK1	0.8033	0.1500	1
CAMKK2	0.6270	-0.2394	4
CAMSAP2	0.9893	0.0122	1
CAND1	0.7229	0.0703	17
CANX	0.7789	0.2402	1
CAP1	0.9102	0.0522	3
CAP2	0.6714	-0.2294	4
CAPN1	0.9669	0.0186	2
CAPN5	0.8950	-0.0999	1
CAPRIN1	0.5648	-0.2407	8
CAPZA1	0.8683	-0.0812	2
CAPZA2	0.8986	0.0469	4
CAPZB	0.6182	0.1400	7
CARD16	0.7955	0.2959	1
CARMIL1	0.3221	-0.3911	4
CARS	0.9761	0.0338	1
CASK	0.8903	-0.0588	5
CASKIN1	0.4193	-0.4116	3
CASP14	0.1657	0.7509	3

Table S10: Non-significant synaptic proteins of cerebellar vermis in adults with idiopathic autism (FDR-adjusted p > 0.05) *(continued)*

Gene name	FDR-adjusted p-value	log2(fold-change)	Number of peptides
CASQ2	0.7159	0.3765	1
CAST	0.7243	0.3467	2
CAT	0.1303	0.6734	4
CAVIN1	0.8690	0.5133	1
CAVIN2	0.8796	-0.1485	2
CBLN1	0.8536	-0.0715	2
CBR1	0.4100	0.3941	3
CBX5	0.5562	-0.7888	1
CBX6	0.8903	0.1486	1
CCAR1	0.7481	0.1806	2
CCAR2	0.6372	-0.1824	6
CCDC124	0.8905	-0.2111	1
CCDC127	0.6587	-0.3499	1
CCDC136	0.5857	-0.5954	1
CCDC183	0.8680	-0.1545	1
CCDC51	0.8783	-0.2061	1
CCDC6	0.9330	0.0777	1
CCDC65	0.7797	0.4902	1
CCDC88A	0.9386	0.1258	1
CCDC91	0.7036	-0.2525	1
CCDC93	0.9535	-0.0343	3
CCNB3	0.5857	-0.4018	1
CCT2	0.2237	-0.2025	16
CCT3	0.8575	-0.1076	8
CCT4	0.1303	-0.2692	13
CCT5	0.9826	-0.0060	14
CCT6A	0.6264	-0.1095	16
CCT7	0.1947	-0.2016	18
CCT8	0.5691	-0.1088	22
CD163L1	0.8033	-0.3449	1
CD2AP	0.9619	0.0446	1
CD47	0.9857	-0.0074	7
CD59	0.8903	0.0786	2
CDC42	0.8606	0.1505	2
CDC42BPB	0.6867	-0.1238	5
CDC42EP1	0.5718	-0.5307	1
CDC42EP4	0.4884	-0.3203	5
CDC5L	0.6375	-0.2680	1
CDH10	0.4352	-0.7594	1
CDH15	0.2911	0.4676	1
CDH2	0.8389	0.1701	1
CDK11A	0.8905	0.1603	1
CDK16	0.8396	-0.1624	1
CDK5	0.5637	-0.2399	4

Table S10: Non-significant synaptic proteins of cerebellar vermis in adults with idiopathic autism (FDR-adjusted p > 0.05) *(continued)*

Gene name	FDR-adjusted p-value	log2(fold-change)	Number of peptides
CDK5RAP3	0.4352	-0.3198	2
CDKN2AIP	0.6818	-0.2791	1
CDSN	0.4131	0.4140	3
CELF1	0.6079	-0.3691	3
CELF2	0.7785	-0.2818	3
CEMIP2	0.9484	-0.0886	1
CEND1	0.9484	-0.0913	2
CENPE	0.7919	0.1384	1
CENPP	0.8548	-0.2672	1
CENPV	0.6079	0.4726	1
CEP170	0.8054	-0.1393	3
CEP170B	0.8826	-0.2032	1
CETN2	0.9484	-0.0870	1
CFL1	0.7306	-0.2283	3
CFL2	0.9893	-0.0076	3
CGGBP1	0.8903	0.1926	1
CHCHD3	0.8963	0.0965	3
CHCHD6	0.7159	-0.6054	1
CHDH	0.4257	-0.5376	2
CHERP	0.7417	-0.3040	1
CHID1	0.7161	-0.3611	1
CHMP1A	0.8680	0.0573	5
CHMP1B	0.9484	0.0432	1
CHMP2A	0.8903	0.1080	2
CHMP2B	0.9832	0.0093	6
CHMP3	0.8796	0.0648	3
CHMP4B	0.9929	0.0043	4
CHMP5	0.8963	0.2422	2
CHP1	0.8690	-0.1363	1
CIAPIN1	0.5637	0.4522	1
CIRBP	0.7565	-0.2441	1
CISD2	0.9312	-0.0496	2
CKAP4	0.8690	-0.0692	6
CKAP5	0.2888	-0.2330	11
CKB	0.2768	-0.2568	13
CKMT1A	0.4837	-0.2619	7
CLASP1	0.5809	-0.3237	2
CLASP2	0.7550	-0.1423	6
CLCN4	0.7550	-0.2461	1
CLDN11	0.7317	-0.1711	3
CLDND1	0.8796	0.1971	1
CLIC1	0.9762	-0.0275	1
CLIP1	0.4628	-0.1957	9
CLIP2	0.2800	-0.2782	9

Table S10: Non-significant synaptic proteins of cerebellar vermis in adults with idiopathic autism (FDR-adjusted p > 0.05) *(continued)*

Gene name	FDR-adjusted p-value	log2(fold-change)	Number of peptides
CLSTN1	0.9946	-0.0026	4
CLTA	0.6391	0.2805	3
CLTB	0.8375	0.1359	6
CLTC	0.6005	0.1565	19
CLU	0.7532	0.0933	5
CLUH	0.8905	0.0805	1
CLVS2	0.8536	-0.1948	1
CLYBL	0.9462	0.1121	1
CMAS	0.6542	-0.2749	3
CMC1	0.8796	-0.2149	1
CMPK1	0.6449	-0.3385	1
CNDP2	0.4226	0.3292	6
CNN1	0.2994	-0.5613	2
CNN3	0.9946	-0.0044	3
CNNM4	0.5809	-0.4440	1
CNOT1	0.9484	-0.0318	3
CNOT11	0.8433	-0.1535	1
CNOT2	0.9746	0.0499	1
CNOT3	0.8680	-0.2802	1
CNOT9	0.7118	-0.4110	1
CNP	0.8679	0.0612	20
CNRIP1	0.8903	0.1860	1
CNTN1	0.0844	-0.1782	15
CNTN2	0.1346	-0.3992	3
CNTNAP1	0.9093	-0.0375	4
CNTNAP2	0.4149	-0.4280	2
COG3	0.4711	0.3994	1
COG5	0.9791	-0.0249	1
COL1A1	0.6181	-0.3230	4
COL1A2	0.9535	-0.0732	2
COL23A1	0.8118	0.1923	1
COL4A2	0.6828	-0.3557	1
COL6A1	0.8690	-0.1917	1
COL6A2	0.5679	-0.4668	1
COL6A3	0.9214	0.0404	6
COLGALT1	0.9330	0.0466	1
COMMD5	0.7912	-0.1278	4
COMMD7	0.9327	-0.1120	1
COMT	0.2619	-0.3914	2
COPA	0.9713	-0.0159	8
COPB1	0.8690	0.0806	3
COPB2	0.7047	0.1844	4
COPG1	0.8330	0.0994	4
COPG2	0.7912	-0.2566	2

Table S10: Non-significant synaptic proteins of cerebellar vermis in adults with idiopathic autism (FDR-adjusted p > 0.05) *(continued)*

Gene name	FDR-adjusted p-value	log2(fold-change)	Number of peptides
COPS2	0.8963	-0.1341	1
COPS3	0.7173	0.3803	1
COPS4	0.9893	-0.0118	1
COPS5	0.9857	0.0091	4
COPS7A	0.8680	-0.1182	1
COQ10B	0.5470	-0.4103	1
COQ8A	0.9900	-0.0226	1
CORO1A	0.2758	-0.3447	7
CORO1B	0.1303	-0.4768	3
CORO1C	0.5515	-0.3428	3
CORO2A	0.6901	-0.2611	3
CORO2B	0.7176	0.1661	5
CORO7	0.4100	-0.2932	4
COTL1	0.7789	-0.1409	3
COX4I1	0.9722	0.0472	2
COX5A	0.6714	0.2811	3
COX5B	0.8375	-0.1314	4
COX6B1	0.9873	-0.0265	1
COX6C	0.9926	-0.0078	2
COX7A2	0.9843	0.0335	1
COX7A2L	0.8529	-0.1066	2
COX7C	0.9260	0.1251	1
CPA4	0.5038	0.7344	1
CPE	0.7949	0.2051	2
CPLX2	0.3563	0.3193	4
CPNE6	0.9812	0.0136	2
CPSF1	0.5691	-0.5502	2
CPSF6	0.4495	-0.4141	2
CPSF7	0.0728	-1.0010	3
CPT1A	0.7532	-0.3608	1
CPT2	0.7789	0.2792	1
CRAT	0.7550	-0.1300	8
CREB1	0.7853	-0.3149	1
CRELD1	0.9089	0.1378	1
CRIP2	0.8563	0.3163	1
CRKL	0.7118	-0.2301	2
CRMP1	0.4210	-0.2375	8
CRYAB	0.8504	0.0804	3
CRYL1	0.8273	-0.2842	1
CRYM	0.8768	-0.0933	4
CRYZ	0.9641	0.0314	6
CS	0.0501	-0.3647	12
CSDC2	0.8122	-0.1287	1
CSDE1	0.8783	-0.0779	4

Table S10: Non-significant synaptic proteins of cerebellar vermis in adults with idiopathic autism (FDR-adjusted p > 0.05) *(continued)*

Gene name	FDR-adjusted p-value	log2(fold-change)	Number of peptides
CSE1L	0.8881	-0.0817	2
CSK	0.9026	-0.0542	3
CSN1S2	0.9791	-0.0402	1
CSN2	0.3912	0.8775	1
CSNK1A1	0.8504	-0.1066	3
CSNK2B	0.9749	-0.0285	2
CSRP1	0.2192	-0.9985	1
CST6	0.4193	0.6520	2
CSTB	0.6270	0.5030	1
CSTF2	0.6828	-0.3774	1
CSTF3	0.4791	-0.5225	1
CT45A2	0.6848	-0.5411	1
CTBP1	0.9804	-0.0144	3
CTNNA2	0.1150	-0.4069	4
CTNNB1	0.7988	0.0940	7
CTPS1	0.1828	-0.4469	3
CTTN	0.0930	-0.5296	4
CUL1	0.6194	0.2657	1
CUL3	0.9214	-0.0774	1
CUL4B	0.2831	0.8169	1
CUL5	0.9900	-0.0097	1
CWF19L1	0.5551	-0.7013	1
CXXC5	0.4791	-0.5954	1
CYB5R1	0.2991	-0.3534	5
CYCS	0.8020	0.1358	5
CYFIP1	0.6901	-0.1006	11
CYFIP2	0.7134	-0.1721	2
CYP51A1	0.2252	-0.5954	1
CYTH2	0.9535	-0.0809	1
CZIB	0.8020	0.3510	1
DAAM1	0.7362	-0.1802	1
DAAM2	0.4630	-0.4214	1
DAB2IP	0.6735	0.6013	2
DAGLA	0.4100	-0.2206	7
DAO	0.8600	-0.1678	1
DAP3	0.9947	-0.0034	1
DARS	0.8494	-0.0902	3
DARS2	0.7118	0.4200	1
DAZAP1	0.4536	-0.6540	1
DBNL	0.8935	-0.1244	1
DBR1	0.9166	0.0751	1
DBT	0.9339	-0.0382	4
DCAKD	0.8780	0.2029	1
DCD	0.0909	0.8978	4

Table S10: Non-significant synaptic proteins of cerebellar vermis in adults with idiopathic autism (FDR-adjusted p > 0.05) *(continued)*

Gene name	FDR-adjusted p-value	log2(fold-change)	Number of peptides
DCLK1	0.5470	-0.1648	6
DCLK2	0.9578	-0.0329	5
DCN	0.6656	-0.7344	1
DCTN1	0.3688	-0.5841	3
DCTN2	0.5151	-0.1763	10
DCTN3	0.9713	-0.0181	5
DCTN4	0.8903	-0.0634	4
DCTN5	0.5927	-0.8125	1
DCXR	0.4646	-0.2982	4
DDAH1	0.6372	-0.2333	4
DDB1	0.8680	-0.0625	6
DDOST	0.4210	0.2708	5
DDX1	0.4630	-0.1622	13
DDX17	0.1557	-0.2824	11
DDX18	0.9630	0.0531	1
DDX19B	0.5857	-0.2739	4
DDX23	0.4421	-0.3182	3
DDX27	0.9797	-0.0222	1
DDX39A	0.6014	-0.2006	6
DDX39B	0.4837	-0.3454	2
DDX3X	0.9034	-0.0534	4
DDX3Y	0.7962	-0.3794	1
DDX42	0.2831	-0.9572	1
DDX46	0.5857	-0.1983	7
DDX5	0.6272	-0.2248	6
DDX6	0.7789	-0.1408	3
DECR1	0.3523	0.2335	14
DECR2	0.7988	-0.1187	2
DEK	0.4729	-0.2544	6
DENR	0.8903	-0.1025	2
DES	0.5210	-0.4960	4
DESI1	0.7556	-0.3652	1
DFFA	0.9811	-0.0234	1
DGKH	0.9639	-0.0993	1
DGKZ	0.9271	0.1211	2
DHRS11	0.8866	0.1380	1
DHRS13	0.5038	-0.4772	1
DHRS4	0.4628	-0.3411	2
DHRS7B	0.9122	0.1145	1
DHX15	0.9106	-0.0900	3
DHX16	0.5857	-0.2813	1
DHX29	0.2755	0.5271	1
DHX30	0.9212	-0.0784	3
DHX8	0.5282	-0.3493	1

Table S10: Non-significant synaptic proteins of cerebellar vermis in adults with idiopathic autism (FDR-adjusted p > 0.05) *(continued)*

Gene name	FDR-adjusted p-value	log2(fold-change)	Number of peptides
DHX9	0.9029	-0.1098	4
DIDO1	0.9762	0.0263	1
DIP2B	0.2180	-0.2675	11
DIP2C	0.5551	-0.2534	4
DIRAS1	0.9166	-0.0628	3
DIRAS2	0.9284	-0.0431	2
DLAT	0.6714	-0.1269	13
DLD	0.5857	0.2023	11
DLG2	0.4628	-0.4147	2
DLG3	0.5702	0.3437	1
DLG4	0.7187	0.2757	1
DLGAP1	0.4507	-0.3314	3
DLGAP3	0.3738	-0.7874	1
DLST	0.4104	0.2438	8
DMD	0.8903	0.0992	5
DMTN	0.3727	-0.3389	6
DMXL1	0.2593	-0.3943	2
DMXL2	0.1691	-0.2690	8
DNAJA1	0.5568	0.7127	1
DNAJA2	0.7752	-0.2875	1
DNAJA4	0.8903	0.1531	2
DNAJB1	0.9857	-0.0131	2
DNAJB2	0.7949	0.4450	2
DNAJB4	0.7949	-0.0812	2
DNAJC11	0.5153	-0.2273	2
DNAJC12	0.7053	-0.2324	1
DNAJC13	0.7159	-0.2448	1
DNAJC19	0.8963	-0.0814	3
DNAJC3	0.8375	-0.1135	3
DNAJC6	0.3238	-0.4589	4
DNAJC8	0.7187	-0.2135	5
DNM1L	0.5470	-0.1722	9
DNM2	0.2220	-0.3993	3
DNMT1	0.4791	1.1360	1
DNPEP	0.7551	0.2079	1
DOCK1	0.6079	-0.2282	4
DOCK9	0.9034	-0.0505	4
DOT1L	0.6008	0.3196	1
DPM1	0.9166	0.0734	1
DPP6	0.7187	-0.2180	3
DPYSL2	0.9733	-0.0132	15
DPYSL3	0.8118	0.1007	8
DPYSL4	0.6161	-0.2061	6
DPYSL5	0.7556	0.0896	10

Table S10: Non-significant synaptic proteins of cerebellar vermis in adults with idiopathic autism (FDR-adjusted p > 0.05) *(continued)*

Gene name	FDR-adjusted p-value	log2(fold-change)	Number of peptides
DR1	0.6816	-0.2145	1
DRG1	0.7565	0.2328	1
DRG2	0.6955	-0.2131	3
DROSHA	0.4100	0.6581	1
DSC1	0.6181	0.5839	1
DSC2	0.9797	0.0272	1
DST	0.8690	-0.0492	12
DSTN	0.2593	-0.3692	4
DTD1	0.9394	-0.0659	1
DTNA	0.7243	0.6830	1
DTX3	0.7935	-0.2377	1
DTYMK	0.9339	-0.0571	1
DUSP3	0.9762	-0.0275	3
DVL3	0.6378	-0.3189	1
DYNC1I1	0.1950	-0.6587	3
DYNC1I2	0.7590	-0.2263	2
DYNC1LI1	0.8986	-0.0485	8
DYNC1LI2	0.6303	0.2655	3
DYNLL2	0.8824	0.1039	2
DYNLRB1	0.4193	-0.4564	2
DYRK1A	0.7487	-0.2651	1
E9PLD3	0.8033	-0.1768	2
EARS2	0.9450	-0.0742	1
ECH1	0.9214	-0.0579	5
ECHDC1	0.6798	-0.3041	1
ECHDC2	0.7789	0.3126	1
ECHS1	0.1828	0.3216	6
ECI2	0.7629	-0.2331	3
ECM1	0.3688	0.7997	1
ECPAS	0.9086	-0.0496	3
EDC4	0.7556	0.1445	3
EDF1	0.6391	-0.2974	3
EDIL3	0.6449	0.3124	2
EEA1	0.9390	0.0235	11
EEF1A1	0.8381	0.3082	1
EEF1B2	0.2758	-0.4631	4
EEF1D	0.4628	-0.3004	3
EEF1E1	0.9963	-0.0017	1
EEF1G	0.4908	-0.2680	7
EEF2	0.7190	0.1049	9
EEFSEC	0.8866	0.3043	1
EFHD2	0.8796	0.2332	2
EFR3B	0.7098	-0.1984	2
EFTUD2	0.7317	-0.1427	5

Table S10: Non-significant synaptic proteins of cerebellar vermis in adults with idiopathic autism (FDR-adjusted p > 0.05) *(continued)*

Gene name	FDR-adjusted p-value	log2(fold-change)	Number of peptides
EGFR	0.7949	-0.1566	2
EHD1	0.5809	-0.2045	6
EHD2	0.9270	0.0562	2
EHD4	0.9870	-0.0077	3
EIF2A	0.7363	0.2722	2
EIF2AK2	0.9761	-0.0279	1
EIF2S1	0.9166	0.0613	2
EIF2S2	0.6578	-0.1984	3
EIF2S3	0.8813	0.1047	3
EIF3A	0.8420	0.1607	1
EIF3CL	0.8536	-0.0899	3
EIF3D	0.6185	-0.3880	2
EIF3E	0.8536	0.2194	1
EIF3F	0.4628	0.3358	3
EIF3H	0.8696	-0.0830	4
EIF3I	0.9417	-0.0661	2
EIF3J	0.9293	0.0894	2
EIF3K	0.7206	0.4095	1
EIF3L	0.5814	-0.6624	1
EIF4A1	0.4917	-0.2285	5
EIF4A2	0.8628	0.1895	1
EIF4A3	0.9618	-0.0301	5
EIF4B	0.7789	0.4095	1
EIF4G1	0.1539	-0.2720	4
EIF4G2	0.9791	0.0105	5
EIF5	0.8726	-0.1331	2
EIF5A2	0.2252	-0.4599	4
EIPR1	0.5002	-0.4408	2
ELAVL1	0.9946	-0.0035	4
ELAVL2	0.4210	-0.2529	9
ELAVL3	0.7053	-0.2232	4
ELMO1	0.7243	0.1869	1
ELMO2	0.7935	-0.5336	2
ELOB	0.7363	-0.1543	6
ELP1	0.6794	-0.3826	1
EMC1	0.9106	-0.0812	2
EMC7	0.9146	-0.0799	2
ENO2	0.8963	-0.0991	3
ENPP6	0.6237	0.5042	1
ENSA	0.9762	0.0509	1
EPB41	0.8628	-0.1278	3
EPB41L1	0.6542	-0.1530	8
EPB41L2	0.4157	-0.2642	4
EPB41L3	0.6828	-0.1365	9

Table S10: Non-significant synaptic proteins of cerebellar vermis in adults with idiopathic autism (FDR-adjusted p > 0.05) *(continued)*

Gene name	FDR-adjusted p-value	log2(fold-change)	Number of peptides
EPB42	0.8680	0.2703	1
EPDR1	0.7789	0.1815	2
EPHB2	0.8493	-0.1968	1
EPHX2	0.8903	0.1818	1
EPN2	0.8218	-0.1331	2
EPPK1	0.5814	0.1678	5
EPRS	0.7551	-0.1024	8
EPS15L1	0.7746	-0.1208	4
EPS8	0.8599	-0.3463	1
ERBIN	0.8493	-0.1605	1
ERC1	0.2593	-0.2442	8
ERGIC1	0.7949	0.1612	2
ERI2	0.9450	-0.0407	1
ERI3	0.7789	0.2707	1
ERLIN1	0.9293	0.0935	1
ERLIN2	0.6303	-0.3359	4
ERMN	0.5562	-0.3009	1
ERP29	0.8536	-0.1518	3
ESYT1	0.9808	0.0248	1
ESYT2	0.7796	0.1872	2
ETFA	0.9260	-0.0760	2
ETFB	0.8102	0.1396	3
EXOC1	0.7746	-0.1362	4
EXOC2	0.8813	-0.1255	1
EXOC3	0.5713	-0.4007	1
EXOC4	0.3935	-0.3256	3
EXOC5	0.8984	0.0994	1
EXOC6	0.9762	0.0224	1
EXOC6B	0.6192	-0.2626	3
EXOC7	0.5003	-0.2547	3
EXOC8	0.9662	-0.0243	1
EXOSC10	0.8542	-0.1688	2
EXOSC5	0.9093	0.1105	1
EZR	0.4628	0.5321	3
FABP3	0.7306	0.4107	2
FABP5	0.4646	1.0274	1
FABP7	0.7317	0.2446	1
FAH	0.8548	0.1863	1
FAHD1	0.5104	-0.8288	1
FAM107B	0.6766	-0.3143	1
FAM126B	0.9816	0.0095	2
FAM129B	0.9812	-0.0138	3
FAM131B	0.6585	-0.5396	1
FAM136A	0.9808	0.0189	2

Table S10: Non-significant synaptic proteins of cerebellar vermis in adults with idiopathic autism (FDR-adjusted p > 0.05) *(continued)*

Gene name	FDR-adjusted p-value	log2(fold-change)	Number of peptides
FAM13C	0.9661	-0.0575	1
FAM160B1	0.6888	-0.1388	2
FAM169A	0.6941	-0.4440	1
FAM171A1	0.4550	-0.4299	1
FAM171B	0.9417	-0.0608	1
FAM177A1	0.4853	-0.2554	1
FAM192A	0.5104	-0.3582	1
FAM45A	0.7589	-0.2773	1
FAM49B	0.7053	-0.3116	1
FAM98B	0.7565	-0.4769	1
FARP1	0.9937	-0.0033	5
FARSA	0.4193	-0.3662	4
FASN	0.4210	-0.1119	22
FAU	0.9258	-0.0897	2
FBXO41	0.5486	0.4141	1
FCGBP	0.5927	0.3125	1
FCHO2	0.9870	-0.0197	1
FECH	0.1169	-0.3428	4
FEN1	0.8645	-0.1406	3
FER	0.8542	-0.1916	1
FERMT2	0.8690	-0.1357	1
FGA	0.9259	-0.0399	6
FGF12	0.9735	-0.0172	2
FH	0.8963	0.0802	4
FHL1	0.9944	-0.0025	5
FIBP	0.9761	0.0603	1
FKBP15	0.9166	0.1267	1
FKBP2	0.4222	-0.7128	1
FKBP3	0.9148	-0.1003	2
FKBP4	0.6714	-0.1693	7
FKBP8	0.9271	0.0855	1
FLG2	0.1828	0.9124	2
FLNA	0.8473	-0.0523	12
FLNB	0.8625	0.0977	3
FLOT1	0.6888	-0.1658	6
FLOT2	0.8774	0.0722	5
FLYWCH2	0.9270	-0.1144	1
FMN2	0.6486	-0.4814	1
FMNL1	0.5562	-0.3946	1
FMNL2	0.8375	-0.1121	3
FN3K	0.7789	-0.2796	2
FNBP1	0.9761	0.0165	7
FNTB	0.9812	0.0335	1
FOXO1	0.6867	0.7300	1

Table S10: Non-significant synaptic proteins of cerebellar vermis in adults with idiopathic autism (FDR-adjusted p > 0.05) *(continued)*

Gene name	FDR-adjusted p-value	log2(fold-change)	Number of peptides
FRY	0.2682	-0.3446	5
FSCN1	0.4854	-0.3172	4
FSD1	0.4094	-0.5501	2
FSD1L	0.7032	-0.2501	2
FTH1	0.3935	0.5745	2
FTL	0.5302	0.7884	1
FTO	0.8903	0.0533	3
FUBP1	0.4193	-0.3375	6
FUBP3	0.6181	-0.4740	2
FUNDC2	0.9535	-0.0359	1
FUS	0.6798	-0.2392	3
FXR1	0.6828	-0.4200	2
FYN	0.4368	-0.2900	2
FYTTD1	0.7590	0.7164	1
G3BP2	0.9809	-0.0220	3
GAA	0.6714	0.3059	1
GABARAPL2	0.9292	0.0609	3
GABBR1	0.4621	-0.2611	4
GABBR2	0.4597	-0.2934	3
GABRA1	0.4193	-0.3119	4
GABRA6	0.7513	-0.2318	3
GABRB2	0.9801	0.0342	3
GABRG2	0.6714	-0.1655	1
GAD1	0.9713	-0.0331	2
GAD2	0.8796	0.3136	1
GAK	0.5857	-0.2446	3
GALK1	0.7775	-0.2352	1
GANAB	0.4884	-0.4078	1
GAP43	0.8446	-0.1539	5
GAPDH	0.0931	-0.1821	29
GAPVD1	0.6901	0.5053	2
GARS	0.7713	-0.2019	2
GART	0.3283	-0.3116	1
GATAD2B	0.9761	-0.0416	1
GATD1	0.5005	-0.8814	1
GATD3B	0.7532	-0.1959	3
GBA	0.6217	0.6168	1
GBE1	0.7187	-0.2693	1
GBF1	0.8783	0.0951	1
GCKR	0.9630	-0.0344	1
GCLC	0.6714	-0.1808	2
GCN1	0.8680	-0.0897	4
GDI1	0.9946	0.0020	7
GDI2	0.5691	0.3370	3

Table S10: Non-significant synaptic proteins of cerebellar vermis in adults with idiopathic autism (FDR-adjusted p > 0.05) *(continued)*

Gene name	FDR-adjusted p-value	log2(fold-change)	Number of peptides
GET4	0.4187	-0.5755	1
GFAP	0.5568	0.1181	29
GFM1	0.8903	0.1151	2
GGA3	0.8153	-0.3417	1
GGH	0.6578	0.2844	1
GIPC1	0.9911	-0.0105	1
GIPC3	0.9535	0.0944	1
GIT1	0.9106	-0.0858	2
GIT2	0.4100	-0.5880	1
GK	0.6714	-0.3344	2
GKAP1	0.4725	0.5200	1
GLCE	0.6853	-0.6485	1
GLIPR2	0.9040	-0.0916	2
GLO1	0.9808	0.0147	3
GLOD4	0.5525	0.3336	3
GLRX	0.9058	0.1206	1
GLRX3	0.9789	-0.0533	1
GLS	0.6008	-0.2308	3
GLUD1	0.9259	-0.0357	13
GLUL	0.9214	0.0568	5
GM2A	0.7415	0.3783	1
GMEB1	0.6676	0.3424	1
GMFB	0.7988	-0.1861	2
GMPS	0.8536	-0.1590	2
GNA11	0.8960	-0.0859	3
GNA12	0.7590	0.3259	1
GNA13	0.7392	-0.2936	1
GNAI2	0.4977	-0.3281	3
GNAO1	0.7912	-0.1031	5
GNAQ	0.8903	-0.0562	8
GNAS	0.8783	-0.0784	4
GNAZ	0.1303	-0.3049	7
GNB1	0.9365	-0.1326	1
GNB4	0.3467	-0.4030	3
GNB5	0.0888	-0.7448	3
GNE	0.9322	-0.0806	1
GNG13	0.8986	0.1378	1
GNL1	0.4210	-0.6565	1
GNPDA1	0.9658	-0.0711	1
GOLGA3	0.1765	-0.4266	3
GOLGB1	0.8574	0.1831	1
GOT1	0.4094	0.2703	8
GOT2	0.9658	0.0233	6
GPD1	0.4628	0.8697	1

Table S10: Non-significant synaptic proteins of cerebellar vermis in adults with idiopathic autism (FDR-adjusted p > 0.05) *(continued)*

Gene name	FDR-adjusted p-value	log2(fold-change)	Number of peptides
GPHN	0.7053	-0.1539	3
GPI	0.9351	-0.0227	10
GPM6A	0.8448	0.2455	1
GPNMB	0.5030	0.6912	1
GPR158	0.3090	-0.3456	2
GPRC5B	0.8726	-0.0904	1
GPRIN1	0.7590	-0.0983	9
GPRIN3	0.8625	-0.1681	3
GPS1	0.9791	0.0176	2
GPSM2	0.9965	-0.0015	1
GPX4	0.8903	-0.1396	1
GRAMD2B	0.5525	0.5551	1
GRB2	0.9713	0.0357	1
GRHPR	0.9762	0.0555	1
GRIA1	0.7417	-0.1450	5
GRIA2	0.1828	-0.2743	6
GRIA3	0.3119	-0.2624	5
GRIA4	0.0593	-0.5189	3
GRID2IP	0.8064	-0.1839	2
GRIN1	0.6469	-0.2714	1
GRIPAP1	0.5231	-0.3972	2
GRK2	0.0721	-0.5172	3
GRK5	0.9900	-0.0127	1
GRM1	0.8645	-0.1445	3
GRM4	0.8054	-0.1260	2
GRM5	0.1013	-1.0787	2
GRPEL1	0.4791	-0.2925	1
GRWD1	0.7609	0.3392	1
GSDMA	0.4193	1.1431	1
GSK3B	0.8054	-0.1719	2
GSN	0.0844	0.3605	10
GSPT1	0.8020	-0.1026	4
GSPT2	0.7832	-0.2700	1
GSR	0.9093	-0.0991	2
GSS	0.2108	0.4636	3
GSTM2	0.6936	0.4463	1
GSTP1	0.7556	0.4516	1
GTF2F2	0.9132	-0.1688	1
GTF2I	0.2439	-0.3365	5
GTPBP1	0.8536	-0.1813	1
GTPBP10	0.8020	-0.1960	1
GTPBP4	0.5562	0.3911	1
GUCY1A1	0.7912	-0.1710	1
GUCY1A2	0.8153	-0.2096	1

Table S10: Non-significant synaptic proteins of cerebellar vermis in adults with idiopathic autism (FDR-adjusted p > 0.05) *(continued)*

Gene name	FDR-adjusted p-value	log2(fold-change)	Number of peptides
GUCY1B1	0.7565	0.1040	2
GYG1	0.7173	0.1999	3
GYS1	0.8796	-0.1145	2
H1F0	0.7176	-0.2151	8
H1FX	0.8690	-0.0769	8
H2AFX	0.7098	-0.2369	3
H2AFY	0.7363	-0.1552	10
H2AFY2	0.4131	-0.3131	8
H3BN98	0.8352	0.1738	3
H3F3A	0.9811	-0.0259	4
HACD3	0.2888	0.3284	3
HADH	0.7481	-0.5154	1
HADHA	0.6185	-0.1137	14
HADHB	0.7775	0.1064	6
HAGH	0.8680	-0.1397	2
HAPLN1	0.6714	0.3653	2
HAPLN2	0.2593	0.7337	3
HARS	0.7792	-0.1166	6
HBA1	0.5573	0.3486	8
HBB	0.8960	0.1235	7
HBD	0.5144	0.3132	7
HBS1L	0.4854	-0.6419	1
HCFC1	0.1436	-0.3431	9
HCN2	0.8118	-0.2462	1
HDAC11	0.2305	0.3960	2
HDAC5	0.9484	-0.0383	1
HDGF	0.7511	-0.2387	1
HDGFL2	0.8625	-0.1368	1
HDGFRP3	0.9926	0.0089	2
HDLBP	0.9929	-0.0095	1
HEBP1	0.2722	0.4855	1
HECTD4	0.4094	-0.2284	3
HECW2	0.6375	-0.3718	1
HEPACAM	0.7635	-0.1277	3
HEXB	0.6577	0.3820	1
HIBCH	0.6867	-0.3034	1
HIP1	0.7565	-0.1372	4
HIP1R	0.7565	-0.1426	5
HIST1H1B	0.9109	-0.0562	12
HIST1H1D	0.5151	-0.3076	11
HIST1H1E	0.8903	-0.1561	2
HIST1H4A	0.7632	-0.1561	13
HIST2H2BC	0.9106	-0.2095	2
HIST2H2BF	0.6714	-0.1975	13

Table S10: Non-significant synaptic proteins of cerebellar vermis in adults with idiopathic autism (FDR-adjusted p > 0.05) *(continued)*

Gene name	FDR-adjusted p-value	log2(fold-change)	Number of peptides
HIST3H2A	0.6714	-0.3459	8
HIST3H2BB	0.8690	0.2602	1
HLCS	0.8905	-0.0942	1
HMGA1	0.8780	0.1281	2
HMGCS1	0.7789	-0.1046	1
HMGN2	0.9812	0.0290	2
HMGN4	0.9484	-0.1196	1
HMOX2	0.9761	-0.0210	1
HNMT	0.7984	0.2780	1
HNRNPA0	0.6798	0.2328	1
HNRNPA1	0.8768	-0.0490	4
HNRNPA2B1	0.4791	-0.3373	5
HNRNPA3	0.4187	-1.2479	1
HNRNPAB	0.8960	-0.1350	1
HNRNPC	0.1312	-0.3992	9
HNRNPCL4	0.5885	-0.6915	1
HNRNPD	0.4837	-0.2882	4
HNRNPDL	0.9812	-0.0225	3
HNRNPF	0.7565	-0.3852	1
HNRNPH1	0.4507	-0.3042	2
HNRNPH3	0.8269	-0.1981	4
HNRNPK	0.0845	-0.2997	11
HNRNPL	0.8381	0.1054	2
HNRNPM	0.2343	-0.4187	5
HNRNPR	0.9270	-0.0477	8
HNRNPU	0.8020	-0.1435	10
HNRNPUL1	0.5302	0.5229	1
HNRNPUL2	0.7949	-0.1273	3
HOMER1	0.3119	-0.4638	3
HOMER2	0.3688	-1.0007	1
HOMER3	0.2100	-0.3417	8
HOOK3	0.9109	-0.0800	1
HP	0.8243	0.1937	2
HP1BP3	0.5568	-0.2994	7
HPCA	0.8054	0.1809	3
HPCAL4	0.9578	0.0703	1
HPRT1	0.7053	0.5740	1
HSD17B10	0.6048	-0.1705	5
HSD17B11	0.7789	-0.4041	1
HSD17B12	0.8963	-0.1503	1
HSD17B4	0.4452	-0.1801	15
HSD17B8	0.8625	-0.1409	3
HSDL2	0.6079	-0.3684	2
HSP90AB1	0.6578	-0.1519	8

Table S10: Non-significant synaptic proteins of cerebellar vermis in adults with idiopathic autism (FDR-adjusted p > 0.05) *(continued)*

Gene name	FDR-adjusted p-value	log2(fold-change)	Number of peptides
HSP90B1	0.7036	0.1337	9
HSPA12A	0.1259	-0.3909	8
HSPA1B	0.9851	-0.0052	21
HSPA2	0.5486	0.1750	9
HSPA4	0.7949	-0.0788	11
HSPA4L	0.8033	-0.1017	5
HSPA5	0.8542	-0.0577	11
HSPA6	0.7690	-0.1625	3
HSPA8	0.0874	-0.2483	17
HSPA9	0.9722	0.0167	11
HSPB1	0.8233	0.0797	7
HSPD1	0.4210	0.1462	18
HSPE1	0.9565	0.0267	8
HSPH1	0.7909	-0.1666	3
HTATSF1	0.8054	-0.2354	1
HTT	0.7513	-0.1534	3
HUWE1	0.5343	-0.1337	10
HYOU1	0.7176	0.2008	1
I3L4J1	0.9669	0.0362	3
IARS	0.8480	-0.0809	6
IARS2	0.8054	-0.1227	4
ICMT	0.8796	-0.1258	1
IDE	0.2088	0.4829	2
IDH1	0.4837	0.5117	1
IDH2	0.5470	-0.3747	2
IDH3A	0.8429	-0.0783	7
IDH3B	0.7989	0.2767	1
IDH3G	0.9166	-0.0503	2
IGBP1	0.2831	-0.4453	2
IGHA1	0.5499	0.5486	2
IGHG1	0.8903	-0.0871	5
IGHG2	0.8368	0.1466	3
IGHG4	0.9166	-0.1829	1
IGHV4	0.5691	0.5981	1
IGKC	0.3432	0.6107	4
IGLC2	0.7523	0.2691	4
IGLL5	0.7628	0.2044	4
IGLON5	0.8903	-0.0804	3
IGLV7	0.9412	-0.1178	1
IGLV8	0.8447	-0.2574	1
IGSF21	0.9019	-0.0649	3
IGSF8	0.3748	-0.1863	13
IGSF9B	0.9271	0.0652	1
IK	0.7565	-0.2880	3

Table S10: Non-significant synaptic proteins of cerebellar vermis in adults with idiopathic autism (FDR-adjusted p > 0.05) *(continued)*

Gene name	FDR-adjusted p-value	log2(fold-change)	Number of peptides
IKBKB	0.9484	0.0316	1
IL16	0.2619	-0.5035	1
IL1RAP	0.9658	0.0798	1
IL36G	0.2745	0.5577	2
IL37	0.5038	0.7055	1
ILF2	0.2800	-0.5052	2
ILF3	0.3757	-0.2955	6
ILK	0.1736	-0.4477	3
ILKAP	0.8665	-0.2590	1
ILVBL	0.5470	-0.3451	2
IMMT	0.3738	-0.1546	16
IMPDH2	0.9312	0.1317	1
INA	0.2100	-0.2653	20
INPP4A	0.7306	0.1845	3
INPP5A	0.6064	-0.2157	5
INPP5F	0.7780	-0.1139	2
INPPL1	0.8489	-0.1675	1
IPO5	0.6393	-0.2878	1
IPO7	0.6449	-0.4441	1
IPO9	0.8625	-0.1642	1
IQGAP1	0.9963	0.0019	1
IQSEC1	0.9761	-0.0429	3
IQSEC3	0.7949	0.1618	1
IRF2BP1	0.9044	-0.1665	1
IRGQ	0.8172	-0.2244	1
ISCA1	0.3016	-0.7193	2
ISCU	0.9259	-0.1149	1
IST1	0.8455	0.0664	7
ITGB1	0.9259	0.0982	1
ITIH2	0.8549	0.2575	1
ITM2B	0.9278	-0.1018	1
ITPA	0.9749	0.0309	1
ITPKB	0.8101	-0.1726	1
ITPR1	0.0743	-0.2375	32
ITPR2	0.7749	-0.1346	1
IVD	0.8020	0.1211	4
J3QQQ9	0.8796	0.1081	2
JAK2	0.9260	-0.0859	1
JCHAIN	0.4094	0.7736	1
JPH3	0.8448	-0.1972	1
KARS	0.9812	0.0106	3
KATNB1	0.7047	-0.1770	1
KAZN	0.4351	-0.2837	5
KBTBD11	0.6901	-0.2305	3

Table S10: Non-significant synaptic proteins of cerebellar vermis in adults with idiopathic autism (FDR-adjusted p > 0.05) *(continued)*

Gene name	FDR-adjusted p-value	log2(fold-change)	Number of peptides
KCNA1	0.4193	-0.2881	3
KCNAB2	0.8054	0.1205	4
KCNK9	0.8448	0.1673	1
KCTD12	0.8446	0.2334	1
KCTD8	0.7097	-0.4531	1
KHDRBS1	0.5450	-0.4681	4
KHSRP	0.6828	-0.5717	1
KIAA0513	0.9761	-0.0322	2
KIAA1107	0.8931	-0.0937	5
KIAA1109	0.4837	-0.3347	1
KIAA1191	0.1691	-0.5878	2
KIAA1217	0.9166	-0.0653	3
KIF1A	0.5302	-0.1802	5
KIF1BP	0.6647	0.2052	2
KIF21A	0.5420	-0.2097	7
KIF2A	0.3563	-0.2961	3
KIF3A	0.4536	-0.3488	2
KIF3B	0.6542	-0.2392	2
KIF5B	0.5210	-0.2259	4
KIF5C	0.8433	-0.1065	7
KIT	0.4121	-0.3301	2
KLC1	0.6598	-0.1435	8
KLC2	0.4854	-0.3916	2
KPNA3	0.7362	-0.3295	1
KPNA5	0.7159	-0.3310	1
KPNB1	0.1013	-0.3139	5
KRT13	0.8549	0.1420	4
KRT19	0.7962	0.3250	1
KRT23	0.5809	0.4536	2
KRT4	0.8279	0.3153	2
KRT73	0.4210	0.6005	3
KRT8	0.5823	1.0859	1
KTN1	0.3042	-0.2259	12
KYAT1	0.9122	-0.0898	2
L1CAM	0.5610	-0.2157	6
LACRT	0.6735	0.4133	2
LACTB	0.9259	-0.1079	1
LAMA2	0.6377	0.4545	1
LAMB2	0.6338	-0.2282	3
LAMP1	0.7935	0.3125	1
LAMTOR1	0.8506	0.1280	2
LAMTOR2	0.8903	0.1215	1
LAP3	0.9893	0.0089	2
LARP1	0.2784	-0.3538	1

Table S10: Non-significant synaptic proteins of cerebellar vermis in adults with idiopathic autism (FDR-adjusted p > 0.05) *(continued)*

Gene name	FDR-adjusted p-value	log2(fold-change)	Number of peptides
LARS	0.8606	-0.0664	7
LARS2	0.1781	-0.5874	2
LASP1	0.8375	0.2165	1
LCN1	0.5637	0.7088	1
LDHA	0.8726	0.0968	3
LDHB	0.5932	0.2056	8
LEMD3	0.9109	0.2452	1
LETM1	0.6005	-0.9085	1
LGALS1	0.8273	0.1225	4
LGALS3BP	0.6630	0.3576	1
LGALS7	0.5231	0.5787	2
LGALSL	0.8703	-0.1556	1
LGI1	0.8123	-0.0626	5
LIG3	0.9178	-0.0614	1
LIMA1	0.9570	-0.0361	1
LIMK1	0.8433	-0.1426	1
LIMS1	0.8494	0.1786	1
LIN7A	0.5005	-0.2695	2
LITAF	0.9169	-0.0914	1
LLGL1	0.5857	-0.3125	2
LMAN1	0.9109	0.1064	1
LMAN2	0.7551	0.2404	1
LMCD1	0.9578	0.0690	1
LMNA	0.9367	-0.0303	7
LMNB1	0.0562	-0.3974	11
LMNB2	0.7565	-0.1431	11
LMO7	0.7792	-0.1689	1
LMTK3	0.9893	0.0123	1
LONP1	0.3523	-0.2373	7
LPP	0.6420	0.7328	1
LRCH3	0.9946	0.0036	1
LRP1	0.9484	-0.0317	3
LRPAP1	0.8680	0.1477	1
LRPPRC	0.3935	-0.2179	9
LRRC34	0.9214	0.0932	1
LRRC47	0.0669	-0.4022	7
LRRC59	0.7532	-0.2648	2
LSAMP	0.4742	-0.1657	8
LSM14B	0.8542	-0.2037	1
LSM6	0.9484	0.0686	1
LSM7	0.7306	-0.2863	1
LSM8	0.9412	0.0526	1
LTA4H	0.8118	0.1487	2
LTF	0.8589	0.1354	2

Table S10: Non-significant synaptic proteins of cerebellar vermis in adults with idiopathic autism (FDR-adjusted p > 0.05) *(continued)*

Gene name	FDR-adjusted p-value	log2(fold-change)	Number of peptides
LUC7L2	0.9148	-0.0856	2
LYN	0.8448	-0.1672	1
LYPLA1	0.4495	0.2979	3
LYPLA2	0.5002	0.3047	2
LYPLAL1	0.9827	-0.0227	1
LYSMD2	0.7321	-0.2997	2
LYZ	0.5144	0.8402	1
LZTFL1	0.5568	-0.4632	1
M0QYV0	0.5885	-0.3405	2
MAB21L2	0.6486	-0.2485	3
MADD	0.8690	0.0478	6
MAFG	0.9529	0.0520	1
MAG	0.7187	0.2645	1
MAGED2	0.7603	-0.1734	3
MAIP1	0.7206	-0.1990	2
MAOA	0.3738	-0.3380	4
MAP11	0.8960	-0.1095	1
MAP1A	0.4421	-0.1292	22
MAP1LC3A	0.5104	-0.2611	3
MAP1S	0.8330	-0.1124	2
MAP2	0.1533	-0.3878	11
MAP2K1	0.9339	-0.0360	3
MAP2K2	0.5104	-0.3531	3
MAP2K4	0.8980	0.2287	1
MAP2K6	0.6391	-0.2341	2
MAP2K7	0.9953	0.0020	1
MAP3K15	0.8963	-0.0632	4
MAP3K5	0.7789	0.3583	1
MAP4	0.3077	-0.2362	16
MAP4K3	0.9946	0.0091	1
MAP4K4	0.4193	-0.2467	4
MAP6	0.8448	-0.0795	9
MAP6D1	0.9313	-0.0562	1
MAP7D1	0.7988	-0.1333	2
MAPK1	0.4628	-0.2302	10
MAPK11	0.4193	-0.2086	1
MAPK3	0.9630	0.0583	2
MAPK8IP3	0.7362	0.1938	2
MAPRE2	0.4837	-0.3154	6
MAPRE3	0.7532	-0.1553	4
MAPT	0.1806	-0.2896	13
MARC2	0.8986	-0.1899	1
MARCKS	0.8796	-0.1092	6
MARCKSL1	0.7795	0.2287	2

Table S10: Non-significant synaptic proteins of cerebellar vermis in adults with idiopathic autism (FDR-adjusted p > 0.05) *(continued)*

Gene name	FDR-adjusted p-value	log2(fold-change)	Number of peptides
MARK3	0.8960	-0.0441	4
MARS	0.8690	-0.1009	4
MATN2	0.7159	-0.5250	1
MATR3	0.7949	-0.1846	6
MCAT	0.7357	-0.2603	1
MCCC1	0.7550	-0.2065	1
MCCC2	0.6094	0.2354	4
MCEE	0.7565	0.4489	1
MCF2L	0.3757	-0.4581	1
MCTS1	0.9791	0.0561	1
MDH1	0.2220	0.3909	7
ME1	0.5497	0.6763	1
ME2	0.9312	0.0683	3
ME3	0.7789	-0.1940	1
MECP2	0.8928	-0.0698	7
MEF2D	0.8680	-0.0892	2
METAP1	0.9334	-0.0910	1
METTL3	0.5272	-0.3828	1
MFN1	0.8033	-0.1410	2
MFN2	0.8625	0.3452	1
MGST3	0.8625	-0.1481	1
MICAL3	0.8322	-0.0969	6
MICOS13	0.9222	-0.0988	1
MICU3	0.8684	-0.0943	2
MLYCD	0.9762	-0.0277	1
MMAA	0.9761	-0.0206	2
MMS19	0.7789	0.2568	1
MMUT	0.6999	0.2937	2
MOBP	0.5809	0.5057	1
MOCS1	0.7749	-0.3007	1
MOG	0.5908	0.7378	1
MORC2	0.5893	-0.5287	1
MORC3	0.7949	0.1819	1
MPI	0.8054	-0.2168	1
MPP2	0.2754	-0.3967	7
MPP3	0.5869	-0.4843	2
MPRIP	0.8806	-0.0617	3
MRAS	0.5035	-0.2111	4
MROH1	0.9618	-0.0583	1
MRPL1	0.9664	0.0284	1
MRPL12	0.9484	-0.0363	3
MRPL14	0.9106	0.1235	1
MRPL19	0.9178	0.0702	1
MRPL20	0.8498	-0.1312	1

Table S10: Non-significant synaptic proteins of cerebellar vermis in adults with idiopathic autism (FDR-adjusted p > 0.05) *(continued)*

Gene name	FDR-adjusted p-value	log2(fold-change)	Number of peptides
MRPL55	0.8473	0.0830	1
MRPS15	0.9812	0.0340	1
MRPS16	0.6017	0.4933	1
MRPS17	0.8797	0.0995	1
MRPS23	0.7363	0.4505	1
MRPS31	0.9762	0.0444	1
MRRF	0.9339	-0.0502	2
MSN	0.0930	0.6802	4
MT	0.8589	-0.1146	2
MTA1	0.5005	-0.2340	2
MTA2	0.5857	-0.3678	1
MTCH1	0.9628	0.0359	3
MTCH2	0.5035	-0.3581	1
MTDH	0.8218	-0.1943	2
MTHFD1	0.2252	-0.2256	9
MTHFD1L	0.5104	-0.5214	2
MTMR1	0.9893	0.0138	1
MTMR10	0.9453	0.1173	1
MTOR	0.8054	-0.1304	2
MTREX	0.1586	-0.4476	1
MTSS1	0.4323	0.5641	1
MTSS2	0.5932	0.3750	3
MTX2	0.8542	0.1278	2
MTX3	0.6161	0.2748	1
MVD	0.5324	0.3426	1
MVK	0.8020	-0.1971	1
MVP	0.8726	0.1944	1
MYBBP1A	0.3688	-0.5763	1
MYCBP	0.9658	0.0671	1
MYEF2	0.9058	0.0929	3
MYH10	0.4664	-0.1478	17
MYH11	0.7317	-0.1791	3
MYH14	0.7363	0.1010	7
MYH7	0.7949	0.4540	1
MYH9	0.2488	0.1821	16
MYL12B	0.9292	0.0597	2
MYL6	0.6391	0.2960	2
MYLK2	0.7032	0.3578	2
MYO18A	0.6181	-0.1233	13
MYO1C	0.7053	-0.1348	4
MYO1D	0.6578	0.2018	3
MYO5A	0.2093	-0.2401	12
MYO6	0.7792	0.0855	7
NAA35	0.8233	-0.1484	1

Table S10: Non-significant synaptic proteins of cerebellar vermis in adults with idiopathic autism (FDR-adjusted p > 0.05) *(continued)*

Gene name	FDR-adjusted p-value	log2(fold-change)	Number of peptides
NAB2	0.9658	0.0894	2
NACA	0.8381	-0.1859	1
NAMPT	0.8054	-0.1108	4
NANS	0.8726	-0.0825	2
NAP1L4	0.8680	-0.1714	1
NAPA	0.9093	0.1248	2
NAPB	0.4854	-0.4587	2
NAPG	0.9484	-0.0270	5
NARS	0.9826	0.0246	1
NASP	0.7565	-0.3158	1
NAV3	0.9679	0.0524	1
NAXD	0.8433	0.1275	4
NAXE	0.8020	0.2128	1
NBAS	0.8690	0.2977	1
NBEA	0.9791	0.0459	3
NCAM1	0.7795	-0.1094	9
NCAM2	0.9761	-0.0289	2
NCAN	0.7532	0.2059	6
NCBP1	0.9388	-0.0725	1
NCDN	0.8806	0.0770	2
NCEH1	0.8548	-0.2140	1
NCK2	0.8123	-0.2480	1
NCKAP1	0.4210	0.1226	17
NCKAP1L	0.3467	0.7543	1
NCL	0.6901	-0.1040	18
NCOA5	0.7949	0.2883	1
NCOA7	0.7912	-0.1380	2
NDRG2	0.7909	0.3979	2
NDRG3	0.7053	-0.4610	1
NDRG4	0.8597	0.2105	2
NDUFA10	0.3552	-0.3558	2
NDUFA12	0.7047	-0.3330	2
NDUFA2	0.8455	-0.1301	4
NDUFA3	0.5420	-0.3532	2
NDUFA4	0.8866	-0.0893	4
NDUFA5	0.4908	-0.5009	1
NDUFA6	0.7976	-0.1886	2
NDUFA7	0.9184	0.0561	2
NDUFA8	0.6315	-0.3143	2
NDUFB10	0.8903	0.1699	1
NDUFB11	0.8377	-0.1602	2
NDUFB3	0.8469	-0.1550	4
NDUFB4	0.5551	-0.7876	1
NDUFB5	0.7813	-0.2358	2

Table S10: Non-significant synaptic proteins of cerebellar vermis in adults with idiopathic autism (FDR-adjusted p > 0.05) *(continued)*

Gene name	FDR-adjusted p-value	log2(fold-change)	Number of peptides
NDUFB6	0.9808	0.0310	1
NDUFC2	0.9808	-0.0356	1
NDUFS1	0.6714	-0.1779	6
NDUFS2	0.7937	0.5033	1
NDUFS3	0.8330	-0.1088	5
NDUFS4	0.7949	-0.1559	4
NDUFS6	0.8054	-0.3024	1
NDUFS7	0.1424	-0.4975	4
NDUFS8	0.6054	-0.7360	1
NDUFV1	0.7792	0.1412	3
NDUFV2	0.8680	-0.1190	2
NEBL	0.2439	-1.3436	1
NECAP1	0.6310	-0.3504	2
NEDD4L	0.6048	-0.5302	1
NEFH	0.6828	-0.1564	8
NEFL	0.4193	-0.2311	19
NEFM	0.6181	-0.1637	16
NEGR1	0.6420	-0.1844	3
NEK8	0.7611	-0.2903	1
NEMF	0.8853	-0.0818	3
NEO1	0.9292	0.0526	2
NES	0.6515	-0.6513	1
NF1	0.7265	-0.2048	2
NFASC	0.7447	0.1509	4
NHP2	0.8494	0.2024	1
NIPSNAP2	0.8020	0.1542	2
NKIRAS1	0.9812	0.0132	3
NLGN3	0.7556	0.4903	1
NME1	0.9146	-0.0851	3
NME3	0.4854	0.3427	1
NME7	0.8797	0.1224	1
NMT1	0.7047	-0.5760	1
NNT	0.6272	-0.1529	9
NOA1	0.6714	0.9186	1
NOLC1	0.9435	0.0691	2
NOMO2	0.8690	-0.2589	1
NONO	0.6542	-0.6748	1
NOP9	0.5302	-0.4704	1
NOS1	0.9060	-0.2400	1
NOVA1	0.1394	-0.2735	13
NOVA2	0.6828	-0.5606	1
NPEPPS	0.4628	0.2191	9
NPM1	0.7789	0.1145	9
NPTN	0.9998	0.0001	1

Table S10: Non-significant synaptic proteins of cerebellar vermis in adults with idiopathic autism (FDR-adjusted p > 0.05) *(continued)*

Gene name	FDR-adjusted p-value	log2(fold-change)	Number of peptides
NRBP1	0.9346	0.0507	1
NRCAM	0.9086	-0.0552	5
NRXN1	0.5501	-0.3314	2
NRXN2	0.1828	-0.2904	4
NRXN3	0.4285	-0.2964	4
NSFL1C	0.6310	-0.3600	1
NSUN2	0.6714	-0.2876	1
NT5E	0.9166	0.0605	3
NTM	0.2824	-0.4014	3
NUBP1	0.5930	0.2994	2
NUBPL	0.9148	0.0879	2
NUCB2	0.8986	-0.0728	1
NUCKS1	0.9944	0.0067	2
NUDC	0.9484	-0.0358	2
NUDCD3	0.8690	-0.2350	1
NUDT16L1	0.9891	-0.0111	2
NUDT21	0.3345	-0.3614	3
NUDT5	0.6867	0.2478	1
NUFIP2	0.8974	-0.1561	1
NUMA1	0.5775	-0.1789	9
NUMB	0.5551	-0.8016	1
NUP133	0.5181	0.4812	2
NUP210	0.9509	-0.0333	3
NUP214	0.8784	0.0807	1
NUP43	0.8680	0.0588	2
NUP54	0.9870	-0.0208	1
NUP88	0.7949	-0.2111	2
NUP93	0.8448	0.1187	4
NUP98	0.8726	0.1244	2
NXF1	0.9093	-0.1169	1
OCIAD1	0.2682	-0.3733	1
OGA	0.9893	0.0110	1
OGDH	0.8960	-0.2219	1
OGT	0.8303	0.0975	8
OLA1	0.9578	-0.0271	4
OLFM1	0.6968	-0.2959	1
OLFM3	0.0672	-0.5240	4
OMG	0.6542	0.5030	1
OPA1	0.6934	-0.1204	11
OPCML	0.6542	-0.1826	5
OPLAH	0.7047	0.3343	1
OPTN	0.9293	0.0971	1
OSBP2	0.7565	0.2413	1
OSBPL11	0.5573	-0.2797	1

Table S10: Non-significant synaptic proteins of cerebellar vermis in adults with idiopathic autism (FDR-adjusted p > 0.05) *(continued)*

Gene name	FDR-adjusted p-value	log2(fold-change)	Number of peptides
OSBPL8	0.6955	-0.3429	1
OTUB1	0.9210	0.1019	1
OXCT1	0.6231	0.3566	2
OXR1	0.4193	-0.3498	4
OXSR1	0.9484	-0.0669	1
P00761	0.1806	0.9590	3
P4HB	0.7565	-0.1391	4
PA2G4	0.5002	-0.2834	8
PABPC1	0.7949	-0.0956	7
PABPC4	0.5151	-0.2835	5
PABPN1	0.6372	0.5524	1
PACS1	0.7556	-0.1299	5
PACS2	0.5747	-0.2409	1
PACSIN1	0.9423	0.0345	9
PACSIN2	0.6682	-0.1640	8
PADI2	0.8536	0.3562	1
PAFAH1B1	0.5420	0.4958	1
PAFAH1B3	0.6014	-0.2119	3
PAICS	0.9630	0.0386	3
PAK1	0.4854	-0.4124	2
PAK2	0.5809	-0.2615	2
PALM	0.7789	-0.0985	11
PANK4	0.4853	-0.2879	4
PARK7	0.1793	0.5799	3
PARP1	0.5679	-0.1678	5
PARVA	0.9264	-0.0753	1
PATJ	0.5780	0.6799	1
PAXX	0.9259	0.0957	1
PBDC1	0.4193	-0.5783	1
PBXIP1	0.8903	0.1332	2
PC	0.7955	-0.1113	5
PCBP1	0.7436	-0.1597	4
PCBP2	0.4927	-0.2378	4
PCBP3	0.4622	-0.2398	8
PCCA	0.9312	0.0561	2
PCCB	0.9812	0.0308	1
PCDH1	0.7556	-0.1917	1
PCDH9	0.8553	0.1034	1
PCK2	0.9484	-0.0474	1
PCLO	0.1607	-0.2446	18
PCMT1	0.7053	-0.2791	1
PCNA	0.9808	0.0212	1
PCNP	0.6372	-0.3377	2
PCP2	0.7053	-0.2257	3

Table S10: Non-significant synaptic proteins of cerebellar vermis in adults with idiopathic autism (FDR-adjusted p > 0.05) *(continued)*

Gene name	FDR-adjusted p-value	log2(fold-change)	Number of peptides
PCP4L1	0.6697	-0.6660	1
PCYOX1	0.8968	-0.1847	1
PDAP1	0.8903	-0.1094	3
PDCD10	0.9527	0.0306	2
PDCD4	0.3646	0.3141	1
PDCD6IP	0.8446	0.0628	10
PDE12	0.9458	0.0718	1
PDE1A	0.8903	-0.1465	1
PDHA1	0.8780	-0.0737	10
PDHB	0.5551	-0.1852	10
PDHX	0.7949	-0.0903	8
PDIA3	0.9034	-0.0528	5
PDIA4	0.5470	-0.5913	1
PDIA6	0.9570	-0.0502	2
PDK3	0.7789	-0.2473	1
PDLIM1	0.9259	-0.0995	1
PDLIM5	0.9086	-0.0851	1
PDPK1	0.7749	-0.1531	4
PDS5A	0.6054	-0.2332	2
PDS5B	0.9502	0.1468	1
PDXP	0.9598	0.0358	2
PEA15	0.1312	-0.4080	4
PEBP1	0.7958	0.1557	5
PEPD	0.8172	-0.1803	1
PEX11B	0.8171	-0.3461	1
PEX19	0.5917	-0.4253	1
PEX5L	0.9888	-0.0071	1
PFDN1	0.6691	-0.2179	4
PFDN2	0.9679	-0.0274	2
PFDN5	0.9926	0.0099	2
PFDN6	0.8960	-0.0876	3
PFKL	0.6079	-0.2256	4
PFKM	0.2619	-0.2609	12
PFKP	0.3619	-0.2554	8
PFN1	0.9462	-0.0390	4
PFN2	0.8293	-0.0806	4
PGAM1	0.8727	0.0811	3
PGAM5	0.8986	-0.0726	2
PGD	0.8504	-0.1037	6
PGK1	0.7053	0.1823	7
PGLS	0.9259	-0.0649	2
PGM1	0.8574	0.1263	2
PGM2	0.3646	0.6164	1
PGM2L1	0.9808	0.0287	1

Table S10: Non-significant synaptic proteins of cerebellar vermis in adults with idiopathic autism (FDR-adjusted p > 0.05) *(continued)*

Gene name	FDR-adjusted p-value	log2(fold-change)	Number of peptides
PGP	0.8273	-0.1639	1
PGRMC1	0.4791	0.2764	3
PHB	0.5373	-0.1459	13
PHB2	0.7912	-0.0877	12
PHC2	0.4193	-0.4037	1
PHF2	0.6542	0.3235	2
PHF20L1	0.9791	0.0209	1
PHF24	0.8680	-0.0893	1
PHF5A	0.9621	-0.0341	2
PHGDH	0.6934	-0.0947	17
PHKB	0.6714	-0.1431	1
PHLDB1	0.9807	0.0427	1
PHOX2B	0.9578	-0.1241	1
PHPT1	0.7789	0.2352	3
PHYHIP	0.6124	-0.3193	4
PHYHIPL	0.8054	0.1044	5
PI4KA	0.8218	0.0854	5
PI4KB	0.4187	0.4715	1
PICALM	0.4104	0.5835	3
PIGR	0.6207	0.4721	1
PIK3C3	0.5593	-0.1334	8
PIK3CA	0.3935	-0.5133	1
PIK3R1	0.6704	-0.2438	1
PIK3R4	0.8377	-0.1266	3
PIKFYVE	0.6490	-0.2170	2
PIN1	0.9109	-0.1646	1
PIP	0.0562	0.6486	4
PIP4K2A	0.7058	-0.2304	2
PIP4K2C	0.5857	-0.2519	2
PIPOX	0.5908	-0.3504	1
PITHD1	0.8606	-0.2490	1
PITPNC1	0.8903	0.1665	1
PITPNM1	0.7796	-0.2541	1
PITPNM2	0.9630	-0.0673	1
PITPNM3	0.9812	-0.0086	2
PKP1	0.5809	0.5322	2
PKP4	0.9862	0.0412	1
PLCB1	0.8574	-0.1583	2
PLCB3	0.8504	0.2617	1
PLCB4	0.5470	-0.4402	1
PLCD1	0.9870	-0.0137	1
PLCD3	0.9312	0.0400	1
PLCL1	0.7032	0.1784	2
PLCL2	0.4210	-0.3042	4

Table S10: Non-significant synaptic proteins of cerebellar vermis in adults with idiopathic autism (FDR-adjusted p > 0.05) *(continued)*

Gene name	FDR-adjusted p-value	log2(fold-change)	Number of peptides
PLD3	0.9816	0.0181	3
PLEC	0.7949	0.0350	61
PLEKHA5	0.9058	0.0926	1
PLEKHD1	0.1394	-0.6739	2
PLEKHH3	0.8797	-0.1118	1
PLEKHM2	0.2888	0.2818	1
PLP1	0.4550	0.3037	10
PLPP3	0.9893	0.0092	2
PLXNA1	0.7551	-0.2567	1
PLXNB1	0.8780	0.1903	1
PLXNB2	0.5530	-0.2556	3
PLXNC1	0.5562	-0.6874	1
PMP2	0.2911	0.6789	3
PMPCB	0.8273	0.1936	1
PMVK	0.8806	0.1319	4
PNN	0.9826	0.0216	1
PNP	0.7789	0.3108	1
PNPT1	0.7053	-0.2224	2
POGZ	0.7749	-0.2223	1
POLB	0.9826	0.0153	1
POLDIP2	0.8535	-0.2681	1
POLDIP3	0.6348	-0.5108	1
POLR2A	0.7912	-0.2144	2
POLR2B	0.9109	0.1876	1
POLR2L	0.9266	-0.0946	1
POMGNT2	0.7949	-0.4030	1
POR	0.7556	-0.1827	2
PPA1	0.8974	0.1575	1
PPA2	0.7053	0.3664	1
PPCS	0.9617	0.0605	1
PPFIA1	0.8153	0.5122	1
PPFIA3	0.3895	-0.4671	3
PPIA	0.8653	-0.1191	4
PPIB	0.5492	-0.2098	7
PPID	0.6971	-0.2060	2
PPIF	0.9811	-0.0272	2
PPIL1	0.9812	-0.0317	1
PPM1A	0.7317	-0.2798	1
PPM1B	0.9141	-0.1245	1
PPM1G	0.7984	0.2210	1
PPME1	0.7161	0.2797	1
PPP1CB	0.6372	-0.2741	1
PPP1R12A	0.9036	-0.1138	1
PPP1R12C	0.7362	0.1521	1

Table S10: Non-significant synaptic proteins of cerebellar vermis in adults with idiopathic autism (FDR-adjusted p > 0.05) *(continued)*

Gene name	FDR-adjusted p-value	log2(fold-change)	Number of peptides
PPP1R21	0.5834	-0.3255	1
PPP1R7	0.6449	0.3350	2
PPP1R9B	0.9417	-0.1155	1
PPP2CA	0.2593	-0.6963	2
PPP2R1A	0.9761	-0.0171	6
PPP2R2A	0.7537	0.2504	1
PPP2R5B	0.8796	0.1319	2
PPP2R5D	0.7949	0.0982	4
PPP3CA	0.7532	-0.1502	5
PPP3CB	0.6054	0.4330	2
PPP3R1	0.9893	-0.0130	1
PPP5C	0.5593	0.5556	1
PPT1	0.7789	-0.2277	1
PRAF2	0.8628	0.1625	1
PRDX1	0.1417	0.2197	16
PRDX2	0.6391	0.2045	6
PRDX5	0.9259	-0.0415	6
PRDX6	0.5562	0.4916	2
PRELP	0.9641	0.0457	1
PREPL	0.9762	0.0378	2
PREX1	0.4628	-0.3946	2
PRKAA1	0.7147	-0.3144	2
PRKACA	0.7789	-0.2986	1
PRKACB	0.8572	0.0857	3
PRKAG1	0.7362	-0.1591	4
PRKAR1A	0.8832	-0.2066	1
PRKAR2A	0.9870	-0.0167	1
PRKAR2B	0.9394	-0.0830	1
PRKCA	0.7142	0.2473	2
PRKCE	0.8950	0.3029	2
PRKCG	0.7789	-0.1583	4
PRKCSH	0.8680	-0.1226	1
PRKDC	0.3738	-0.1578	12
PRKG1	0.9749	-0.0248	2
PRKRA	0.9351	-0.0531	5
PRMT1	0.9578	-0.0547	2
PRMT5	0.7796	0.2264	1
PRNP	0.5648	0.5707	1
PRPF19	0.4193	-0.6585	3
PRPF31	0.8542	-0.1987	2
PRPF40A	0.7853	-0.1415	1
PRPF4B	0.2051	-1.5070	1
PRPF6	0.7949	0.1958	3
PRPF8	0.7789	-0.1714	3

Table S10: Non-significant synaptic proteins of cerebellar vermis in adults with idiopathic autism (FDR-adjusted p > 0.05) *(continued)*

Gene name	FDR-adjusted p-value	log2(fold-change)	Number of peptides
PRPS1	0.8389	0.1493	3
PRPSAP1	0.9103	0.0567	1
PRPSAP2	0.7176	0.2371	3
PRRC2A	0.8866	-0.0879	1
PRTFDC1	0.8574	-0.1058	2
PRXL2A	0.6542	0.3851	1
PSAT1	0.8680	0.0695	8
PSD3	0.4664	-0.1460	18
PSIP1	0.2736	-0.3465	5
PSMA1	0.6391	0.1642	6
PSMA2	0.7492	-0.1694	3
PSMA3	0.5551	0.2181	6
PSMA4	0.8448	0.2296	1
PSMA5	0.6542	-0.2247	2
PSMA6	0.9812	0.0126	4
PSMA7	0.8903	0.0790	4
PSMB1	0.6931	0.3076	2
PSMB2	0.8464	0.1904	1
PSMB4	0.7789	0.1640	4
PSMB5	0.8768	0.1184	4
PSMB6	0.5231	0.3138	3
PSMB7	0.8625	0.2237	1
PSMB8	0.7949	-0.2590	1
PSMB9	0.7538	0.4325	1
PSMC1	0.0672	-0.5292	3
PSMC2	0.8233	0.1498	4
PSMC3	0.7949	-0.1693	2
PSMC4	0.8330	-0.0834	5
PSMC5	0.4285	-0.1842	5
PSMC6	0.9761	-0.0204	3
PSMD1	0.1828	0.2853	7
PSMD10	0.6828	0.2473	2
PSMD11	0.7550	-0.1217	6
PSMD12	0.9106	-0.0761	2
PSMD13	0.8153	0.0953	7
PSMD14	0.6830	-0.2026	3
PSMD2	0.7243	0.1437	4
PSMD3	0.8663	-0.0740	4
PSMD4	0.8903	0.0824	2
PSMD7	0.9789	0.0218	1
PSMD8	0.6828	0.1729	4
PSMD9	0.6500	-0.4718	1
PSME2	0.7053	-0.3650	1
PTBP1	0.5343	-0.2908	3

Table S10: Non-significant synaptic proteins of cerebellar vermis in adults with idiopathic autism (FDR-adjusted p > 0.05) *(continued)*

Gene name	FDR-adjusted p-value	log2(fold-change)	Number of peptides
PTBP2	0.0930	-0.6293	4
PTGES3	0.8448	0.1714	1
PTK2B	0.9900	0.0044	2
PTMS	0.8796	-0.1051	2
PTPA	0.3935	-0.2867	2
PTPN1	0.1950	0.5972	1
PTPN11	0.8796	0.0691	3
PTPN23	0.9106	-0.0834	2
PTPRE	0.8973	-0.0640	1
PTPRG	0.8504	0.0924	1
PTPRZ1	0.8726	0.0443	12
PTRHD1	0.9791	-0.0353	1
PUF60	0.4628	-0.3786	3
PURA	0.5515	0.2234	7
PURB	0.6542	0.3188	3
PXN	0.8986	0.1037	1
PYCR3	0.8396	-0.1480	1
PYGB	0.7023	0.0916	11
PYGL	0.8628	-0.3006	1
PYGM	0.8836	-0.1202	2
QARS	0.9109	0.0977	3
QDPR	0.7945	0.1246	8
RAB10	0.7265	-0.1328	6
RAB11A	0.8680	-0.0747	3
RAB11FIP5	0.7949	-0.4001	1
RAB12	0.8903	-0.0781	1
RAB14	0.8418	0.1849	2
RAB1A	0.6377	0.3086	2
RAB24	0.7749	-0.1425	3
RAB28	0.9649	0.0493	2
RAB2A	0.8727	0.1107	3
RAB30	0.8796	-0.0805	2
RAB33A	0.8328	0.2434	1
RAB33B	0.7713	0.2931	1
RAB35	0.6714	-0.4223	1
RAB37	0.9791	-0.0330	1
RAB39B	0.5002	-0.2786	4
RAB3C	0.7853	0.2423	2
RAB3GAP1	0.3563	-0.2377	2
RAB3GAP2	0.4346	-0.2956	3
RAB3IP	0.9412	0.0451	1
RAB4A	0.9219	0.1019	1
RAB4B	0.7742	0.4083	1
RAB5A	0.6901	-0.5350	1

Table S10: Non-significant synaptic proteins of cerebellar vermis in adults with idiopathic autism (FDR-adjusted p > 0.05) *(continued)*

Gene name	FDR-adjusted p-value	log2(fold-change)	Number of peptides
RAB5B	0.7931	0.1970	3
RAB5C	0.8690	-0.0761	5
RAB6B	0.7362	-0.2120	3
RAB7A	0.7161	0.2204	3
RABGAP1L	0.8796	0.1217	1
RABL3	0.9036	-0.0899	3
RAC1	0.3738	-0.2183	4
RAC3	0.7789	0.2746	1
RAD1	0.9786	-0.0338	1
RAD23A	0.9270	0.0615	1
RALA	0.2850	-0.3950	3
RALGAPA1	0.5135	0.2006	1
RALY	0.3119	-0.4038	5
RALYL	0.9749	-0.0728	1
RAN	0.6005	-0.2496	3
RANBP1	0.9904	-0.0124	2
RANBP2	0.9293	0.2336	1
RANBP3	0.8150	-0.1795	1
RANBP6	0.9808	-0.0553	1
RANGAP1	0.8054	-0.0939	4
RAP1B	0.9462	0.0394	2
RAP1GAP	0.8726	0.0827	2
RAP1GAP2	0.7556	-0.1710	5
RAP1GDS1	0.8606	-0.0583	6
RAP2A	0.9260	0.0635	3
RAPGEF4	0.4210	-0.2861	4
RARS	0.8984	0.0775	4
RASA3	0.5210	-0.3187	3
RASGRF1	0.7912	-0.2020	1
RBBP5	0.8774	0.1469	2
RBFOX3	0.8628	-0.1360	1
RBM12	0.7417	-0.7191	1
RBM14	0.5857	-0.2702	9
RBM17	0.4210	-0.5939	1
RBM22	0.5011	0.4539	1
RBM25	0.8625	-0.1496	1
RBM26	0.9891	0.0077	3
RBM39	0.3090	-0.4317	4
RBMX	0.1828	-0.3254	9
RBMXL1	0.6054	-0.2409	2
RCN1	0.9761	0.0251	2
RDH11	0.6181	-0.3657	3
RDH13	0.8690	-0.3266	1
RDH14	0.6348	-0.2330	1

Table S10: Non-significant synaptic proteins of cerebellar vermis in adults with idiopathic autism (FDR-adjusted p > 0.05) *(continued)*

Gene name	FDR-adjusted p-value	log2(fold-change)	Number of peptides
RDX	0.0845	0.3470	13
RECQL	0.5151	0.4103	1
REEP2	0.4368	-0.2490	3
REEP5	0.9630	0.0346	2
REL	0.5795	-0.4261	1
RELA	0.6828	0.3090	1
RELCH	0.7538	0.2874	1
RELN	0.9356	0.0391	3
REPS1	0.6400	-0.2469	2
REPS2	0.9318	-0.0706	2
RFTN1	0.7457	-0.4084	1
RFTN2	0.9484	-0.0655	1
RGS17	0.4735	-0.6682	1
RGS6	0.8783	0.1635	1
RGS7	0.2722	-0.5484	1
RHEB	0.9808	0.0153	5
RHOT1	0.3935	-0.4249	2
RHOT2	0.7789	-0.1973	2
RIC8A	0.8980	0.0933	1
RIDA	0.7382	-0.3656	2
RIMS1	0.7400	-0.1632	7
RIMS4	0.9812	0.0260	1
RMC1	0.9028	-0.1324	1
RMDN3	0.8322	0.1103	3
RND1	0.6490	-0.7771	1
RNF20	0.7949	-0.2065	2
RNF213	0.9658	0.0604	1
RNF214	0.7989	0.2835	1
RNF40	0.2865	-0.6411	2
RNH1	0.9259	-0.0466	2
RNMT	0.8903	-0.1780	1
RNPS1	0.7940	-0.3550	1
RO60	0.9630	0.0242	3
ROCK2	0.2984	-0.3781	3
ROGDI	0.8679	-0.1051	3
RPA1	0.5577	-0.2577	3
RPA2	0.9809	0.0205	2
RPAP3	0.8861	-0.1294	1
RPL10A	0.9036	-0.0730	2
RPL11	0.9808	-0.0232	4
RPL12	0.7742	0.1293	5
RPL13	0.6348	0.3189	3
RPL13A	0.9809	0.0143	4
RPL14	0.9570	0.0591	1

Table S10: Non-significant synaptic proteins of cerebellar vermis in adults with idiopathic autism (FDR-adjusted p > 0.05) *(continued)*

Gene name	FDR-adjusted p-value	log2(fold-change)	Number of peptides
RPL15	0.9578	0.0505	1
RPL17	0.8986	-0.1376	1
RPL18	0.9164	0.0568	3
RPL18A	0.7523	0.4235	2
RPL19	0.7949	0.2405	1
RPL22	0.9344	-0.0596	2
RPL23	0.7742	-0.1446	4
RPL23A	0.7909	-0.1583	3
RPL24	0.6500	-0.2112	4
RPL27	0.9946	0.0057	1
RPL27A	0.7551	-0.4265	1
RPL28	0.9535	-0.0606	2
RPL29	0.9761	0.0453	3
RPL3	0.7789	0.1192	6
RPL30	0.7949	0.2690	2
RPL31	0.8726	-0.1981	1
RPL34	0.6828	0.4777	3
RPL35	0.9484	0.0651	2
RPL35A	0.9888	0.0178	1
RPL38	0.8342	0.1319	2
RPL4	0.7033	0.2054	6
RPL5	0.8963	-0.1190	1
RPL6	0.9578	0.0233	6
RPL7	0.5551	0.1726	11
RPL7A	0.4837	0.2960	7
RPL8	0.8796	0.1174	2
RPL9	0.8726	0.0820	3
RPLP0	0.7988	0.0910	9
RPLP2	0.5637	0.2224	6
RPN1	0.8455	0.1125	5
RPN2	0.6739	-0.2588	1
RPS11	0.1700	0.3488	5
RPS14	0.9214	-0.1122	1
RPS16	0.7362	0.1955	4
RPS18	0.7742	0.2971	2
RPS19	0.5747	0.2145	6
RPS2	0.7931	-0.1359	4
RPS20	0.9812	-0.0161	1
RPS21	0.8473	-0.2627	1
RPS23	0.6828	-0.4338	1
RPS25	0.7949	0.1540	4
RPS26	0.9270	0.0705	2
RPS28	0.8963	-0.1418	1
RPS3	0.0532	0.3944	8

Table S10: Non-significant synaptic proteins of cerebellar vermis in adults with idiopathic autism (FDR-adjusted p > 0.05) *(continued)*

Gene name	FDR-adjusted p-value	log2(fold-change)	Number of peptides
RPS3A	0.2824	0.4655	5
RPS4X	0.7629	0.1245	5
RPS5	0.8813	0.0792	2
RPS6	0.7949	0.2480	1
RPS6KA3	0.6714	-0.1902	4
RPS6KA4	0.7532	-0.5034	1
RPS6KA5	0.1303	-0.8220	2
RPS7	0.9808	-0.0164	4
RPS8	0.7098	0.1978	3
RPS9	0.8986	0.0776	3
RPSA	0.9109	-0.1070	2
RPTOR	0.7562	0.1999	2
RRAGC	0.9926	0.0130	1
RRAS2	0.4346	-0.5396	1
RRBP1	0.9563	0.0298	8
RSF1	0.5525	-0.7496	1
RSL1D1	0.8118	-0.2209	1
RSU1	0.2722	-0.4010	3
RTCA	0.7243	-0.4555	1
RTCB	0.1386	-0.2870	11
RTF1	0.9808	0.0309	1
RTN1	0.5551	0.2635	2
RTN4	0.7855	-0.1064	4
RTN4R	0.7700	-0.3294	1
RTRAF	0.9133	-0.0640	4
RUFY1	0.2946	0.3612	2
RUFY2	0.8166	-0.1241	1
RUFY3	0.2619	-0.3390	7
RUVBL1	0.3935	0.3272	7
RUVBL2	0.5144	0.3623	4
RWDD1	0.8460	0.0891	1
RXRG	0.5637	-0.5500	1
RYR2	0.3238	-0.3409	5
S100A13	0.6088	0.4170	2
S100A14	0.4368	0.7731	1
S100A6	0.8726	0.2092	1
S100A7	0.5294	0.9941	1
S100A8	0.6079	0.3683	2
S100A9	0.6024	0.8534	1
SACM1L	0.7159	-0.2771	3
SAE1	0.7789	-0.1875	5
SAFB	0.8433	-0.0845	7
SAMD8	0.9857	-0.0376	1
SAMM50	0.7789	0.1530	2

Table S10: Non-significant synaptic proteins of cerebellar vermis in adults with idiopathic autism (FDR-adjusted p > 0.05) *(continued)*

Gene name	FDR-adjusted p-value	log2(fold-change)	Number of peptides
SAP130	0.7362	-0.4272	1
SAP18	0.7949	-0.3943	1
SAR1A	0.7935	-0.1451	3
SARDH	0.8903	-0.1681	1
SARNP	0.6854	-0.2194	3
SARS	0.8680	0.0896	4
SART1	0.7363	-0.5078	1
SART3	0.8727	-0.1496	2
SBDS	0.7795	-0.1514	4
SBF1	0.5151	-0.1704	6
SBF2	0.9777	0.0399	1
SBNO1	0.5932	-0.4517	1
SC5D	0.9761	-0.0242	1
SCAF4	0.6853	0.4598	1
SCAF8	0.7940	0.2977	1
SCAMP1	0.9569	-0.0791	1
SCAMP5	0.8085	0.1911	1
SCCPDH	0.8690	-0.1157	2
SCFD1	0.7556	-0.1278	4
SCGN	0.9166	-0.0830	1
SCIN	0.7243	0.3945	1
SCN2A	0.7173	-0.1025	7
SCNM1	0.8980	0.1454	1
SCO1	0.5005	-0.5893	1
SCO2	0.6714	-0.3512	1
SCRIB	0.3935	-0.3311	1
SCRN1	0.9857	-0.0089	4
SCYL1	0.1312	-0.5283	1
SDHB	0.9624	0.0808	1
SDR39U1	0.7159	0.1709	1
SDSL	0.8330	-0.1916	1
SEC11C	0.8726	0.2013	1
SEC14L2	0.9808	-0.0166	2
SEC16A	0.5385	-0.3061	3
SEC22B	0.7550	-0.1352	6
SEC23A	0.7053	-0.1592	6
SEC24C	0.0888	-0.4471	6
SEC24D	0.9260	0.1195	1
SEC31A	0.9946	0.0030	4
SEC61A1	0.6005	0.4425	1
SEC62	0.9937	0.0050	2
SELENOH	0.5802	0.3788	1
SEMG1	0.6921	0.2969	9
SEMG2	0.7415	0.3393	5

Table S10: Non-significant synaptic proteins of cerebellar vermis in adults with idiopathic autism (FDR-adjusted p > 0.05) *(continued)*

Gene name	FDR-adjusted p-value	log2(fold-change)	Number of peptides
SEPHS1	0.8273	-0.1698	2
SEPTIN10	0.1651	-0.5019	4
SEPTIN2	0.2553	-0.2517	8
SEPTIN7	0.2593	-0.3226	6
SEPTIN8	0.9761	0.0273	3
SERBP1	0.8054	-0.2550	1
SERPINA1	0.4269	0.2647	5
SERPINA12	0.1332	0.5441	5
SERPINA3	0.5932	0.4783	1
SERPINB1	0.7033	0.3740	1
SERPINB12	0.2051	0.6785	3
SERPINB5	0.8575	0.4183	1
SERPINB7	0.4193	0.7757	1
SERPINE2	0.8194	-0.1047	2
SERPINH1	0.8903	-0.1492	1
SESTD1	0.9692	0.0615	2
SETD3	0.8806	0.1630	1
SF3A1	0.7161	-0.1785	4
SF3A3	0.9169	0.0681	3
SF3B2	0.9961	-0.0017	2
SF3B3	0.9205	-0.0363	7
SFPQ	0.1691	-0.3202	10
SFXN3	0.9109	-0.0849	2
SGCD	0.7525	-0.1684	3
SGPL1	0.7611	-0.1556	2
SGSM1	0.7984	-0.2576	1
SGTA	0.7632	-0.3655	1
SH3BGRL	0.7912	0.2410	2
SH3BGRL2	0.8680	-0.1465	1
SH3GL1	0.2619	-0.3200	7
SH3GL2	0.5562	-0.1642	10
SH3GL3	0.5908	-0.1803	6
SH3GLB2	0.7590	-0.2327	2
SHANK1	0.5873	-0.1819	4
SHANK2	0.8841	0.0776	1
SHF	0.9106	0.0786	2
SHMT2	0.1436	-0.3510	6
SHTN1	0.8963	0.1517	1
SIN3A	0.6828	0.2379	2
SIPA1L1	0.5030	-0.8788	1
SIRPA	0.8796	-0.1557	2
SIRT2	0.9064	-0.1102	2
SKIV2L	0.6388	-0.2700	1
SKP1	0.8903	0.0900	1

Table S10: Non-significant synaptic proteins of cerebellar vermis in adults with idiopathic autism (FDR-adjusted p > 0.05) *(continued)*

Gene name	FDR-adjusted p-value	log2(fold-change)	Number of peptides
SLC12A2	0.8690	-0.0912	4
SLC12A5	0.8428	-0.0872	7
SLC12A6	0.9109	0.0609	2
SLC17A7	0.9141	0.2030	1
SLC1A3	0.7912	0.2328	1
SLC25A1	0.8532	-0.1768	1
SLC25A11	0.7789	-0.0927	8
SLC25A12	0.7937	-0.1582	5
SLC25A22	0.2051	-0.4311	4
SLC25A3	0.9804	-0.0136	10
SLC25A4	0.9903	-0.0047	4
SLC25A5	0.9797	0.0159	4
SLC25A6	0.9166	-0.0283	11
SLC27A1	0.8666	0.5252	1
SLC39A10	0.6054	0.6997	1
SLC39A6	0.7550	-0.2213	1
SLC3A2	0.8841	0.1289	2
SLC4A1	0.9900	-0.0107	3
SLC4A10	0.7317	0.3529	2
SLC6A11	0.8504	0.2567	1
SLC8A1	0.6348	0.2219	1
SLC8A2	0.7565	-0.2379	2
SLC9A3R1	0.9146	-0.0763	3
SLIRP	0.5005	-0.5587	1
SLITRK2	0.8796	-0.0869	1
SLITRK4	0.5691	-0.4196	1
SLK	0.5470	-0.2139	4
SLMAP	0.4193	-0.6019	2
SLTM	0.9106	-0.1551	1
SLURP2	0.6714	0.5512	1
SMAD4	0.6652	-0.2975	2
SMAD5	0.9570	-0.1051	1
SMARCA1	0.8726	-0.1066	2
SMARCA2	0.6854	-0.1495	3
SMARCA5	0.8389	-0.1554	2
SMARCC2	0.6542	-0.2781	5
SMARCD1	0.3688	-0.3473	1
SMC1A	0.4804	-0.2165	10
SMC3	0.9658	-0.0425	5
SMNDC1	0.4193	-0.9611	1
SMPD4	0.8841	-0.1545	1
SMS	0.7949	-0.1771	2
SMU1	0.5470	-0.2793	3
SNAP25	0.8448	0.0792	4

Table S10: Non-significant synaptic proteins of cerebellar vermis in adults with idiopathic autism (FDR-adjusted p > 0.05) *(continued)*

Gene name	FDR-adjusted p-value	log2(fold-change)	Number of peptides
SNAP91	0.7713	0.2724	2
SNCA	0.2768	-0.4440	5
SNCB	0.8690	-0.0851	5
SNCG	0.9851	0.0085	6
SND1	0.9904	-0.0041	10
SNRNP200	0.8535	-0.1020	4
SNRPA	0.4094	0.3634	3
SNRPA1	0.5800	0.3456	1
SNRPB	0.9450	0.0640	2
SNRPB2	0.9749	0.0442	1
SNRPC	0.8348	0.3418	1
SNRPD2	0.8553	-0.1311	3
SNRPD3	0.9801	-0.0239	3
SNRPE	0.7556	-0.2543	1
SNTA1	0.8545	-0.1198	4
SNTB1	0.9944	0.0057	4
SNX1	0.7317	-0.1521	3
SNX12	0.9669	0.0130	5
SNX15	0.8273	-0.1770	1
SNX2	0.6578	-0.1603	7
SNX27	0.7957	-0.1592	3
SNX3	0.7417	-0.2942	2
SNX30	0.7523	-0.3837	1
SNX5	0.8903	-0.0986	2
SNX6	0.7556	-0.3779	1
SOD1	0.8054	0.2069	2
SOD2	0.8549	0.2910	1
SOGA1	0.5186	-0.3198	1
SOGA3	0.5302	-0.3362	4
SON	0.6005	-0.3698	2
SORBS1	0.8783	-0.0825	3
SORBS2	0.8796	-0.1265	1
SORCS2	0.9058	-0.1397	1
SORD	0.8296	0.2308	2
SOX11	0.8680	0.2221	1
SPAG1	0.5151	-0.5071	1
SPAG9	0.9166	-0.0648	2
SPART	0.9169	-0.1199	1
SPATA20	0.7949	-0.1416	3
SPECC1L	0.6017	0.3349	2
SPOCK2	0.7058	0.1901	1
SPTA1	0.9210	-0.0469	12
SPTAN1	0.9109	0.0160	50
SPTB	0.2779	-0.2499	15

Table S10: Non-significant synaptic proteins of cerebellar vermis in adults with idiopathic autism (FDR-adjusted p > 0.05) *(continued)*

Gene name	FDR-adjusted p-value	log2(fold-change)	Number of peptides
SPTBN2	0.0845	-0.3339	11
SPTBN4	0.7974	-0.1080	2
SRC	0.1330	-0.9404	1
SRCIN1	0.9761	0.0102	12
SRGAP2	0.4791	-0.3100	5
SRGAP3	0.6691	-0.1322	4
SRI	0.3912	0.6239	1
SRP14	0.8342	-0.2114	2
SRP54	0.8690	0.0558	5
SRP68	0.7363	-0.2129	1
SRP72	0.6984	-0.2255	2
SRP9	0.8780	0.0612	3
SRPK2	0.9351	0.0884	1
SRPRB	0.7481	-0.3638	1
SRRT	0.8233	-0.1057	1
SRSF1	0.8020	0.2464	1
SRSF11	0.7749	-0.3402	1
SRSF5	0.8963	-0.1285	1
SRSF7	0.8726	-0.2385	1
SSB	0.9146	-0.0585	3
SSBP1	0.4102	-0.3969	2
SSR1	0.8041	0.2167	1
SSRP1	0.5679	0.4010	1
ST13	0.7550	-0.1485	6
STAG1	0.9109	-0.1400	1
STAG2	0.8366	-0.0970	2
STAM	0.4791	-0.2953	3
STAT3	0.9487	0.0656	1
STAT6	0.9812	-0.0254	1
STAU2	0.9169	0.0576	3
STIP1	0.4368	0.3488	5
STK33	0.6685	0.6073	1
STK39	0.8645	-0.3279	1
STMN1	0.6955	-0.2902	2
STMN2	0.8903	-0.0735	5
STOM	0.5915	0.3403	2
STRAP	0.4854	-0.4464	2
STRBP	0.5343	-0.3894	2
STRN3	0.8153	0.1964	1
STUB1	0.6735	-0.2813	2
STX12	0.5809	-0.2945	2
STX17	0.6714	-0.1750	1
STX1A	0.9484	0.0590	2
STX1B	0.4094	0.2985	8

Table S10: Non-significant synaptic proteins of cerebellar vermis in adults with idiopathic autism (FDR-adjusted p > 0.05) *(continued)*

Gene name	FDR-adjusted p-value	log2(fold-change)	Number of peptides
STX5	0.7538	0.2655	1
STX6	0.9535	0.0899	1
STXBP3	0.7176	-0.1187	10
STXBP5	0.5551	-0.3685	1
STXBP5L	0.7556	0.5859	1
SUB1	0.9565	-0.0443	1
SUCLA2	0.3411	-0.1965	15
SUCLG1	0.9411	-0.0451	3
SUGP2	0.9630	-0.0314	5
SUMO3	0.7206	-0.2444	1
SUN1	0.8020	0.2410	1
SUPT16H	0.3210	-0.7526	1
SV2B	0.4791	-0.4846	1
SVIP	0.8106	0.2890	1
SYN3	0.7187	-0.2084	2
SYNCRIP	0.8676	-0.1117	2
SYNGR1	0.8507	-0.3151	1
SYNGR3	0.8680	-0.1530	1
SYNJ1	0.8542	-0.0621	14
SYP	0.8680	-0.1113	3
SYT1	0.5551	-0.1832	8
SYT12	0.2294	-0.3550	5
SYT2	0.1607	-0.2853	9
SYT3	0.8690	0.0980	2
SYT7	0.8796	0.1460	1
TAB3	0.5691	0.1992	1
TACO1	0.9651	-0.0493	1
TAGLN	0.1864	-0.2658	6
TALDO1	0.5026	0.2159	7
TAOK1	0.8686	0.2134	1
TARDBP	0.7109	-0.6007	1
TARS	0.6714	-0.1539	7
TBC1D10B	0.7053	-0.2710	1
TBC1D15	0.8676	0.0936	2
TBC1D17	0.8820	-0.1707	1
TBC1D9B	0.8984	-0.1063	1
TBCA	0.8984	0.1833	1
TBCB	0.1806	-0.4478	3
TBCD	0.8054	-0.1626	1
TBP	0.5809	-0.7796	1
TBRG4	0.8375	-0.1790	1
TCAF1	0.9914	0.0089	1
TCEA1	0.4686	-0.4307	2
TCEAL2	0.6867	-0.4020	1

Table S10: Non-significant synaptic proteins of cerebellar vermis in adults with idiopathic autism (FDR-adjusted p > 0.05) *(continued)*

Gene name	FDR-adjusted p-value	log2(fold-change)	Number of peptides
TCERG1	0.3688	-0.4232	2
TCOF1	0.5932	-0.2195	6
TCP1	0.4210	-0.1490	14
TDP2	0.9019	-0.1315	1
TDRKH	0.8690	0.6274	1
TERF2	0.7556	-0.1948	1
TFAM	0.7481	-0.2263	3
TFCP2	0.8433	-0.2565	1
TFG	0.9462	0.0518	2
TGFBRAP1	0.8984	-0.0974	1
TGM1	0.0930	0.6818	3
TGM3	0.1013	0.5244	5
TGM6	0.4131	-0.4514	1
THNSL1	0.9857	0.0107	1
THOC3	0.9662	-0.0235	1
THRAP3	0.6714	-0.2590	2
THUMPD1	0.7988	-0.1518	3
THUMPD3	0.7789	-0.4844	1
THY1	0.5893	0.5092	1
THYN1	0.1436	-0.3583	4
TIAL1	0.3634	-0.5083	1
TIAM1	0.4977	-0.2560	4
TIMM13	0.6312	-0.3590	2
TIMM44	0.5464	-0.2418	2
TIPRL	0.9658	0.0776	1
TJP1	0.9214	-0.0642	1
TJP2	0.9064	-0.0551	4
TKFC	0.9808	-0.0199	1
TKT	0.1256	0.2528	13
TLN1	0.8903	0.0498	14
TLN2	0.2192	-0.1848	17
TMA16	0.9761	-0.0478	1
TMA7	0.6542	-0.4350	1
TMED8	0.8960	0.2103	1
TMEM109	0.4352	0.4940	2
TMEM230	0.9789	-0.0417	1
TMEM263	0.8504	-0.1156	2
TMEM65	0.8273	-0.2065	1
TMEM94	0.7789	-0.3058	1
TMOD1	0.9641	0.0301	3
TMOD2	0.9811	0.0067	9
TMPO	0.7556	-0.7397	1
TMSB4X	0.7565	0.5946	1
TMX2	0.9651	0.0225	2

Table S10: Non-significant synaptic proteins of cerebellar vermis in adults with idiopathic autism (FDR-adjusted p > 0.05) *(continued)*

Gene name	FDR-adjusted p-value	log2(fold-change)	Number of peptides
TNC	0.1700	-0.5476	5
TNKS1BP1	0.8381	-0.2338	1
TNPO1	0.7053	-0.1086	5
TNR	0.4210	-0.1855	11
TNS3	0.7523	-0.1791	1
TOLLIP	0.9271	0.0405	2
TOM1L2	0.4094	0.3102	2
TOMM40	0.7629	-0.4719	1
TOMM5	0.5646	0.5755	1
TOMM70	0.0584	-0.4143	8
TOP1	0.9529	0.0344	6
TOP2B	0.8230	-0.1186	3
TOR1AIP1	0.8020	-0.1253	2
TOX4	0.7415	-0.4190	1
TP53BP1	0.2946	-1.3332	1
TP53I11	0.4131	-0.6890	1
TP53RK	0.8986	0.1115	1
TPD52	0.9629	-0.0233	3
TPD52L1	0.7949	0.2604	1
TPD52L2	0.7415	-0.1649	2
TPI1	0.2124	0.3585	9
TPM1	0.0743	0.3073	9
TPM2	0.7363	0.1418	5
TPM3	0.8903	0.0944	3
TPM4	0.8680	0.0877	5
TPP2	0.9630	0.0282	7
TPPP	0.8680	-0.1205	3
TPPP3	0.9630	0.0446	3
TPR	0.2323	-0.4259	5
TPRG1L	0.8680	-0.1114	1
TRA2B	0.7033	-0.4860	1
TRAF3	0.6017	0.4122	1
TRAP1	0.9293	-0.0545	2
TRAPPC11	0.7538	-0.1872	1
TRAPPC3	0.9312	0.0806	1
TRAPPC4	0.8574	-0.1333	1
TRAPPC6B	0.9292	-0.0814	1
TRAPPC9	0.9109	-0.0496	1
TRIM2	0.2294	-0.3191	5
TRIM25	0.9367	-0.0775	2
TRIM26	0.8273	-0.1919	1
TRIM28	0.5005	-0.2883	6
TRIM3	0.8273	-0.1324	3
TRIM32	0.4854	-0.2104	3

Table S10: Non-significant synaptic proteins of cerebellar vermis in adults with idiopathic autism (FDR-adjusted p > 0.05) *(continued)*

Gene name	FDR-adjusted p-value	log2(fold-change)	Number of peptides
TRIM67	0.9749	0.0252	1
TRIM72	0.9578	-0.0679	3
TRIM9	0.4193	0.3132	1
TRIO	0.8116	-0.0747	10
TRIP11	0.7912	-0.3009	1
TRMT10C	0.5915	-0.4713	1
TRNT1	0.9995	-0.0003	1
TSC1	0.4285	0.4395	1
TSC2	0.3320	-0.6460	1
TSC22D4	0.8679	-0.1179	1
TSPAN14	0.5231	0.9132	1
TSTA3	0.9953	0.0031	1
TTC1	0.8903	-0.0819	1
TTC37	0.8680	-0.0985	1
TTC39A	0.8986	-0.1119	1
TTC7B	0.8963	-0.0683	4
TTC9	0.7550	-0.3514	1
TTC9B	0.7789	-0.1851	1
TTN	0.9356	-0.0685	1
TUBA1C	0.7363	0.1779	5
TUBA3D	0.1533	-0.2430	21
TUBA4A	0.3875	-0.3423	4
TUBA8	0.8679	0.0978	2
TUBAL3	0.7949	-0.1300	2
TUBB	0.6542	-0.2258	6
TUBB2B	0.9106	-0.2080	1
TUBB4A	0.1303	-0.3964	10
TUBB4B	0.7550	-0.3126	2
TUBB6	0.6310	-0.1533	4
TUBB8	0.5470	-0.2584	4
TUBG1	0.8986	-0.1634	1
TUBGCP2	0.9649	-0.0696	1
TUFM	0.5568	-0.1542	11
TWF1	0.8020	0.3722	1
TXN	0.2051	1.0694	2
TXNDC17	0.9561	-0.0685	1
TXNDC9	0.5562	-0.3204	1
TXNL1	0.7481	-0.2612	2
TXNL4A	0.6161	-0.3630	1
TXNRD1	0.5420	0.4229	1
U2AF2	0.7243	-0.1576	4
U2SURP	0.8690	-0.1754	1
UBA1	0.5893	-0.1909	5
UBA2	0.4094	-0.3698	4

Table S10: Non-significant synaptic proteins of cerebellar vermis in adults with idiopathic autism (FDR-adjusted p > 0.05) *(continued)*

Gene name	FDR-adjusted p-value	log2(fold-change)	Number of peptides
UBA3	0.9106	-0.1394	1
UBA52	0.4290	0.2732	3
UBA6	0.5874	-0.2931	3
UBAP2L	0.8690	0.0657	5
UBASH3B	0.9630	0.0284	3
UBE2F	0.7789	0.1920	1
UBE2H	0.7590	-0.2904	1
UBE2K	0.5551	-0.4185	1
UBE2M	0.6388	0.3261	1
UBE2NL	0.6233	0.2901	3
UBE2O	0.8342	0.2447	1
UBE2V1	0.9917	0.0138	1
UBE4A	0.7537	-0.2434	1
UBL5	0.9961	-0.0034	1
UBR4	0.5775	-0.2365	3
UBTF	0.7556	0.5125	1
UCHL1	0.6079	0.1989	6
UEVLD	0.4837	-0.3028	1
UFC1	0.7949	0.2659	1
UFD1	0.7789	-0.2687	1
UFL1	0.4854	-0.3483	2
UGGT1	0.9957	0.0018	3
UGP2	0.8796	-0.0510	14
UHRF1BP1L	0.7742	0.2384	1
ULK3	0.7565	0.2265	1
UMPS	0.7949	0.1721	1
UNC13A	0.8597	-0.0971	3
UNC13C	0.8866	0.1524	1
UNC45A	0.9900	0.0194	1
UPF1	0.7550	-0.1812	4
UPF2	0.5646	-0.2849	1
UQCC1	0.7987	0.2454	1
UQCRB	0.8885	-0.1507	3
UQCRC1	0.9214	-0.1219	1
UQCRC2	0.6542	0.2736	4
UQCRFS1P1	0.9484	0.0558	2
USO1	0.9367	-0.0644	2
USP10	0.6714	0.2254	3
USP15	0.9122	0.1116	1
USP24	0.9484	0.1760	1
USP46	0.7481	-0.1772	1
USP47	0.1828	-0.4510	1
USP5	0.8903	-0.2190	2
USP7	0.3646	-0.4129	2

Table S10: Non-significant synaptic proteins of cerebellar vermis in adults with idiopathic autism (FDR-adjusted p > 0.05) *(continued)*

Gene name	FDR-adjusted p-value	log2(fold-change)	Number of peptides
USP8	0.5302	-0.3701	1
USP9X	0.5809	-0.1975	3
UXT	0.9417	-0.1241	1
VAC14	0.8330	-0.1590	3
VAMP2	0.7611	0.4831	1
VAPA	0.9963	0.0019	2
VAPB	0.3278	-0.3750	4
VARS	0.9146	0.0839	2
VASP	0.8813	0.2308	1
VAT1	0.4346	0.8980	1
VAT1L	0.7550	-0.1182	13
VBP1	0.9791	-0.0142	2
VCP	0.8690	0.0725	10
VCPIP1	0.8905	-0.0785	3
VDAC1	0.1700	-0.3825	13
VDAC2	0.3575	-0.3271	10
VDAC3	0.4187	-0.3536	8
VIM	0.7789	0.1867	2
VPS13A	0.8448	0.1235	1
VPS16	0.9617	0.0492	1
VPS25	0.5444	0.4919	1
VPS26A	0.9260	0.0723	1
VPS29	0.9946	0.0024	2
VPS33A	0.5592	-0.3240	2
VPS35	0.8628	-0.0986	4
VPS35L	0.7945	-0.3896	1
VPS39	0.9581	0.0454	1
VPS41	0.8986	0.0834	2
VPS45	0.8153	-0.1448	4
VPS50	0.7565	-0.2556	1
VPS51	0.7400	-0.3013	2
VPS53	0.7989	-0.1157	3
VPS8	0.8054	-0.0930	1
VSNL1	0.9781	-0.0214	4
VTI1B	0.7668	0.1890	2
VWA8	0.8436	-0.1914	2
WASF3	0.9946	-0.0029	3
WASH4P	0.8680	-0.1775	1
WASHC5	0.8574	0.3717	1
WASL	0.6931	-0.3802	1
WDR11	0.8645	0.1390	2
WDR13	0.6079	-0.3084	1
WDR17	0.8418	-0.2005	1
WDR33	0.9106	0.3842	1

Table S10: Non-significant synaptic proteins of cerebellar vermis in adults with idiopathic autism (FDR-adjusted p > 0.05) *(continued)*

Gene name	FDR-adjusted p-value	log2(fold-change)	Number of peptides
WDR37	0.6181	-0.5462	1
WDR45	0.9462	-0.0386	2
WDR45B	0.7949	-0.2504	1
WDR47	0.5470	-0.3886	2
WDR61	0.5481	0.5880	1
WDR7	0.8406	-0.0713	5
WDR87	0.8512	0.2523	1
WDR91	0.5857	-0.3606	1
WDTC1	0.6828	-0.3774	1
WIPF2	0.9417	-0.0984	1
WIPI2	0.7949	0.1602	3
WNK2	0.9929	-0.0058	1
WRNIP1	0.9809	-0.0205	2
WTAP	0.6850	0.3461	1
XPNPEP1	0.9873	0.0176	1
XPO1	0.2252	-0.3086	7
YARS	0.6828	-0.1411	6
YES1	0.9270	-0.0616	4
YKT6	0.7362	0.1343	4
YWHAB	0.5788	-0.1910	6
YWHAE	0.5562	0.1848	12
YWHAG	0.4193	-0.3128	7
YWHAH	0.9791	0.0111	6
YWHAQ	0.8680	0.2714	1
YWHAZ	0.7853	-0.0772	13
YY1	0.8806	-0.3390	1
ZC2HC1A	0.6714	0.2210	3
ZC3H15	0.7363	-0.3062	2
ZCWPW1	0.9761	0.0299	1
ZFR	0.9808	-0.0164	5
ZFYVE1	0.9351	0.0726	1
ZFYVE21	0.7789	-0.3177	1
ZG16B	0.4193	0.5126	2
ZMIZ2	0.1438	-0.9625	1
ZNF326	0.9812	-0.0232	1
ZNF512	0.9662	0.0442	1
ZNF516	0.7795	0.3178	1
ZNF532	0.8233	0.5838	1
ZNF638	0.5761	-0.2578	3
ZRANB2	0.9826	-0.0185	1
ZW10	0.6079	-0.2990	1
ZYX	0.5079	-0.4274	1
ZZEF1	0.8054	-0.2520	1
